# Causal relationship between modifiable risk factors and knee osteoarthritis: a Mendelian randomization study

**DOI:** 10.3389/fmed.2024.1405188

**Published:** 2024-09-02

**Authors:** Zhihao Diao, Danyang Guo, Jingzhi Zhang, Ruiyu Zhang, Chunjing Li, Hao Chen, Yuxia Ma

**Affiliations:** ^1^School of Acupuncture and Tuina, Shandong University of Traditional Chinese Medicine, Jinan, China; ^2^The First Clinical Medical College, Shandong University of Traditional Chinese Medicine, Jinan, China; ^3^Complutense University of Madrid, Madrid, Spain

**Keywords:** Mendelian randomization, knee osteoarthritis, risk factors, causal relationship, genetic variants

## Abstract

**Background:**

While several risk factors for knee osteoarthritis (KOA) have been recognized, the pathogenesis of KOA and the causal relationship between modifiable risk factors and KOA in genetic epidemiology remain unclear. This study aimed to determine the causal relationship between KOA and its risk factors.

**Methods:**

Data were obtained from published Genome-Wide Association study (GWAS) databases. A two-sample Mendelian randomization (MR) analysis was performed with genetic variants associated with risk factors as instrumental variables and KOA as outcome. First, inverse variance weighting was used as the main MR analysis method, and then a series of sensitivity analyses were conducted to comprehensively evaluate the causal relationship between them.

**Results:**

Univariate forward MR analysis revealed that genetically predicted hypothyroidism, hyperthyroidism/thyrotoxicosis, educational level, income level, metabolic syndrome (MS), essential hypertension, height, hot drink temperature, diet (abstaining from sugar-sweetened or wheat products), and psychological and psychiatric disorders (stress, depression, and anxiety) were causally associated with KOA. Reverse MR exhibits a causal association between KOA and educational attainment. Multivariate MR analysis adjusted for the inclusion of potential mediators, such as body mass index (BMI), smoking, alcohol consumption, and sex, exhibited some variation in causal effects. However, hyperthyroidism/thyrotoxicosis had a significant causal effect on KOA, and there was good evidence that height, hypothyroidism, educational level, psychological and psychiatric disorders (stress, depression, and anxiety), and abstaining from wheat products had an independent causal relationship. The mediating effect of BMI as a mediator was also identified.

**Conclusion:**

This study used MR to validate the causal relationship between KOA and its risk factors, providing new insights for preventing and treating KOA in clinical practice and for developing public health policies.

## 1 Introduction

Knee osteoarthritis (KOA) is a common type of osteoarthritis (1), and its clinical manifestations include chronic knee pain, limited activity, and dysfunction (2). KOA affects approximately 16% of the population worldwide, and the number of new cases of KOA reached 86.7 million in 2020 (3, 4). Therefore, KOA significantly contributes to disability (5), imposing substantial social and economic burdens and presenting a significant challenge to global public health (6, 7). The pathogenesis of knee osteoarthritis remains unclear. Previous observational studies have found increasing evidence that risk factors such as educational level, economic level (8, 9), metabolic syndrome (MS), essential hypertension (10), thyroid dysfunction (11), diet (12), hot drink temperature (13), height (14), and psychological and mental diseases (such as stress, depression, and anxiety) (15) are associated with the pathogenesis of KOA. Despite this relationship, the causal association obtained from observational studies of traditional epidemiology may be challenging and cannot be used as reliable evidence because residual confounding factors and reverse causality may lead to bias in traditional observational studies (16). Concurrently, large-sample randomized controlled studies are expensive and time-consuming, making them difficult to use in practice.

Mendelian randomization (MR) is an emerging method for inferring causal associations in genetic epidemiology that provides reliable evidence for inferring causal effects between risk factors and outcomes using genetic variants from genome-wide association studies (GWAS) as instrumental variables (IV) (17). Because human genetic variation is characterized by random allocation, irreversibility, and fixity, MR can be used to effectively avoid the influence of confounding factors and reverse causality (18). Thus, univariate MR could be used to estimate the direct causal association between each risk factor and KOA. Multivariate MR is an extension of MR and has great advantages in avoiding unobserved confounding factors and collider biases. It can assess direct effects even when a single nucleotide polymorphism (SNP) is associated with multiple exposures. Multivariate MR can also be used to infer the potentially causal relationship between risk factors and KOA and the effect of mediating factors (19–21). Therefore, this study aimed to investigate the relationship between KOA and thyroid dysfunction, educational level, economic income, MS, essential hypertension, diet, height, hot drink temperature, and psychological and mental disorders (tension, depression, and anxiety) using MR, from the perspective of genetic inheritance.

## 2 Methods

### 2.1 Study design

**Figure 1:**
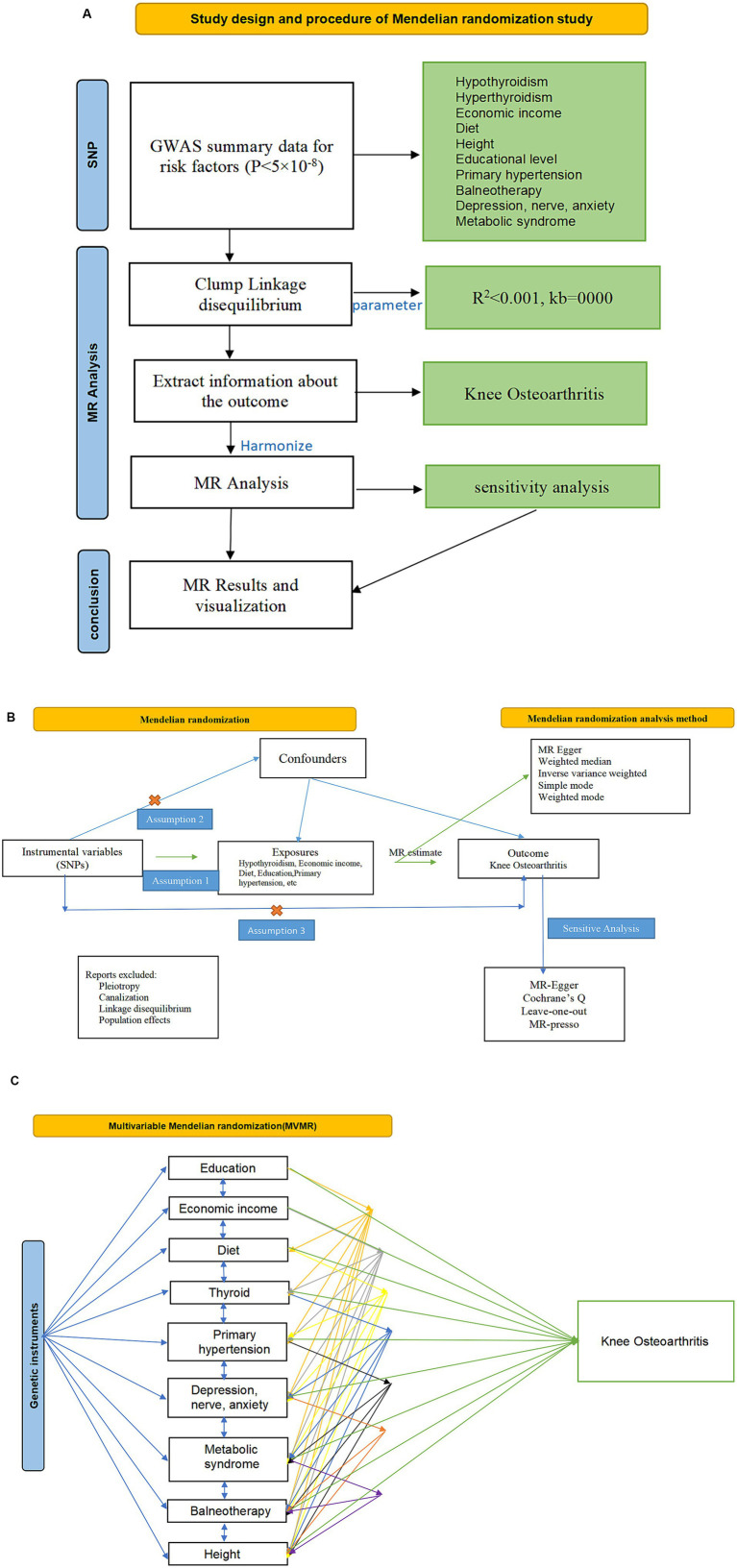
Schematic diagram of Mendel randomization research design process. **(A)** Flow chart of Mendelian randomization. **(B)** Univariate two-sample Mendelian randomization analysis of risk factors and KOA. **(C)** Multivariate Mendelian randomization analysis of risk factors and KOA. SNP, single nucleotide polymorphism; KOA, knee osteoarthritis; MR, Mendelian randomization.

This study follows the Strengthening the Reporting of Observational Studies in Epidemiology using MR (STROBE-MR) reporting guidelines (22, 23) (Supplementary Table S1). In this study, genetic variants significantly associated with exposures were selected as IV to infer causal relationships between exposures and outcomes, and we chose SNPs significantly associated with each risk factor as IVs for the MR analyses. To ensure the validity of IVs, we needed to satisfy the three assumptions of relevance, independence, and exclusivity for MR. Firstly, the genetic variants should be directly associated with the exposures in question. Second, genetic variants should be uncorrelated with any of the other confounders. Third, genetic variants should not be directly related to the outcome (KOA), but should only influence the outcome through exposure (16). In this study, SNPs were obtained from published GWAS data, and causal and sensitivity analyses were performed. Figure 1 shows a schematic of this process.

All data in this study were obtained from published data, and ethical approval for each study was obtained from the appropriate ethics committee. The current study was a secondary analysis of the data and did not therefore require ethical approval. In this study, three types of analyses–namely, two-sample bidirectional MR, multivariate MR, and mediation analyses–were conducted as research methods.

### 2.2 Data sources

All GWAS data used in this study are available from the IEU OpenGWAS project^1^ and FinnGen alliance.^2^ The pooled KOA data used in this study were obtained from a large GWAS conducted by Tachmazidou et al. (24), which included 24,955 cases and 378,169 controls from England, Scotland, Wales, and Northern Ireland. Additionally, meta-analysis was performed using GWAS data from the United Kingdom Biobank and arcOGEN database. Based on a comprehensive consideration of previous literature review and clinical observation, the risk factors for KOA included in this study were hyperthyroidism, hypothyroidism, economic income, diet (abstaining from sugar-sweetened or wheat products), height, essential hypertension, educational level, hot drink temperature, psychological and mental disorders (stress, depression, and anxiety), and MS. Educational level was included in both subtypes. Diseases associated with these risk factors were diagnosed according to the International Classification of Diseases-10th Revision criteria. Hot drink temperature was derived from a questionnaire containing the following main question: ‘What hot drinks do you prefer (e.g., coffee or tea)?’, with the response options being ‘very hot’, ‘hot’, ‘mildly hot’, and ‘non-hot’ drinks (13). Additionally, genetic variation data on risk factors were provided by the FinnGen Consortium, Medical Research Council Integrative Epidemiology Unit, United Kingdom Biobank, Neale Lab, and Science Genetic Association Consortium (Supplementary Table S2). The present study also included potentially relevant mediators such as BMI, alcohol consumption, smoking, and sex (25–27), with BMI and smoking coming from the United Kingdom Biobank’s big data analysis and with alcohol consumption from the GWAS and Sequencing Consortium of Alcohol and Nicotine use. Pooled data on sex were from the combined analysis data of five different cohorts (23andMe, United Kingdom Biobank, iPSYCH, FinnGen, and Biobank Japan), totalling 3,309,398 samples by Pirastu et al. (28).

### 2.3 Genetic instrument selection

Independent SNPs with thresholds smaller than the genome-wide significance threshold (5 × 10^−8^) were selected as IV to ensure the authenticity and reliability of the causal relationship between risk factors and KOA. Simultaneously, to avoid bias caused by linkage disequilibrium among IVs, we used the TwoSampleMR package in R to remove linkage disequilibrium (*R*^2^ < 0.001 and clumping distance = 10,000 kb) (29), resulting in a total of 2039 SNPs. The F statistic was employed to evaluate the effects of weak instrumental variables. When *F* > 10, there is no bias caused by the influence of weak IV (30). The *F* values of the SNPs in this study were > 29.69, providing sufficient evidence that the strong association will not introduce bias. SNP summary data for risk factors are presented in Supplementary Table S3. To ensure that the SNP effects on exposure and outcome correspond to the same alleles and to avoid any distortions in strand orientation or allele coding (31), we excluded SNPs with incompatible alleles and those exhibiting palindromic structures with intermediate allele frequencies. Finally, 14 incompatible alleles and 57 SNPs with palindromic structures were excluded (Supplementary Table S4).

### 2.4 Statistical analysis

#### 2.4.1 Univariate and multivariate MR analyses

A univariate two-sample bidirectional MR analysis was performed to clarify the direct genetic causality between KOA and its risk factors. In this study, the Wald ratio method was first used to test the causal effect size of each IV on KOA and provide evidence for the identified association (32). Multiple independent and valid IVs were included, and inverse variance weighting (IVW) was used as the main analysis method to test causality. Additionally, to prevent the influence of unknown confounding factors and ensure the reliability of the results, four supplementary analysis methods—weighted median estimation, MR-Egger regression, simple mode, and weighted mode—were used for verification (33). The premise of IVW analysis is that there is no pleiotropy in the IV. Fixed effect IVW is usually used when all included SNPs are valid, and random effect IVW is used only when there is obvious heterogeneity among the SNPs (34–36). When the instrumental strength meets the requirement of independence from the direct effect, the MR-Egger regression test can provide stronger proof for the causal estimate. Additionally, when the intercept of the MR-Egger regression is infinitely close to zero, the result is infinitely close to the IVW (37). Even if up to 50% of the IVs are invalid, the weighted median estimation can still provide robust analytical results (38). Simple and weighted modes ensure that when some IVs are invalid, the results will not be distorted by the influence of bias (39). To further assess the direct causality, mediating effects, and potential horizontal multidirectionality between KOA and thyroid dysfunction, income, educational level, diet, mental disorders, height, essential hypertension, multiple sclerosis, and other risk factors, all factors (including the four mediators of BMI, smoking, drinking, and sex) were incorporated in the same model, and multivariate MR analysis was conducted based on linear weighted regression IVW and MR-Egger methods to jointly estimate their causal effects on the risk of KOA. However, because more exposures are likely to cause collinearity, the mv-lasso function was applied to remove unnecessary exposures and correct the results. Additionally, the formula $F=\left(N-k-1\right)/k\times R2/\left(1-R2\right)$ was used to determine whether a weak instrument bias influenced the *F*-value measurement.

#### 2.4.2 Mediation analysis

Multivariate MR results indicated that BMI might be a mediator; hence, mediation analysis was performed using R software in order to further evaluate the mediating effect and mediator share of BMI in the causal relationship between KOA and the risk factors. First, two-sample MR was conducted to ensure that there was a causal relationship between exposure and BMI and between BMI and KOA, and the effect value between exposure and BMI (BetaXZ) was then calculated. Second, multivariate MR was used to examine the effect of BMI as a potential mediator on KOA (BetaZY) and the adjusted effect value between exposure and KOA (BetaXY1). Finally, univariate MR was performed to evaluate the causal effect between exposure and KOA (BetaXY) and to calculate the mediating effect (BetaXZ×BetaZY) and the share of the mediating effect (BetaXZ×BetaZY/BetaXY). Additionally, the coefficient product test was used to calculate the mediating effect and its confidence interval (CI).

#### 2.4.3 Sensitivity analyses

Sensitivity analyses can be used to test whether the causal associations from MR analyses are robust. First, heterogeneity among the IVs was assessed by calculating Cochran’s Q value; if significant heterogeneity existed, a random-effects model was used (29). Second, MR-Egger regression was performed, and the *p* value of its intercept was used to test horizontal pleiotropy. Leave-one-out tests were performed to assess whether causal associations were driven by a specific SNP (40). Finally, using MR-PRESSO detection, we re-examined whether level pleiotropy existed, removed the outliers, and corrected the causal effect values (41). We also used R language to visualize the MR analysis results, including scatter, forest, and funnel plots and sensitivity analysis. All statistical analyses were performed using the ‘TwoSampleMR’, ‘MR-PRESSO’, ‘MendelianRandomization’, and ‘MVMR’ packages of R software version 4.2.2 (The R Foundation for Statistical Computing, Vienna, Austria), with two-sided *p* values <0.05 being considered statistically significant.

## 3 Results

### 3.1 Causal effects of risk factors and KOA

**Figure 2:**
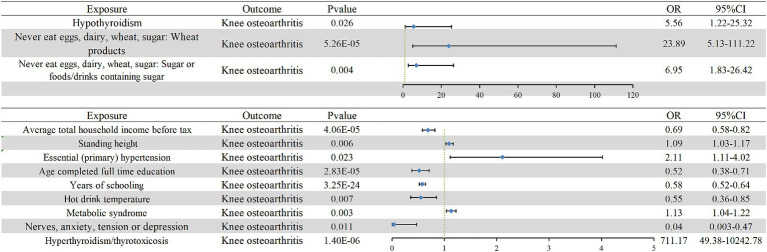
Univariate Mendelian randomization results for the causal relationship between KOA and its risk factors. The blue diamond and error line indicate the odds ratio and 95% confidence interval, respectively. OR, odds ratio.

Univariate MR analysis revealed that genetically predicted hypothyroidism, hyperthyroidism/thyrotoxicosis, economic income, diet (abstaining from sugar-sweetened or wheat products), height, essential hypertension, educational level, hot drink temperature, mental disorders (stress, depression, and anxiety), and MS were causally associated with KOA (Supplementary Figure S1). The results indicated that hypothyroidism (odds ratio (OR): 5.56, 95% CI: 1.22–25.32, *p* = 0.026), hyperthyroidism/thyrotoxicosis (OR: 711.17, 95% CI: 49.38–10242.78, *p* = 1.40E-06), abstaining from wheat products (OR: 23.89, 95% CI: 5.13–111.22, *p* = 5.256E-05), abstaining from sugar-sweetened products (OR: 6.95, 95% CI: 1.83–26.42, *p* = 0.004), height (OR: 1.09, 95% CI: 1.03–1.17, *p* = 0.006), primary hypertension (OR: 2.11, 95% CI: 1.11–4.02, *p* = 0.023), and MS (OR: 1.13, 95% CI: 1.04–1.22, *p* = 0.003) increased the risk of KOA, whereas income (OR: 0.69, 95% CI: 0.58–0.82, *p* = 4.062E-05), educational level (OR: 0.52, 95% CI: 0.38–0.71, *p* = 2.829E-5), years of schooling(OR: 0.58, 95% CI: 0.52–0.64, *p* = 3.254E-24), hot drink temperature (OR: 0.55, 95% CI: 0.36–0.85, *p* = 0.007), and psychiatric disorders such as stress, depression, and anxiety (OR: 0.37, 95% CI: 0.003–0.47, *p* = 0.011) reduced the risk of KOA (Figure 2; Supplementary Table S5; Supplementary Figure S2). In the reverse MR analysis, KOA was only associated with educational level (OR: 0.96, 95% CI: 0.93–0.99, *p* = 0.011), and no significant evidence was obtained for the causal relationship or association of KOA with hypothyroidism (*p* = 0.362), hyperthyroidism/thyrotoxicosis (*p* = 0.662), income (*p* = 0.171), abstinence from sugar-sweetened products (*p* = 0.228), abstaining from wheat products (*p* = 0.651), essential hypertension (*p* = 0.533), MS (*p* = 0.675), height (*p* = 0.148), hot drink temperature (*p* = 0.823), and mental disorders (stress, depression, and anxiety) (*p* = 0.447).

### 3.2 Multivariate MR analysis

**Figure 3:**
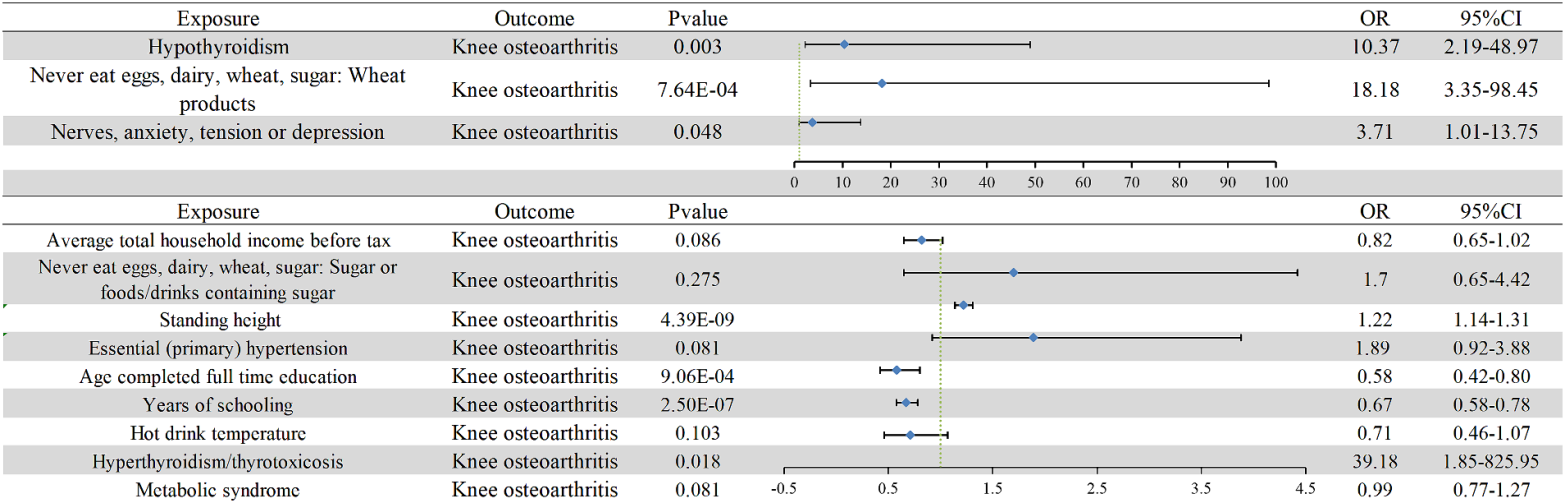
Multivariate Mendelian randomization results for the direct causal relationship between KOA and its risk factors. The blue diamond and error line indicate the odds ratio and 95% confidence interval, respectively. OR, odds ratio.

In the multivariate MR model, owing to the potential collinearity problem, we used the mv-lasso function to perform a lasso test (collinearity correction) to correct outliers (34). After incorporating the mediators of BMI, smoking, alcohol consumption, and sex, the adjusted results indicated that hyperthyroidism/thyrotoxicosis (OR: 39.18, 95% CI: 1.85–825.95) had a significant causal relationship with KOA. Sufficient evidence also suggested that hypothyroidism (OR: 10.37, 95% CI: 2.19–48.97), abstaining from wheat products (OR: 18.18, 95% CI: 3.35–98.45), mental disorders (stress, depression, and anxiety) (OR: 3.71, 95% CI: 1.01–13.75), height (OR: 1.22, 95% CI: 1.14–1.31), and educational level (OR: 0.58, 95% CI: 0.42–0.80; OR: 0.67, 95% CI: 0.58–0.78) were casually associated with KOA, whereas essential hypertension (OR: 1.89, 95% CI: 0.92–3.88), MS (OR: 0.99, 95% CI: 0.77–1.27), income (OR: 0.82, 95% CI: 0.65–1.02), hot drink temperature (OR: 0.71, 95% CI: 0.46–1.07), and abstaining from sugar-sweetened products (OR: 1.7, 95% CI: 0.65–4.42) were not statistically significant. Therefore, there was no evidence of a direct effect on the incidence of KOA (Figure 3). In addition, we found in a multivariate MR study that adjusted for smoking, alcohol consumption, and sex were not mediators between risk factors and KOA, and that the true mediator of significance was BMI.

### 3.3 Sensitivity analyses

The F-statistic for the genetic instrument ranged from 29.69 to 1976.82 (Supplementary Table S3). Therefore, there was sufficient evidence to suggest that a weak instrument bias was unlikely. Heterogeneity tests revealed potential heterogeneity in the causal effect estimates between KOA and hypothyroidism, abstaining from wheat and sugar-sweetened products, height, essential hypertension, educational level, hot drink temperature, MS, and psychiatric disorders (Supplementary Figure S3). A random-effects model was used to estimate the MR effect size, and the results suggested a causal relationship (*p* < 0.05). Based on the evidence of the pleiotropic effect of essential hypertension on KOA, determined by means of the MR-Egger’s intercept test, we repeated the MR-PRESSO test after deleting the outliers in the IV, and no significant pleiotropic effect was observed (Supplementary Table S6). Finally, leave-one-out analysis showed that the causal effect between risk factors and KOA was robust (Supplementary Figure S4).

### 3.4 BMI mediated The genetic predictive effect of risk factors On KOA

Mediation analysis revealed that genetically predicted BMI mediated income (mediation effect = −0.2736; 95% CI: −0.3138, −0.2334; mediated proportion = 73.99%), abstaining from wheat products (mediation effect = 0.9789; 95% CI: 0.6204, 1.3374; mediated proportion = 30.85%), abstinence from sugar-sweetened products (mediation effect = 1.1101; 95% CI: 0.5556, 1.6646; mediated proportion = 57.27%), height (mediation effect = −0.0717; 95% CI: −0.1142, −0.0292; mediated proportion = 99.18%), educational level (mediation effect = −0.2774; 95% CI: −0.3172, −0.2376; mediated proportion = 42.66%; mediation effect = −0.2442; 95% CI: −0.2755, −0.2129; mediated proportion = 44.18%), mental disorders (nerves, anxiety, tension, or depression) (mediation effect = −0.675; 95% CI: −1.0436, −0.3064; mediated proportion = 20.47%), and KOA.(Table 1).

**Table 1:** BMI mediated the genetic predictive effect of risk factors on KOA.

Exposure	Intermediary factors	Outcome	Mediation effect	Proportion of mediation effect	95%CI
Average household income before taxes	BMI	KOA	−0.2736	73.99%	−0.3138, −0.2334
Never eat eggs, dairy, wheat, sugar: Wheat products	BMI	KOA	0.9789	30.85%	0.6204, 1.3374
Never eat eggs, dairy, wheat, sugar: Sugar or foods/drinks containing sugar	BMI	KOA	1.1101	57.27%	0.5556, 1.6646
Standing height	BMI	KOA	−0.0717	99.18%	−0.1142, −0.0292
Age at completion of full-time education	BMI	KOA	−0.2774	42.66%	−0.3172, −0.2376
Education level	BMI	KOA	−0.2442	44.18%	−0.2755, −0.2129
nerves, anxiety, tension or depression	BMI	KOA	−0.6750	20.47%	−1.0436, −0.3064

## 4 Discussion

Currently, the prevention, diagnosis, and treatment of KOA are major challenges for the public health system. In this study, based on large GWAS data, we used an MR analysis system to verify the causal association between 12 KOA-related risk factors and KOA and to determine whether this relationship could be trusted. Single-sample MR analysis based on genetic predictions revealed that hypothyroidism, hyperthyroidism/thyrotoxicosis, income, educational level, height, hot drink temperature, diet (abstaining from wheat and sugar-sweetened products), psychiatric disorders (stress, depression, and anxiety), and KOA risk were causal factors. MVMR analyses showed that the causal effects somewhat changed after the inclusion of BMI, sex, smoking, and alcohol consumption and that hyperthyroidism/thyrotoxicosis, essential hypertension, educational level, abstaining from wheat products, and income remained to be significantly causally associated with KOA and could directly influence KOA, whereas height, hot drink temperature, hypothyroidism, abstinence from sugar-sweetened products, depression, and other psychological factors were not directly causally associated with KOA. A mediating factor, BMI, was also identified.

### 4.1 Thyroid function and KOA

Few observational studies have examined the association between thyroid function and the incidence of KOA. In the Framingham Osteoarthritis Study of 1996, involving 798 women and 577 men, no association was observed between serum thyroid-stimulating hormone (TSH) concentration and KOA. Therefore, there was no evidence that thyroid function was associated with KOA. However, this study only considered serum TSH concentration over a certain period and ignored the development and changes in TSH levels (42, 43). Our MR study made important adjustments, and the results showed that there was a significant positive correlation between hyperthyroidism or hypothyroidism and KOA. A recent retrospective study of 109 patients with thyroid dysfunction who underwent musculoskeletal ultrasound (MSUS) examination and clinical evaluation showed that the knee joint effusion rate, overall MSUS severity score, Visual Analog Scale score, and abnormal frequency of MSUS were significantly higher in patients with thyroid dysfunction, and the imaging examination results were consistent with the clinical evaluation. Therefore, hypothyroidism and hyperthyroidism are causally associated with KOA and could increase the risk of KOA (11). The potential mechanism may be that TSH affects the synthesis of hyaluronic acid and proteoglycans, and increases the viscosity of the knee synovial fluid, leading to KOA-related symptoms (44, 45). Another prospective cohort study also demonstrated that hypothyroidism could cause knee joint degeneration through chondrocalcinosis (46), which is consistent with the results of our MVMR analysis.

### 4.2 Hypertension, MS, and KOA

Hypertension is an important risk factor for KOA (47, 48), and some recent studies have identified MS as a new risk factor for KOA, which has increasingly gained attention (49, 50). Univariate MR results indicated a causal relationship between MS, hypertension, and KOA; in contrast, multivariate MR analysis showed no statistical significance, which was a somewhat surprising result. We randomly performed mediation analysis and found that BMI mediated the association between MS, essential hypertension, and KOA. A large meta-analysis of four databases–EMBASE, PubMed, Cochrane Library, and MEDLINE–and conference materials, including 1,609 articles, showed that KOA was positively correlated with MS (OR: 1.41, 95% CI: 1.16–1.73) and hypertension (OR: 1.70, 95% CI: 1.411–2.052) in radiology studies and with MS (OR: 1.17, 95% CI: 1.03–1.33) and hypertension (OR: 1.32, 95% CI: 1.18–1.47) in symptomatic studies (10). A previous meta-analysis combined with MR analysis highlighted that hypertension increased the incidence of KOA by 62% and that the association between hypertension and KOA persisted even when the association strength decreased from 3.06 to 1.42 after adjustment for body mass index (BMI) (51). The underlying mechanism may be that hypertension may cause intraosseous hypertension, leading to arterial and venous blockage, reduced bone blood flow, subchondral bone ischaemia, and bone cell apoptosis. Therefore, osteoclasts mediate bone resorption, destroy the mechanical support of the covering cartilage, interfere with the exchange of gasses and metabolites in the bone-cartilage functional unit, and thus induce KOA (52, 53). Concurrently, hypertension can also damage vascular endothelial cells and promote the secretion of prostaglandins, which leads to joint inflammation and cartilage damage (54). A cohort study based on baseline data and controlling for covariates such as sex, race, and BMI not only demonstrated a causal relationship between hypertension and KOA but also demonstrated an association between hypertension and pain in patients with KOA (55). MS is a series of diseases caused by abnormal human metabolism, including central obesity, dyslipidaemia, and insulin resistance (56). However, the mechanisms underlying the relationship between MS and KOA remain unclear. Inflammatory mechanisms, oxidative stress, the accumulation of advanced glycation end products (AGE), and ectopic lipid deposition in chondrocytes caused by abnormal lipid metabolism can cause KOA (57). Recent studies have shown that macrophages play a key role in this process. On the one hand, MS can promote the polarization of M1 macrophages by acting on AGE and free fatty acids (FFAs) of macrophages, and AGE can increase the transcription of interleukin (IL)-1β by regulating the NF-κB pathway. FFAs bind to toll-like receptor 4 to release pro-inflammatory factors (58, 59), whereas the adipokine leptin activates the JAK2-STAT3 and PI3K-AKT–mTOR pathways in macrophages, promoting a pro-inflammatory phenotype through the secretion of tumor necrosis factor (TNF)-α and IL-1β (60, 61), resulting in chronic inflammatory hyporesponse and cartilage destruction or deformation. On the other hand, MS can increase chondrocyte degradation through AMPK activity inhibition, whereas excessive mTOR activation inhibits chondrocyte autophagy, preventing self-repair and causing knee chondrocyte damage and KOA (62–64).

### 4.3 Educational attainment, income, and KOA

With respect to the social economy, educational level and income are closely related to KOA onset (9). This is supported by the results of the univariate MR analysis in the present study, which indicated that educational level and income were negatively associated with KOA onset. Nevertheless, no independent effect of income on KOA was observed in multivariate MR, which may be mediated by BMI. A study based on data from the Korean National Health and Nutrition Examination Survey (KNHANES V) found that the risk of KOA was 1.5 times higher for people with low income than for those with high income, and 2.6 times higher for those with a low educational level than for those with college or higher education. These findings did not vary based on sex. The results remained significant after adjusting for confounding factors such as age and BMI, and the imaging findings provided evidence supporting this idea (65). This point of view has also been confirmed in relevant studies in China, the United States, Denmark, and Japan (66–69). The underlying reason may be that people with a lower relative income engage in relatively heavy physical labor, experience more serious wear on the knee joint, and are less willing to seek medical treatment when they experience discomfort in the early stage (66). Educational level was generally positively correlated with income. However, this correlation was not absolute. People with low educational levels are less aware of KOA and related health policies, which cannot be effectively prevented. This is also a potential cause of KOA development and aggravation (70).

### 4.4 Depression and KOA

Previous studies have found that mental disorders such as depression increase the risk of KOA (71, 72). This was supported by a recent national cohort study, which showed that depression increased the risk of KOA at baseline over a 4-year follow-up and found a bidirectional association between depression and KOA (73). The association between depression and KOA may be explained by the inflammatory immune mechanism, which is related to the production and release of TNF-α, IL-1β, IL-6, and IL-8 (74, 75). Additionally, the proposed bone-brain axis may provide new insights into this association (76). Our MR results showed that psychological factors such as depression were negatively associated with KOA. We also performed reverse MR; however, this was contrary to the above clinical observations and did not achieve the expected results. Therefore, more rigorous clinical trials in genetics should be conducted to verify this association, which is a new direction for future in-depth research.

### 4.5 Diet and KOA

In this study, we mainly studied the consumption of whole wheat and its products and the consumption of sugar-sweetened products. Univariate and multivariate MR revealed a significant positive correlation between abstaining from whole wheat and its products and the risk of KOA. In other words, the intake of wheat and its derivative products was negatively correlated with the risk of KOA. The results of previous studies were consistent with our MR analysis results (77). Wheat is an important source of dietary grain fiber (78, 79), and it is also part of the grains included in the Mediterranean diet, which can reduce the risk of KOA owing to its high dietary fiber content (80, 81). Presently, there is limited research on the mechanism linking wheat and its products to KOA; however, the whole wheat diet may be associated with a reduced inflammatory response (82), and clinical trials have demonstrated that it can lower blood lipid and cholesterol levels (83), which could impact KOA. The univariate MR analysis of genetic prediction also showed a causal association between sugar deprivation and KOA; however, the multivariate MR analysis showed that this association was not obvious. Therefore, we believe that this association can be due to the existence of mediating factors. The mechanism underlying the relationship between glucose deprivation and KOA remains unclear. Current studies have shown that articular cartilage is an indispensable part of the knee joint, where only chondrocytes reside. The metabolic homeostasis of chondrocytes is related to the structure and function of cartilage tissue. Chondrocytes are required to obtain glucose and oxygen from the subchondral valley and synovial fluid for glucose metabolism (84). If this process leads to an insufficient nutrient supply, the cartilage will be damaged. Approximately 95% of adenosine triphosphate in chondrocytes is produced by glucose metabolism. We hypothesized that prolonged fasting from carbohydrate products may restrict chondrocyte glucose intake, leading to inhibited mitochondrial respiration, overactive or impaired glycolysis, and reduced total adenosine triphosphate production, assuming other factors remain constant (85–87). Therefore, it leads to a glucose metabolism disorder in chondrocytes and destroys the stability of the structure and function of cartilage tissue, leading to joint degeneration (88, 89). Additionally, current experiments have demonstrated that a high-sugar diet can increase the risk of KOA (90). A high-sugar diet can increase the release of free radicals, accelerate the process of degeneration, and increase the production of pro-inflammatory factors, leading to the formation of a local pro-inflammatory environment and increased risk or aggravation of KOA (91). Therefore, sugar should be moderately consumed in daily life.

### 4.6 Height and KOA

We observed a possible association between height and genetically predicted KOA. A Finnish population-based cohort study that adjusted for confounding factors yielded consistent results (92). Current research has focused on obesity and KOA. Particularly, most studies have focused on the associations among BMI, weight, and KOA. Interestingly, few studies have focused on the causal relationship between height and KOA. Hart et al. incidentally found a positive correlation between height and KOA in knee joint radiological observations in middle-aged British women (93). Presently, the basic index for determining obesity is BMI; however, most researchers consider weight, waist circumference, and fat (94) and ignore the effect of height, especially in patients with KOA having a normal BMI. From a mechanical perspective, leg length is directly proportional to the pressure generated by the knee. In addition, some studies have found that height and upper body weight are closely related to knee cartilage compression (92). Concurrently, some studies have shown that height is closely related to bone morphology and cartilage thickness, which may be a potential factor influencing the relationship between the two (95).

### 4.7 Hot drink temperature, balneotherapy, and KOA

In daily life, drinking hot drinks helps attenuate the risk of KOA, as compared to cold drinks. Nonetheless, very little research has been conducted on this topic, with a greater focus on balneotherapy and KOA. We found a genetic link between hot drink temperature and KOA, whereas previous studies reported that balneotherapy effectively relieved the symptoms of KOA (96, 97). As a non-drug complementary therapy for KOA, the mechanism of balneotherapy remains unclear; however, balneotherapy has been verified to affect the progression of KOA through thermal, chemical, and mechanical pathways, among which thermal stimulation plays a more important role (98). Matsumoto et al. found that balneotherapy can significantly improve pain, stiffness, and functional limitations of patients and greatly improve their quality of life (99). Moreover, soaking the knee joint in warm water causes a neuroendocrine response through overheating stimulation, which increases the concentration of serum opioid peptides (such as enkephin) to achieve analgesic and sedative effects (100, 101). Heat stimulation also relieves muscle spasms, inflammation, and oxidative stress. A hot mud bath may reduce the release of pro-inflammatory factors IL-1β, TNF-α, and IL-8, increase the level of anti-inflammatory transforming growth factor-β, and increase the level of cortisol to reduce the inflammatory response. Promoting the reduction in serum extracellular heat shock protein 72 kDa levels can also reduce the release of pro-inflammatory factors (102, 103). Cells and microRNAs (miRNAs) can regulate each other. miRNAs play an important role in the pathogenesis of osteoarthritis and have been detected in human synovial fluid. Therefore, miRNAs can be used as both diagnostic markers and therapeutic targets (104, 105). miRNA-181a has a positive correlation with KOA owing to its involvement in cartilage degradation (106), and the upregulation of miRNA-155, miRNA-223, miRNA-181a, and miRNA-146a levels all play a role in the pathogenesis of cartilage damage and synovitis (107). A previous trial found that after mud bath treatment, miRNA-155, miRNA-223, miRNA-181a, and miRNA146a levels in patients with KOA were significantly decreased, suggesting that balneotherapy has a unique therapeutic effect on KOA (108). In our MR study, hydrotherapy was the main focus of balneotherapy, and the multivariate MR results suggest a potential mediating effect. Through literature review, we found that balneotherapy also includes mud therapy; therefore, confounding factors may affect the MR results. Consequently, we included mud therapy in this study. Bath therapy has the advantages of safety and convenience, and as an alternative therapy for KOA, it has a certain effect, which can appropriately reduce the use of non-steroidal anti-inflammatory drugs, as well as reduce pain and certain economic burden for patients with KOA (109), with good economic and social benefits (110).

### 4.8 Advantages and limitations

This study has several advantages. First, the MR method used was novel and could avoid bias caused by traditional epidemiological observational studies. In this study, we used univariate and multivariate MR analyses to evaluate the causal relationship between risk factors and KOA from genetics and conducted multiple sensitivity analyses to eliminate outliers. A comprehensive assessment of causality makes the results more reliable. Second, we used publicly available large GWAS data to ensure sufficient sample size and reliable results. We also used independent data for validation to avoid an overlap between the exposure and outcome samples (111). Third, the data were obtained from a European population to avoid bias caused by different populations. We used stringent standard screening tool variables to ensure that extreme values did not affect the results.

However, this study had some limitations. First, there was significant heterogeneity between some risk factors and KOA. Although the evaluation using the random-effects model showed that the results were robust, it inevitably posed some challenges to causality. Second, our use of rigorous criteria for the selection of IV might have missed some outcomes. Third, because of the excessive number of risk factors studied, the results from the multivariate MR analysis were inaccurate owing to bias caused by the existence of collinear problems; therefore, our application of the mv-lasso function to solve collinear problems corrected some outliers. Fourth, this study was based on a European population, which limits the generalizability of the results because of genetic differences among ethnic groups. Finally, the results of the MR analysis suggested a causal association; however, further clinical trials are needed to investigate the underlying mechanisms.

### 4.9 Clinical implications

This study provides an in-depth causal exploration of several potential risk factors for KOA. We found that BMI plays an irreplaceable role as a mediator between risk factors and KOA, which is in line with previous MR and meta-analysis studies on the causal relationship (112–114) between BMI and KOA that found that BMI increases the risk of KOA in line with our-study results. In clinical settings, physicians can emphasize the importance of weight management in KOA prevention and can encourage healthy eating (advising them to avoid prolonged abstinence from wheat products) and regular exercise to reduce obesity and related health problems. By identifying factors that have a clear causal relationship with KOA (hyperthyroidism/thyrotoxicosis, essential hypertension, MS), physicians can be more precise in assessing a patient’s risk, and concurrently treating and managing his or her thyroid disease, hypertension, and MS may help to reduce the risk of KOA. Public health policymakers can build on these findings to develop more targeted prevention and intervention measures, and by improving public health education, the associations between educational attainment and income and KOA risk found in the study remind public health policymakers of the need to consider the impact of socioeconomic factors on health. Policies can target low-income and less-educated populations with more health support and resources. The findings could also be used to develop health education policies for the general public, particularly in the areas of diet and lifestyle, and consideration could be given to incorporating screening and management of thyroid disorders and hypertension into public health programs, as well as raising public awareness of KOA risk factors.

## 5 Conclusion

In summary, our study systematically analyzed the causal relationships between genetically predicted KOA and thyroid dysfunction, MS, essential hypertension, educational level, income, dietary factors, height, balneotherapy, and psychophysiological factors. This study highlights the impact of modifiable risk factors on KOA and suggests that the use of drugs that interfere with risk factors can also affect the progression of KOA. Therefore, this study provides a new research direction for the prevention and treatment of KOA and information for the formulation of public health policies.

## Data availability statement

The original contributions presented in the study are included in the article/Supplementary material, further inquiries can be directed to the corresponding author/s.

## Ethics statement

This study was based on publicly available datasets. Ethical review and approval was not required for the study, in accordance with the local legislation and institutional requirements.

## Author contributions

ZD: Conceptualization, Methodology, Software, Visualization, Writing – original draft, Writing – review, editing. DG: Formal analysis, Methodology, Resources, Validation, Visualization, Writing – review, editing. JZ: Data curation, Formal analysis, Writing – review, editing. RZ: Validation, Writing – review, editing. CL: Resources, Validation, Writing – review, editing. HC: Data curation, Methodology, Writing.YM: Conceptualization, Funding acquisition, Methodology, Supervision, Writing–review and editing.

## Footnotes

^1^https://gwas.mrcieu.ac.uk/

^2^https://r8.finngen.fi/
