# Supplementary material for: Causal relationship between modifiable risk factors and knee osteoarthritis: a Mendelian randomization study
Source: Front Med (Lausanne). 2024 Sep 2;11:1405188. doi: 10.3389/fmed.2024.1405188 (PMC11402680; doi:10.3389/fmed.2024.1405188)

**Legends of Supplementary figures**

**Supplementary Figure 1.**Scatter plot of causal relationship between KOA and risk factors by Mendelian randomization.

**Supplementary Figure 2.**Forest plot of causal relationship between KOA and risk factors by Mendelian randomization.

**Supplementary Figure 3.**Funnel plots of Mendelian randomization of KOA and risk factors.

**Supplementary Figure 4.**Leave-one-out analysis of MR Causal effect between KOA and risk factors.

**Supplementary Figure 1.**Scatter plot of the causal relationship between KOA and its risk factors by Mendelian randomisation. Each of these points represents an IV, whereas the horizontal line at each point indicates a 95% confidence interval. The five coloured lines represent the Mendelian randomisation fitting results of the five analytical methods. IV, instrumental variable.


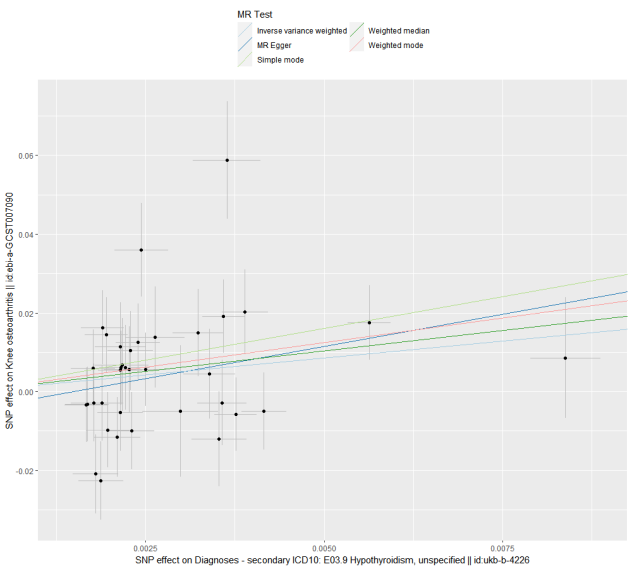

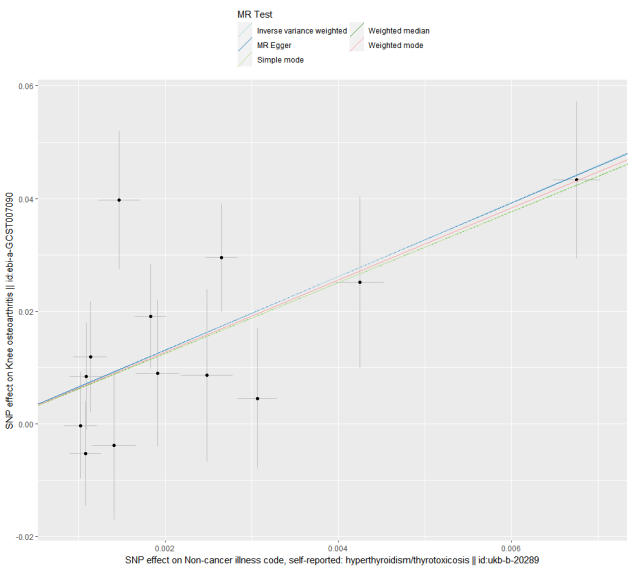

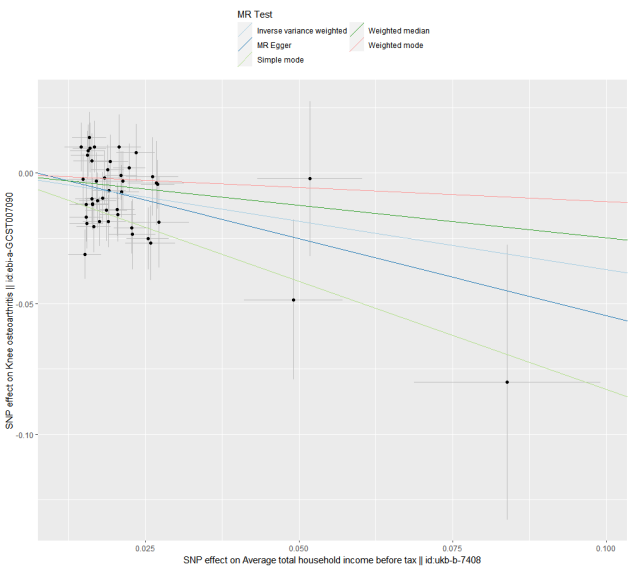

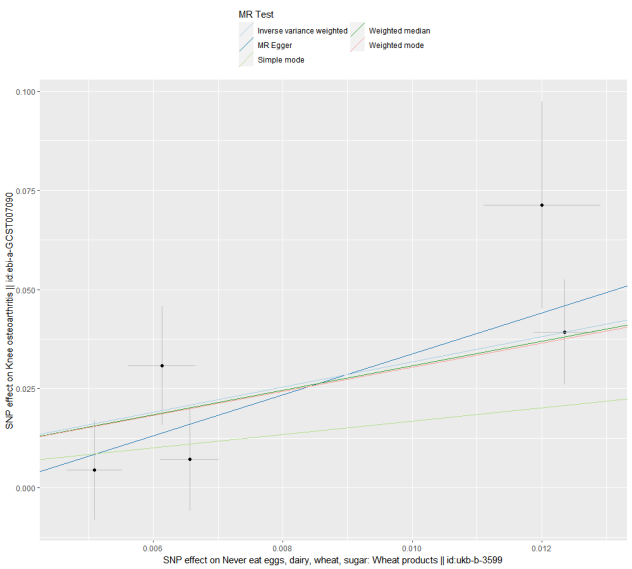

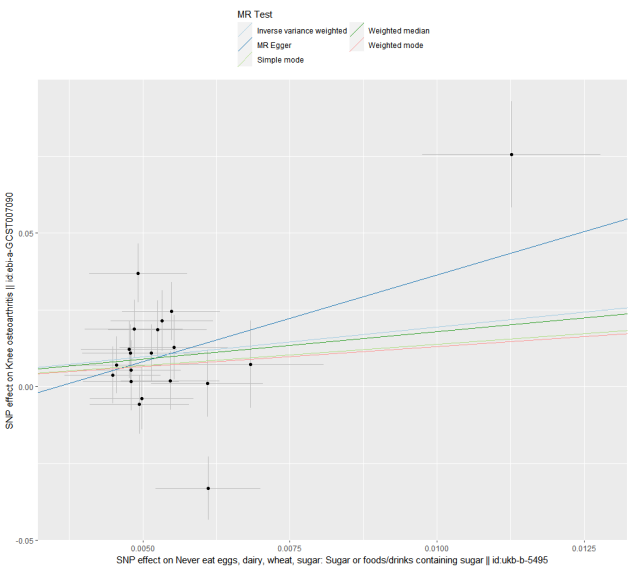

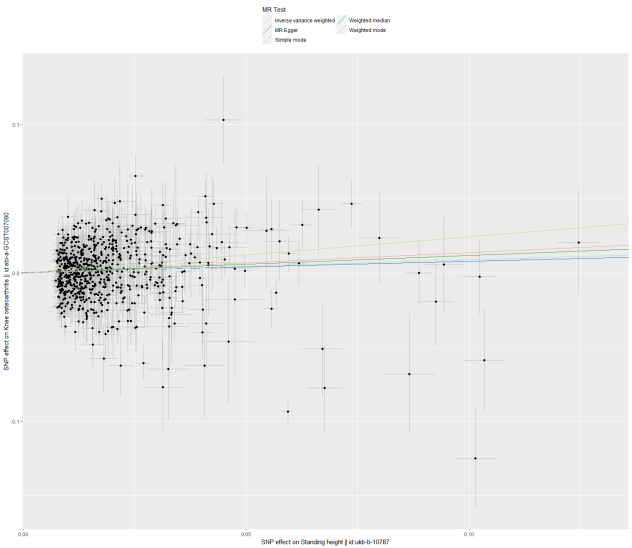

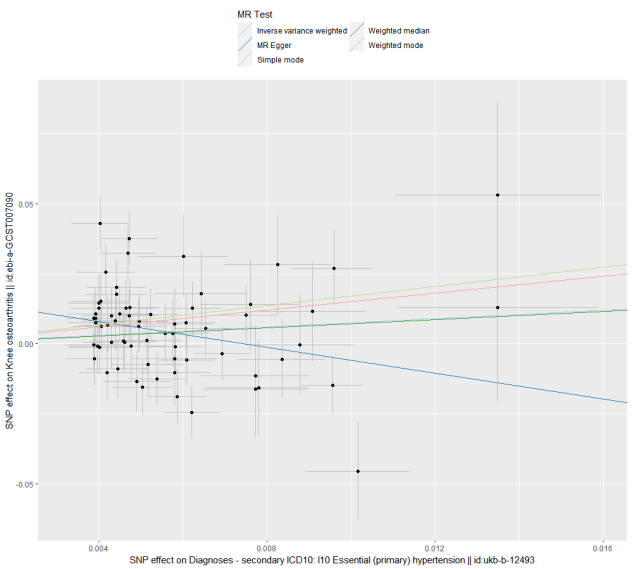

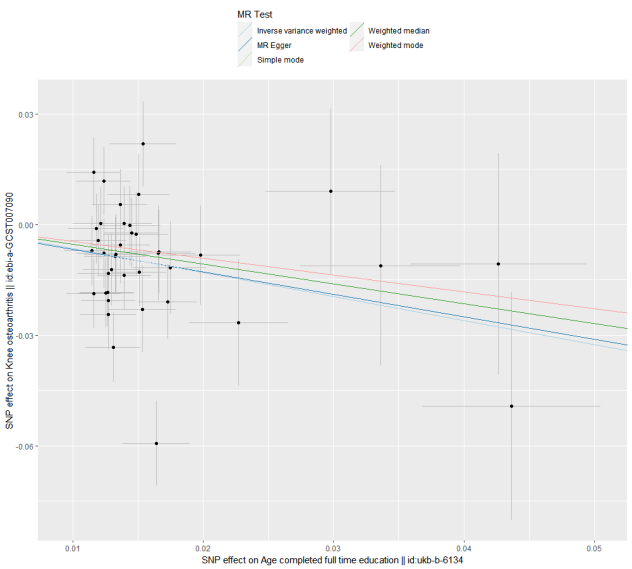

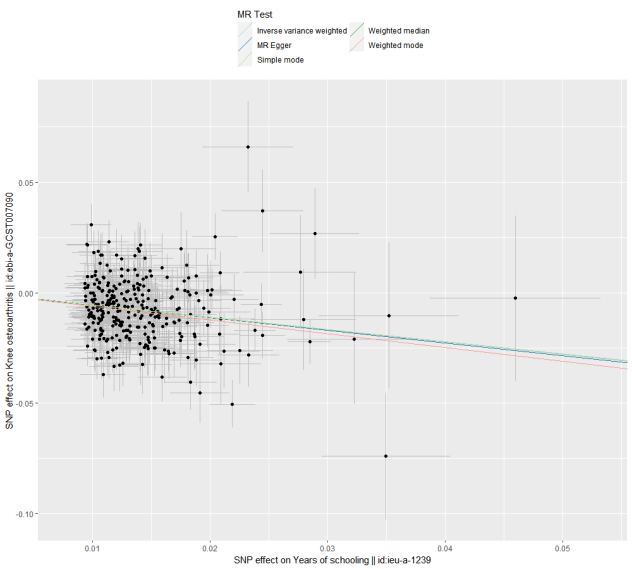

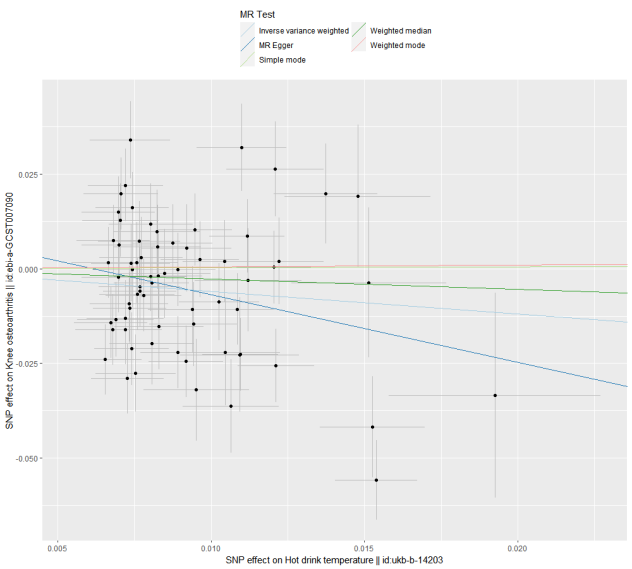

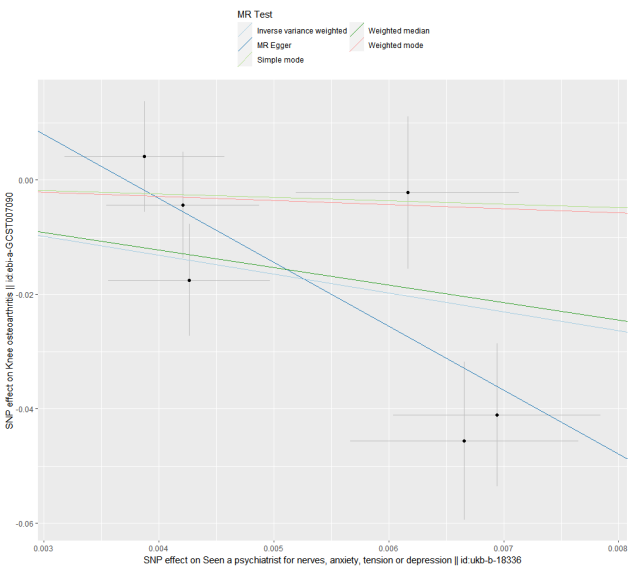

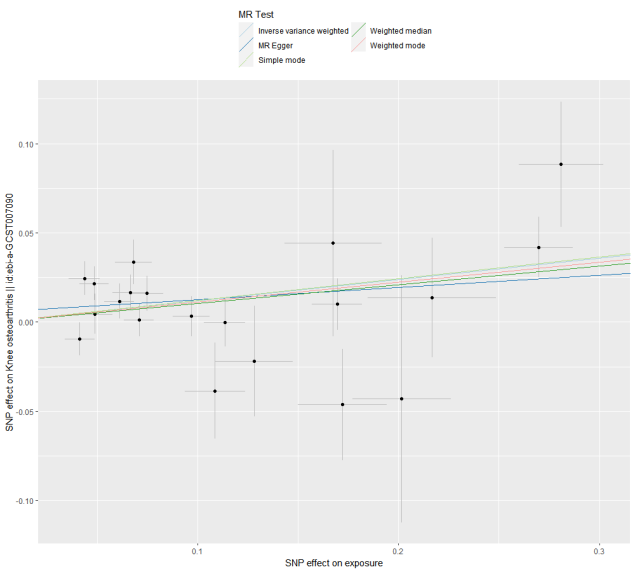


**Supplementary Figure 2.** Forest plot of causal relationship between KOA and risk factors by Mendelian randomization.


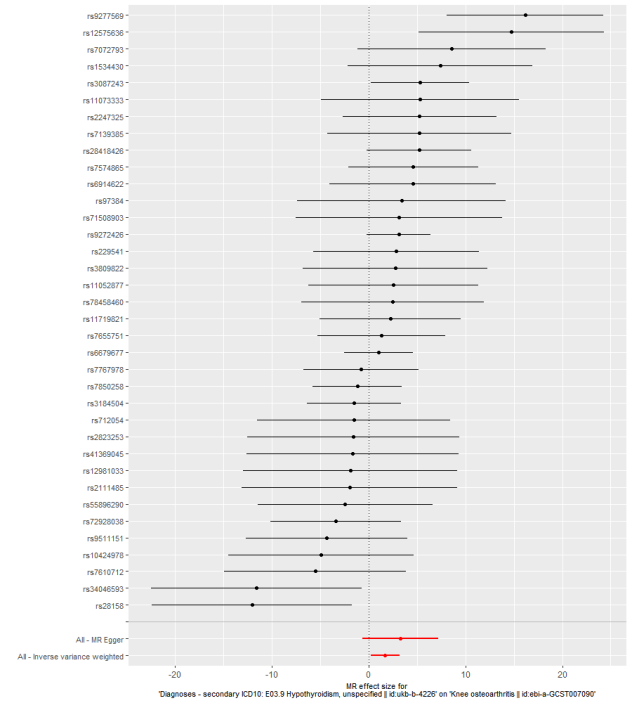

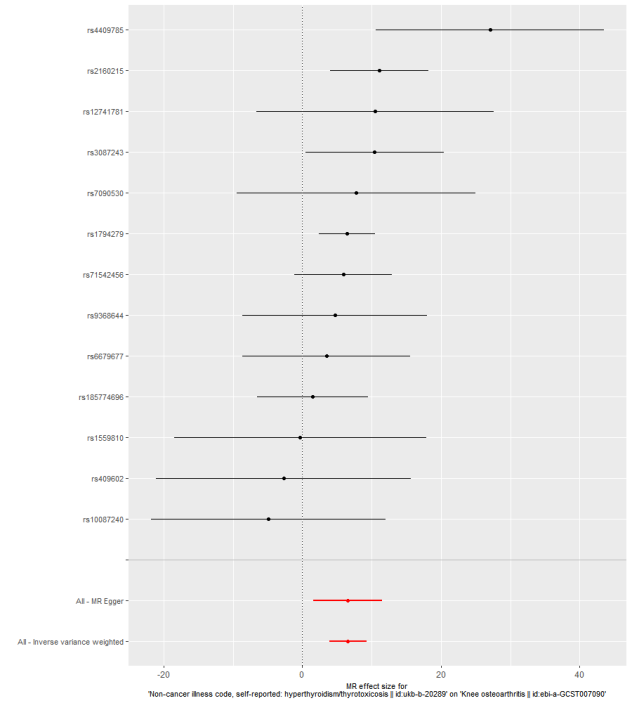

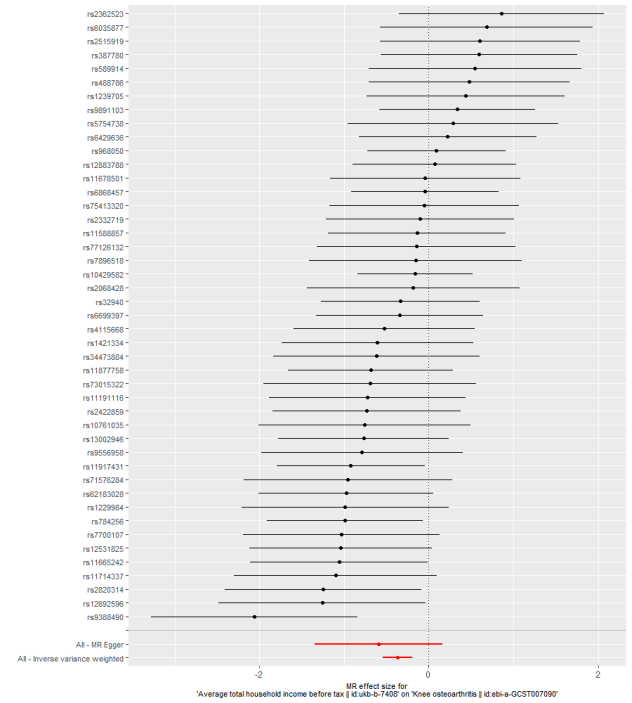

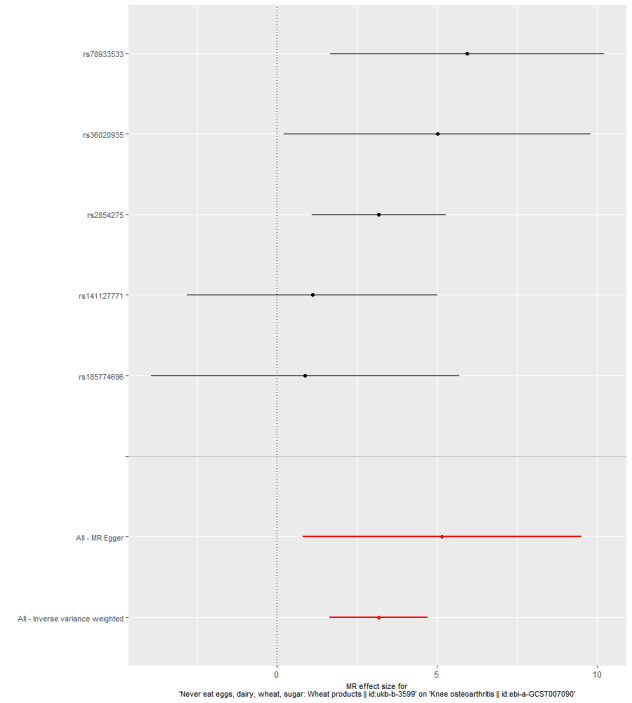

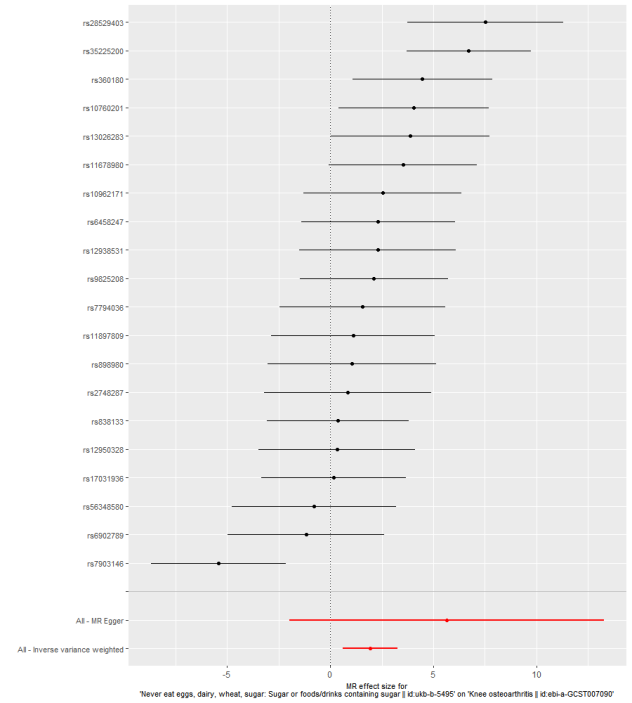

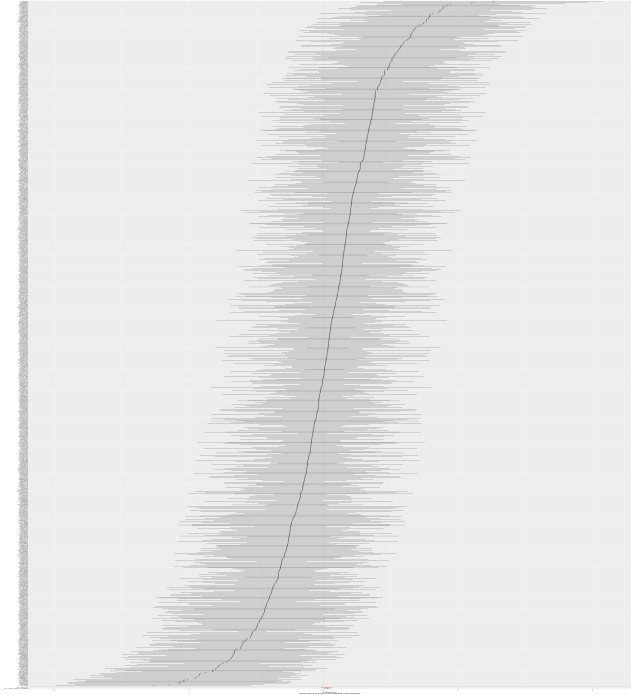

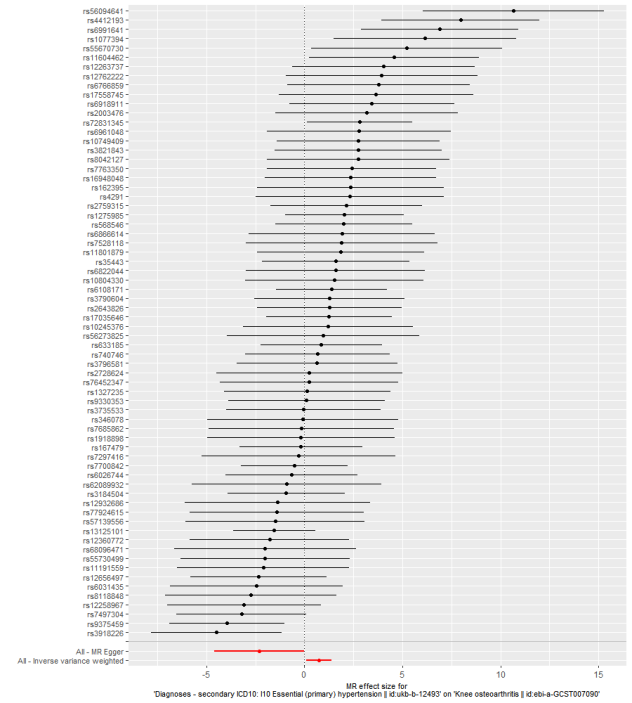

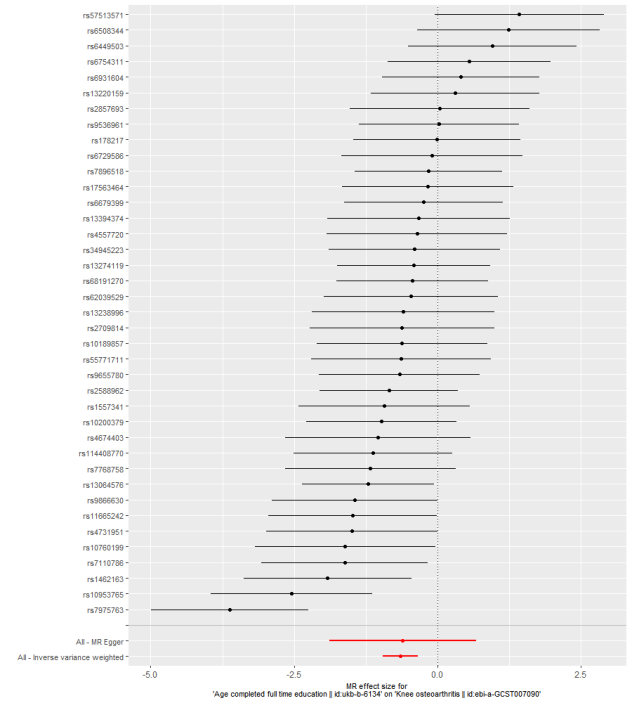

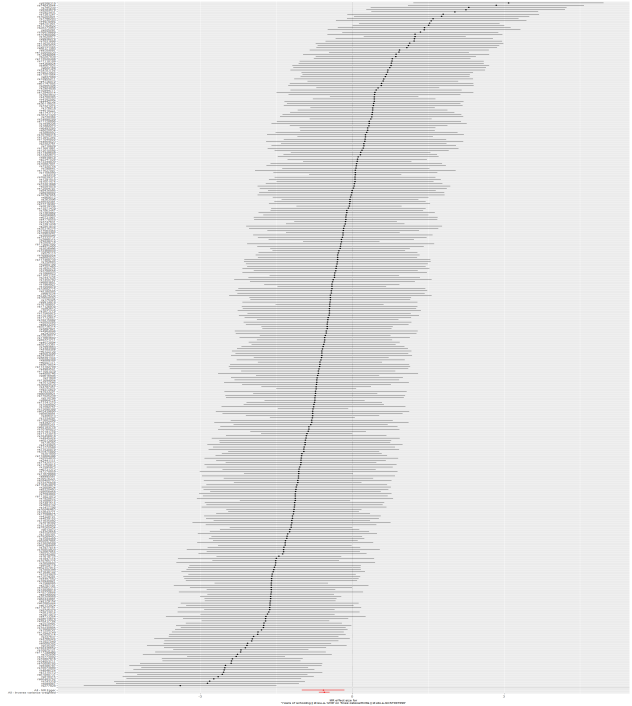

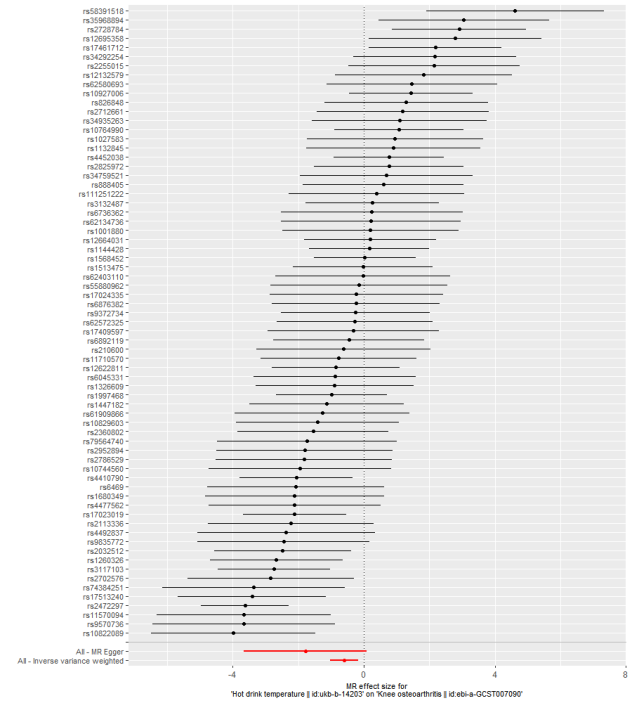

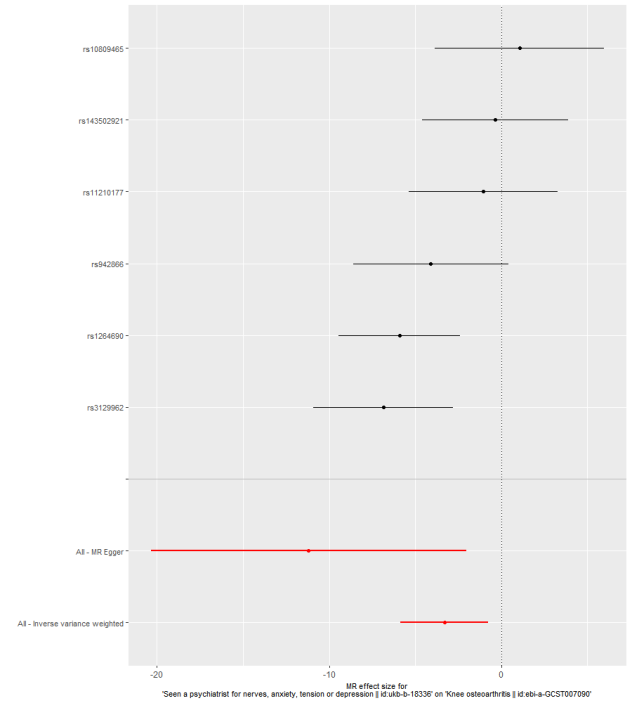

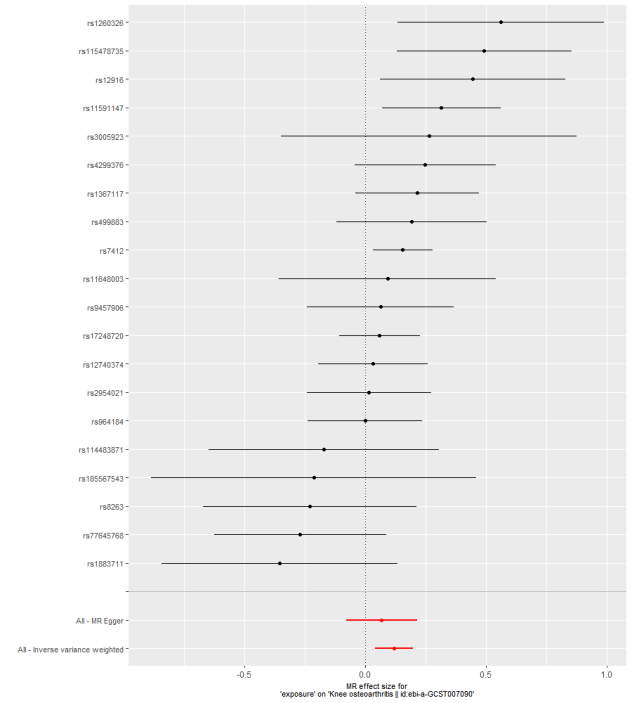


**Supplementary Figure 3.** Funnel plots of Mendelian randomization of KOA and risk factors.


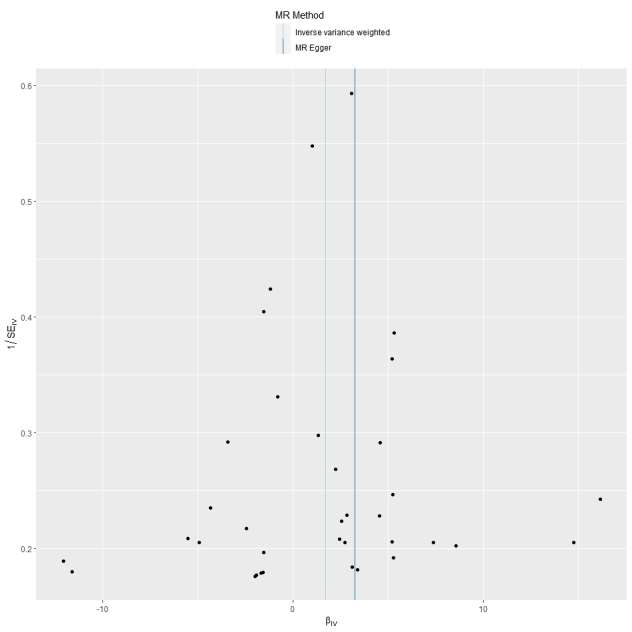

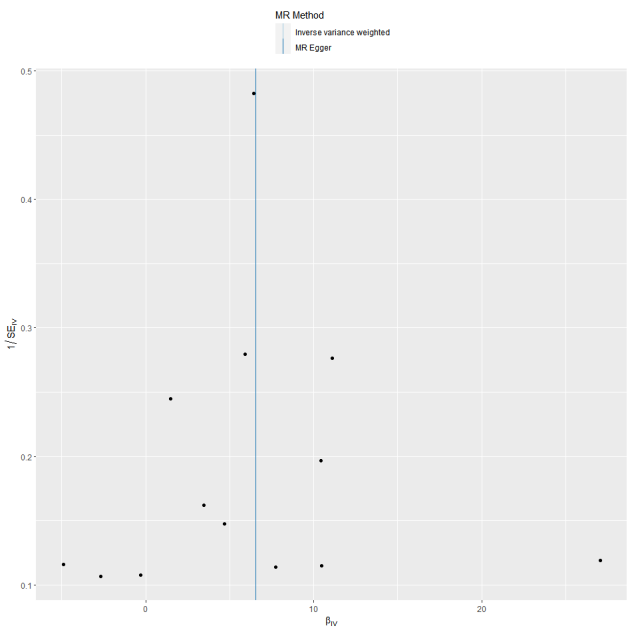

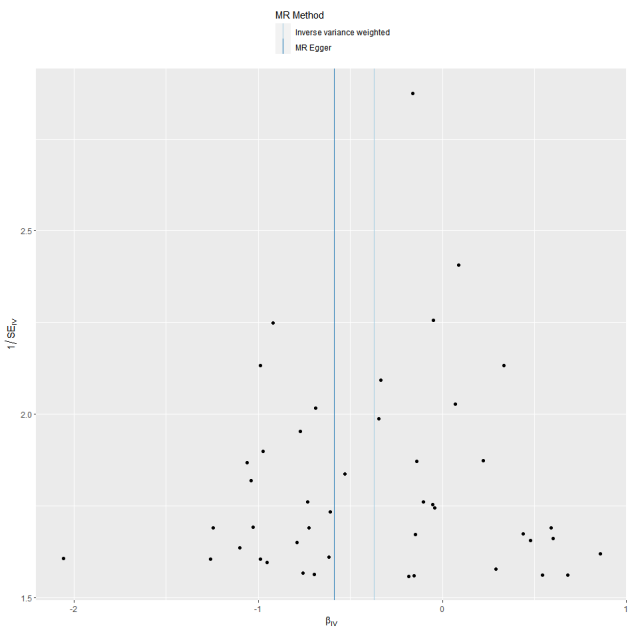

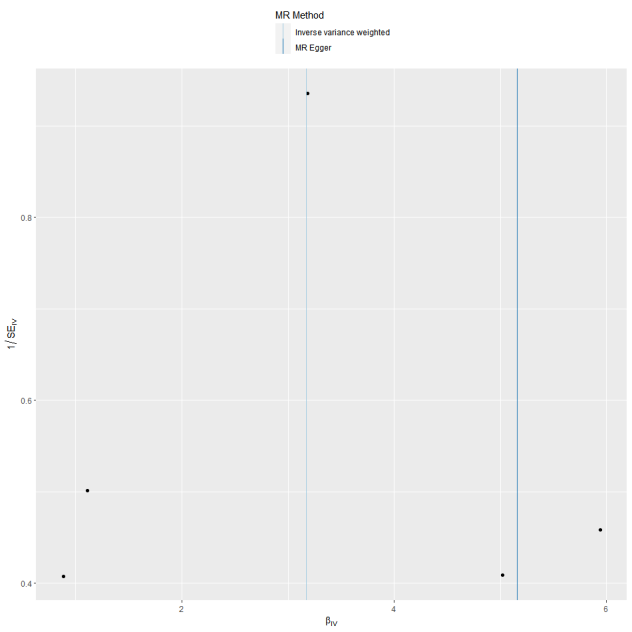

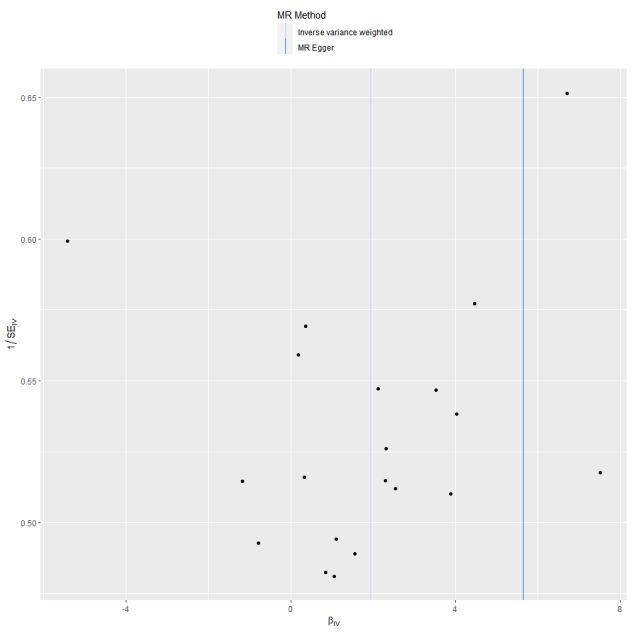

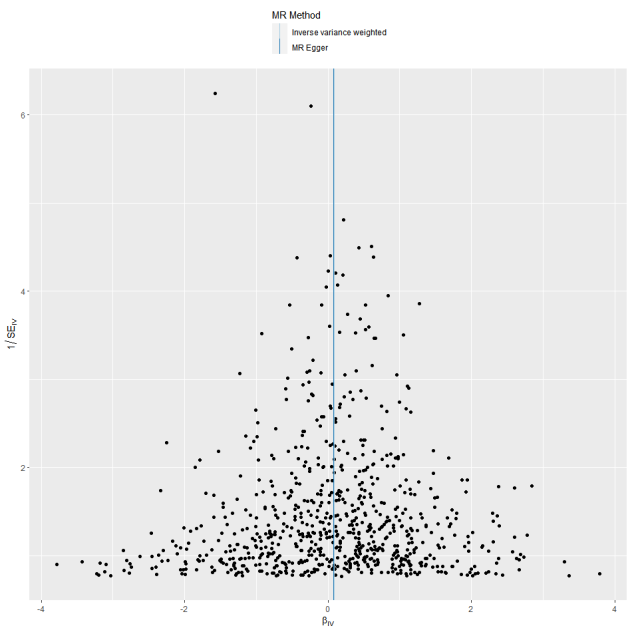

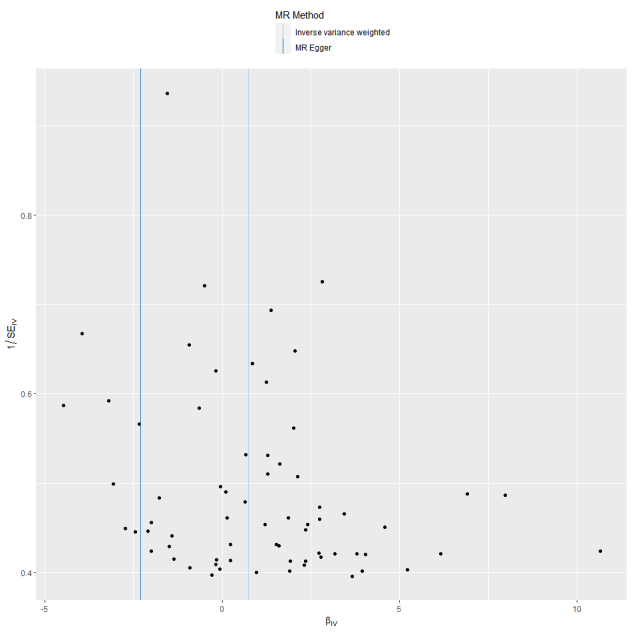

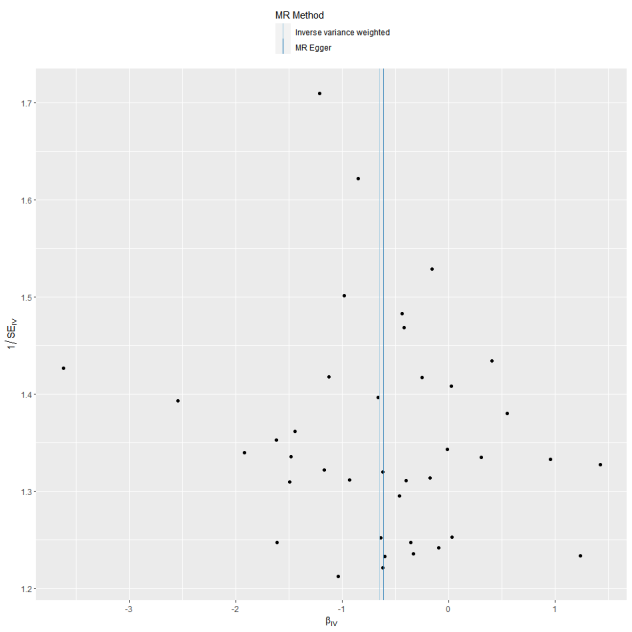

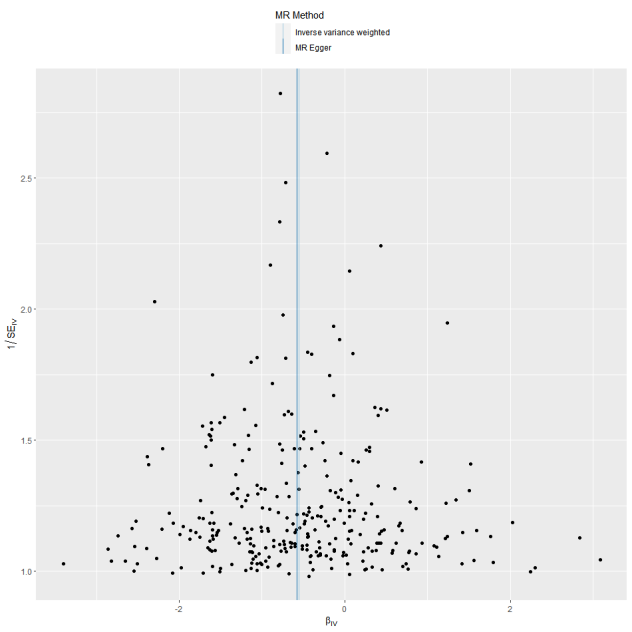

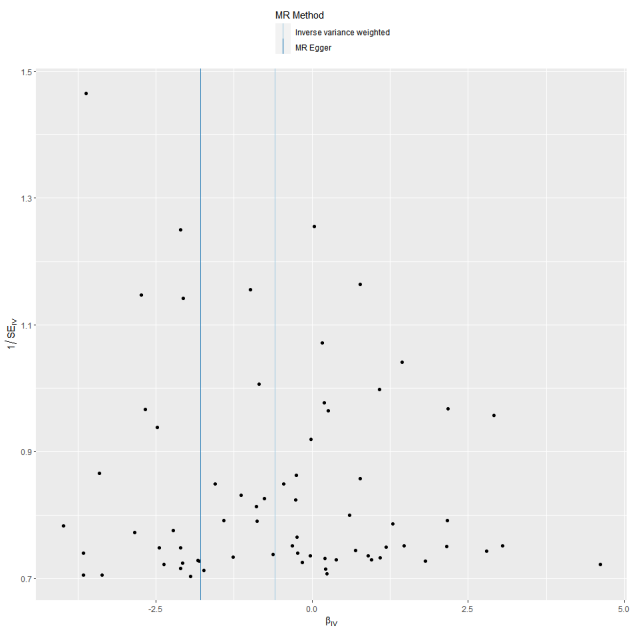

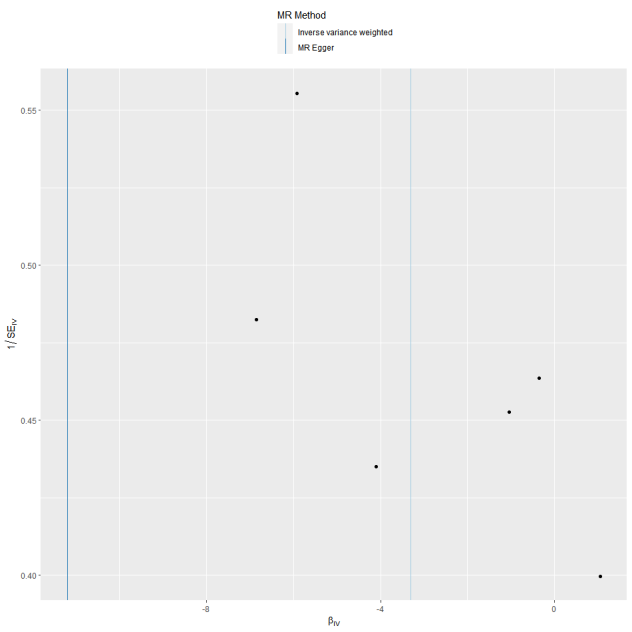

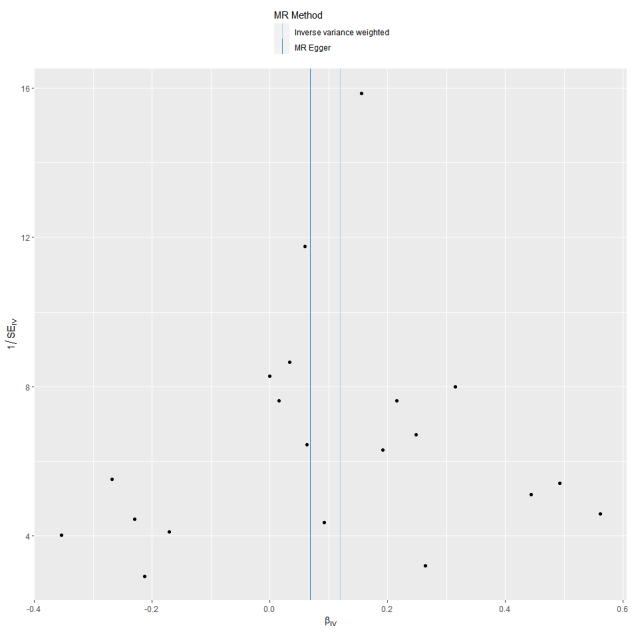


**Supplementary Figure 4.** Leave-one-out analysis of MR Causal effect between KOA and risk factors.


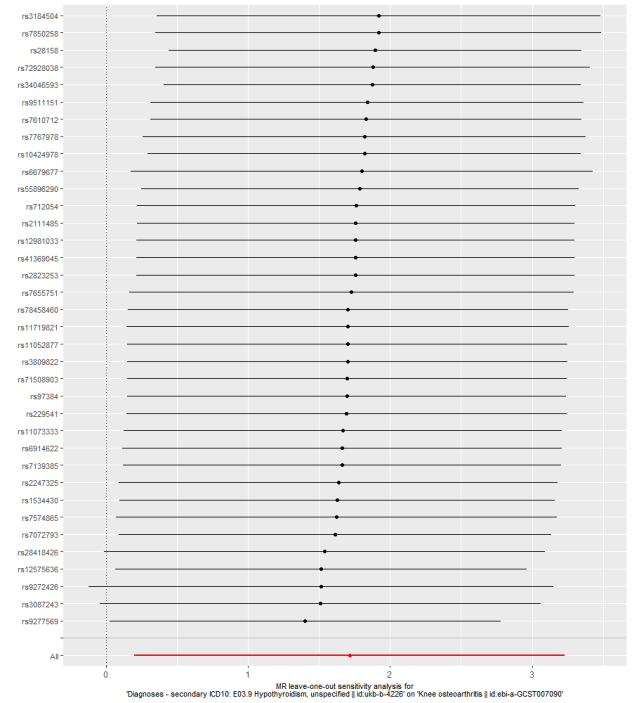

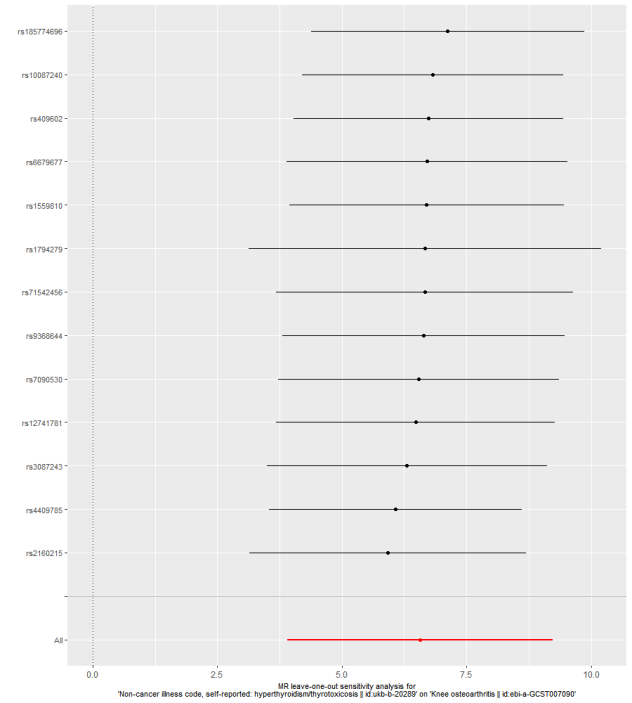

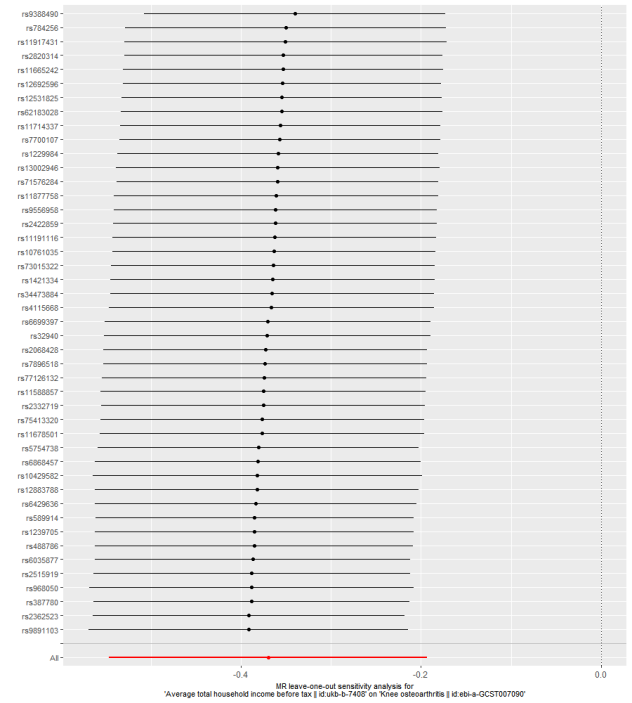

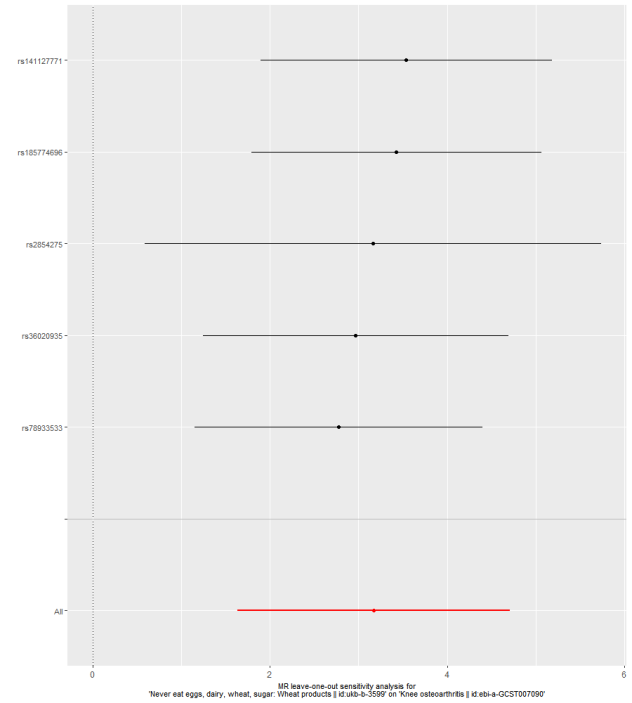

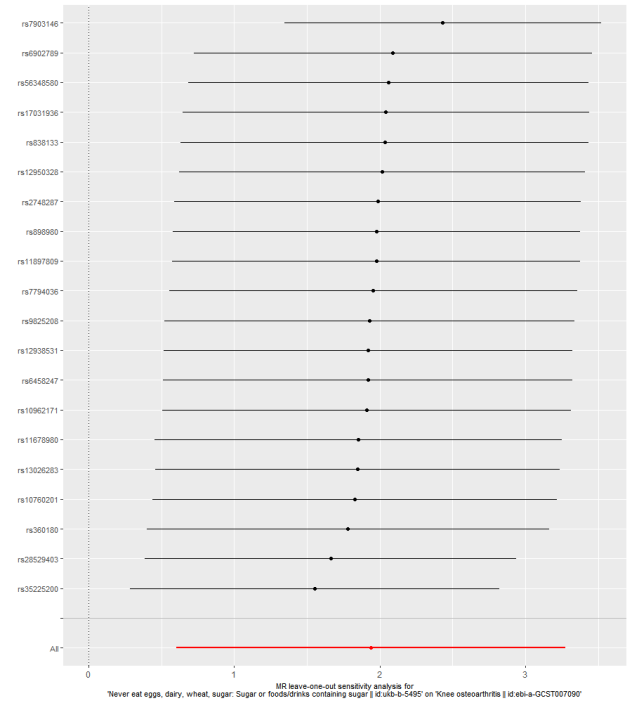

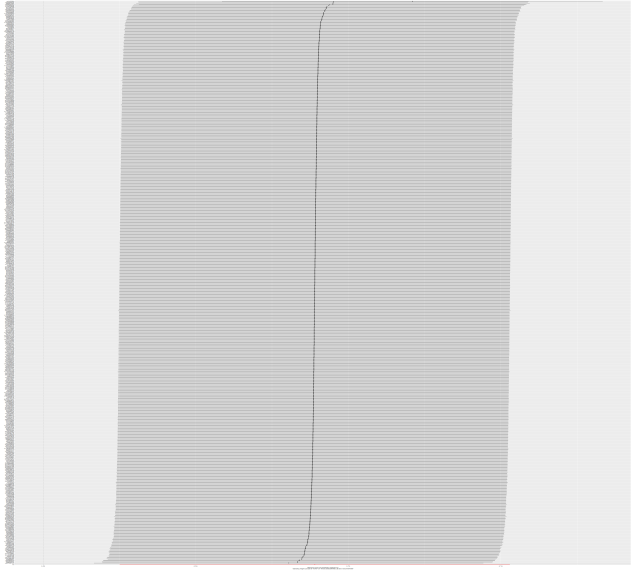

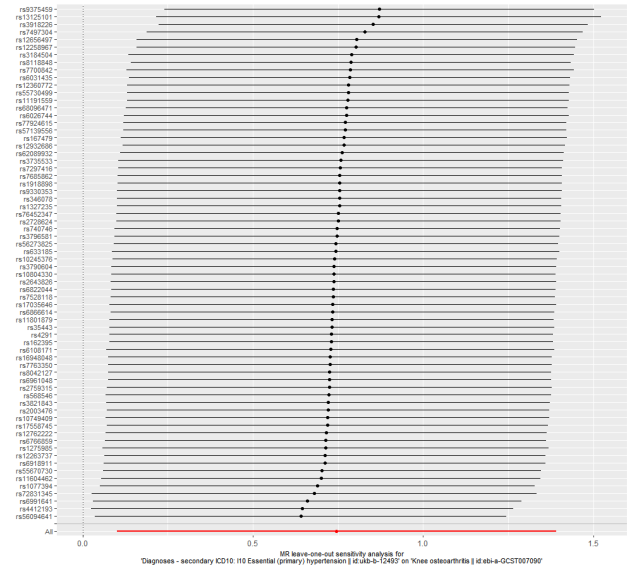

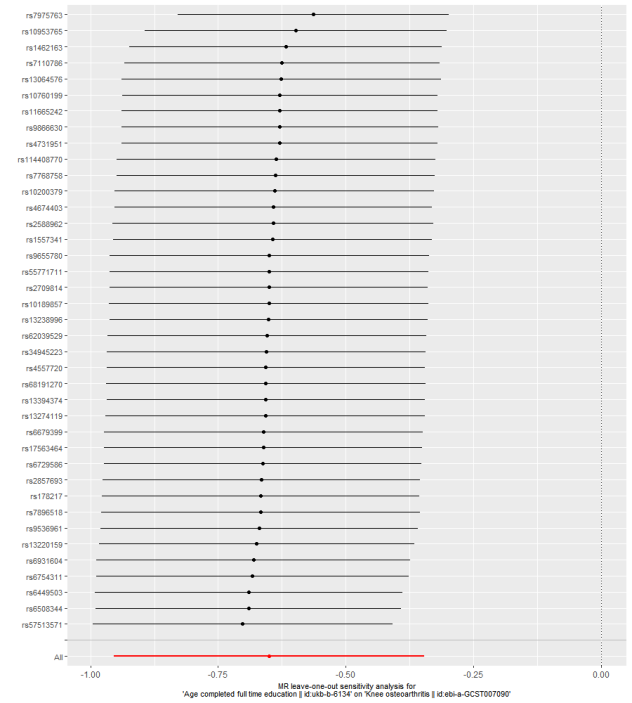

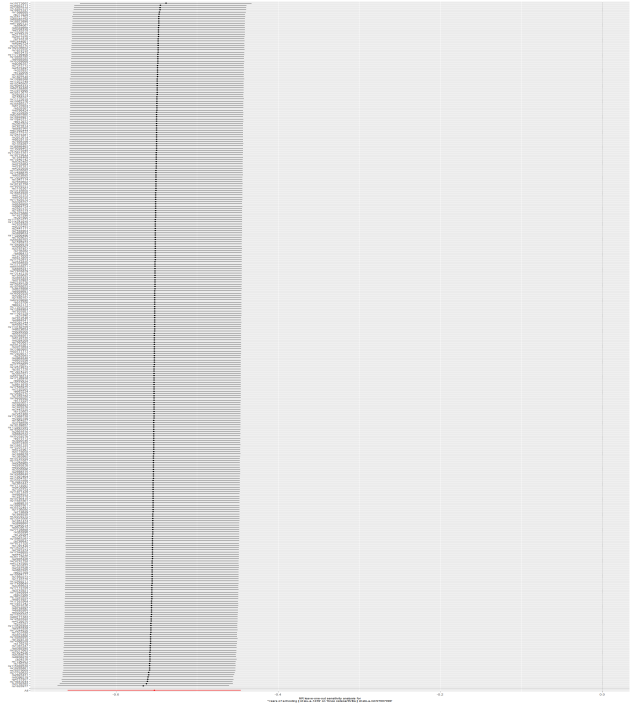

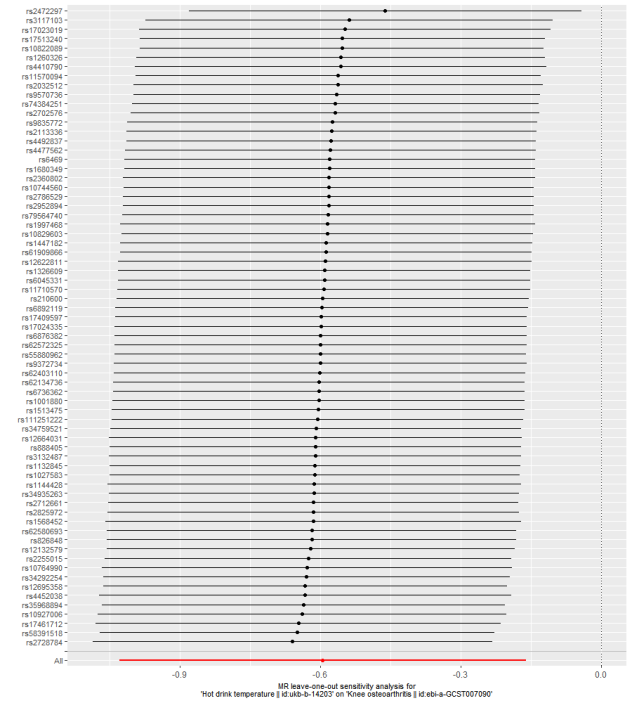

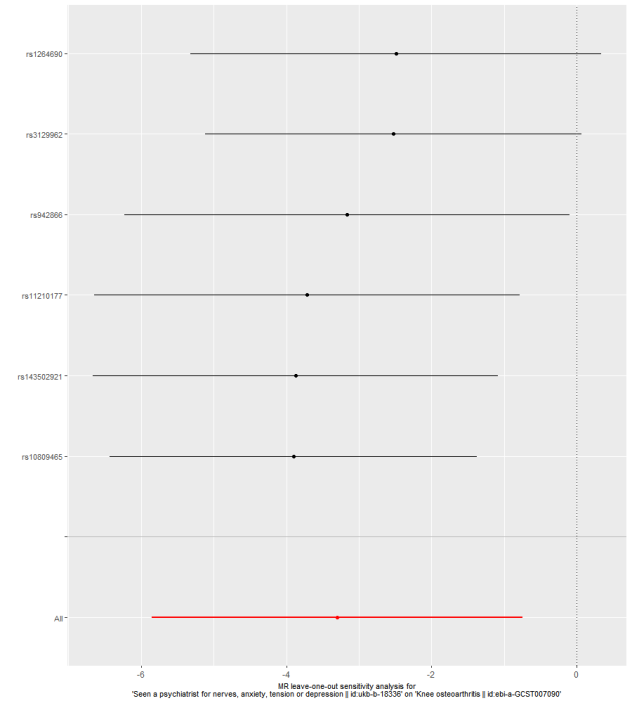

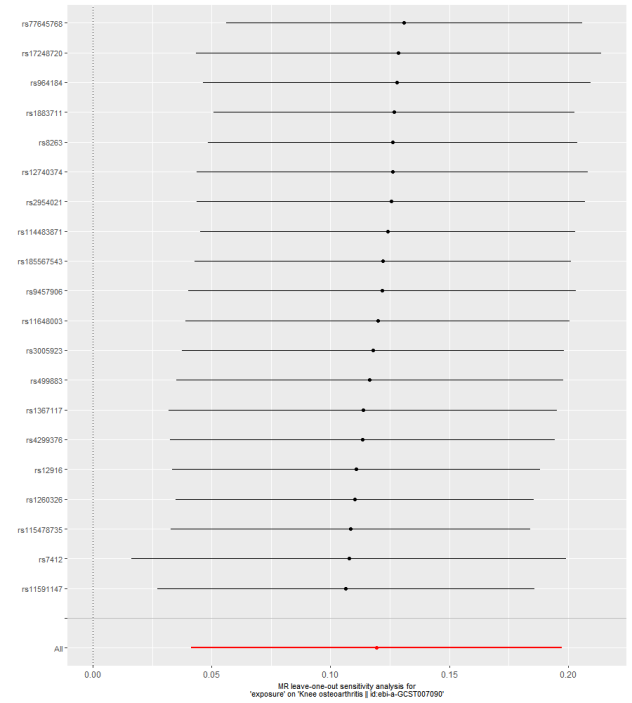

Supplement: Supplementary file 1 [file Data_Sheet_1.docx]
