# Supplementary material for: Causal relationship between modifiable risk factors and knee osteoarthritis: a Mendelian randomization study
Source: Front Med (Lausanne). 2024 Sep 2;11:1405188. doi: 10.3389/fmed.2024.1405188 (PMC11402680; doi:10.3389/fmed.2024.1405188)
Supplement: Supplementary file 3 [file Table_2.docx]

**Supplementary Table 2. Overview of the source of risk factors data.**

| **GWAS ID** | **Year** | **Trait** | **Consortium** | **Sample size** | **Case** | **Control** | **Number of SNPs** | **Population** | **Access address** |
| --- | --- | --- | --- | --- | --- | --- | --- | --- | --- |
| ukb-b-4226 | 2018 | Hypothyroidism, unspecified | MRC-IEU | 463,010 | 9,674 | 453,336 | 9,851,867 | European | <https://gwas.mrcieu.ac.uk/datasets/ukb-b-4226/> |
| ukb-b-20289 | 2018 | Hyperthyroidism/thyrotoxicosis | MRC-IEU | 462,933 | 3,545 | 459,388 | 9,851,867 | European | <https://gwas.mrcieu.ac.uk/datasets/ukb-b-20289/> |
| ukb-b-7408 | 2018 | Average total household income before tax | MRC-IEU | 397,751 | NA | NA | 9,851,867 | European | <https://gwas.mrcieu.ac.uk/datasets/ukb-b-7408/> |
| ukb-b-3599 | 2018 | Never eat eggs, dairy, wheat, sugar: Wheat products | MRC-IEU | 461,046 | 12,408 | 448,638 | 9,851,867 | European | <https://gwas.mrcieu.ac.uk/datasets/ukb-b-3599/> |
| ukb-b-5495 | 2018 | Never eat eggs, dairy, wheat, sugar: Sugar or foods/drinks containing sugar | MRC-IEU | 461,046 | 86,648 | 374,398 | 9,851,867 | European | <https://gwas.mrcieu.ac.uk/datasets/ukb-b-5495/> |
| ukb-a-389 | 2017 | Standing height | Neale Lab | 336,474 | NA | NA | 10,894,596 | European | <https://gwas.mrcieu.ac.uk/datasets/ukb-a-389/> |
| ukb-b-10787 | 2018 | Standing height | MRC-IEU | 461,950 | NA | NA | 9,851,867 | European | <https://gwas.mrcieu.ac.uk/datasets/ukb-b-10787/> |
| ukb-b-12493 | 2018 | Essential (primary) hypertension | MRC-IEU | 463,010 | 54,358 | 408,652 | 9,851,867 | European | <https://gwas.mrcieu.ac.uk/datasets/ukb-b-12493/> |
| ukb-b-6134 | 2018 | Age completed full time education | MRC-IEU | 307,897 | NA | NA | 9,851,867 | European | <https://gwas.mrcieu.ac.uk/datasets/ukb-b-6134/> |
| ieu-a-1239 | 2018 | Years of schooling | SSGAC | 766,345 | NA | NA | 10,101,242 | European | <https://gwas.mrcieu.ac.uk/datasets/ieu-a-1239/> |
| ukb-b-14203 | 2018 | Hot drink temperature | MRC-IEU | 457,873 | NA | NA | 9,851,867 | European | <https://gwas.mrcieu.ac.uk/datasets/ukb-b-14203/> |
| ukb-b-18336 | 2018 | Seen a psychiatrist for nerves, anxiety, tension or depression | MRC-IEU | 460,702 | 53,414 | 407,288 | 9,851,867 | European | <https://gwas.mrcieu.ac.uk/datasets/ukb-b-18336/> |
| finngen_R8_E4_METABOLIA | 2021 | Metabolic disorders | FINNGEN | 342,499 | 46,119 | 296,380 | 16380311 | European | https://storage.googleapis.com/finngen-public-data-r8/summary_stats/finngen_R8_E4_METABOLIA.gz |
