# Supplementary material for: Causal relationship between modifiable risk factors and knee osteoarthritis: a Mendelian randomization study
Source: Front Med (Lausanne). 2024 Sep 2;11:1405188. doi: 10.3389/fmed.2024.1405188 (PMC11402680; doi:10.3389/fmed.2024.1405188)
Supplement: Supplementary file 4 [file Table_3.docx]

**Supplementary Table 3.Summary information on SNPS associated with risk factors used in the analysis.**

| Genome-wide significant SNPs for Hypothyroidism | | | | | | | | | | | |  |  |
| --- | --- | --- | --- | --- | --- | --- | --- | --- | --- | --- | --- | --- | --- |
| SNP | Chr | Position | EA | OA | EAF | Hypothyroidism | | | Knee Osteoarthritis | | | R2 | F |
|  |  |  |  |  |  | beta | SE | pval | beta | SE | pval |  |  |
| rs10424978 | 19 | 4837557 | A | C | 0.600213 | -0.00197147 | 0.000305752 | 1.09999E-10 | 0.0097 | 0.0096 | 0.3116 | 8.97868E-05 | 41.57572742 |
| rs11052877 | 12 | 9905690 | G | A | 0.371778 | -0.00214561 | 0.000307289 | 2.90001E-12 | -0.0055 | 0.0096 | 0.5651 | 0.000105286 | 48.75348496 |
| rs11073333 | 15 | 38820647 | A | G | 0.781022 | -0.00214797 | 0.000357683 | 1.89998E-09 | -0.0114 | 0.0112 | 0.3102 | 7.78817E-05 | 36.06267583 |
| rs11719821 | 3 | 188088318 | C | T | 0.453831 | -0.00249797 | 0.000297221 | 4.30031E-17 | -0.0056 | 0.0093 | 0.5448 | 0.000152531 | 70.63396382 |
| rs12575636 | 11 | 95311260 | G | T | 0.189951 | 0.00244056 | 0.000378652 | 1.2E-10 | 0.036 | 0.0119 | 0.00239299 | 8.97158E-05 | 41.54287913 |
| rs12981033 | 19 | 50197406 | G | A | 0.393274 | -0.00168124 | 0.000303567 | 3.09999E-08 | 0.0032 | 0.0095 | 0.7345 | 6.62417E-05 | 30.67244772 |
| rs1534430 | 2 | 12644736 | T | C | 0.390306 | -0.00195149 | 0.000304131 | 1.40001E-10 | -0.0144 | 0.0095 | 0.1316 | 8.89165E-05 | 41.17270537 |
| rs2111485 | 2 | 163110536 | G | A | 0.606729 | 0.00167012 | 0.000302818 | 3.50002E-08 | -0.0033 | 0.0095 | 0.730801 | 6.56921E-05 | 30.41796138 |
| rs2247325 | 6 | 167369992 | G | A | 0.352495 | -0.00239418 | 0.000310151 | 1.20005E-14 | -0.0126 | 0.0097 | 0.1955 | 0.000128683 | 59.58889741 |
| rs229541 | 22 | 37591318 | A | G | 0.425573 | 0.00215015 | 0.000299726 | 7.29962E-13 | 0.0061 | 0.0094 | 0.5191 | 0.000111135 | 51.46201716 |
| rs28158 | 5 | 102595892 | G | C | 0.322381 | -0.00187415 | 0.000316808 | 3.29997E-09 | 0.0226 | 0.0099 | 0.02298 | 7.55776E-05 | 34.99568824 |
| rs2823253 | 21 | 16781136 | A | C | 0.331052 | -0.00177613 | 0.00031733 | 2.19999E-08 | 0.0028 | 0.0099 | 0.781601 | 6.76562E-05 | 31.32747271 |
| rs28418426 | 6 | 32619654 | C | T | 0.528825 | 0.00389207 | 0.00032544 | 5.79963E-33 | 0.0203 | 0.0107 | 0.0579495 | 0.000308813 | 143.026841 |
| rs3087243 | 2 | 204738919 | A | G | 0.450736 | -0.00358862 | 0.000297514 | 1.69981E-33 | -0.0191 | 0.0093 | 0.0401301 | 0.000314133 | 145.4917128 |
| rs3184504 | 12 | 111884608 | C | T | 0.517267 | -0.00376121 | 0.000295972 | 5.30029E-37 | 0.0057 | 0.0093 | 0.5387 | 0.000348668 | 161.4923796 |
| rs34046593 | 4 | 26111593 | A | G | 0.311638 | 0.001802 | 0.000320008 | 1.79999E-08 | -0.0209 | 0.01 | 0.0369403 | 6.84806E-05 | 31.7092541 |
| rs3809822 | 17 | 7234112 | G | C | 0.243432 | 0.00221653 | 0.000344787 | 1.29999E-10 | 0.0061 | 0.0108 | 0.5754 | 8.92517E-05 | 41.32792362 |
| rs41369045 | 13 | 50819594 | A | G | 0.084408 | 0.00298477 | 0.000532689 | 0.000000021 | -0.005 | 0.0167 | 0.765701 | 6.78039E-05 | 31.39586178 |
| rs55896290 | 2 | 1406507 | A | G | 0.332238 | 0.00214794 | 0.00031725 | 1.29987E-11 | -0.0052 | 0.0099 | 0.5989 | 9.89937E-05 | 45.83942423 |
| rs6679677 | 1 | 114303808 | A | C | 0.100759 | 0.00837666 | 0.000491138 | 3.19963E-65 | 0.0086 | 0.0153 | 0.575999 | 0.000627873 | 290.8927021 |
| rs6914622 | 6 | 148514301 | T | G | 0.322274 | 0.0022842 | 0.000318474 | 7.39946E-13 | 0.0104 | 0.01 | 0.2956 | 0.000111092 | 51.4420673 |
| rs7072793 | 10 | 6106266 | C | T | 0.411342 | 0.00189909 | 0.000301006 | 2.80001E-10 | 0.0163 | 0.0094 | 0.0847891 | 8.59633E-05 | 39.80511773 |
| rs712054 | 8 | 133919473 | A | G | 0.619442 | -0.00188727 | 0.000304944 | 6.1E-10 | 0.0029 | 0.0096 | 0.763001 | 8.27183E-05 | 38.30240212 |
| rs7139385 | 12 | 103897139 | T | C | 0.163109 | 0.00263591 | 0.000409511 | 1.2E-10 | 0.0138 | 0.0128 | 0.2821 | 8.94748E-05 | 41.43125946 |
| rs71508903 | 10 | 63779871 | T | C | 0.194137 | 0.00216752 | 0.000378058 | 9.80009E-09 | 0.0068 | 0.0118 | 0.5671 | 7.09885E-05 | 32.87058898 |
| rs72928038 | 6 | 90976768 | A | G | 0.17835 | 0.00352915 | 0.00038653 | 6.79986E-20 | -0.012 | 0.0121 | 0.3197 | 0.000180014 | 83.3627333 |
| rs7574865 | 2 | 191964633 | G | T | 0.77616 | -0.00323653 | 0.000355004 | 7.70016E-20 | -0.0149 | 0.0111 | 0.1794 | 0.000179483 | 83.11720007 |
| rs7610712 | 3 | 105918060 | A | G | 0.687971 | -0.00210498 | 0.000320783 | 5.30029E-11 | 0.0116 | 0.0101 | 0.2495 | 9.29913E-05 | 43.05973794 |
| rs7655751 | 4 | 149633421 | T | C | 0.211131 | -0.00339543 | 0.000362866 | 8.19974E-21 | -0.0045 | 0.0114 | 0.691 | 0.000189071 | 87.55785876 |
| rs7767978 | 6 | 31202486 | T | C | 0.243663 | -0.00357174 | 0.00034432 | 3.29989E-25 | 0.0028 | 0.0108 | 0.7941 | 0.000232351 | 107.6052101 |
| rs78458460 | 1 | 108347599 | T | G | 0.240785 | 0.00226489 | 0.000347362 | 7.00003E-11 | 0.0056 | 0.0109 | 0.6064 | 9.1812E-05 | 42.51358602 |
| rs7850258 | 9 | 100549013 | G | A | 0.668016 | 0.0041563 | 0.000314539 | 7.29962E-40 | -0.0049 | 0.0098 | 0.621901 | 0.000376973 | 174.6075003 |
| rs9272426 | 6 | 32605189 | G | A | 0.452211 | 0.00563363 | 0.000302216 | 1.50003E-77 | 0.0175 | 0.0095 | 0.0660906 | 0.000749938 | 347.4880377 |
| rs9277569 | 6 | 33058402 | T | C | 0.109461 | 0.0036389 | 0.000474194 | 1.69981E-14 | 0.0588 | 0.015 | 8.41492E-05 | 0.000127169 | 58.88792572 |
| rs9511151 | 13 | 24786576 | A | G | 0.345851 | -0.00230609 | 0.000312478 | 1.59993E-13 | 0.01 | 0.0098 | 0.3078 | 0.000117618 | 54.46427621 |
| rs97384 | 11 | 61624181 | C | T | 0.623482 | 0.00176266 | 0.000310305 | 1.29999E-08 | 0.006 | 0.0097 | 0.5361 | 6.96849E-05 | 32.26693212 |
| Genome-wide significant SNPs for Hyperthyroidism/thyrotoxicosis | | | | | | | | | | | | | |
| SNP | Chr | Position | EA | OA | EAF | Hyperthyroidism/thyrotoxicosis | | | Knee Osteoarthritis | | | R2 | F |
|  |  |  |  |  |  | beta | SE | pval | beta | SE | pval |  |  |
| rs10087240 | 8 | 129012574 | T | C | 0.457137 | 0.00107896 | 0.000182413 | 3.29997E-09 | -0.0053 | 0.4572 | 0.5699 | 7.55698E-05 | 34.98623758 |
| rs12741781 | 1 | 243428152 | G | T | 0.326869 | 0.00113606 | 0.000193411 | 4.30002E-09 | 0.0119 | 0.3261 | 0.2289 | 7.45229E-05 | 34.5015273 |
| rs1559810 | 3 | 188124354 | A | C | 0.407243 | 0.00102383 | 0.000184608 | 2.90001E-08 | -0.0003 | 0.4076 | 0.9788 | 6.64366E-05 | 30.75762454 |
| rs1794279 | 6 | 32667595 | T | G | 0.125825 | 0.00675577 | 0.000274324 | 6.5013E-134 | 0.0433 | 0.127 | 0.00200798 | 0.001308384 | 606.4852254 |
| rs185774696 | 6 | 32618190 | T | C | 0.296706 | 0.00306374 | 0.000230776 | 3.19963E-40 | 0.0045 | 0.2951 | 0.719201 | 0.000380574 | 176.246562 |
| rs2160215 | 14 | 81461472 | C | T | 0.375804 | 0.00265332 | 0.000187423 | 1.69981E-45 | 0.0295 | 0.3756 | 0.00209802 | 0.00043274 | 200.4156422 |
| rs3087243 | 2 | 204738919 | A | G | 0.450735 | -0.00183165 | 0.000181895 | 7.50067E-24 | -0.0191 | 0.45 | 0.0401301 | 0.000218993 | 101.4008468 |
| rs409602 | 5 | 156608284 | A | T | 0.144597 | 0.00140738 | 0.000257525 | 4.60002E-08 | -0.0038 | 0.1448 | 0.7752 | 6.45116E-05 | 29.86634532 |
| rs4409785 | 11 | 95311422 | C | T | 0.172636 | 0.00146705 | 0.000239633 | 9.20005E-10 | 0.0397 | 0.1721 | 0.001226 | 8.09549E-05 | 37.47957915 |
| rs6679677 | 1 | 114303808 | A | C | 0.100758 | 0.00248074 | 0.000300307 | 1.39991E-16 | 0.0086 | 0.1016 | 0.575999 | 0.000147384 | 68.23853767 |
| rs7090530 | 10 | 6110875 | A | C | 0.602142 | 0.00108379 | 0.00018483 | 4.49997E-09 | 0.0084 | 0.6035 | 0.3751 | 7.42668E-05 | 34.38297692 |
| rs71542456 | 6 | 32631807 | G | A | 0.204648 | 0.00424713 | 0.000282006 | 2.90001E-51 | 0.0251 | 0.2054 | 0.0985689 | 0.000489715 | 226.8153986 |
| rs9368644 | 6 | 30797083 | T | C | 0.151864 | -0.00191548 | 0.000251861 | 2.80027E-14 | -0.009 | 0.1499 | 0.4884 | 0.000124928 | 57.84043101 |
| Genome-wide significant SNPs for Average total household income before tax | | | | | | | | | | | | | |
| SNP | Chr | Position | EA | OA | EAF | Average total household income before tax | | | Knee Osteoarthritis | | | R2 | F |
|  |  |  |  |  |  | beta | SE | pval | beta | SE | pval |  |  |
| rs10429582 | 9 | 23346850 | C | T | 0.416388 | 0.0270261 | 0.00268405 | 7.59976E-24 | -0.0044 | 0.0094 | 0.641599 | 0.000254838 | 101.3872565 |
| rs10761035 | 9 | 99236092 | A | G | 0.188004 | 0.0186402 | 0.00338512 | 3.69999E-08 | -0.0141 | 0.0119 | 0.2346 | 7.6227E-05 | 30.32150925 |
| rs11165472 | 1 | 96200401 | T | A | 0.486343 | -0.0152441 | 0.0026424 | 0.000000008 | 0.0111 | 0.0093 | 0.2313 | 8.36679E-05 | 33.28160585 |
| rs11191116 | 10 | 103555611 | T | C | 0.351782 | -0.0163993 | 0.00277643 | 3.50002E-09 | 0.0119 | 0.0097 | 0.2224 | 8.77057E-05 | 34.88791221 |
| rs11588857 | 1 | 204587047 | A | G | 0.209469 | 0.0213402 | 0.00324615 | 4.90004E-11 | -0.003 | 0.0114 | 0.7899 | 0.000108643 | 43.21729879 |
| rs11665242 | 18 | 50907127 | G | A | 0.422902 | -0.0175478 | 0.00268166 | 6.00067E-11 | 0.0186 | 0.0094 | 0.0480397 | 0.000107642 | 42.81892304 |
| rs11678501 | 2 | 188919210 | C | T | 0.024727 | -0.0517926 | 0.00851645 | 1.2E-09 | 0.0022 | 0.0297 | 0.9415 | 9.29751E-05 | 36.98418397 |
| rs11714337 | 3 | 71582521 | A | G | 0.431007 | 0.0153807 | 0.00267881 | 9.40005E-09 | -0.0169 | 0.0094 | 0.0723902 | 8.28745E-05 | 32.96599557 |
| rs11877758 | 18 | 35138110 | G | T | 0.312055 | -0.0203602 | 0.00286697 | 1.20005E-12 | 0.014 | 0.0101 | 0.1638 | 0.00012678 | 50.43309243 |
| rs11917431 | 3 | 49644012 | T | C | 0.300831 | 0.0227135 | 0.00288021 | 3.10027E-15 | -0.0209 | 0.0101 | 0.0394103 | 0.000156329 | 62.18958103 |
| rs1229984 | 4 | 100239319 | C | T | 0.972949 | -0.0491072 | 0.00804788 | 0.000000001 | 0.0485 | 0.0306 | 0.1135 | 9.35999E-05 | 37.23275533 |
| rs1239705 | 13 | 51152422 | G | A | 0.538393 | 0.0155639 | 0.00265785 | 4.70002E-09 | 0.0068 | 0.0093 | 0.4645 | 8.6204E-05 | 34.29049428 |
| rs1239705 | 13 | 51152422 | G | A | 0.538393 | 0.0155639 | 0.00265785 | 4.70002E-09 | 0.137 | 0.0675 | 0.0423 | 8.6204E-05 | 34.29049428 |
| rs12531825 | 7 | 8005174 | A | G | 0.122596 | -0.0258177 | 0.00406172 | 2.1E-10 | 0.0268 | 0.0142 | 0.0590201 | 0.000101569 | 40.40293859 |
| rs12692596 | 2 | 161265910 | T | C | 0.3717 | -0.0154093 | 0.00273347 | 0.000000017 | 0.0194 | 0.0096 | 0.0436204 | 7.98898E-05 | 31.7786185 |
| rs12883788 | 14 | 33303540 | T | C | 0.459864 | -0.0188574 | 0.00266308 | 1.39991E-12 | -0.0013 | 0.0093 | 0.8866 | 0.000126046 | 50.14100297 |
| rs13002946 | 2 | 100801959 | A | T | 0.269216 | 0.0205047 | 0.00299285 | 7.29962E-12 | -0.0158 | 0.0105 | 0.1329 | 0.000117998 | 46.93909952 |
| rs1421334 | 8 | 30865733 | C | A | 0.549035 | 0.0162948 | 0.00267028 | 0.000000001 | -0.0099 | 0.0094 | 0.2884 | 9.36122E-05 | 37.23765115 |
| rs1455350 | 2 | 199497115 | A | T | 0.477454 | -0.0172341 | 0.00265961 | 9.20026E-11 | -0.0064 | 0.0093 | 0.4936 | 0.000105556 | 41.98934683 |
| rs2068428 | 9 | 1792147 | T | C | 0.239041 | 0.0169724 | 0.00309515 | 4.20001E-08 | -0.0031 | 0.0109 | 0.777301 | 7.55926E-05 | 30.06913474 |
| rs2332719 | 3 | 123712966 | G | A | 0.278917 | -0.0183042 | 0.00295323 | 5.69994E-10 | 0.0019 | 0.0104 | 0.8517 | 9.65726E-05 | 38.41534856 |
| rs2362523 | 19 | 5002301 | G | A | 0.339926 | 0.0158667 | 0.00279836 | 1.40001E-08 | 0.0136 | 0.0098 | 0.1643 | 8.08202E-05 | 32.14873348 |
| rs2422859 | 20 | 3132828 | G | T | 0.471922 | 0.0163711 | 0.00264718 | 6.19998E-10 | -0.012 | 0.0093 | 0.1958 | 9.6147E-05 | 38.24605039 |
| rs2515919 | 6 | 31564167 | G | A | 0.369978 | -0.0159385 | 0.00273539 | 5.69994E-09 | -0.0096 | 0.0096 | 0.3193 | 8.53509E-05 | 33.95112738 |
| rs2820314 | 1 | 201872209 | C | A | 0.338157 | -0.0165601 | 0.00278855 | 2.90001E-09 | 0.0206 | 0.0098 | 0.03555 | 8.86583E-05 | 35.26686491 |
| rs3130264 | 6 | 33301229 | G | C | 0.513869 | -0.015448 | 0.00264313 | 5.1E-09 | -0.012 | 0.0093 | 0.1943 | 8.58734E-05 | 34.15901112 |
| rs32940 | 5 | 141132286 | C | T | 0.700979 | 0.0211431 | 0.00289645 | 2.90001E-13 | -0.0071 | 0.0101 | 0.4846 | 0.000133948 | 53.28476714 |
| rs34473884 | 10 | 133761285 | A | G | 0.248977 | 0.017225 | 0.00305604 | 0.000000017 | -0.0106 | 0.0107 | 0.3241 | 7.98646E-05 | 31.76860967 |
| rs387780 | 2 | 32502495 | C | T | 0.674498 | 0.0167299 | 0.00282732 | 3.29997E-09 | 0.0099 | 0.0099 | 0.3168 | 8.80212E-05 | 35.01342341 |
| rs4115668 | 16 | 28607532 | A | G | 0.346945 | -0.0179965 | 0.00279169 | 1.09999E-10 | 0.0095 | 0.0098 | 0.3291 | 0.000104468 | 41.55655482 |
| rs488786 | 1 | 20888207 | T | C | 0.164058 | 0.0206878 | 0.00356762 | 6.69993E-09 | 0.0099 | 0.0125 | 0.4309 | 8.45324E-05 | 33.62553899 |
| rs5754738 | 22 | 34280249 | G | A | 0.709551 | -0.0162464 | 0.00292038 | 2.69998E-08 | -0.0047 | 0.0103 | 0.6483 | 7.78019E-05 | 30.94805561 |
| rs589914 | 11 | 57661032 | G | A | 0.684776 | 0.0156098 | 0.00285076 | 4.39997E-08 | 0.0085 | 0.01 | 0.393 | 7.53754E-05 | 29.98273048 |
| rs6035877 | 20 | 21512532 | C | A | 0.469244 | -0.0145138 | 0.00265042 | 4.30002E-08 | -0.0099 | 0.0093 | 0.2857 | 7.53857E-05 | 29.98683877 |
| rs62183028 | 2 | 212631483 | T | G | 0.310562 | -0.0189832 | 0.00286114 | 3.19963E-11 | 0.0185 | 0.01 | 0.0646696 | 0.000110663 | 44.0208521 |
| rs6429636 | 1 | 44183540 | T | G | 0.719691 | 0.0192858 | 0.00293518 | 5.00035E-11 | 0.0043 | 0.0103 | 0.675601 | 0.000108529 | 43.17214774 |
| rs6699397 | 1 | 91212216 | G | A | 0.369644 | -0.0190757 | 0.00274153 | 3.50026E-12 | 0.0066 | 0.0096 | 0.4946 | 0.000121706 | 48.41420206 |
| rs6868457 | 5 | 60550041 | C | T | 0.478238 | 0.0209797 | 0.0026601 | 3.10027E-15 | -0.001 | 0.0093 | 0.9165 | 0.000156359 | 62.20143975 |
| rs71576284 | 7 | 126244536 | A | C | 0.008961 | -0.0838859 | 0.0151769 | 3.29997E-08 | 0.08 | 0.0526 | 0.1282 | 7.6801E-05 | 30.54988697 |
| rs73015322 | 6 | 163857445 | T | G | 0.076734 | -0.0271853 | 0.00495368 | 4.09996E-08 | 0.0189 | 0.0174 | 0.2776 | 7.57126E-05 | 30.11689393 |
| rs75413320 | 19 | 13149854 | C | T | 0.108161 | -0.0261129 | 0.00426829 | 9.49992E-10 | 0.0014 | 0.0149 | 0.9276 | 9.40915E-05 | 37.42830755 |
| rs7700107 | 4 | 17880416 | C | A | 0.138299 | -0.0228296 | 0.00382992 | 2.5E-09 | 0.0235 | 0.0135 | 0.0819106 | 8.93238E-05 | 35.53162057 |
| rs77126132 | 7 | 54966738 | A | G | 0.093289 | 0.0267483 | 0.00457921 | 5.19996E-09 | -0.0039 | 0.016 | 0.8097 | 8.57754E-05 | 34.12000343 |
| rs784256 | 18 | 53398626 | A | G | 0.810934 | -0.0253813 | 0.00339684 | 7.89951E-14 | 0.0251 | 0.0119 | 0.0352298 | 0.000140348 | 55.83099389 |
| rs7896518 | 10 | 65104500 | G | A | 0.428029 | 0.014813 | 0.00270047 | 4.09996E-08 | -0.0023 | 0.0095 | 0.8085 | 7.5642E-05 | 30.08881942 |
| rs9388490 | 6 | 126704795 | T | C | 0.439901 | 0.015105 | 0.00266861 | 0.000000015 | -0.0311 | 0.0094 | 0.00090099 | 8.05425E-05 | 32.03827001 |
| rs9556958 | 13 | 99100046 | T | C | 0.524062 | -0.0153409 | 0.0026641 | 8.50002E-09 | 0.0121 | 0.0093 | 0.1947 | 8.33591E-05 | 33.15877281 |
| rs968050 | 6 | 98574560 | T | C | 0.483185 | 0.0223829 | 0.00265073 | 3.10027E-17 | 0.002 | 0.0093 | 0.8287 | 0.000179231 | 71.30164318 |
| rs9891103 | 17 | 44091886 | T | C | 0.229018 | -0.0234517 | 0.00314818 | 9.3994E-14 | -0.0078 | 0.011 | 0.4783 | 0.000139495 | 55.49161506 |
| Genome-wide significant SNPs for Never eat eggs, dairy, wheat, sugar: Wheat products | | | | | | | | | | | | | |
| SNP | Chr | Position | EA | OA | EAF | Never eat eggs, dairy, wheat, sugar: Wheat products | | | Knee Osteoarthritis | | | R2 | F |
|  |  |  |  |  |  | beta | SE | pval | beta | SE | pval |  |  |
| rs141127771 | 6 | 32632200 | A | G | 0.259608 | 0.00656279 | 0.000452886 | 1.39991E-47 | 0.0073 | 0.0131 | 0.578901 | 0.000455258 | 209.9893843 |
| rs185774696 | 6 | 32618190 | T | C | 0.296721 | 0.00509133 | 0.00042972 | 2.19989E-32 | 0.0045 | 0.0125 | 0.719201 | 0.000304379 | 140.3749053 |
| rs2854275 | 6 | 32628428 | A | C | 0.146173 | 0.0123508 | 0.000476448 | 3.6983E-148 | 0.0393 | 0.0132 | 0.00300601 | 0.0014554 | 671.9814396 |
| rs36020935 | 6 | 32519791 | T | G | 0.199675 | 0.00613647 | 0.000516636 | 1.50003E-32 | 0.0308 | 0.015 | 0.0403999 | 0.000305908 | 141.0801837 |
| rs78933533 | 6 | 32604198 | A | G | 0.06572 | 0.0120034 | 0.000904652 | 3.50026E-40 | 0.0713 | 0.0262 | 0.00657506 | 0.000381712 | 176.0530601 |
| Genome-wide significant SNPs for Never eat eggs, dairy, wheat, sugar: Sugar or foods/drinks containing sugar | | | | | | | | | | | | | |
| SNP | Chr | Position | EA | OA | EAF | Never eat eggs, dairy, wheat, sugar: ugar or foods/drinks containing sugar | | | Knee Osteoarthritis | | | R2 | F |
|  |  |  |  |  |  | beta | SE | pval | beta | SE | pval |  |  |
| rs10760201 | 9 | 124641918 | A | G | 0.677669 | -0.0053284 | 0.000870915 | 9.49992E-10 | -0.0215 | 0.0099 | 0.0303403 | 8.11825E-05 | 37.43175711 |
| rs10962171 | 9 | 15812829 | A | G | 0.463127 | 0.00476219 | 0.00081613 | 5.39995E-09 | 0.0121 | 0.0093 | 0.1931 | 7.38445E-05 | 34.04809898 |
| rs11678980 | 2 | 162101261 | A | G | 0.46139 | 0.00524985 | 0.000842044 | 4.49997E-10 | 0.0185 | 0.0096 | 0.0539896 | 8.43031E-05 | 38.87069756 |
| rs11897809 | 2 | 15021145 | C | A | 0.35418 | 0.00479452 | 0.000849763 | 0.000000017 | 0.0053 | 0.0097 | 0.5869 | 6.90431E-05 | 31.83411279 |
| rs12938531 | 17 | 8125541 | G | A | 0.502832 | 0.00478751 | 0.000813496 | 0.000000004 | 0.011 | 0.0093 | 0.2348 | 7.51159E-05 | 34.63431963 |
| rs12950328 | 17 | 46967061 | T | C | 0.539392 | 0.00480014 | 0.000815445 | 3.89996E-09 | 0.0016 | 0.0093 | 0.8609 | 7.51522E-05 | 34.65106411 |
| rs13026283 | 2 | 100842638 | T | C | 0.393454 | -0.00484746 | 0.000832691 | 5.80003E-09 | -0.0188 | 0.0095 | 0.0481704 | 7.34995E-05 | 33.88900695 |
| rs17031936 | 3 | 34615898 | G | A | 0.237496 | -0.00609385 | 0.000956033 | 1.79999E-10 | -0.0011 | 0.0109 | 0.9232 | 8.81161E-05 | 40.62897529 |
| rs2748287 | 6 | 92139101 | A | G | 0.473693 | 0.00448685 | 0.000816096 | 3.79997E-08 | 0.0038 | 0.0093 | 0.6863 | 6.55583E-05 | 30.22725567 |
| rs28529403 | 16 | 30134656 | C | T | 0.398732 | -0.00491871 | 0.000831081 | 3.2E-09 | -0.037 | 0.0095 | 0.0001034 | 7.59694E-05 | 35.0278793 |
| rs35225200 | 4 | 103146888 | C | A | 0.083249 | 0.0112689 | 0.00151372 | 9.70063E-14 | 0.0757 | 0.0173 | 1.18801E-05 | 0.000120192 | 55.42045256 |
| rs360180 | 14 | 47091395 | G | T | 0.610617 | -0.00548382 | 0.000833862 | 4.79954E-11 | -0.0245 | 0.0095 | 0.0100201 | 9.37979E-05 | 43.24900618 |
| rs3756362 | 5 | 122108890 | G | C | 0.530473 | 0.00477938 | 0.000814153 | 4.30002E-09 | 0.0207 | 0.0093 | 0.0260202 | 7.47402E-05 | 34.46110387 |
| rs56348580 | 12 | 121432117 | C | G | 0.307311 | -0.00497762 | 0.000882584 | 0.000000017 | 0.0039 | 0.0101 | 0.7001 | 6.89854E-05 | 31.8074969 |
| rs6458247 | 6 | 41820654 | G | A | 0.732251 | 0.00552538 | 0.000917755 | 1.7E-09 | 0.0128 | 0.0105 | 0.2213 | 7.86126E-05 | 36.24673563 |
| rs6902789 | 6 | 105358192 | A | G | 0.367712 | -0.00494138 | 0.000846164 | 5.19996E-09 | 0.0058 | 0.0096 | 0.5486 | 7.39624E-05 | 34.10244629 |
| rs7794036 | 7 | 104870448 | C | T | 0.529654 | -0.00454915 | 0.000814506 | 2.30001E-08 | -0.0071 | 0.0093 | 0.4456 | 6.76548E-05 | 31.19392723 |
| rs7903146 | 10 | 114758349 | T | C | 0.290668 | 0.00611206 | 0.000894042 | 8.10028E-12 | -0.0331 | 0.0102 | 0.00121199 | 0.000101361 | 46.73664046 |
| rs838133 | 19 | 49259529 | G | A | 0.549378 | 0.00546478 | 0.000840291 | 7.89951E-11 | 0.002 | 0.0096 | 0.8324 | 9.1728E-05 | 42.29451584 |
| rs898980 | 1 | 38658152 | T | C | 0.877965 | 0.00683096 | 0.00124475 | 4.09996E-08 | 0.0072 | 0.0142 | 0.611599 | 6.53171E-05 | 30.11600292 |
| rs9825208 | 3 | 101274807 | T | G | 0.443156 | 0.00514445 | 0.000820353 | 3.59998E-10 | 0.0109 | 0.0094 | 0.2446 | 8.52894E-05 | 39.32552018 |
| Genome-wide significant SNPs for Standing height（ukb-a-389） | | | | | | | | | | | | | |
| SNP | Chr | Position | EA | OA | EAF | Standing height | | | Knee Osteoarthritis | | | R2 | F |
|  |  |  |  |  |  | beta | SE | pval | beta | SE | pval |  |  |
| rs10029732 | 4 | 48498679 | G | A | 0.503149 | 0.0133062 | 0.00172813 | 1.3671E-14 | -0.0064 | 0.0097 | 0.511 | 0.000176168 | 59.2860742 |
| rs10033541 | 4 | 45123920 | A | G | 0.280787 | 0.0104862 | 0.00192032 | 4.74701E-08 | -0.0006 | 0.0104 | 0.957 | 8.86133E-05 | 29.8185473 |
| rs10069931 | 5 | 156797005 | T | C | 0.326708 | -0.0124816 | 0.00184013 | 1.17896E-11 | -0.0054 | 0.0099 | 0.585901 | 0.00013672 | 46.00880233 |
| rs10082476 | 10 | 124164654 | G | A | 0.247868 | -0.0197598 | 0.00200055 | 5.26502E-23 | -0.0357 | 0.0108 | 0.000910102 | 0.00028986 | 97.55817943 |
| rs10084690 | 3 | 33591384 | C | A | 0.0352285 | 0.0364183 | 0.00468466 | 7.62957E-15 | 0.0007 | 0.0252 | 0.9779 | 0.000179578 | 60.43389754 |
| rs10139746 | 14 | 75040338 | G | A | 0.548609 | -0.0154097 | 0.0017332 | 6.08976E-19 | 0.0013 | 0.0093 | 0.8876 | 0.000234875 | 79.0475521 |
| rs10151561 | 14 | 92430184 | G | A | 0.422541 | -0.0222033 | 0.00175337 | 9.64051E-37 | -0.0095 | 0.0094 | 0.3154 | 0.000476354 | 160.3560424 |
| rs10155941 | 7 | 134422732 | C | T | 0.460683 | -0.0134864 | 0.00173119 | 6.70502E-15 | -0.0101 | 0.0093 | 0.2794 | 0.000180332 | 60.68760826 |
| rs10165255 | 2 | 10199601 | G | A | 0.557247 | -0.0189129 | 0.00174443 | 2.20242E-27 | -0.0084 | 0.0094 | 0.3678 | 0.000349226 | 117.5456542 |
| rs1020048 | 9 | 86666993 | C | A | 0.809832 | -0.0187231 | 0.00222023 | 3.38298E-17 | 0.0042 | 0.0119 | 0.7252 | 0.000211308 | 71.11430976 |
| rs10200995 | 2 | 164448385 | A | T | 0.136746 | -0.01556 | 0.00251084 | 5.75705E-10 | 0.0137 | 0.0135 | 0.3076 | 0.000114125 | 38.40418264 |
| rs10202701 | 2 | 232328681 | T | C | 0.54321 | 0.0202842 | 0.00173873 | 1.92619E-31 | 0.001 | 0.0093 | 0.9178 | 0.000404319 | 136.0971064 |
| rs10207579 | 2 | 103103289 | T | C | 0.495007 | 0.0118967 | 0.00173491 | 7.03072E-12 | 0.0069 | 0.0093 | 0.4622 | 0.000139729 | 47.02150639 |
| rs10212258 | 3 | 41349386 | A | G | 0.332356 | 0.0115683 | 0.00183277 | 2.75956E-10 | 0.0195 | 0.0098 | 0.0476497 | 0.000118391 | 39.84011368 |
| rs10215853 | 7 | 20416244 | C | T | 0.472877 | 0.0207996 | 0.00172831 | 2.37465E-33 | 0.0173 | 0.0093 | 0.0623606 | 0.000430257 | 144.831857 |
| rs1025337 | 2 | 141573067 | A | G | 0.546542 | 0.0108671 | 0.00173716 | 3.96232E-10 | 0.0083 | 0.0093 | 0.3754 | 0.000116291 | 39.13317672 |
| rs10401891 | 19 | 4962848 | T | C | 0.327362 | -0.0209169 | 0.00184176 | 6.92947E-30 | -0.0126 | 0.0099 | 0.2041 | 0.000383187 | 128.981073 |
| rs10403206 | 19 | 46967912 | C | T | 0.96337 | 0.0277326 | 0.00459559 | 1.59518E-09 | 0.0154 | 0.0247 | 0.5326 | 0.000108218 | 36.4163188 |
| rs1043413 | 19 | 41939297 | G | C | 0.388111 | 0.0212344 | 0.00176746 | 3.04369E-33 | 0.0174 | 0.0095 | 0.0664492 | 0.000428788 | 144.337181 |
| rs1043547 | 14 | 93406789 | T | G | 0.249392 | -0.0123796 | 0.00201828 | 8.5929E-10 | -0.0078 | 0.0108 | 0.4715 | 0.000111802 | 37.62251378 |
| rs1044322 | 10 | 89623323 | A | G | 0.0131762 | -0.0487593 | 0.00754969 | 1.0592E-10 | -0.0091 | 0.0405 | 0.8219 | 0.000123951 | 41.71133637 |
| rs10448110 | 8 | 4809797 | C | A | 0.465377 | -0.011496 | 0.00173798 | 3.73164E-11 | 0.0175 | 0.0093 | 0.0611406 | 0.000130016 | 43.75234953 |
| rs10498672 | 6 | 7797840 | G | C | 0.177045 | 0.0215592 | 0.00225916 | 1.39605E-21 | 0.0087 | 0.0122 | 0.4739 | 0.000270584 | 91.06861284 |
| rs1065368 | 16 | 1036860 | C | T | 0.469448 | 0.0146961 | 0.00177137 | 1.07671E-16 | 0.0304 | 0.0095 | 0.001373 | 0.000204524 | 68.83083612 |
| rs10737170 | 1 | 156063880 | A | C | 0.908778 | -0.0174324 | 0.00302323 | 8.11764E-09 | 0.0101 | 0.0162 | 0.5327 | 9.88047E-05 | 33.2482971 |
| rs1074683 | 20 | 32304653 | G | C | 0.261856 | -0.0303983 | 0.00196594 | 6.48933E-54 | 0.0068 | 0.0106 | 0.52 | 0.000710065 | 239.0867419 |
| rs10748128 | 12 | 69827658 | T | G | 0.344848 | 0.0265161 | 0.00181522 | 2.59717E-48 | 0.0298 | 0.0098 | 0.00223501 | 0.000633773 | 213.3821954 |
| rs10756792 | 9 | 16726119 | T | C | 0.744728 | 0.0161738 | 0.00198667 | 3.92826E-16 | -0.0332 | 0.0107 | 0.00187301 | 0.000196941 | 66.27810581 |
| rs10765754 | 11 | 95348025 | G | A | 0.348718 | 0.0125067 | 0.00182275 | 6.8281E-12 | -0.0059 | 0.0098 | 0.5455 | 0.0001399 | 47.07915613 |
| rs10817161 | 9 | 113960572 | C | G | 0.569873 | -0.0106514 | 0.00174924 | 1.13624E-09 | -0.0015 | 0.0094 | 0.8696 | 0.000110183 | 37.07763353 |
| rs10826579 | 10 | 29491536 | A | G | 0.153527 | -0.0157053 | 0.00244216 | 1.27022E-10 | -0.006 | 0.0131 | 0.6446 | 0.000122896 | 41.3562988 |
| rs10851704 | 15 | 62202482 | T | C | 0.545436 | -0.0122552 | 0.00173186 | 1.4832E-12 | -0.0035 | 0.0093 | 0.7105 | 0.000148799 | 50.07404342 |
| rs10870597 | 13 | 114999636 | G | A | 0.234812 | -0.0169386 | 0.00203461 | 8.4489E-17 | 0.0099 | 0.0109 | 0.3668 | 0.000205945 | 69.30907359 |
| rs10874746 | 1 | 93323971 | C | T | 0.655199 | 0.0178725 | 0.00181328 | 6.47441E-23 | 0.0117 | 0.0098 | 0.2305 | 0.000288645 | 97.1489894 |
| rs10877013 | 12 | 58165085 | T | C | 0.327228 | -0.0124485 | 0.00184241 | 1.41416E-11 | 0.0003 | 0.0099 | 0.9752 | 0.00013566 | 45.65190465 |
| rs10913200 | 1 | 176521655 | A | G | 0.0288672 | -0.0684155 | 0.00514067 | 2.11057E-40 | -0.0252 | 0.0278 | 0.365 | 0.000526126 | 177.1197448 |
| rs10914505 | 1 | 32393578 | T | G | 0.132601 | 0.0273837 | 0.0025449 | 5.35797E-27 | 0.0098 | 0.0137 | 0.4747 | 0.000343987 | 115.7817826 |
| rs10916606 | 1 | 224570425 | T | C | 0.789658 | 0.0186426 | 0.00212382 | 1.6734E-18 | 0.0044 | 0.0114 | 0.696101 | 0.000228943 | 77.05041262 |
| rs10922475 | 1 | 89142142 | A | C | 0.537727 | 0.0204339 | 0.00172882 | 3.13979E-32 | 0.0148 | 0.0093 | 0.1114 | 0.000415022 | 139.7012814 |
| rs10937460 | 3 | 190851583 | A | G | 0.85829 | -0.0155116 | 0.00248698 | 4.46252E-10 | 0.0137 | 0.0134 | 0.3041 | 0.000115602 | 38.90147129 |
| rs10952289 | 7 | 150524681 | C | T | 0.334807 | 0.0181168 | 0.00182893 | 3.96187E-23 | -0.0376 | 0.0098 | 0.000131701 | 0.000291535 | 98.12196369 |
| rs10995318 | 10 | 52762249 | G | A | 0.234872 | -0.017598 | 0.00203693 | 5.67283E-18 | 0.0265 | 0.011 | 0.01562 | 0.000221782 | 74.64003531 |
| rs10998288 | 10 | 70331038 | T | C | 0.395855 | 0.0178219 | 0.00176489 | 5.68329E-24 | 0.03 | 0.0095 | 0.00156401 | 0.000302963 | 101.9694645 |
| rs11014285 | 10 | 25178864 | A | G | 0.165536 | 0.0205196 | 0.00235188 | 2.67855E-18 | 0.0083 | 0.0126 | 0.5115 | 0.000226182 | 76.12106955 |
| rs11047211 | 12 | 24171106 | G | C | 0.598541 | -0.00965167 | 0.0017701 | 4.96684E-08 | -0.0029 | 0.0095 | 0.762801 | 8.83526E-05 | 29.73081014 |
| rs11077964 | 17 | 79966487 | G | T | 0.705035 | 0.0147458 | 0.00192114 | 1.65158E-14 | 0.0114 | 0.0103 | 0.271 | 0.000175062 | 58.91366866 |
| rs11111305 | 12 | 102961450 | T | C | 0.144692 | 0.0230361 | 0.0025301 | 8.6876E-20 | 0.0199 | 0.0136 | 0.1426 | 0.000246311 | 82.89721791 |
| rs11144782 | 9 | 78795213 | G | C | 0.174923 | -0.0131971 | 0.00227624 | 6.72574E-09 | -0.0311 | 0.0122 | 0.01096 | 9.98909E-05 | 33.61385574 |
| rs11152363 | 18 | 53057188 | A | G | 0.184703 | 0.0125252 | 0.00224272 | 2.34159E-08 | 0.0068 | 0.012 | 0.5706 | 9.26888E-05 | 31.19009124 |
| rs11161673 | 1 | 86258390 | A | C | 0.161626 | -0.0142954 | 0.00237007 | 1.62491E-09 | -0.0343 | 0.0128 | 0.00721606 | 0.000108111 | 36.38041896 |
| rs11205303 | 1 | 149906413 | C | T | 0.409126 | 0.039022 | 0.00175552 | 2.2029E-109 | -0.0095 | 0.0099 | 0.34 | 0.001466286 | 494.0886807 |
| rs11233117 | 11 | 69924352 | G | C | 0.452229 | -0.0112539 | 0.00173442 | 8.67961E-11 | -0.019 | 0.0093 | 0.0409902 | 0.00012511 | 42.10124865 |
| rs11245333 | 10 | 126383088 | A | C | 0.404196 | 0.0138422 | 0.00176265 | 4.07099E-15 | 0.0018 | 0.0095 | 0.851 | 0.000183251 | 61.67021857 |
| rs11252860 | 10 | 5024356 | C | A | 0.745697 | 0.0156201 | 0.00203905 | 1.85738E-14 | 0.0218 | 0.1475 | 0.8824 | 0.000174375 | 58.68259192 |
| rs11252860 | 10 | 5024356 | C | A | 0.745697 | 0.0156201 | 0.00203905 | 1.85738E-14 | -0.0088 | 0.0109 | 0.4206 | 0.000174375 | 58.68259192 |
| rs11259936 | 15 | 84580582 | C | A | 0.521001 | 0.0373656 | 0.00172559 | 6.5917E-104 | -0.0191 | 0.0093 | 0.0393604 | 0.001391594 | 468.8847507 |
| rs112705086 | 2 | 1647769 | A | G | 0.075835 | 0.0206794 | 0.00328354 | 3.02072E-10 | 0.013 | 0.0176 | 0.4613 | 0.000117866 | 39.66328346 |
| rs112957890 | 14 | 36220876 | G | A | 0.265827 | 0.015573 | 0.00197056 | 2.73401E-15 | 0.0012 | 0.0106 | 0.9062 | 0.000185581 | 62.45434529 |
| rs1132148 | 1 | 172363526 | G | A | 0.189892 | 0.0216587 | 0.00219945 | 7.08761E-23 | 0.0042 | 0.0118 | 0.7214 | 0.000288111 | 96.96924248 |
| rs114693875 | 2 | 200323452 | G | A | 0.169035 | -0.0160706 | 0.00230339 | 3.02343E-12 | 0.0048 | 0.0124 | 0.698499 | 0.000144649 | 48.67731866 |
| rs114784809 | 5 | 127674221 | A | G | 0.0264265 | 0.033936 | 0.00546167 | 5.18824E-10 | 0.0119 | 0.0295 | 0.6875 | 0.000114728 | 38.60715585 |
| rs115227414 | 2 | 135818487 | C | T | 0.0447243 | -0.0232458 | 0.00418948 | 2.88184E-08 | 0.0289 | 0.0223 | 0.196 | 9.14908E-05 | 30.78691477 |
| rs11553764 | 12 | 104415244 | T | C | 0.16873 | 0.0136886 | 0.00230873 | 3.05014E-09 | -0.0008 | 0.0124 | 0.9477 | 0.000104466 | 35.15355024 |
| rs11555134 | 7 | 50659193 | T | C | 0.233178 | 0.0163403 | 0.00205726 | 1.98335E-15 | -0.0131 | 0.011 | 0.2358 | 0.00018746 | 63.08688817 |
| rs11588850 | 1 | 227927242 | G | A | 0.172177 | -0.0343674 | 0.00229435 | 1.04376E-50 | 0.0008 | 0.0124 | 0.946 | 0.000666397 | 224.3734339 |
| rs11591214 | 1 | 22485138 | A | G | 0.129673 | -0.0218034 | 0.00257544 | 2.55211E-17 | 0.0153 | 0.0138 | 0.2682 | 0.000212962 | 71.67092177 |
| rs11600815 | 11 | 14797227 | A | G | 0.0536908 | 0.0323128 | 0.00395716 | 3.20848E-16 | 0.0075 | 0.0213 | 0.724101 | 0.000198127 | 66.67751146 |
| rs11618507 | 13 | 30172751 | T | G | 0.229198 | 0.0151572 | 0.00205818 | 1.78361E-13 | 0.0174 | 0.0111 | 0.1151 | 0.000161157 | 54.23362995 |
| rs11664336 | 18 | 46604851 | T | A | 0.433938 | 0.0253235 | 0.00174052 | 6.09397E-48 | -0.04 | 0.0094 | 0.00001986 | 0.000628731 | 211.6834195 |
| rs11666304 | 19 | 17505196 | G | T | 0.832172 | 0.0144985 | 0.00230968 | 3.44953E-10 | 0.0058 | 0.0124 | 0.6386 | 0.000117095 | 39.40396611 |
| rs11667331 | 19 | 31050571 | G | A | 0.165932 | 0.0143091 | 0.00232322 | 7.32032E-10 | -0.0434 | 0.0125 | 0.000525303 | 0.000112731 | 37.93511287 |
| rs11678288 | 2 | 65774687 | A | T | 0.591403 | -0.0102398 | 0.00176438 | 6.49516E-09 | -0.0077 | 0.0095 | 0.4154 | 0.000100093 | 33.68186374 |
| rs116782923 | 5 | 102331465 | T | A | 0.0537235 | -0.02117 | 0.00384323 | 3.62376E-08 | 0.0631 | 0.0206 | 0.00219801 | 9.01692E-05 | 30.34215223 |
| rs11681299 | 2 | 88901732 | T | C | 0.288826 | 0.022783 | 0.00190147 | 4.49469E-33 | 0.0033 | 0.0102 | 0.7493 | 0.000426487 | 143.5622589 |
| rs116920478 | 21 | 28442933 | T | C | 0.0653175 | -0.0223587 | 0.00352174 | 2.17325E-10 | 0.0208 | 0.019 | 0.274 | 0.000119777 | 40.3065787 |
| rs11722554 | 4 | 5016883 | A | G | 0.037125 | -0.0369234 | 0.00456497 | 6.06457E-16 | -0.0368 | 0.0244 | 0.1317 | 0.000194398 | 65.42216819 |
| rs11731421 | 4 | 1749160 | A | G | 0.332928 | 0.0181407 | 0.00183258 | 4.23643E-23 | 0.05 | 0.0099 | 3.89099E-07 | 0.000291142 | 97.98951678 |
| rs11738691 | 5 | 54880229 | T | G | 0.662511 | -0.0242446 | 0.00182759 | 3.73938E-40 | 0.0025 | 0.0098 | 0.8017 | 0.00052275 | 175.9826767 |
| rs11744543 | 5 | 67100580 | T | A | 0.161193 | 0.0212337 | 0.00235328 | 1.83908E-19 | 0.0005 | 0.0127 | 0.9692 | 0.000241907 | 81.41446346 |
| rs11746047 | 5 | 123997308 | T | C | 0.38247 | 0.0128192 | 0.00178272 | 6.45506E-13 | 0.0182 | 0.0096 | 0.0579202 | 0.000153652 | 51.70743463 |
| rs11747997 | 5 | 142790752 | C | A | 0.329762 | 0.0112834 | 0.00184333 | 9.29587E-10 | -0.0026 | 0.0099 | 0.7926 | 0.000111346 | 37.46892254 |
| rs11774206 | 8 | 144375815 | C | T | 0.765589 | -0.0160877 | 0.00203448 | 2.6333E-15 | -0.0026 | 0.0109 | 0.8098 | 0.000185801 | 62.52856859 |
| rs11775903 | 8 | 25321076 | G | A | 0.399028 | 0.0107601 | 0.00176056 | 9.86734E-10 | 0.0047 | 0.0095 | 0.6204 | 0.000111002 | 37.35324812 |
| rs11777239 | 8 | 145031265 | A | G | 0.400971 | -0.0209863 | 0.00176633 | 1.50245E-32 | 0.0013 | 0.0095 | 0.8925 | 0.000419367 | 141.164496 |
| rs1178162 | 7 | 18786967 | A | C | 0.207358 | 0.0177682 | 0.00212933 | 7.1796E-17 | 0.0104 | 0.0114 | 0.3617 | 0.0002069 | 69.63031194 |
| rs11783086 | 8 | 123980448 | C | T | 0.35356 | 0.0158071 | 0.00181338 | 2.87012E-18 | -0.0116 | 0.0097 | 0.2324 | 0.000225776 | 75.98435426 |
| rs117982737 | 8 | 26241010 | T | C | 0.236985 | -0.0119895 | 0.00204194 | 4.31966E-09 | 0.0012 | 0.011 | 0.9134 | 0.000102452 | 34.47574098 |
| rs118083081 | 16 | 15504423 | T | C | 0.0272436 | -0.0301975 | 0.00533596 | 1.5217E-08 | -0.0212 | 0.0291 | 0.4652 | 9.51752E-05 | 32.0268531 |
| rs11864330 | 16 | 86724388 | A | G | 0.214778 | -0.0123496 | 0.00211162 | 4.96695E-09 | 0.014 | 0.0113 | 0.2182 | 0.000101643 | 34.20358807 |
| rs11955153 | 5 | 170864548 | C | T | 0.235492 | -0.0256313 | 0.00203539 | 2.35776E-36 | -0.0218 | 0.011 | 0.0469797 | 0.000471075 | 158.5781742 |
| rs12051048 | 16 | 783864 | A | C | 0.230661 | 0.0282529 | 0.00205003 | 3.37676E-43 | -0.0088 | 0.011 | 0.4226 | 0.000564169 | 189.9341467 |
| rs12103006 | 16 | 24726237 | G | A | 0.570767 | 0.0132454 | 0.00174642 | 3.35197E-14 | 0.0066 | 0.0094 | 0.4809 | 0.000170926 | 57.5214973 |
| rs12216030 | 6 | 22103237 | T | G | 0.472475 | 0.0102168 | 0.0017287 | 3.42224E-09 | 0.02 | 0.0093 | 0.0315602 | 0.000103799 | 34.92914366 |
| rs12269901 | 11 | 116973929 | C | G | 0.284485 | -0.0104965 | 0.00190826 | 3.788E-08 | 0.0169 | 0.0103 | 0.1001 | 8.99132E-05 | 30.25598839 |
| rs12296480 | 12 | 1514912 | C | T | 0.116556 | -0.0163469 | 0.00271229 | 1.67236E-09 | -0.005 | 0.0145 | 0.732399 | 0.000107944 | 36.32419386 |
| rs12306741 | 12 | 24033225 | A | G | 0.660296 | -0.0117404 | 0.00182755 | 1.32807E-10 | 0.0165 | 0.0098 | 0.0934308 | 0.000122637 | 41.26906891 |
| rs12312144 | 12 | 47092662 | A | C | 0.180419 | 0.0127817 | 0.00224674 | 1.27894E-08 | -0.0016 | 0.0121 | 0.8969 | 9.61786E-05 | 32.36450908 |
| rs12347137 | 9 | 119122721 | C | A | 0.203743 | -0.0311773 | 0.00214261 | 5.94566E-48 | -0.0052 | 0.0115 | 0.654 | 0.000628877 | 211.732813 |
| rs12404594 | 1 | 182988027 | A | G | 0.549235 | -0.0137987 | 0.00173301 | 1.69395E-15 | -0.0133 | 0.0093 | 0.154 | 0.000188383 | 63.39742614 |
| rs12408482 | 1 | 54112148 | A | G | 0.240437 | 0.0147946 | 0.00202208 | 2.55094E-13 | 0.0058 | 0.0109 | 0.595701 | 0.00015907 | 53.53122805 |
| rs12417930 | 11 | 127986202 | A | G | 0.182481 | -0.0168159 | 0.00223305 | 5.06874E-14 | 0.0136 | 0.012 | 0.2561 | 0.000168507 | 56.70753321 |
| rs12452505 | 17 | 63556402 | G | C | 0.140525 | -0.0334793 | 0.00249066 | 3.51884E-41 | -0.0029 | 0.0133 | 0.8271 | 0.000536709 | 180.6846524 |
| rs12458880 | 18 | 29794353 | C | T | 0.0637413 | -0.0233554 | 0.00353158 | 3.76444E-11 | 0.0185 | 0.019 | 0.3322 | 0.000129966 | 43.73548436 |
| rs12483653 | 21 | 17355049 | T | C | 0.780315 | -0.0140727 | 0.00210158 | 2.1424E-11 | -0.0146 | 0.0113 | 0.1954 | 0.000133246 | 44.83946519 |
| rs12529733 | 6 | 109654132 | C | T | 0.421269 | -0.0185987 | 0.00175605 | 3.30446E-26 | 0.0017 | 0.0094 | 0.8535 | 0.000333269 | 112.173131 |
| rs12533527 | 7 | 130626948 | T | C | 0.140622 | -0.0188885 | 0.00250643 | 4.85736E-14 | -0.0334 | 0.0135 | 0.0134899 | 0.000168756 | 56.79122013 |
| rs12572775 | 10 | 104285594 | T | A | 0.556692 | 0.0230505 | 0.00173624 | 3.25987E-40 | 0.0048 | 0.0093 | 0.6051 | 0.000523555 | 176.2538468 |
| rs12594720 | 15 | 67007018 | G | C | 0.271435 | 0.0165007 | 0.00195703 | 3.4261E-17 | -0.0058 | 0.0105 | 0.5798 | 0.000211235 | 71.08978309 |
| rs12615742 | 2 | 37995727 | T | C | 0.505196 | 0.0211311 | 0.00176725 | 6.05759E-33 | -0.0018 | 0.0095 | 0.8462 | 0.000424729 | 142.9702407 |
| rs12628193 | 22 | 20791438 | A | C | 0.376269 | 0.0142301 | 0.00177821 | 1.22321E-15 | 0.0019 | 0.0096 | 0.842 | 0.00019029 | 64.03939586 |
| rs12634315 | 3 | 157728016 | C | T | 0.767275 | -0.0186419 | 0.00204112 | 6.68498E-20 | 0.0188 | 0.011 | 0.0867601 | 0.000247847 | 83.41433467 |
| rs12656497 | 5 | 32831939 | C | T | 0.596357 | -0.0256624 | 0.00175914 | 3.46019E-48 | -0.0126 | 0.0095 | 0.183 | 0.000632074 | 212.8095684 |
| rs12682937 | 9 | 108924249 | A | G | 0.278505 | -0.0196621 | 0.00193085 | 2.37739E-24 | 0.0007 | 0.0104 | 0.9499 | 0.00030809 | 103.6955565 |
| rs12740738 | 1 | 103542260 | G | A | 0.574124 | -0.0179228 | 0.00174588 | 1.01344E-24 | -0.0107 | 0.0094 | 0.252 | 0.000313109 | 105.3853776 |
| rs12900132 | 15 | 100537494 | T | C | 0.636501 | 0.0119224 | 0.00179025 | 2.74979E-11 | -0.0035 | 0.0096 | 0.714399 | 0.000131793 | 44.35038872 |
| rs12942811 | 17 | 76406692 | C | T | 0.658189 | 0.0117555 | 0.0019258 | 1.03405E-09 | -0.0062 | 0.0103 | 0.5482 | 0.000110729 | 37.26123674 |
| rs12948439 | 17 | 46967124 | A | G | 0.541809 | -0.0238998 | 0.00173196 | 2.64484E-43 | 0.0013 | 0.0093 | 0.8913 | 0.000565608 | 190.4189809 |
| rs12952981 | 17 | 79409189 | A | G | 0.35575 | 0.0130456 | 0.00180486 | 4.90908E-13 | 0.0177 | 0.0097 | 0.0676597 | 0.000155247 | 52.2442495 |
| rs12968652 | 18 | 46501070 | A | G | 0.398387 | -0.0134999 | 0.00178141 | 3.51156E-14 | -0.0184 | 0.0096 | 0.05424 | 0.000170651 | 57.42893331 |
| rs12981554 | 19 | 3416668 | G | A | 0.542554 | -0.0160136 | 0.00174304 | 4.05509E-20 | -0.0005 | 0.0094 | 0.9537 | 0.000250786 | 84.40354035 |
| rs12985850 | 19 | 2155042 | A | G | 0.400221 | 0.0285025 | 0.00176754 | 1.77542E-58 | 0.0205 | 0.0095 | 0.0310099 | 0.000772219 | 260.0308201 |
| rs13021881 | 2 | 33452191 | C | T | 0.521434 | 0.0132448 | 0.00173966 | 2.67609E-14 | -0.0598 | 0.0093 | 1.548E-10 | 0.000172241 | 57.96415009 |
| rs13080165 | 3 | 25391116 | G | A | 0.237578 | 0.0121661 | 0.00202736 | 1.96332E-09 | 0.0087 | 0.0109 | 0.4231 | 0.000107015 | 36.01126978 |
| rs13126069 | 4 | 152212486 | T | C | 0.438434 | 0.0101599 | 0.00173929 | 5.1812E-09 | -0.0181 | 0.0094 | 0.0527206 | 0.0001014 | 34.12182767 |
| rs13168903 | 5 | 158517233 | G | T | 0.414552 | -0.00958748 | 0.00175258 | 4.49107E-08 | 0.0091 | 0.0094 | 0.3331 | 8.8933E-05 | 29.92613687 |
| rs1317149 | 11 | 47486885 | T | C | 0.350455 | -0.0229719 | 0.00180659 | 4.93856E-37 | -0.0185 | 0.0097 | 0.0568303 | 0.000480302 | 161.6858629 |
| rs1317867 | 17 | 21276114 | G | A | 0.509607 | 0.0209217 | 0.00174158 | 3.0839E-33 | 0.0036 | 0.0094 | 0.6967 | 0.000428716 | 144.3126833 |
| rs13210323 | 6 | 35005084 | C | A | 0.280377 | -0.0300151 | 0.0019243 | 7.85416E-55 | 0.0069 | 0.0103 | 0.5036 | 0.000722551 | 243.2940277 |
| rs13238709 | 7 | 100783736 | T | C | 0.431962 | 0.010599 | 0.00173975 | 1.11445E-09 | 0.004 | 0.0093 | 0.6651 | 0.000110295 | 37.11534591 |
| rs13252528 | 8 | 117505270 | G | A | 0.519585 | -0.00995477 | 0.00173551 | 9.70577E-09 | -0.005 | 0.0093 | 0.5882 | 9.77719E-05 | 32.90073789 |
| rs1327313 | 13 | 80677558 | A | T | 0.218682 | 0.0145483 | 0.00208715 | 3.16665E-12 | 0.0132 | 0.0112 | 0.238 | 0.000144379 | 48.58638534 |
| rs13334364 | 16 | 67332365 | C | T | 0.0747956 | -0.0306756 | 0.00328037 | 8.71164E-21 | 0.0364 | 0.0177 | 0.0400904 | 0.000259822 | 87.44558505 |
| rs13401747 | 2 | 218284814 | C | T | 0.3147 | -0.0206501 | 0.00186065 | 1.293E-28 | -0.0133 | 0.01 | 0.1826 | 0.000365935 | 123.1721046 |
| rs134092 | 22 | 28068444 | T | A | 0.494244 | 0.00992545 | 0.00173377 | 1.0366E-08 | -0.019 | 0.0093 | 0.04128 | 9.73921E-05 | 32.77289922 |
| rs13430869 | 2 | 218146818 | T | G | 0.747688 | 0.0160628 | 0.00199004 | 6.96145E-16 | 0.0351 | 0.0107 | 0.000984895 | 0.00019359 | 65.15028362 |
| rs1345991 | 6 | 143689515 | T | C | 0.574397 | 0.00984882 | 0.00174345 | 1.61469E-08 | -0.0065 | 0.0094 | 0.490601 | 9.48324E-05 | 31.91147109 |
| rs1352090 | 7 | 46193843 | G | C | 0.371116 | 0.0131679 | 0.00178716 | 1.7354E-13 | 0.0331 | 0.0096 | 0.000568997 | 0.000161319 | 54.28796808 |
| rs1355603 | 4 | 145566477 | C | T | 0.830705 | 0.0624867 | 0.00229834 | 1.3614E-162 | 0.0135 | 0.0124 | 0.2755 | 0.002192008 | 739.1695141 |
| rs1360504 | 1 | 56627905 | A | G | 0.192263 | 0.016351 | 0.00219057 | 8.39847E-14 | -0.009 | 0.0118 | 0.4463 | 0.000165558 | 55.71495502 |
| rs1366594 | 5 | 88376061 | C | A | 0.46933 | -0.0221856 | 0.0017278 | 9.9426E-38 | -0.0352 | 0.0093 | 0.000154198 | 0.000489769 | 164.8742512 |
| rs1369703 | 2 | 25481898 | C | T | 0.417142 | 0.0302393 | 0.00175536 | 1.78484E-66 | 0.0274 | 0.0094 | 0.00363999 | 0.000881204 | 296.7621374 |
| rs137933811 | 12 | 120755120 | G | C | 0.0301617 | -0.0371639 | 0.00506166 | 2.10523E-13 | 0.0215 | 0.0272 | 0.4291 | 0.00016019 | 53.90810246 |
| rs138809270 | 2 | 219929394 | T | C | 0.0169381 | -0.0846533 | 0.00685539 | 5.05475E-35 | 0.0593 | 0.0368 | 0.1071 | 0.000452976 | 152.4828044 |
| rs138937927 | 16 | 51095561 | T | C | 0.0263835 | -0.0465272 | 0.00557695 | 7.28283E-17 | -0.1031 | 0.0303 | 0.000677205 | 0.000206814 | 69.6013642 |
| rs1393421 | 15 | 38369210 | A | G | 0.936456 | 0.0193816 | 0.00354441 | 4.54915E-08 | 0.0425 | 0.019 | 0.0257502 | 8.8859E-05 | 29.90121012 |
| rs1395602 | 16 | 4911239 | C | A | 0.482599 | 0.0119952 | 0.00173226 | 4.38026E-12 | 0.0094 | 0.0093 | 0.3105 | 0.000142487 | 47.94973942 |
| rs1403987 | 7 | 38111941 | G | A | 0.654751 | 0.0173905 | 0.00181494 | 9.58959E-22 | 0.0082 | 0.0097 | 0.3983 | 0.000272791 | 91.81148489 |
| rs140574823 | 20 | 49161754 | C | A | 0.0808617 | 0.0190739 | 0.00316636 | 1.7042E-09 | -0.0355 | 0.017 | 0.0371604 | 0.000107835 | 36.28739902 |
| rs141495779 | 14 | 89307031 | G | A | 0.015525 | -0.0394228 | 0.00699633 | 1.7542E-08 | -0.0279 | 0.0377 | 0.4591 | 9.43543E-05 | 31.75058856 |
| rs1415701 | 6 | 130345835 | A | G | 0.267014 | -0.0331987 | 0.00197301 | 1.65959E-63 | 0.0084 | 0.0106 | 0.4305 | 0.00084075 | 283.1268137 |
| rs1417488 | 1 | 218523730 | T | C | 0.254532 | 0.0161427 | 0.00199158 | 5.27108E-16 | 0.0165 | 0.0107 | 0.124 | 0.000195218 | 65.69831899 |
| rs142190120 | 2 | 219953832 | T | C | 0.013111 | -0.0505985 | 0.00774088 | 6.30522E-11 | 0.0463 | 0.0413 | 0.2626 | 0.000126966 | 42.72598002 |
| rs142669291 | 13 | 32695207 | G | A | 0.0941521 | 0.0164238 | 0.00295647 | 2.77485E-08 | -0.0128 | 0.0159 | 0.4225 | 9.17084E-05 | 30.86013071 |
| rs143222446 | 7 | 101043928 | T | C | 0.0504855 | -0.0246638 | 0.00419912 | 4.26933E-09 | 0.0286 | 0.0225 | 0.2038 | 0.00010252 | 34.49854882 |
| rs143384 | 20 | 34025756 | G | A | 0.402796 | 0.0641051 | 0.00175427 | 1E-200 | -0.0935 | 0.0095 | 4.77309E-23 | 0.003952944 | 1335.333393 |
| rs143743568 | 22 | 39289014 | A | G | 0.153644 | 0.015745 | 0.0024001 | 5.3827E-11 | 0.0045 | 0.0129 | 0.7248 | 0.000127885 | 43.03522467 |
| rs143783471 | 2 | 232834177 | T | C | 0.0134935 | -0.0672609 | 0.00762413 | 1.1285E-18 | -0.0241 | 0.041 | 0.5563 | 0.000231256 | 77.82913096 |
| rs143840904 | 11 | 2813322 | T | C | 0.0192646 | -0.104656 | 0.00647581 | 9.98849E-59 | 0.0586 | 0.0343 | 0.0875105 | 0.000775625 | 261.1785377 |
| rs1443536 | 4 | 82174165 | G | A | 0.304922 | 0.0331584 | 0.00187578 | 6.7655E-70 | 0.0195 | 0.0101 | 0.0536896 | 0.000927831 | 312.4789374 |
| rs1443749 | 7 | 121960438 | T | C | 0.363546 | 0.0116215 | 0.00179371 | 9.24485E-11 | 0.0208 | 0.0096 | 0.0305197 | 0.000124742 | 41.97757371 |
| rs145435234 | 6 | 75311925 | T | A | 0.0398311 | -0.0258382 | 0.00440597 | 4.51274E-09 | -0.0283 | 0.0237 | 0.2326 | 0.000102199 | 34.39052915 |
| rs1467847 | 21 | 35714544 | C | G | 0.567837 | 0.0176471 | 0.00174457 | 4.75664E-24 | -0.0043 | 0.0094 | 0.6448 | 0.000304009 | 102.3215975 |
| rs146851424 | 13 | 50377910 | C | A | 0.0222599 | 0.104464 | 0.00590781 | 6.17021E-70 | 0.0058 | 0.032 | 0.8558 | 0.000928379 | 312.6638576 |
| rs147110934 | 19 | 55993436 | T | G | 0.024103 | -0.071485 | 0.00562024 | 4.71628E-37 | -0.0427 | 0.0304 | 0.1602 | 0.000480574 | 161.7772834 |
| rs147478180 | 15 | 51180777 | T | C | 0.00700921 | -0.058171 | 0.0105723 | 3.75284E-08 | 0.1839 | 0.0571 | 0.00128301 | 8.99671E-05 | 30.27411955 |
| rs148537227 | 7 | 148622699 | A | G | 0.0274675 | 0.0583378 | 0.0054245 | 5.70164E-27 | -0.0199 | 0.0295 | 0.500299 | 0.000343621 | 115.6586413 |
| rs148683230 | 11 | 46141422 | A | G | 0.150235 | 0.0206034 | 0.00242617 | 2.03657E-17 | 0.0162 | 0.013 | 0.2145 | 0.000214284 | 72.116186 |
| rs1490384 | 6 | 126851160 | T | C | 0.498187 | 0.0363154 | 0.00172465 | 2.29879E-98 | -0.0303 | 0.0093 | 0.001073 | 0.001316001 | 443.3810599 |
| rs151123887 | 20 | 34593288 | A | G | 0.0225534 | -0.0414578 | 0.00611485 | 1.20504E-11 | 0.0623 | 0.0346 | 0.0717596 | 0.000136594 | 45.9661687 |
| rs1533269 | 3 | 114214611 | A | C | 0.304055 | -0.0125687 | 0.00187903 | 2.25113E-11 | -0.0099 | 0.0101 | 0.3261 | 0.000132955 | 44.74151762 |
| rs1535197 | 6 | 132539860 | A | T | 0.413619 | 0.00988251 | 0.00175407 | 1.76177E-08 | 0.0194 | 0.0094 | 0.0396497 | 9.43296E-05 | 31.7422788 |
| rs153560 | 5 | 112254377 | A | G | 0.605622 | 0.0106324 | 0.00177925 | 2.29203E-09 | 0.0305 | 0.0096 | 0.00142298 | 0.000106118 | 35.70969025 |
| rs1573891 | 15 | 99186488 | C | G | 0.156436 | -0.0364775 | 0.0023893 | 1.32099E-52 | 0.0341 | 0.0128 | 0.00792994 | 0.00069224 | 233.0806307 |
| rs1574220 | 5 | 314518 | C | T | 0.0820406 | 0.0221028 | 0.00319068 | 4.29635E-12 | 0.0274 | 0.0171 | 0.1095 | 0.000142598 | 47.98721063 |
| rs1593071 | 5 | 42786309 | C | A | 0.452291 | -0.0198207 | 0.00173342 | 2.84971E-30 | 0.0046 | 0.0093 | 0.619699 | 0.000388428 | 130.7458127 |
| rs1599473 | 8 | 120475358 | T | G | 0.243251 | -0.0218248 | 0.00201707 | 2.79512E-27 | 0.0002 | 0.0108 | 0.9826 | 0.000347821 | 117.0728049 |
| rs16824590 | 2 | 203137347 | A | G | 0.11307 | -0.0181549 | 0.00272068 | 2.51131E-11 | 0.0016 | 0.0146 | 0.9131 | 0.000132319 | 44.52769483 |
| rs16905189 | 8 | 135598132 | G | C | 0.367489 | -0.0246773 | 0.00179505 | 5.42126E-43 | -0.0085 | 0.0096 | 0.3796 | 0.000561367 | 188.9903372 |
| rs16942324 | 15 | 89383854 | A | C | 0.0262308 | -0.0968407 | 0.00539208 | 4.3481E-72 | 0.0197 | 0.0287 | 0.4934 | 0.000957713 | 322.5526428 |
| rs17038164 | 1 | 118862669 | C | T | 0.254615 | -0.0327781 | 0.00197929 | 1.42233E-61 | 0.0181 | 0.0106 | 0.0885401 | 0.00081441 | 274.2496672 |
| rs17157112 | 7 | 28779946 | G | T | 0.470735 | -0.0180806 | 0.00173644 | 2.19483E-25 | -0.0025 | 0.0093 | 0.7902 | 0.000322118 | 108.4185344 |
| rs17197114 | 14 | 21894526 | C | T | 0.177444 | 0.0170419 | 0.00227644 | 7.10559E-14 | 0.0114 | 0.0122 | 0.3502 | 0.000166533 | 56.04295513 |
| rs1729137 | 8 | 13180014 | T | C | 0.382144 | -0.00974966 | 0.00177936 | 4.27287E-08 | 0.0112 | 0.0096 | 0.2409 | 8.92198E-05 | 30.02262857 |
| rs17378391 | 20 | 47772601 | C | T | 0.240533 | 0.0275376 | 0.00201911 | 2.42717E-42 | -0.0006 | 0.0109 | 0.9575 | 0.000552511 | 186.0071479 |
| rs17400325 | 2 | 178565913 | C | T | 0.0410336 | 0.0418546 | 0.00434537 | 5.89658E-22 | -0.0246 | 0.0234 | 0.2931 | 0.000275652 | 92.77485552 |
| rs1741288 | 20 | 4102954 | A | G | 0.632978 | -0.0238336 | 0.00179775 | 4.1783E-40 | -0.0154 | 0.0097 | 0.1115 | 0.000522086 | 175.7592199 |
| rs174600 | 11 | 61622227 | C | T | 0.329968 | -0.0122309 | 0.0018404 | 3.02134E-11 | 0.003 | 0.0099 | 0.764901 | 0.000131245 | 44.16617614 |
| rs17472816 | 10 | 12020794 | G | A | 0.056397 | -0.0230217 | 0.00380064 | 1.38538E-09 | 0.009 | 0.0203 | 0.6574 | 0.000109034 | 36.69093081 |
| rs17516020 | 10 | 79538734 | A | G | 0.269568 | -0.0152622 | 0.00196918 | 9.17698E-15 | -0.005 | 0.0106 | 0.634699 | 0.000178498 | 60.07044744 |
| rs17647041 | 18 | 33097189 | A | G | 0.194783 | 0.0145852 | 0.00219247 | 2.88802E-11 | -0.0083 | 0.0118 | 0.4793 | 0.000131507 | 44.254239 |
| rs17647719 | 15 | 51568204 | G | A | 0.0422392 | -0.0434982 | 0.00429268 | 3.971E-24 | -0.0008 | 0.023 | 0.9737 | 0.000305071 | 102.6793144 |
| rs17673793 | 16 | 75205213 | G | A | 0.313751 | -0.0129019 | 0.00186133 | 4.17061E-12 | -0.0154 | 0.01 | 0.1232 | 0.000142773 | 48.04608521 |
| rs17690703 | 17 | 43925297 | T | C | 0.264575 | -0.0128553 | 0.00194967 | 4.29932E-11 | -0.0047 | 0.0105 | 0.6526 | 0.000129192 | 43.47500418 |
| rs17741562 | 2 | 148563066 | C | T | 0.199792 | 0.0124539 | 0.00216041 | 8.19219E-09 | -0.0091 | 0.0116 | 0.4318 | 9.87516E-05 | 33.23041838 |
| rs17743415 | 2 | 71562503 | C | T | 0.566799 | -0.0252081 | 0.00174316 | 2.20191E-47 | -0.0066 | 0.0094 | 0.4801 | 0.000621133 | 209.1236456 |
| rs17855988 | 7 | 73474825 | C | G | 0.0985486 | 0.0187365 | 0.00293494 | 1.72818E-10 | -0.0201 | 0.0159 | 0.2052 | 0.000121108 | 40.75453093 |
| rs1788161 | 8 | 100696695 | T | A | 0.81925 | 0.0139683 | 0.00226943 | 7.5164E-10 | -0.0049 | 0.0122 | 0.686099 | 0.000112578 | 37.8835739 |
| rs1797623 | 3 | 112826681 | T | C | 0.366167 | 0.0130113 | 0.00180367 | 5.45381E-13 | 0.0043 | 0.0097 | 0.6544 | 0.000154635 | 52.03848397 |
| rs183743021 | 2 | 97283676 | C | T | 0.0245478 | -0.058851 | 0.00577673 | 2.27091E-24 | -0.0422 | 0.031 | 0.1732 | 0.00030836 | 103.7865255 |
| rs183761758 | 2 | 5832634 | A | C | 0.0987725 | -0.0165509 | 0.00289398 | 1.07199E-08 | -0.0038 | 0.0156 | 0.8088 | 9.71983E-05 | 32.70766923 |
| rs1862901 | 2 | 23886149 | C | T | 0.689522 | 0.0219138 | 0.00187442 | 1.43747E-31 | -0.0023 | 0.0101 | 0.8219 | 0.000406045 | 136.678117 |
| rs1884897 | 20 | 6612832 | G | A | 0.628254 | -0.0371123 | 0.00178932 | 1.69746E-95 | -0.0195 | 0.0096 | 0.0432803 | 0.001276889 | 430.1868457 |
| rs1886686 | 1 | 67390468 | G | C | 0.734725 | 0.0129973 | 0.00195376 | 2.88735E-11 | 0.0187 | 0.0105 | 0.0749894 | 0.000131509 | 44.2548912 |
| rs1910252 | 8 | 49407362 | T | C | 0.164083 | 0.0169182 | 0.00233113 | 3.95094E-13 | 0.0018 | 0.0125 | 0.8831 | 0.000156515 | 52.67114277 |
| rs1910466 | 3 | 147086268 | C | T | 0.500347 | -0.013096 | 0.00173662 | 4.67197E-14 | 0.0038 | 0.0093 | 0.6805 | 0.000168983 | 56.86763336 |
| rs1921129 | 12 | 107506589 | G | C | 0.59015 | -0.0122442 | 0.00175886 | 3.37443E-12 | 0.0109 | 0.0095 | 0.2486 | 0.000144007 | 48.46137181 |
| rs1936792 | 6 | 127423055 | A | G | 0.247403 | 0.0135747 | 0.00200855 | 1.39701E-11 | 0.009 | 0.0108 | 0.4025 | 0.000135733 | 45.67647754 |
| rs1948047 | 6 | 47585912 | C | T | 0.276817 | 0.0143506 | 0.00192855 | 1.00046E-13 | 0.0245 | 0.0104 | 0.0178801 | 0.000164534 | 55.37015399 |
| rs1952256 | 1 | 184035116 | G | A | 0.34736 | 0.0338351 | 0.00181213 | 9.25763E-78 | -0.0167 | 0.0098 | 0.0859805 | 0.001035035 | 348.6210823 |
| rs1990656 | 5 | 171220510 | A | T | 0.429153 | -0.0178195 | 0.00174709 | 2.00863E-24 | -0.0069 | 0.0094 | 0.4615 | 0.000309083 | 104.0298304 |
| rs2005053 | 13 | 47152942 | C | T | 0.600334 | 0.00990264 | 0.00175728 | 1.75001E-08 | -0.0255 | 0.0095 | 0.00702199 | 9.43686E-05 | 31.75539126 |
| rs2035901 | 4 | 145521867 | G | A | 0.465477 | 0.0248043 | 0.00173266 | 1.80136E-46 | -0.0015 | 0.0093 | 0.8711 | 0.000608711 | 204.9390272 |
| rs2088483 | 4 | 106216367 | C | T | 0.392998 | -0.0264502 | 0.00177037 | 1.86466E-50 | -0.0065 | 0.0095 | 0.495 | 0.000662965 | 223.2169943 |
| rs2117149 | 8 | 142324925 | C | T | 0.519155 | -0.0107544 | 0.00173132 | 5.24916E-10 | -0.0055 | 0.0093 | 0.5532 | 0.000114661 | 38.58469733 |
| rs2122858 | 15 | 85547816 | G | A | 0.607032 | -0.0132314 | 0.00176633 | 6.85804E-14 | 0.0104 | 0.0095 | 0.2757 | 0.000166742 | 56.11323742 |
| rs2131371 | 12 | 46796522 | C | A | 0.700138 | 0.0146894 | 0.00188394 | 6.35038E-15 | -0.0002 | 0.0101 | 0.9852 | 0.000180653 | 60.79549492 |
| rs2137777 | 11 | 18009721 | T | C | 0.542245 | 0.0123761 | 0.00173221 | 9.03649E-13 | -0.021 | 0.0093 | 0.0240099 | 0.000151687 | 51.04626319 |
| rs2139028 | 2 | 233163140 | T | G | 0.984052 | -0.0410735 | 0.00716964 | 1.01228E-08 | -0.0316 | 0.038 | 0.406 | 9.75292E-05 | 32.81906042 |
| rs2140046 | 2 | 169706079 | C | T | 0.363523 | -0.0210636 | 0.00179367 | 7.75532E-32 | 0.0134 | 0.0096 | 0.1629 | 0.000409686 | 137.9042109 |
| rs215226 | 12 | 591300 | G | A | 0.402219 | 0.0211865 | 0.00176709 | 4.09543E-33 | -0.0048 | 0.0095 | 0.614899 | 0.000427036 | 143.746909 |
| rs2181834 | 10 | 102661251 | T | G | 0.550242 | 0.0170463 | 0.00173475 | 8.73172E-23 | 0.002 | 0.0093 | 0.8301 | 0.000286887 | 96.55702603 |
| rs2194411 | 3 | 185548663 | A | G | 0.127999 | 0.0454192 | 0.00261267 | 1.16439E-67 | 0.0169 | 0.014 | 0.2281 | 0.000897364 | 302.2089085 |
| rs2230033 | 21 | 39671476 | A | G | 0.565337 | -0.0204177 | 0.00173963 | 8.37336E-32 | 0.0086 | 0.0094 | 0.3581 | 0.000409233 | 137.7517987 |
| rs224313 | 10 | 64616940 | C | T | 0.347798 | 0.0100906 | 0.00181372 | 2.64588E-08 | -0.0075 | 0.0098 | 0.4434 | 9.19818E-05 | 30.95215674 |
| rs2270894 | 3 | 9975386 | G | C | 0.205074 | -0.0290478 | 0.00221956 | 3.98658E-39 | 0.0008 | 0.0119 | 0.9451 | 0.000508768 | 171.2734835 |
| rs227745 | 17 | 54762165 | A | T | 0.272407 | 0.0205129 | 0.00193664 | 3.27567E-26 | 0.0362 | 0.0104 | 0.000517798 | 0.000333319 | 112.1898969 |
| rs2278093 | 12 | 29534209 | A | C | 0.284319 | 0.0170633 | 0.00192156 | 6.72512E-19 | -0.0028 | 0.0103 | 0.784701 | 0.000234296 | 78.85251905 |
| rs2282580 | 11 | 126081403 | C | T | 0.309107 | -0.0104701 | 0.00186545 | 1.99434E-08 | -0.0002 | 0.01 | 0.982 | 9.36143E-05 | 31.50154481 |
| rs2289635 | 1 | 172208620 | A | G | 0.212318 | 0.0366989 | 0.00210918 | 8.89201E-68 | 0.0358 | 0.0113 | 0.00156498 | 0.000898952 | 302.7444732 |
| rs2290154 | 18 | 77211175 | C | T | 0.357129 | -0.0147957 | 0.00180461 | 2.43613E-16 | 0.0016 | 0.0097 | 0.8655 | 0.00019974 | 67.22049916 |
| rs229624 | 14 | 65197767 | A | G | 0.194379 | 0.0123043 | 0.00218196 | 1.71057E-08 | 0.0166 | 0.0117 | 0.1574 | 9.44991E-05 | 31.79930819 |
| rs2296316 | 14 | 65520246 | C | T | 0.464337 | -0.0205133 | 0.00174294 | 5.69639E-32 | 0.0249 | 0.0094 | 0.00789005 | 0.000411506 | 138.5171692 |
| rs2303597 | 2 | 71780821 | C | T | 0.707283 | -0.0135824 | 0.00190741 | 1.07473E-12 | 0.0244 | 0.0102 | 0.01714 | 0.000150677 | 50.70634893 |
| rs2305769 | 19 | 17264961 | C | G | 0.231393 | -0.0198139 | 0.0020669 | 9.18967E-22 | -0.0068 | 0.0111 | 0.54 | 0.000273043 | 91.89638375 |
| rs233598 | 21 | 28330838 | G | T | 0.773971 | 0.0124006 | 0.00207899 | 2.45256E-09 | 0.0022 | 0.0112 | 0.8458 | 0.000105726 | 35.57771203 |
| rs2342082 | 12 | 123090380 | C | T | 0.740297 | 0.0191217 | 0.00197413 | 3.47616E-22 | 0.0295 | 0.0106 | 0.00534897 | 0.000278759 | 93.82075476 |
| rs2417860 | 12 | 20827579 | G | A | 0.633009 | -0.0172528 | 0.00179302 | 6.48485E-22 | -0.0178 | 0.0096 | 0.0649995 | 0.000275092 | 92.58621423 |
| rs2434576 | 5 | 138917674 | G | A | 0.290907 | 0.0180161 | 0.00190455 | 3.11315E-21 | -0.0104 | 0.0102 | 0.3082 | 0.00026587 | 89.4817005 |
| rs244711 | 5 | 176509193 | T | C | 0.687357 | 0.039336 | 0.00199264 | 1.08168E-86 | 0.0174 | 0.0106 | 0.102 | 0.001156827 | 389.6907714 |
| rs2451948 | 9 | 109518208 | G | A | 0.157898 | 0.0194331 | 0.00238245 | 3.45303E-16 | -0.0086 | 0.0128 | 0.4992 | 0.000197697 | 66.53252405 |
| rs2456725 | 10 | 121652073 | C | T | 0.600755 | -0.0106357 | 0.0017604 | 1.52704E-09 | 0.0074 | 0.0095 | 0.4354 | 0.00010847 | 36.50117595 |
| rs246185 | 16 | 14395432 | C | T | 0.321129 | 0.0178947 | 0.00186351 | 7.83791E-22 | 0.0079 | 0.01 | 0.4279 | 0.000273977 | 92.21101601 |
| rs2513299 | 11 | 68410157 | T | C | 0.844524 | 0.025297 | 0.00240007 | 5.69639E-26 | 0.001 | 0.0129 | 0.9367 | 0.000330062 | 111.0932426 |
| rs2531986 | 16 | 4029577 | C | T | 0.812517 | 0.0158205 | 0.00220838 | 7.8614E-13 | 0.0128 | 0.0119 | 0.2793 | 0.000152502 | 51.3204219 |
| rs2581828 | 3 | 53133149 | G | C | 0.589653 | -0.0244886 | 0.00175082 | 1.92886E-44 | 0.015 | 0.0094 | 0.1122 | 0.000581087 | 195.6330994 |
| rs2610986 | 4 | 18037231 | T | C | 0.65789 | -0.0231712 | 0.00186369 | 1.76198E-35 | 0.0203 | 0.01 | 0.0419295 | 0.000459197 | 154.5777994 |
| rs2631676 | 10 | 93037409 | G | A | 0.190337 | 0.0243485 | 0.00219717 | 1.55597E-28 | -0.0138 | 0.0118 | 0.2434 | 0.000364844 | 122.8045676 |
| rs2650965 | 20 | 6709838 | G | A | 0.328146 | -0.0180808 | 0.00184274 | 1.00716E-22 | 0.0014 | 0.0099 | 0.8874 | 0.000286043 | 96.27301589 |
| rs2655225 | 3 | 13551420 | A | G | 0.877676 | -0.0258117 | 0.00264411 | 1.65044E-22 | -0.0234 | 0.0142 | 0.1001 | 0.000283139 | 95.29532573 |
| rs2690034 | 1 | 200143844 | G | C | 0.697092 | 0.0107438 | 0.00190096 | 1.58921E-08 | -0.0075 | 0.0102 | 0.4657 | 9.49242E-05 | 31.94238146 |
| rs2715553 | 17 | 38496320 | A | G | 0.541072 | 0.0123367 | 0.00173454 | 1.14288E-12 | 0.0092 | 0.0093 | 0.325 | 0.000150319 | 50.58558621 |
| rs2737215 | 8 | 116625707 | T | C | 0.560122 | -0.0123733 | 0.00174972 | 1.53426E-12 | -0.0285 | 0.0094 | 0.00245098 | 0.0001486 | 50.00706804 |
| rs273974 | 7 | 137614713 | T | C | 0.483525 | 0.011133 | 0.00172718 | 1.15178E-10 | 0.0099 | 0.0093 | 0.2859 | 0.000123465 | 41.5476664 |
| rs2746026 | 17 | 18134354 | T | C | 0.40321 | 0.0134811 | 0.00176077 | 1.91779E-14 | -0.0155 | 0.0095 | 0.102 | 0.000174188 | 58.6195957 |
| rs274715 | 5 | 6723429 | G | A | 0.671308 | -0.012159 | 0.0018345 | 3.408E-11 | -0.0237 | 0.0098 | 0.0161998 | 0.000130543 | 43.92964597 |
| rs2763263 | 6 | 168814392 | A | T | 0.243119 | -0.0253872 | 0.00202547 | 4.95564E-36 | -0.0106 | 0.0109 | 0.3303 | 0.000466685 | 157.099715 |
| rs2780226 | 6 | 34199092 | T | C | 0.911435 | -0.0672272 | 0.00303185 | 7.3961E-109 | -0.0326 | 0.0163 | 0.0449004 | 0.001459113 | 491.6681134 |
| rs2802346 | 1 | 26434354 | A | G | 0.176233 | 0.0205174 | 0.00226326 | 1.24767E-19 | -0.0025 | 0.0122 | 0.8385 | 0.000244185 | 82.18133151 |
| rs2812237 | 13 | 51299120 | A | G | 0.160395 | -0.0185642 | 0.00235622 | 3.31589E-15 | -0.0208 | 0.0126 | 0.0996506 | 0.000184454 | 62.07521318 |
| rs28410896 | 12 | 123901143 | C | T | 0.806748 | -0.0278336 | 0.00219204 | 6.21584E-37 | 0.0606 | 0.0118 | 2.66901E-07 | 0.000478941 | 161.2275417 |
| rs28470731 | 4 | 7848144 | T | C | 0.515375 | 0.0142751 | 0.00174037 | 2.36592E-16 | -0.028 | 0.0093 | 0.00272402 | 0.000199911 | 67.27792063 |
| rs2851023 | 4 | 100439152 | T | C | 0.675758 | -0.0129257 | 0.001843 | 2.33024E-12 | -0.0032 | 0.0099 | 0.743401 | 0.000146165 | 49.18751611 |
| rs28519617 | 3 | 135874930 | G | T | 0.271111 | -0.0232026 | 0.00195417 | 1.65158E-32 | 0.0253 | 0.0105 | 0.0161801 | 0.000418808 | 140.9762783 |
| rs2853993 | 6 | 31333191 | C | T | 0.590281 | -0.0291966 | 0.00175259 | 2.75486E-62 | -0.0214 | 0.0094 | 0.0231201 | 0.000824127 | 277.5244942 |
| rs2871960 | 3 | 141121814 | C | A | 0.447942 | 0.0592759 | 0.00173199 | 1E-200 | -0.0134 | 0.0093 | 0.151 | 0.003469004 | 1171.286052 |
| rs2885697 | 1 | 41544279 | T | G | 0.664376 | -0.0296652 | 0.00182658 | 2.73338E-59 | 0.0014 | 0.0098 | 0.8832 | 0.000783295 | 263.7634312 |
| rs28880687 | 15 | 41437593 | C | T | 0.372602 | -0.0152749 | 0.00178593 | 1.20504E-17 | 0.0013 | 0.0096 | 0.8927 | 0.000217361 | 73.15184848 |
| rs2888604 | 12 | 102343853 | T | C | 0.445861 | 0.0177449 | 0.0017414 | 2.21615E-24 | 0.0111 | 0.0094 | 0.2349 | 0.000308507 | 103.8358807 |
| rs2890718 | 9 | 35932874 | A | G | 0.489313 | -0.0130555 | 0.00174545 | 7.46449E-14 | -0.0154 | 0.0094 | 0.1002 | 0.000166245 | 55.94607351 |
| rs28929474 | 14 | 94844947 | T | C | 0.0204301 | 0.113956 | 0.00609701 | 6.49232E-78 | -0.1249 | 0.0331 | 0.000163399 | 0.001037142 | 349.3316997 |
| rs2900208 | 12 | 11878464 | A | C | 0.355387 | 0.0215292 | 0.00180574 | 9.1601E-33 | 0.0228 | 0.0097 | 0.01883 | 0.00042229 | 142.1486587 |
| rs2916261 | 2 | 47287158 | G | A | 0.787611 | -0.011999 | 0.00211255 | 1.34896E-08 | 0.0147 | 0.0113 | 0.1935 | 9.58701E-05 | 32.26068108 |
| rs291970 | 10 | 121123633 | T | C | 0.229508 | 0.0196334 | 0.00205097 | 1.04761E-21 | 0.0236 | 0.011 | 0.03211 | 0.000272272 | 91.63678254 |
| rs2925155 | 8 | 75886297 | T | C | 0.260513 | -0.0194114 | 0.00198087 | 1.13999E-22 | -0.0173 | 0.0106 | 0.1041 | 0.000285316 | 96.02828816 |
| rs2967363 | 16 | 82204132 | G | C | 0.770916 | -0.0186357 | 0.00205468 | 1.19757E-19 | -0.0073 | 0.011 | 0.5091 | 0.000244425 | 82.26222508 |
| rs2974427 | 5 | 168261021 | C | T | 0.405169 | -0.0138677 | 0.00176326 | 3.70595E-15 | -0.005 | 0.0095 | 0.5989 | 0.0001838 | 61.85482513 |
| rs2985313 | 1 | 21084999 | T | C | 0.291948 | -0.0186095 | 0.00193561 | 7.0081E-22 | -0.0123 | 0.0104 | 0.2355 | 0.000274639 | 92.43386515 |
| rs3003137 | 20 | 62830802 | T | G | 0.42625 | -0.0101273 | 0.00175903 | 8.55539E-09 | 0.007 | 0.0094 | 0.4579 | 9.85023E-05 | 33.14654573 |
| rs300982 | 3 | 87325753 | A | G | 0.0489815 | -0.0251723 | 0.00398557 | 2.68999E-10 | 0.0355 | 0.0214 | 0.0967498 | 0.000118539 | 39.88984365 |
| rs3011897 | 6 | 155573834 | C | T | 0.442085 | -0.0132094 | 0.00173764 | 2.9255E-14 | 0.0058 | 0.0093 | 0.5357 | 0.00017172 | 57.78884184 |
| rs3020407 | 6 | 152307261 | A | G | 0.681179 | -0.0137781 | 0.0018506 | 9.70287E-14 | 0.0013 | 0.0099 | 0.8984 | 0.000164714 | 55.43077719 |
| rs3020619 | 17 | 61993137 | G | A | 0.271528 | 0.039056 | 0.00195284 | 6.25605E-89 | 0.02 | 0.0105 | 0.05611 | 0.001187339 | 399.9812363 |
| rs303084 | 4 | 124066948 | A | G | 0.796617 | -0.0150542 | 0.00215248 | 2.67855E-12 | -0.0199 | 0.0116 | 0.0843607 | 0.000145352 | 48.91415234 |
| rs3094626 | 6 | 30323623 | C | T | 0.403263 | 0.0158081 | 0.00175661 | 2.28507E-19 | 0.0185 | 0.0094 | 0.0497096 | 0.000240632 | 80.9852759 |
| rs310792 | 12 | 77450413 | T | C | 0.702091 | 0.0164414 | 0.00190066 | 5.15466E-18 | -0.0066 | 0.0102 | 0.5204 | 0.000222342 | 74.8283449 |
| rs3116200 | 2 | 233073182 | A | G | 0.0183586 | -0.070308 | 0.00642264 | 6.95184E-28 | -0.0022 | 0.0344 | 0.9494 | 0.000356021 | 119.8339148 |
| rs3118906 | 13 | 51106788 | A | G | 0.277922 | -0.0414089 | 0.00192262 | 8.1096E-103 | -0.0106 | 0.0103 | 0.3051 | 0.001376736 | 463.8717183 |
| rs3213332 | 19 | 44062687 | A | G | 0.189209 | 0.0164189 | 0.00220515 | 9.66496E-14 | 0.0092 | 0.0118 | 0.4377 | 0.000164736 | 55.43821786 |
| rs3218036 | 19 | 30305684 | A | G | 0.326917 | 0.0107145 | 0.00184326 | 6.14979E-09 | 0.0074 | 0.0099 | 0.456 | 0.00010041 | 33.7884321 |
| rs332194 | 10 | 28922228 | C | T | 0.798703 | -0.0161863 | 0.00215809 | 6.38263E-14 | -0.0007 | 0.0116 | 0.9501 | 0.00016716 | 56.25400983 |
| rs34118426 | 12 | 94201279 | G | A | 0.304915 | -0.0209403 | 0.0018772 | 6.84069E-29 | 0.0377 | 0.0101 | 0.000188101 | 0.000369686 | 124.4348779 |
| rs34517439 | 1 | 78450517 | A | C | 0.125765 | 0.0337435 | 0.00264526 | 2.9363E-37 | 0.0459 | 0.0143 | 0.00128201 | 0.000483373 | 162.7200029 |
| rs34642857 | 3 | 123051019 | C | T | 0.253279 | -0.0145584 | 0.00199692 | 3.09671E-13 | 0.0113 | 0.0107 | 0.293 | 0.000157938 | 53.15001368 |
| rs34647936 | 19 | 19548239 | G | T | 0.186507 | -0.0239267 | 0.00221708 | 3.79402E-27 | -0.0061 | 0.0119 | 0.6081 | 0.00034602 | 116.4663054 |
| rs34776209 | 7 | 23513093 | T | C | 0.248293 | -0.031181 | 0.00199968 | 8.49767E-55 | 0.0265 | 0.0108 | 0.0136499 | 0.000722094 | 243.1400441 |
| rs34919557 | 20 | 32510282 | T | C | 0.0923713 | -0.0198889 | 0.00298964 | 2.88337E-11 | -0.0051 | 0.0161 | 0.7519 | 0.000131515 | 44.25691687 |
| rs35307904 | 9 | 78511889 | A | G | 0.122158 | -0.0432015 | 0.00264818 | 8.32722E-60 | 0.0083 | 0.0142 | 0.5612 | 0.000790329 | 266.1338204 |
| rs35467921 | 16 | 30048553 | T | C | 0.401665 | 0.0169061 | 0.00175937 | 7.35868E-22 | 0.0231 | 0.0095 | 0.0148898 | 0.000274348 | 92.33575847 |
| rs35485741 | 1 | 151241909 | C | G | 0.11211 | -0.0197797 | 0.00273968 | 5.22035E-13 | 0.0061 | 0.0147 | 0.6773 | 0.000154889 | 52.12392852 |
| rs35539500 | 2 | 121613995 | G | T | 0.168621 | -0.0231454 | 0.00230459 | 9.9243E-24 | -0.0123 | 0.0124 | 0.3189 | 0.000299682 | 100.8647587 |
| rs35628589 | 20 | 61440005 | T | C | 0.0704586 | 0.0343206 | 0.00349224 | 8.61787E-23 | 0.0337 | 0.0188 | 0.0731897 | 0.000286963 | 96.58262327 |
| rs35629473 | 6 | 169465291 | G | C | 0.166257 | 0.0167646 | 0.00236199 | 1.27145E-12 | -0.007 | 0.0127 | 0.5805 | 0.000149697 | 50.37646636 |
| rs35713889 | 3 | 49162583 | T | C | 0.0447258 | 0.0426216 | 0.0041745 | 1.80385E-24 | 0.0457 | 0.0225 | 0.0423799 | 0.000309717 | 104.2432603 |
| rs35873563 | 9 | 139324029 | T | C | 0.286106 | 0.0198542 | 0.001906 | 2.09942E-25 | 0.0032 | 0.0103 | 0.7576 | 0.00032238 | 108.5066638 |
| rs35874463 | 15 | 67457698 | G | A | 0.057733 | 0.0638607 | 0.00369111 | 4.92266E-67 | 0.0295 | 0.02 | 0.141 | 0.000888824 | 299.3303766 |
| rs35897527 | 1 | 173937885 | A | G | 0.0802938 | 0.0184949 | 0.0032075 | 8.1182E-09 | -0.011 | 0.0172 | 0.5231 | 9.88044E-05 | 33.24819426 |
| rs35943760 | 2 | 47011755 | T | A | 0.470421 | 0.0139377 | 0.00173675 | 1.01742E-15 | 0.0238 | 0.0093 | 0.01065 | 0.00019137 | 64.40284136 |
| rs35954730 | 10 | 12943111 | A | G | 0.288871 | -0.0228305 | 0.00191016 | 6.42984E-33 | 0.003 | 0.0103 | 0.7722 | 0.000424381 | 142.8528047 |
| rs35990522 | 9 | 119274481 | A | T | 0.0753294 | 0.0256022 | 0.00327254 | 5.15822E-15 | 0.0065 | 0.0176 | 0.709601 | 0.000181867 | 61.20431802 |
| rs36101807 | 5 | 95549286 | T | G | 0.307108 | 0.0132275 | 0.00186925 | 1.48252E-12 | 0.0239 | 0.01 | 0.01726 | 0.000148801 | 50.07467973 |
| rs36226649 | 14 | 24835500 | C | T | 0.0673934 | 0.0360997 | 0.00344248 | 1.00531E-25 | 0.0066 | 0.0185 | 0.721601 | 0.000326716 | 109.9668418 |
| rs3732858 | 3 | 43097765 | A | G | 0.167653 | -0.0167204 | 0.00230836 | 4.38531E-13 | -0.0161 | 0.0124 | 0.1954 | 0.000155908 | 52.4666897 |
| rs3751695 | 16 | 89764549 | A | G | 0.169827 | 0.0147712 | 0.00231009 | 1.61603E-10 | -0.0215 | 0.0124 | 0.0840698 | 0.000121498 | 40.88567529 |
| rs3761181 | 20 | 48895112 | G | A | 0.534954 | 0.0115601 | 0.00173682 | 2.81968E-11 | -0.0235 | 0.0093 | 0.01185 | 0.000131645 | 44.30073966 |
| rs3789280 | 9 | 118953372 | A | T | 0.359315 | 0.0134944 | 0.00180384 | 7.39776E-14 | 0.0105 | 0.0097 | 0.2805 | 0.000166298 | 55.96397462 |
| rs3796515 | 4 | 39335475 | A | G | 0.526639 | 0.0132659 | 0.00172738 | 1.59809E-14 | -0.004 | 0.0093 | 0.6656 | 0.000175255 | 58.97868471 |
| rs3798417 | 6 | 116721371 | T | C | 0.732922 | 0.0155436 | 0.00195207 | 1.68927E-15 | 0.0044 | 0.0105 | 0.674401 | 0.000188399 | 63.40300873 |
| rs3802342 | 9 | 133473303 | G | C | 0.353294 | -0.0235696 | 0.00180609 | 6.48784E-39 | 0.0105 | 0.0097 | 0.2798 | 0.000505888 | 170.3033013 |
| rs3812163 | 6 | 7725760 | T | A | 0.470148 | 0.033883 | 0.00173082 | 2.75233E-85 | -0.0375 | 0.0093 | 5.64495E-05 | 0.001137664 | 383.2280773 |
| rs3816562 | 2 | 219206919 | T | C | 0.521063 | 0.0160033 | 0.00173327 | 2.64545E-20 | 0.0024 | 0.0093 | 0.7938 | 0.000253294 | 85.24797501 |
| rs3828559 | 4 | 184211030 | G | A | 0.75833 | 0.017618 | 0.00201532 | 2.29985E-18 | -0.0032 | 0.0108 | 0.7671 | 0.000227078 | 76.42273924 |
| rs3914294 | 3 | 99294991 | T | C | 0.63447 | -0.016503 | 0.00179595 | 3.98566E-20 | 0.0158 | 0.0097 | 0.1028 | 0.000250886 | 84.4373772 |
| rs3983746 | 9 | 103877068 | G | A | 0.232877 | -0.0126918 | 0.00205004 | 5.98467E-10 | 0.0125 | 0.011 | 0.2552 | 0.000113899 | 38.32826743 |
| rs400040 | 2 | 199422 | T | G | 0.869825 | 0.0167079 | 0.0025642 | 7.23936E-11 | -0.0131 | 0.0138 | 0.3435 | 0.000126163 | 42.45583252 |
| rs41177 | 22 | 30438447 | A | G | 0.363357 | 0.0122417 | 0.00179329 | 8.72369E-12 | 0.0221 | 0.0096 | 0.0219402 | 0.000138475 | 46.59934687 |
| rs41271299 | 6 | 19839415 | T | C | 0.0517069 | 0.0944273 | 0.00389694 | 1.3521E-129 | 0.0001 | 0.021 | 0.9966 | 0.001741963 | 587.1446717 |
| rs41271357 | 3 | 113004093 | A | G | 0.205539 | -0.0125323 | 0.00213625 | 4.45502E-09 | -0.0034 | 0.0115 | 0.763999 | 0.000102273 | 34.41555933 |
| rs41369549 | 11 | 9462534 | C | G | 0.426717 | 0.0103102 | 0.00174699 | 3.60139E-09 | 0.0011 | 0.0094 | 0.9056 | 0.000103504 | 34.82978233 |
| rs4151450 | 13 | 48918174 | G | C | 0.72186 | -0.0113237 | 0.00192675 | 4.17927E-09 | 0.0054 | 0.0104 | 0.6011 | 0.000102643 | 34.54008431 |
| rs42044 | 7 | 92250140 | G | T | 0.270004 | 0.0443174 | 0.00194358 | 5.3703E-115 | 0.0165 | 0.0104 | 0.113 | 0.001542843 | 519.9255053 |
| rs4235816 | 6 | 83733785 | T | C | 0.683854 | -0.0118463 | 0.00185619 | 1.74977E-10 | -0.0035 | 0.01 | 0.727999 | 0.000121037 | 40.73033805 |
| rs4245596 | 10 | 63725862 | A | G | 0.572619 | -0.0113724 | 0.00174756 | 7.64716E-11 | -0.0166 | 0.0094 | 0.0774408 | 0.000125844 | 42.34844624 |
| rs4252548 | 19 | 55879672 | T | C | 0.0213598 | -0.0698462 | 0.00596318 | 1.11045E-31 | 0.0512 | 0.0319 | 0.1088 | 0.000407569 | 137.1914819 |
| rs4258054 | 9 | 4297892 | C | T | 0.280586 | -0.0110748 | 0.00194248 | 1.18962E-08 | 0.0157 | 0.0104 | 0.1312 | 9.65973E-05 | 32.50544311 |
| rs4287972 | 4 | 88645798 | T | C | 0.501492 | -0.0147702 | 0.00172567 | 1.14183E-17 | -0.0171 | 0.0093 | 0.0659903 | 0.000217676 | 73.25793496 |
| rs4291276 | 8 | 23173591 | G | A | 0.763958 | -0.021542 | 0.00203442 | 3.39234E-26 | -0.0277 | 0.011 | 0.01149 | 0.000333115 | 112.1213268 |
| rs4316494 | 11 | 12664541 | G | A | 0.490111 | 0.0176958 | 0.00173551 | 2.07635E-24 | -0.0306 | 0.0093 | 0.001034 | 0.000308888 | 103.9641435 |
| rs4318495 | 12 | 14483481 | C | T | 0.552812 | -0.0154747 | 0.00173453 | 4.62062E-19 | 0.0193 | 0.0093 | 0.0388204 | 0.000236498 | 79.59362119 |
| rs439205 | 6 | 33173842 | A | G | 0.242603 | -0.0112237 | 0.00201112 | 2.3953E-08 | -0.04 | 0.0108 | 0.000218902 | 9.2556E-05 | 31.14537386 |
| rs4401680 | 6 | 140164286 | G | T | 0.784414 | 0.012487 | 0.00209507 | 2.52156E-09 | -0.0051 | 0.0112 | 0.6496 | 0.000105565 | 35.52356684 |
| rs440292 | 5 | 67609408 | G | C | 0.74501 | 0.0157095 | 0.00197948 | 2.0917E-15 | 0.017 | 0.0106 | 0.1092 | 0.00018715 | 62.98250175 |
| rs4417735 | 2 | 106762787 | C | T | 0.127544 | 0.0159217 | 0.00260811 | 1.03117E-09 | 0.0074 | 0.014 | 0.5988 | 0.000110746 | 37.26700427 |
| rs4421120 | 5 | 32763118 | A | G | 0.161362 | 0.0276617 | 0.00234629 | 4.48539E-32 | -0.0085 | 0.0126 | 0.4999 | 0.000412918 | 138.9926302 |
| rs4466161 | 5 | 122766856 | C | T | 0.232344 | -0.0147259 | 0.00205173 | 7.12361E-13 | 0.0214 | 0.011 | 0.0514304 | 0.000153075 | 51.51345692 |
| rs45446698 | 7 | 99332948 | G | T | 0.0427848 | 0.0389883 | 0.00426097 | 5.71874E-20 | -0.0235 | 0.0233 | 0.3138 | 0.000248767 | 83.72382549 |
| rs45528934 | 14 | 23793305 | T | C | 0.163779 | 0.0224422 | 0.00233144 | 6.25605E-22 | -0.0183 | 0.0126 | 0.1454 | 0.000275303 | 92.65733155 |
| rs459193 | 5 | 55806751 | G | A | 0.746842 | -0.011209 | 0.00197974 | 1.49886E-08 | 0.0185 | 0.0107 | 0.0832799 | 9.52631E-05 | 32.05640682 |
| rs4621412 | 4 | 8605338 | A | G | 0.402443 | -0.0168452 | 0.00176604 | 1.45949E-21 | 0.0066 | 0.0095 | 0.4868 | 0.000270323 | 90.98057767 |
| rs4700662 | 5 | 64392906 | T | C | 0.666172 | 0.0131555 | 0.00183675 | 7.94511E-13 | 0.0111 | 0.0099 | 0.2606 | 0.000152439 | 51.29937091 |
| rs472659 | 6 | 18999707 | T | C | 0.111108 | 0.0155852 | 0.00274241 | 1.32434E-08 | -0.0189 | 0.0148 | 0.2013 | 9.59769E-05 | 32.29664504 |
| rs4735766 | 8 | 78099782 | T | G | 0.285453 | 0.0314322 | 0.00191046 | 8.47813E-61 | 0.028 | 0.0103 | 0.00651193 | 0.000803846 | 270.6892935 |
| rs4761529 | 12 | 94207194 | G | A | 0.74091 | -0.0112597 | 0.00197155 | 1.12336E-08 | 0.0034 | 0.0106 | 0.749601 | 9.69269E-05 | 32.61635807 |
| rs4767492 | 12 | 117436861 | T | C | 0.91297 | 0.0192056 | 0.00307484 | 4.21425E-10 | -0.0345 | 0.0165 | 0.0371099 | 0.000115933 | 39.01289091 |
| rs4768862 | 12 | 51194827 | A | G | 0.671624 | -0.0143863 | 0.0018361 | 4.69137E-15 | 0.0026 | 0.0099 | 0.792399 | 0.000182421 | 61.3907561 |
| rs4775769 | 15 | 48939888 | G | T | 0.907218 | 0.0280016 | 0.00298503 | 6.59478E-21 | -0.0037 | 0.016 | 0.8186 | 0.000261459 | 87.99656369 |
| rs4776470 | 15 | 69992329 | G | A | 0.345701 | -0.0170979 | 0.00181453 | 4.42079E-21 | -0.0071 | 0.0098 | 0.4656 | 0.00026381 | 88.78807868 |
| rs4788815 | 16 | 71634811 | T | A | 0.656375 | -0.0114869 | 0.00181768 | 2.62621E-10 | -0.015 | 0.0098 | 0.1242 | 0.000118677 | 39.93633823 |
| rs4788891 | 17 | 73391145 | G | A | 0.838232 | 0.0188894 | 0.00234292 | 7.50931E-16 | 0.0143 | 0.0125 | 0.2556 | 0.000193146 | 65.00081855 |
| rs481237 | 9 | 98316094 | A | G | 0.0858277 | -0.0560061 | 0.00315643 | 2.08497E-70 | -0.0145 | 0.017 | 0.3923 | 0.000934804 | 314.8297443 |
| rs4814656 | 20 | 17769540 | A | G | 0.437574 | -0.0116785 | 0.00172963 | 1.46016E-11 | 0.0033 | 0.0093 | 0.7227 | 0.000135474 | 45.58953157 |
| rs4834802 | 4 | 120614296 | C | G | 0.335267 | -0.0117385 | 0.00183417 | 1.55726E-10 | 0.0055 | 0.0099 | 0.5761 | 0.000121714 | 40.95844457 |
| rs4842133 | 9 | 139127566 | A | C | 0.278935 | 0.021417 | 0.00192529 | 9.69393E-29 | 0.0101 | 0.0104 | 0.3301 | 0.000367632 | 123.743496 |
| rs4868125 | 5 | 171281875 | G | C | 0.580137 | 0.0275398 | 0.00177748 | 3.99301E-54 | 0.0057 | 0.0095 | 0.551099 | 0.000712937 | 240.0543261 |
| rs4872482 | 8 | 22249171 | C | T | 0.464282 | 0.0100475 | 0.00173 | 6.33563E-09 | 0.0179 | 0.0093 | 0.0545406 | 0.000100237 | 33.73038063 |
| rs4885084 | 13 | 73851888 | C | T | 0.680518 | 0.0102754 | 0.00184964 | 2.77287E-08 | -0.0092 | 0.01 | 0.3551 | 9.17132E-05 | 30.86174475 |
| rs4899012 | 14 | 61003889 | C | G | 0.61037 | -0.0294862 | 0.00176999 | 2.76121E-62 | -0.0075 | 0.0095 | 0.4318 | 0.000824112 | 277.5194378 |
| rs4899401 | 14 | 37004549 | G | A | 0.555727 | -0.013774 | 0.00174118 | 2.56626E-15 | 0.003 | 0.0094 | 0.7466 | 0.000185952 | 62.57923299 |
| rs4901548 | 14 | 55274849 | G | C | 0.551238 | 0.0145138 | 0.00174153 | 7.84874E-17 | -0.0061 | 0.0094 | 0.512 | 0.000206376 | 69.45408061 |
| rs4906203 | 14 | 102928991 | T | C | 0.232822 | -0.0176328 | 0.00204156 | 5.79963E-18 | -0.0292 | 0.011 | 0.00772805 | 0.000221651 | 74.59602495 |
| rs4927132 | 1 | 54953404 | T | A | 0.100255 | -0.0236951 | 0.00289032 | 2.45132E-16 | -0.0093 | 0.0156 | 0.552 | 0.000199704 | 67.20826037 |
| rs494938 | 6 | 72195837 | A | G | 0.313208 | 0.0121147 | 0.00186273 | 7.84513E-11 | 0.012 | 0.01 | 0.2303 | 0.000125695 | 42.29830149 |
| rs4965600 | 15 | 100765385 | A | G | 0.61412 | -0.021476 | 0.00177715 | 1.29599E-33 | -0.019 | 0.0095 | 0.0469602 | 0.000433829 | 146.0346914 |
| rs4980067 | 10 | 81136129 | A | C | 0.50449 | -0.023203 | 0.0017264 | 3.60579E-41 | -0.0045 | 0.0093 | 0.630199 | 0.000536563 | 180.6353912 |
| rs4985445 | 16 | 69867835 | G | A | 0.458105 | -0.0185371 | 0.00173316 | 1.07845E-26 | 0.0526 | 0.0094 | 1.94402E-08 | 0.000339866 | 114.3941168 |
| rs4986172 | 17 | 43216281 | T | C | 0.340296 | -0.0212323 | 0.00182087 | 2.05731E-31 | 0.0327 | 0.0098 | 0.000839692 | 0.000403933 | 135.9670331 |
| rs498685 | 18 | 13088673 | C | T | 0.591455 | -0.0128034 | 0.00175444 | 2.93359E-13 | 0.025 | 0.0094 | 0.008179 | 0.000158254 | 53.25630184 |
| rs506154 | 7 | 28202079 | C | T | 0.704735 | -0.0373877 | 0.0018903 | 5.09448E-87 | -0.0252 | 0.0102 | 0.0132999 | 0.001161288 | 391.1951379 |
| rs519384 | 3 | 172168507 | A | T | 0.289156 | 0.0270486 | 0.00190986 | 1.60768E-45 | 0.0226 | 0.0103 | 0.0282202 | 0.000595767 | 200.5782951 |
| rs52826764 | 2 | 20205541 | T | C | 0.0296294 | -0.0619446 | 0.00508908 | 4.45143E-34 | -0.0213 | 0.0274 | 0.4377 | 0.000440135 | 148.1582257 |
| rs55652172 | 1 | 113147607 | A | G | 0.205368 | -0.0165544 | 0.00213359 | 8.58618E-15 | -0.0055 | 0.0115 | 0.6292 | 0.000178886 | 60.2008154 |
| rs55726184 | 14 | 24834971 | A | G | 0.11254 | -0.0185473 | 0.00284045 | 6.59781E-11 | 0.0175 | 0.0152 | 0.2512 | 0.000126701 | 42.63679134 |
| rs55749333 | 17 | 7371932 | T | C | 0.638267 | -0.026452 | 0.00179737 | 5.18681E-49 | -0.0025 | 0.0097 | 0.7947 | 0.000643296 | 216.5905327 |
| rs55778236 | 5 | 115022380 | C | T | 0.277222 | 0.0173869 | 0.00192747 | 1.88018E-19 | 0.0003 | 0.0104 | 0.974 | 0.000241776 | 81.37040484 |
| rs55852614 | 2 | 172416869 | C | T | 0.250674 | -0.0192525 | 0.00199134 | 4.14763E-22 | -0.0076 | 0.0107 | 0.4781 | 0.000277723 | 93.47185201 |
| rs55933602 | 3 | 50343600 | T | C | 0.104424 | -0.0251712 | 0.00283076 | 6.02837E-19 | 0.0012 | 0.0153 | 0.937 | 0.000234935 | 79.06770968 |
| rs56165045 | 21 | 30114027 | A | G | 0.137784 | -0.017408 | 0.00250434 | 3.63078E-12 | -0.0056 | 0.0134 | 0.6758 | 0.000143581 | 48.31796046 |
| rs56302937 | 11 | 2805911 | G | T | 0.0325312 | 0.0368419 | 0.00495413 | 1.03538E-13 | -0.0386 | 0.0266 | 0.147 | 0.000164334 | 55.3027414 |
| rs563537 | 11 | 118595207 | A | T | 0.398229 | 0.013947 | 0.00177001 | 3.29458E-15 | -0.0052 | 0.0095 | 0.581201 | 0.000184492 | 62.08798712 |
| rs56383911 | 1 | 2068714 | A | G | 0.236435 | 0.0164195 | 0.00204349 | 9.38858E-16 | -0.0195 | 0.011 | 0.0766796 | 0.00019184 | 64.56129618 |
| rs56400819 | 8 | 143925374 | G | A | 0.551897 | 0.0108075 | 0.0017362 | 4.82759E-10 | -0.0198 | 0.0093 | 0.0335398 | 0.000115146 | 38.74792078 |
| rs57176772 | 7 | 19632595 | A | G | 0.150247 | 0.0214954 | 0.00242843 | 8.67162E-19 | -0.0098 | 0.0131 | 0.4526 | 0.000232802 | 78.34969355 |
| rs5742915 | 15 | 74336633 | C | T | 0.461723 | 0.0237874 | 0.00173033 | 5.4275E-43 | -0.0065 | 0.0093 | 0.4848 | 0.000561359 | 188.9876804 |
| rs575050 | 11 | 85339542 | T | G | 0.496619 | 0.00984623 | 0.0017302 | 1.26558E-08 | 0.0051 | 0.0093 | 0.584901 | 9.62396E-05 | 32.38506372 |
| rs58087925 | 14 | 105983096 | T | C | 0.238109 | 0.0136789 | 0.00204474 | 2.23872E-11 | -0.0126 | 0.0115 | 0.2735 | 0.000132989 | 44.75314722 |
| rs58408429 | 4 | 57769824 | C | T | 0.184787 | 0.0244738 | 0.00222328 | 3.53834E-28 | 0.0222 | 0.012 | 0.0635302 | 0.000360003 | 121.1746878 |
| rs58416107 | 12 | 28696578 | A | G | 0.294759 | -0.0323109 | 0.00189599 | 4.29536E-65 | -0.0335 | 0.0102 | 0.001041 | 0.000862382 | 290.4179424 |
| rs58512942 | 10 | 105475620 | A | G | 0.141064 | 0.0165862 | 0.00249009 | 2.72584E-11 | 0.0071 | 0.0133 | 0.5942 | 0.000131842 | 44.36710852 |
| rs59985551 | 2 | 56106928 | T | C | 0.224341 | -0.0518655 | 0.00206575 | 5.5463E-139 | -0.0305 | 0.0111 | 0.00587598 | 0.001869981 | 630.3750762 |
| rs6007043 | 22 | 45838646 | C | T | 0.200322 | 0.0182298 | 0.00215724 | 2.91072E-17 | -0.0179 | 0.0116 | 0.1238 | 0.000212189 | 71.41086564 |
| rs6016223 | 20 | 38640842 | T | A | 0.824555 | 0.0143507 | 0.00227006 | 2.59006E-10 | 0.0348 | 0.0122 | 0.00415002 | 0.00011876 | 39.96399698 |
| rs6037736 | 20 | 4030226 | A | G | 0.276691 | -0.0113887 | 0.00193934 | 4.29804E-09 | -0.0035 | 0.0104 | 0.738501 | 0.000102481 | 34.4856022 |
| rs6082358 | 20 | 21230455 | T | C | 0.66947 | -0.0131016 | 0.00185312 | 1.55203E-12 | -0.0039 | 0.0099 | 0.6955 | 0.000148534 | 49.98492031 |
| rs6119945 | 20 | 31329256 | A | G | 0.176867 | -0.0128734 | 0.00228642 | 1.79958E-08 | 0.0105 | 0.0123 | 0.3936 | 9.42068E-05 | 31.70092432 |
| rs61729527 | 8 | 77761919 | T | C | 0.0511612 | -0.0280155 | 0.00392083 | 8.99705E-13 | 0.0322 | 0.021 | 0.1248 | 0.000151713 | 51.05498457 |
| rs61732778 | 3 | 187443314 | A | G | 0.0710233 | 0.0364863 | 0.00335143 | 1.34648E-27 | -0.0265 | 0.018 | 0.1404 | 0.000352123 | 118.5213971 |
| rs61776719 | 1 | 38461319 | A | C | 0.555633 | -0.0178343 | 0.00173531 | 8.99083E-25 | -0.0187 | 0.0093 | 0.0451502 | 0.000313812 | 105.6222501 |
| rs61980810 | 14 | 76265926 | A | G | 0.136934 | 0.0190371 | 0.00251178 | 3.48819E-14 | 0.0118 | 0.0135 | 0.382 | 0.000170692 | 57.44282696 |
| rs62046625 | 16 | 81578706 | A | G | 0.116304 | 0.0239693 | 0.00269915 | 6.70039E-19 | 0.0157 | 0.0145 | 0.2805 | 0.000234317 | 78.85951107 |
| rs62111457 | 19 | 7208242 | A | G | 0.407752 | -0.0153257 | 0.00176591 | 4.02254E-18 | -0.0209 | 0.0095 | 0.0278099 | 0.000223797 | 75.31837245 |
| rs62275579 | 3 | 156345708 | C | T | 0.0334105 | -0.0348595 | 0.00489911 | 1.11738E-12 | -0.0178 | 0.0261 | 0.4962 | 0.00015045 | 50.62969885 |
| rs62294340 | 3 | 169155476 | A | G | 0.383176 | 0.0106355 | 0.00177473 | 2.06557E-09 | 0.0136 | 0.0095 | 0.1533 | 0.000106722 | 35.9127499 |
| rs62296061 | 4 | 1014172 | A | G | 0.216086 | -0.0202555 | 0.00209697 | 4.51232E-22 | -0.0207 | 0.0113 | 0.06608 | 0.000277223 | 93.30371155 |
| rs62348757 | 5 | 31533952 | C | T | 0.311482 | 0.0107217 | 0.00186407 | 8.84036E-09 | 0.0049 | 0.01 | 0.6272 | 9.83125E-05 | 33.08264974 |
| rs62358926 | 5 | 37783679 | A | G | 0.07273 | 0.0212292 | 0.00333065 | 1.84523E-10 | -0.0048 | 0.0179 | 0.7904 | 0.000120727 | 40.62624477 |
| rs62370472 | 5 | 52767109 | C | T | 0.210202 | -0.0131063 | 0.002124 | 6.81099E-10 | 0.0189 | 0.0114 | 0.097949 | 0.000113149 | 38.07576195 |
| rs62390617 | 6 | 1623627 | C | G | 0.135145 | 0.0168568 | 0.00256233 | 4.75335E-11 | -0.0107 | 0.0138 | 0.4358 | 0.000128609 | 43.27903453 |
| rs62396185 | 6 | 26180634 | C | G | 0.256464 | -0.0431163 | 0.00197794 | 2.8314E-105 | -0.0519 | 0.0106 | 0.000001012 | 0.001410238 | 475.1756331 |
| rs62438578 | 6 | 158871624 | T | C | 0.232111 | 0.0177769 | 0.00204179 | 3.14775E-18 | 0.0076 | 0.011 | 0.4907 | 0.000225237 | 75.80316384 |
| rs62501195 | 8 | 24041988 | C | A | 0.171613 | -0.0296779 | 0.00232269 | 2.23718E-37 | -0.0063 | 0.0125 | 0.6136 | 0.000484978 | 163.260549 |
| rs62515437 | 8 | 57160328 | T | G | 0.225806 | 0.0335483 | 0.00206217 | 1.74301E-59 | 0.0071 | 0.0111 | 0.5239 | 0.000785958 | 264.6607418 |
| rs62621197 | 19 | 8670147 | T | C | 0.035921 | -0.111749 | 0.00477563 | 5.3456E-121 | 0.0026 | 0.0253 | 0.9194 | 0.001624682 | 547.549474 |
| rs640119 | 5 | 72402966 | A | G | 0.142303 | 0.0156314 | 0.0024799 | 2.91864E-10 | 0.0218 | 0.0134 | 0.1028 | 0.000118066 | 39.73057355 |
| rs6442266 | 3 | 11650010 | A | G | 0.95228 | 0.0239596 | 0.00405533 | 3.46234E-09 | 0.0262 | 0.0218 | 0.2296 | 0.000103731 | 34.90632632 |
| rs6463758 | 7 | 8117636 | G | A | 0.553949 | 0.0186752 | 0.00174201 | 8.23948E-27 | -0.0151 | 0.0094 | 0.1063 | 0.000341452 | 114.928213 |
| rs6482468 | 10 | 25334906 | G | A | 0.367703 | 0.0107534 | 0.00179018 | 1.89326E-09 | -0.0027 | 0.0096 | 0.780001 | 0.000107226 | 36.08241676 |
| rs6484787 | 11 | 35489891 | A | G | 0.649338 | 0.0105363 | 0.00181105 | 5.96953E-09 | 0.0165 | 0.0097 | 0.0900596 | 0.000100582 | 33.84642418 |
| rs6486109 | 11 | 13267867 | A | T | 0.260351 | 0.014211 | 0.00197461 | 6.1759E-13 | -0.0178 | 0.0106 | 0.0934093 | 0.000153911 | 51.79454631 |
| rs648831 | 6 | 80956208 | T | C | 0.530977 | 0.0224018 | 0.0017392 | 5.91425E-38 | -0.0171 | 0.0094 | 0.0680393 | 0.000492835 | 165.9068036 |
| rs6496050 | 15 | 86069250 | G | A | 0.638461 | 0.0129854 | 0.00179744 | 5.04429E-13 | -0.0041 | 0.0097 | 0.675 | 0.00015509 | 52.19143986 |
| rs6508358 | 18 | 22854030 | C | A | 0.378799 | -0.014837 | 0.00177819 | 7.21772E-17 | 0.003 | 0.0096 | 0.7535 | 0.000206869 | 69.61988231 |
| rs6550834 | 3 | 24049919 | C | G | 0.336347 | -0.0113412 | 0.00182869 | 5.5883E-10 | 0.0071 | 0.0098 | 0.4689 | 0.000114297 | 38.46230053 |
| rs6567160 | 18 | 57829135 | C | T | 0.234055 | 0.0231325 | 0.00203666 | 6.84699E-30 | 0.0313 | 0.011 | 0.00437804 | 0.000383257 | 129.0046879 |
| rs6570505 | 6 | 142616333 | C | T | 0.461084 | -0.0162127 | 0.00173843 | 1.10535E-20 | 0.0012 | 0.0093 | 0.9013 | 0.000258424 | 86.97485147 |
| rs6581626 | 12 | 65711378 | G | A | 0.53516 | -0.0206712 | 0.00173218 | 8.02417E-33 | 0.0083 | 0.0093 | 0.3715 | 0.000423068 | 142.4107444 |
| rs659418 | 11 | 75284334 | G | T | 0.140143 | 0.0362415 | 0.00248808 | 4.77749E-48 | 0.0012 | 0.0134 | 0.9312 | 0.000630171 | 212.1685786 |
| rs66930764 | 6 | 164103243 | A | G | 0.130816 | 0.0208626 | 0.00256041 | 3.70766E-16 | 0.0142 | 0.0137 | 0.3012 | 0.000197279 | 66.39192361 |
| rs6693113 | 1 | 36757801 | T | G | 0.500808 | 0.0120658 | 0.00172525 | 2.68349E-12 | 0.0065 | 0.0093 | 0.4862 | 0.000145343 | 48.91089287 |
| rs6693667 | 1 | 146817245 | T | A | 0.911162 | 0.0178172 | 0.00303036 | 4.11747E-09 | 0.0146 | 0.0163 | 0.369 | 0.000102729 | 34.56908457 |
| rs6714546 | 2 | 33361425 | G | A | 0.708352 | 0.0248916 | 0.00190344 | 4.54569E-39 | -0.025 | 0.0102 | 0.01456 | 0.00050799 | 171.0112424 |
| rs6724911 | 2 | 105876744 | G | A | 0.136764 | -0.0155364 | 0.0026295 | 3.45557E-09 | 0.0135 | 0.0141 | 0.3372 | 0.000103743 | 34.91016137 |
| rs6734579 | 2 | 60154807 | A | C | 0.561956 | -0.0112589 | 0.00174126 | 1.00811E-10 | 0.0103 | 0.0094 | 0.2719 | 0.000124239 | 41.80826017 |
| rs6739701 | 2 | 105132332 | G | A | 0.4291 | 0.0140015 | 0.00175287 | 1.37816E-15 | 0.0044 | 0.0094 | 0.641501 | 0.00018959 | 63.80388617 |
| rs6740086 | 2 | 191735930 | G | A | 0.262339 | 0.011587 | 0.00196268 | 3.55902E-09 | 0.0078 | 0.0106 | 0.4594 | 0.000103573 | 34.85302185 |
| rs67551338 | 12 | 3393100 | T | C | 0.0615245 | 0.0278945 | 0.00362819 | 1.49555E-14 | 0.0147 | 0.0195 | 0.4501 | 0.000175642 | 59.10912589 |
| rs6761320 | 2 | 129050354 | T | C | 0.43126 | -0.0108239 | 0.0017496 | 6.15772E-10 | 0.0125 | 0.0094 | 0.1845 | 0.000113734 | 38.27255193 |
| rs6762578 | 3 | 128992047 | A | G | 0.778186 | 0.030201 | 0.00208515 | 1.5838E-47 | -0.0044 | 0.0112 | 0.693999 | 0.000623083 | 209.7806663 |
| rs6762851 | 3 | 56686329 | C | T | 0.35575 | -0.0209078 | 0.00180261 | 4.24326E-31 | -0.0091 | 0.0097 | 0.3468 | 0.000399658 | 134.5273356 |
| rs6802104 | 3 | 58015300 | T | C | 0.625125 | -0.0147174 | 0.00178647 | 1.75307E-16 | -0.0305 | 0.0096 | 0.001487 | 0.000201666 | 67.86848437 |
| rs6821305 | 4 | 122713863 | C | A | 0.397184 | 0.014248 | 0.00176398 | 6.6512E-16 | 0.0162 | 0.0095 | 0.0867301 | 0.000193858 | 65.2405957 |
| rs6824748 | 4 | 17997066 | A | G | 0.156993 | -0.0601845 | 0.00236798 | 2.2909E-142 | 0.0238 | 0.0127 | 0.0616694 | 0.001916147 | 645.9675059 |
| rs686489 | 11 | 57659832 | G | A | 0.439247 | 0.0102957 | 0.00174305 | 3.4939E-09 | 0.024 | 0.0094 | 0.0104299 | 0.00010368 | 34.88907755 |
| rs6872083 | 5 | 39418421 | G | A | 0.293401 | -0.0181767 | 0.00190401 | 1.35021E-21 | -0.0053 | 0.0102 | 0.6017 | 0.000270784 | 91.13580779 |
| rs6874142 | 5 | 172753555 | G | T | 0.111485 | 0.0303324 | 0.00286201 | 3.06408E-26 | 0.0333 | 0.0154 | 0.03094 | 0.000333714 | 112.3229947 |
| rs6889138 | 5 | 90335635 | A | G | 0.291605 | -0.0114669 | 0.00189935 | 1.5691E-09 | 0.0051 | 0.0102 | 0.6169 | 0.000108314 | 36.44848376 |
| rs6902771 | 6 | 152157881 | T | C | 0.462804 | 0.0242501 | 0.00172616 | 8.0891E-45 | -0.0164 | 0.0093 | 0.0782402 | 0.000586218 | 197.3614781 |
| rs6906411 | 6 | 81913276 | A | G | 0.448333 | -0.0241131 | 0.00174643 | 2.37356E-43 | 0.0113 | 0.0094 | 0.2304 | 0.000566247 | 190.6343452 |
| rs6919321 | 6 | 33724004 | A | G | 0.608131 | 0.0148398 | 0.00177439 | 6.12209E-17 | -0.0233 | 0.0095 | 0.0147001 | 0.000207834 | 69.94478698 |
| rs6938592 | 6 | 45097503 | T | A | 0.743343 | 0.019919 | 0.00197743 | 7.31476E-24 | 0.0246 | 0.0106 | 0.02069 | 0.000301474 | 101.4682673 |
| rs6950680 | 7 | 120790287 | G | A | 0.3854 | -0.0127068 | 0.00177341 | 7.78574E-13 | -0.0003 | 0.0095 | 0.9781 | 0.000152558 | 51.33948421 |
| rs6956736 | 7 | 148893472 | C | G | 0.745454 | -0.0124511 | 0.00198513 | 3.56443E-10 | -0.0156 | 0.0107 | 0.1446 | 0.000116906 | 39.34005435 |
| rs6960257 | 7 | 73302272 | G | A | 0.397885 | -0.010623 | 0.00179346 | 3.16068E-09 | -0.0082 | 0.0096 | 0.3925 | 0.000104259 | 35.08394259 |
| rs6962887 | 7 | 135045786 | G | T | 0.307142 | -0.0169302 | 0.0018794 | 2.10329E-19 | 0.0113 | 0.0101 | 0.2643 | 0.000241118 | 81.14899593 |
| rs7027509 | 9 | 136927876 | T | C | 0.328465 | 0.0109824 | 0.00183729 | 2.26778E-09 | -0.0073 | 0.0099 | 0.4598 | 0.00010618 | 35.73028752 |
| rs7030316 | 9 | 99250463 | T | C | 0.18802 | 0.0326235 | 0.00220569 | 1.74663E-49 | -0.014 | 0.0119 | 0.2373 | 0.000649738 | 218.7608362 |
| rs7031064 | 9 | 14455076 | G | A | 0.485749 | 0.0107935 | 0.00172628 | 4.0452E-10 | -0.0165 | 0.0093 | 0.0760799 | 0.000116172 | 39.09304796 |
| rs703998 | 10 | 80912499 | T | A | 0.404967 | -0.0210989 | 0.0017708 | 1.00531E-32 | 0.0094 | 0.0095 | 0.3233 | 0.000421741 | 141.963932 |
| rs704660 | 11 | 30447998 | T | C | 0.409887 | 0.0147632 | 0.00175445 | 3.95276E-17 | 0.0095 | 0.0094 | 0.3122 | 0.000210395 | 70.807039 |
| rs7090035 | 10 | 100026791 | C | G | 0.429295 | 0.0131893 | 0.00174723 | 4.40859E-14 | 0.0047 | 0.0094 | 0.618399 | 0.000169324 | 56.9824025 |
| rs7090067 | 10 | 81234628 | C | T | 0.158269 | 0.0173332 | 0.0023683 | 2.50669E-13 | -0.0026 | 0.0127 | 0.8385 | 0.000159171 | 53.56504634 |
| rs7102462 | 11 | 122828767 | G | C | 0.568526 | 0.013052 | 0.00174502 | 7.47309E-14 | -0.0148 | 0.0094 | 0.1144 | 0.000166238 | 55.94364136 |
| rs71356070 | 18 | 2809160 | T | C | 0.472058 | 0.0107714 | 0.00173381 | 5.21795E-10 | 0.0144 | 0.0093 | 0.122 | 0.000114694 | 38.59568199 |
| rs71385734 | 16 | 2160503 | G | T | 0.169452 | -0.0291475 | 0.00231421 | 2.29245E-36 | 0.008 | 0.0125 | 0.5185 | 0.000471239 | 158.6333547 |
| rs71472903 | 15 | 51582242 | C | T | 0.0422999 | -0.0307196 | 0.00431818 | 1.12928E-12 | 0.0481 | 0.0233 | 0.03874 | 0.000150388 | 50.60891752 |
| rs71499905 | 9 | 98267643 | T | A | 0.0271635 | -0.0364581 | 0.00531449 | 6.89128E-12 | 0.0258 | 0.0285 | 0.3646 | 0.000139847 | 47.06112381 |
| rs7208285 | 17 | 76746325 | A | G | 0.52698 | -0.0130782 | 0.0017342 | 4.66445E-14 | -0.006 | 0.0093 | 0.5193 | 0.000168995 | 56.87154202 |
| rs7221345 | 17 | 45405144 | A | G | 0.60008 | -0.0137084 | 0.00176201 | 7.27445E-15 | 0.0189 | 0.0095 | 0.0464505 | 0.000179857 | 60.52770642 |
| rs7223535 | 17 | 29211667 | A | G | 0.270191 | -0.0408306 | 0.00194161 | 4.09638E-98 | -0.0343 | 0.0104 | 0.00100399 | 0.001312579 | 442.2266816 |
| rs723149 | 7 | 46577056 | G | A | 0.563721 | -0.018316 | 0.00173961 | 6.4254E-26 | 0.0013 | 0.0094 | 0.8878 | 0.000329354 | 110.8549007 |
| rs7235010 | 18 | 20724810 | A | G | 0.784089 | 0.0505729 | 0.00209297 | 6.9984E-129 | 0.0001 | 0.0113 | 0.994 | 0.001732229 | 583.8578558 |
| rs72656010 | 8 | 57122215 | C | T | 0.130834 | -0.0483961 | 0.00256462 | 2.18524E-79 | -0.0307 | 0.0138 | 0.0259102 | 0.001057216 | 356.1001035 |
| rs72722756 | 8 | 129186110 | C | T | 0.177512 | 0.02071 | 0.00226335 | 5.71347E-20 | 0.0254 | 0.0122 | 0.0363396 | 0.00024877 | 83.72481202 |
| rs72742475 | 1 | 218879783 | T | C | 0.0222406 | 0.0396022 | 0.00584784 | 1.27116E-11 | -0.0378 | 0.0313 | 0.2273 | 0.000136282 | 45.86116479 |
| rs72755233 | 15 | 100692953 | A | G | 0.112887 | -0.06223 | 0.00272175 | 1.3002E-115 | -0.0065 | 0.0147 | 0.6567 | 0.001551234 | 522.757843 |
| rs72776502 | 5 | 77539937 | C | T | 0.236291 | -0.0197564 | 0.00202915 | 2.12618E-22 | -0.0156 | 0.0109 | 0.1533 | 0.000281652 | 94.79484808 |
| rs72800392 | 5 | 134356061 | T | C | 0.304175 | -0.0248048 | 0.00188036 | 1.00485E-39 | 0.0127 | 0.0101 | 0.2086 | 0.000516908 | 174.0150737 |
| rs72801818 | 16 | 53476042 | T | C | 0.300935 | 0.0222145 | 0.00188069 | 3.43795E-32 | -0.0065 | 0.0101 | 0.5236 | 0.000414483 | 139.5198733 |
| rs72843872 | 2 | 85820027 | G | A | 0.307112 | 0.0145933 | 0.0018719 | 6.41062E-15 | -0.0105 | 0.0101 | 0.2974 | 0.000180598 | 60.77698486 |
| rs72887152 | 2 | 156604025 | A | T | 0.165933 | 0.0169227 | 0.00231955 | 2.97852E-13 | 0.0025 | 0.0125 | 0.8383 | 0.000158165 | 53.2266646 |
| rs72891014 | 4 | 109418112 | T | C | 0.296395 | 0.0150646 | 0.00188953 | 1.55776E-15 | -0.0143 | 0.0102 | 0.1598 | 0.000188875 | 63.56309632 |
| rs72904749 | 1 | 51367420 | T | C | 0.0892173 | 0.0354582 | 0.00302274 | 9.02402E-32 | -0.0022 | 0.0163 | 0.8936 | 0.000408792 | 137.6034121 |
| rs72931083 | 2 | 202039524 | A | G | 0.0330932 | 0.0304183 | 0.00482155 | 2.81514E-10 | -0.0663 | 0.0258 | 0.0101899 | 0.000118275 | 39.80099658 |
| rs7293204 | 22 | 46231145 | T | G | 0.419699 | 0.0132233 | 0.00175862 | 5.52841E-14 | -0.0128 | 0.0094 | 0.1743 | 0.000168001 | 56.53704488 |
| rs7295354 | 12 | 124777480 | T | C | 0.308488 | 0.0168825 | 0.00186933 | 1.70451E-19 | 0.0007 | 0.01 | 0.9453 | 0.000242351 | 81.56408784 |
| rs72976986 | 19 | 4050424 | A | G | 0.191187 | 0.0174154 | 0.00221813 | 4.12762E-15 | -0.0091 | 0.0119 | 0.4454 | 0.000183173 | 61.64393232 |
| rs7299310 | 12 | 66121262 | C | T | 0.1369 | 0.0166805 | 0.00251 | 3.02413E-11 | 0.0304 | 0.0135 | 0.0241002 | 0.000131239 | 44.16396984 |
| rs73125634 | 20 | 20069826 | T | G | 0.278233 | -0.0113267 | 0.00193362 | 4.69559E-09 | -0.0092 | 0.0104 | 0.3742 | 0.000101969 | 34.31325798 |
| rs73148102 | 7 | 65438864 | A | G | 0.214631 | 0.0162684 | 0.00210888 | 1.22011E-14 | 0.0201 | 0.0113 | 0.0754102 | 0.000176831 | 59.50909786 |
| rs7319045 | 13 | 92024574 | G | A | 0.631529 | -0.0210747 | 0.00178793 | 4.60999E-32 | 0.0084 | 0.0096 | 0.3837 | 0.000412754 | 138.9374097 |
| rs73259805 | 5 | 137768494 | T | C | 0.171678 | 0.0124804 | 0.00228607 | 4.783E-08 | -0.0066 | 0.0123 | 0.5891 | 8.85703E-05 | 29.80405658 |
| rs7330667 | 13 | 21447045 | T | C | 0.818785 | -0.0176379 | 0.00223792 | 3.24713E-15 | 0.0205 | 0.012 | 0.0886401 | 0.000184575 | 62.11579607 |
| rs73453871 | 15 | 89261671 | A | G | 0.151943 | 0.0145885 | 0.00241708 | 1.58544E-09 | 0.0353 | 0.013 | 0.00657204 | 0.000108253 | 36.42811076 |
| rs734764 | 9 | 90852624 | C | G | 0.788294 | -0.0237934 | 0.00211669 | 2.59956E-29 | 0.0143 | 0.0114 | 0.2103 | 0.000375391 | 126.356013 |
| rs74187143 | 2 | 220051676 | G | A | 0.0378011 | 0.0441885 | 0.00452419 | 1.56819E-22 | -0.0019 | 0.0243 | 0.9376 | 0.000283441 | 95.39690096 |
| rs7430034 | 3 | 134342127 | T | C | 0.628682 | 0.0144629 | 0.00179238 | 7.10559E-16 | -0.0009 | 0.0096 | 0.9278 | 0.000193471 | 65.1100467 |
| rs743760 | 13 | 33071127 | C | G | 0.349203 | 0.0131516 | 0.00181998 | 4.97622E-13 | -0.003 | 0.0098 | 0.7573 | 0.000155169 | 52.21813667 |
| rs745749 | 5 | 179715803 | G | A | 0.334958 | -0.012897 | 0.00184136 | 2.49115E-12 | -0.0139 | 0.0099 | 0.1599 | 0.000145776 | 49.05659592 |
| rs74590624 | 11 | 68155790 | T | C | 0.155901 | 0.0140642 | 0.00238588 | 3.75569E-09 | -0.0053 | 0.0128 | 0.6805 | 0.000103261 | 34.74803838 |
| rs7468898 | 9 | 118544291 | T | G | 0.446177 | -0.00975262 | 0.00173334 | 1.84069E-08 | -0.0114 | 0.0093 | 0.22 | 9.40768E-05 | 31.6572004 |
| rs747680 | 20 | 61507075 | C | T | 0.50489 | 0.0108399 | 0.00172488 | 3.29428E-10 | 0.011 | 0.0093 | 0.236 | 0.000117363 | 39.49391589 |
| rs75076724 | 14 | 104014690 | C | T | 0.143113 | 0.0225917 | 0.00247793 | 7.75175E-20 | -0.0095 | 0.0133 | 0.4779 | 0.00024698 | 83.12222807 |
| rs75091181 | 2 | 232986399 | T | C | 0.0793211 | 0.0353844 | 0.00325988 | 1.91867E-27 | 0.0261 | 0.0176 | 0.1371 | 0.000350039 | 117.8196866 |
| rs7516464 | 1 | 71518153 | T | A | 0.264612 | 0.012861 | 0.00195943 | 5.25775E-11 | -0.0085 | 0.0105 | 0.4189 | 0.000128021 | 43.08116003 |
| rs7524908 | 1 | 10290640 | C | T | 0.503643 | 0.0128839 | 0.00172682 | 8.60201E-14 | 0.0028 | 0.0093 | 0.7622 | 0.000165416 | 55.66701951 |
| rs7532266 | 1 | 23551623 | C | A | 0.694898 | 0.0124543 | 0.00187946 | 3.44191E-11 | 0.0195 | 0.0101 | 0.0530603 | 0.000130486 | 43.91065242 |
| rs753602 | 16 | 88807224 | C | T | 0.70197 | 0.0197835 | 0.0018883 | 1.11353E-25 | 0.0376 | 0.0102 | 0.000215998 | 0.000326115 | 109.7644453 |
| rs75475720 | 5 | 108200114 | G | C | 0.0834433 | 0.0340124 | 0.00312807 | 1.56207E-27 | -0.0152 | 0.0168 | 0.365 | 0.000351251 | 118.2276475 |
| rs7571231 | 2 | 36726921 | G | T | 0.273823 | -0.0161761 | 0.00194091 | 7.82528E-17 | -0.0092 | 0.0104 | 0.3764 | 0.000206393 | 69.4599185 |
| rs7585767 | 2 | 70338344 | G | A | 0.487457 | -0.0138155 | 0.0017445 | 2.39276E-15 | -0.0097 | 0.0094 | 0.2986 | 0.000186363 | 62.71749233 |
| rs7593987 | 2 | 44393532 | G | A | 0.808763 | -0.0234985 | 0.00219449 | 9.43192E-27 | 0.0071 | 0.0118 | 0.5449 | 0.000340654 | 114.6596171 |
| rs7599488 | 2 | 60718347 | T | C | 0.426383 | -0.00991768 | 0.00174366 | 1.2876E-08 | 0.0081 | 0.0094 | 0.3869 | 9.61398E-05 | 32.35146796 |
| rs76156143 | 15 | 77365134 | T | C | 0.0892767 | 0.0234844 | 0.00302692 | 8.61589E-15 | 0.0088 | 0.0163 | 0.590999 | 0.000178866 | 60.19417556 |
| rs76157805 | 12 | 102571933 | G | A | 0.014398 | -0.0809239 | 0.00722871 | 4.37522E-29 | 0.0031 | 0.0386 | 0.9362 | 0.000372322 | 125.3226079 |
| rs7621604 | 3 | 61557718 | G | A | 0.445442 | -0.0199447 | 0.00173717 | 1.66341E-30 | -0.0102 | 0.0093 | 0.2753 | 0.000391605 | 131.8158983 |
| rs76359852 | 2 | 174768849 | T | A | 0.18874 | -0.0149466 | 0.00221461 | 1.49005E-11 | -0.009 | 0.0119 | 0.4504 | 0.000135357 | 45.54993164 |
| rs76364830 | 8 | 13372120 | A | G | 0.0627567 | -0.0315998 | 0.00359533 | 1.51391E-18 | 0.0063 | 0.0193 | 0.744399 | 0.00022953 | 77.2482357 |
| rs7641322 | 3 | 145868489 | T | G | 0.548298 | -0.00997483 | 0.00173257 | 8.55658E-09 | 0.0119 | 0.0093 | 0.2011 | 9.84998E-05 | 33.14567317 |
| rs7651940 | 3 | 169256240 | T | C | 0.511121 | -0.0117869 | 0.00173323 | 1.044E-11 | -0.0048 | 0.0093 | 0.603 | 0.000137428 | 46.24706984 |
| rs7652177 | 3 | 171969077 | G | C | 0.504597 | 0.0264951 | 0.00172445 | 2.95937E-53 | -0.0037 | 0.0093 | 0.690601 | 0.00070109 | 236.0626789 |
| rs76604417 | 1 | 119425396 | C | T | 0.124517 | 0.0150758 | 0.00262033 | 8.75447E-09 | -0.0246 | 0.0141 | 0.0814892 | 9.83682E-05 | 33.10138572 |
| rs768023 | 6 | 108876002 | A | G | 0.628564 | 0.0177553 | 0.00178517 | 2.64545E-23 | 0.0244 | 0.0096 | 0.0110999 | 0.000293912 | 98.92231887 |
| rs7681267 | 4 | 87073599 | C | T | 0.983019 | 0.0443951 | 0.00673259 | 4.28549E-11 | 0.007 | 0.0362 | 0.8462 | 0.000129211 | 43.4813641 |
| rs7688346 | 4 | 38677453 | G | A | 0.14038 | -0.0174215 | 0.00250409 | 3.47696E-12 | 0.0145 | 0.0135 | 0.2821 | 0.000143833 | 48.40259447 |
| rs76895963 | 12 | 4384844 | G | T | 0.0192794 | 0.132179 | 0.00668555 | 5.95525E-87 | 0.0205 | 0.0353 | 0.562101 | 0.001160366 | 390.8841495 |
| rs7697556 | 4 | 73515313 | C | T | 0.52566 | -0.0242096 | 0.00172691 | 1.22772E-44 | -0.0024 | 0.0093 | 0.792301 | 0.000583755 | 196.5319837 |
| rs76997372 | 4 | 144555923 | T | C | 0.154293 | 0.0148243 | 0.002385 | 5.11694E-10 | 0.0141 | 0.0128 | 0.2708 | 0.000114807 | 38.63394367 |
| rs7705189 | 5 | 131623358 | G | A | 0.465249 | 0.0288754 | 0.00173802 | 5.84521E-62 | -0.0155 | 0.0093 | 0.096801 | 0.00081967 | 276.0221264 |
| rs77124518 | 15 | 38362264 | C | T | 0.109075 | 0.0166653 | 0.00277118 | 1.81422E-09 | 0.0191 | 0.0149 | 0.2006 | 0.000107473 | 36.16547875 |
| rs77216358 | 11 | 120311157 | G | A | 0.0465422 | -0.0376258 | 0.00413547 | 9.22147E-20 | 0.0163 | 0.0222 | 0.4642 | 0.000245959 | 82.77880537 |
| rs7744288 | 6 | 34153441 | A | G | 0.0183304 | 0.0531573 | 0.00642521 | 1.30828E-16 | 0.0526 | 0.0347 | 0.1289 | 0.000203381 | 68.44608197 |
| rs7753558 | 6 | 117523471 | A | C | 0.633709 | 0.0183603 | 0.00180407 | 2.52872E-24 | 0.0016 | 0.0097 | 0.8723 | 0.000307728 | 103.573868 |
| rs77672559 | 17 | 28135103 | T | A | 0.110076 | -0.0235875 | 0.00276648 | 1.5188E-17 | -0.0376 | 0.0148 | 0.01104 | 0.000216005 | 72.69527355 |
| rs7774592 | 6 | 129823611 | C | T | 0.743177 | 0.01488 | 0.00197501 | 4.9272E-14 | -0.0026 | 0.0106 | 0.8048 | 0.000168672 | 56.76291409 |
| rs77797953 | 12 | 680676 | G | A | 0.0805044 | 0.0199124 | 0.00320478 | 5.1929E-10 | 0.0022 | 0.0172 | 0.8984 | 0.000114723 | 38.60541192 |
| rs78051210 | 6 | 131379491 | C | T | 0.0772111 | 0.0347863 | 0.003268 | 1.86767E-26 | 0.0144 | 0.0175 | 0.4124 | 0.000336631 | 113.3051888 |
| rs7814625 | 8 | 130541447 | A | G | 0.363794 | -0.0129711 | 0.00180057 | 5.86273E-13 | -0.0041 | 0.0097 | 0.667999 | 0.000154211 | 51.89565691 |
| rs7816131 | 8 | 130719718 | T | A | 0.201629 | -0.036711 | 0.0021493 | 2.2136E-65 | 0.0094 | 0.0115 | 0.4175 | 0.000866304 | 291.7398603 |
| rs78198962 | 2 | 233094868 | T | C | 0.0318477 | 0.0754162 | 0.00490916 | 3.05281E-53 | -0.0022 | 0.0263 | 0.9337 | 0.000700905 | 236.0001811 |
| rs78760367 | 7 | 5392630 | A | G | 0.116538 | -0.0163386 | 0.00268113 | 1.10301E-09 | -0.0059 | 0.0145 | 0.6849 | 0.000110356 | 37.13567783 |
| rs78805413 | 11 | 2131018 | A | G | 0.0571354 | -0.034578 | 0.00379588 | 8.33105E-20 | -0.0108 | 0.0203 | 0.5935 | 0.000246556 | 82.97976669 |
| rs78817479 | 9 | 17040371 | A | C | 0.0857313 | -0.0211432 | 0.00309354 | 8.23379E-12 | -0.0166 | 0.0167 | 0.3194 | 0.000138809 | 46.71188388 |
| rs7908715 | 10 | 23803020 | T | C | 0.334713 | 0.0103871 | 0.00183605 | 1.53869E-08 | 0.0125 | 0.0099 | 0.2042 | 9.51101E-05 | 32.00494139 |
| rs7911018 | 10 | 131348554 | T | C | 0.607781 | 0.0117641 | 0.0017685 | 2.89468E-11 | -0.0046 | 0.0095 | 0.6275 | 0.000131492 | 44.24912685 |
| rs79112217 | 1 | 218622949 | C | T | 0.107512 | 0.0303671 | 0.00280263 | 2.36919E-27 | -0.0089 | 0.0151 | 0.5577 | 0.000348796 | 117.401197 |
| rs7916821 | 10 | 69933969 | A | G | 0.497337 | 0.0193418 | 0.00172536 | 3.67198E-29 | -0.0166 | 0.0093 | 0.0726708 | 0.000373354 | 125.6700374 |
| rs79378907 | 22 | 29346667 | T | C | 0.0317335 | -0.0379199 | 0.00493835 | 1.61176E-14 | 0.01 | 0.0265 | 0.706999 | 0.000175204 | 58.96143593 |
| rs7952436 | 11 | 67024534 | T | C | 0.0832031 | -0.0780658 | 0.00312331 | 9.3756E-138 | -0.0466 | 0.0168 | 0.00554498 | 0.001853251 | 624.7249693 |
| rs7968682 | 12 | 66371880 | T | G | 0.51578 | -0.0429273 | 0.00173284 | 2.3335E-135 | -0.0192 | 0.0093 | 0.0392898 | 0.00182057 | 613.6880077 |
| rs7974437 | 12 | 31735697 | A | G | 0.68941 | -0.0117231 | 0.00186755 | 3.44953E-10 | -0.0068 | 0.01 | 0.4978 | 0.000117095 | 39.40377964 |
| rs79749090 | 17 | 27417209 | G | A | 0.0732268 | 0.0184299 | 0.00335413 | 3.91679E-08 | 0.0186 | 0.0181 | 0.3032 | 8.97213E-05 | 30.19142377 |
| rs798488 | 7 | 2802522 | C | T | 0.300367 | -0.0438036 | 0.00188204 | 1E-119 | -0.005 | 0.0101 | 0.6206 | 0.001607355 | 541.7006149 |
| rs7987477 | 13 | 73709446 | G | A | 0.131487 | -0.0171859 | 0.00259595 | 3.59087E-11 | -0.0111 | 0.0139 | 0.4253 | 0.00013024 | 43.82776584 |
| rs79883557 | 2 | 68383901 | T | C | 0.0810234 | -0.0246413 | 0.0031652 | 6.98715E-15 | 0.0028 | 0.0169 | 0.8682 | 0.000180092 | 60.60693712 |
| rs8006145 | 14 | 64699450 | A | C | 0.28177 | -0.0130786 | 0.00191647 | 8.84912E-12 | 0.0027 | 0.0103 | 0.7913 | 0.000138391 | 46.57103424 |
| rs8019890 | 14 | 21538067 | A | C | 0.53047 | 0.0153983 | 0.00175383 | 1.64513E-18 | -0.0253 | 0.0094 | 0.00719896 | 0.000229044 | 77.08466379 |
| rs8023451 | 15 | 101789281 | T | G | 0.158231 | -0.0174214 | 0.00238279 | 2.65033E-13 | -0.0021 | 0.0128 | 0.8701 | 0.000158845 | 53.45544949 |
| rs8029053 | 15 | 89450087 | T | C | 0.285889 | -0.015693 | 0.0019167 | 2.67609E-16 | 0.0015 | 0.0103 | 0.8833 | 0.000199189 | 67.03491798 |
| rs8035987 | 15 | 75750383 | C | T | 0.252423 | 0.0174564 | 0.00199673 | 2.28981E-18 | -0.0329 | 0.0107 | 0.002157 | 0.000227102 | 76.43074664 |
| rs8074840 | 17 | 1674429 | C | T | 0.303677 | 0.0155415 | 0.00187376 | 1.09623E-16 | -0.0137 | 0.0101 | 0.1754 | 0.000204417 | 68.79475951 |
| rs8112355 | 19 | 10731745 | C | T | 0.780816 | -0.0209342 | 0.00208931 | 1.25835E-23 | -0.0232 | 0.0112 | 0.0383301 | 0.000298281 | 100.3932216 |
| rs8125976 | 20 | 48599020 | C | T | 0.117567 | -0.0231148 | 0.00267617 | 5.78096E-18 | 0.0255 | 0.0144 | 0.0751104 | 0.000221669 | 74.60197118 |
| rs8181166 | 9 | 89116628 | C | G | 0.498821 | 0.0184344 | 0.00173145 | 1.82264E-26 | 0.018 | 0.0093 | 0.0523805 | 0.000336775 | 113.3536536 |
| rs822530 | 7 | 148631555 | T | A | 0.794451 | 0.0346333 | 0.00214994 | 2.32006E-58 | -0.0121 | 0.0116 | 0.2957 | 0.000770635 | 259.4970879 |
| rs823116 | 1 | 205720483 | A | G | 0.546802 | -0.0155649 | 0.00173123 | 2.46888E-19 | 0.0037 | 0.0093 | 0.6925 | 0.000240175 | 80.83148353 |
| rs832552 | 5 | 56113850 | T | G | 0.652926 | 0.0148866 | 0.00181494 | 2.36755E-16 | -0.0018 | 0.0097 | 0.8538 | 0.000199907 | 67.27658096 |
| rs862041 | 14 | 74986403 | C | T | 0.637235 | 0.0225572 | 0.00179728 | 4.01421E-36 | 0.0064 | 0.0097 | 0.5095 | 0.000467933 | 157.5202222 |
| rs876122 | 6 | 6886297 | G | A | 0.879417 | 0.0256229 | 0.00264761 | 3.77485E-22 | 0.0655 | 0.0142 | 4.21299E-06 | 0.000278276 | 93.65823223 |
| rs880090 | 19 | 19740729 | G | C | 0.737978 | -0.0117615 | 0.00196774 | 2.27269E-09 | 0.0121 | 0.0106 | 0.2527 | 0.000106168 | 35.72624864 |
| rs893819 | 15 | 74229524 | G | A | 0.353548 | 0.0183736 | 0.00180169 | 2.04174E-24 | -0.0107 | 0.0097 | 0.2699 | 0.000308989 | 103.9981939 |
| rs903347 | 1 | 235586613 | T | C | 0.592051 | 0.0103873 | 0.00175399 | 3.18149E-09 | -0.0014 | 0.0094 | 0.8802 | 0.000104221 | 35.07103173 |
| rs9300607 | 13 | 101223459 | C | A | 0.480006 | -0.0100402 | 0.00172937 | 6.41549E-09 | 0.0125 | 0.0093 | 0.1773 | 0.000100165 | 33.70592925 |
| rs933561 | 16 | 49874676 | G | A | 0.347575 | -0.012969 | 0.00181292 | 8.46837E-13 | -0.0072 | 0.0098 | 0.4593 | 0.000152068 | 51.17444235 |
| rs9359128 | 6 | 76250456 | T | C | 0.114825 | 0.0298366 | 0.00270221 | 2.43557E-28 | -0.0058 | 0.0146 | 0.692 | 0.000362203 | 121.9152094 |
| rs9365939 | 6 | 166336825 | G | A | 0.550031 | -0.0115781 | 0.00173238 | 2.33938E-11 | 0.0013 | 0.0093 | 0.8857 | 0.000132733 | 44.66688727 |
| rs9367296 | 6 | 47635800 | C | T | 0.637078 | 0.010134 | 0.00179851 | 1.75517E-08 | -0.0248 | 0.0097 | 0.01037 | 9.43504E-05 | 31.74925249 |
| rs9379084 | 6 | 7231843 | A | G | 0.117158 | -0.0330416 | 0.00278516 | 1.85952E-32 | -0.0308 | 0.0149 | 0.0391796 | 0.000418108 | 140.7405548 |
| rs9391254 | 6 | 105377347 | T | C | 0.321611 | 0.0374108 | 0.00184542 | 2.56744E-91 | 0.0007 | 0.0099 | 0.9407 | 0.001219893 | 410.9613347 |
| rs941972 | 1 | 42040740 | C | T | 0.395391 | 0.0104764 | 0.0017678 | 3.10227E-09 | -0.003 | 0.0095 | 0.755099 | 0.000104366 | 35.12006437 |
| rs9435731 | 1 | 17306029 | A | C | 0.520523 | 0.0288937 | 0.00172613 | 7.21772E-63 | -0.0028 | 0.0093 | 0.759899 | 0.000832044 | 280.1926481 |
| rs9442571 | 1 | 9349611 | A | T | 0.129357 | 0.0276147 | 0.00258385 | 1.17679E-26 | 0.0122 | 0.0139 | 0.3798 | 0.000339349 | 114.2203487 |
| rs9496369 | 6 | 142724918 | T | C | 0.28353 | -0.0442541 | 0.00191479 | 4.3652E-118 | 0.0034 | 0.0103 | 0.738901 | 0.001584982 | 534.1486596 |
| rs9575875 | 13 | 85931285 | G | A | 0.37463 | 0.0115364 | 0.00178456 | 1.01716E-10 | 0.0078 | 0.0096 | 0.4158 | 0.000124186 | 41.79032241 |
| rs9607978 | 22 | 43284334 | A | G | 0.492641 | -0.0116696 | 0.00172512 | 1.33999E-11 | 0.0065 | 0.0093 | 0.480799 | 0.000135976 | 45.75839028 |
| rs9634212 | 12 | 93993266 | A | C | 0.221307 | 0.0411581 | 0.00208205 | 6.28637E-87 | 0.0411 | 0.0112 | 0.000238199 | 0.001160039 | 390.7740761 |
| rs9693857 | 8 | 9267117 | T | C | 0.443332 | 0.0123561 | 0.00174103 | 1.27703E-12 | -0.0261 | 0.0094 | 0.00532599 | 0.00014967 | 50.36719188 |
| rs9696458 | 9 | 136398985 | T | C | 0.333102 | -0.0125274 | 0.00189337 | 3.68468E-11 | 0.0137 | 0.0101 | 0.1776 | 0.00013009 | 43.7772347 |
| rs9747062 | 17 | 8023057 | C | A | 0.604584 | 0.0162396 | 0.00176898 | 4.32514E-20 | 0.0015 | 0.0095 | 0.8713 | 0.000250406 | 84.27567286 |
| rs9803993 | 1 | 243672125 | A | G | 0.222429 | 0.0166713 | 0.0020856 | 1.31553E-15 | -0.0187 | 0.0112 | 0.09621 | 0.000189864 | 63.89609478 |
| rs9809116 | 3 | 72397279 | G | A | 0.40736 | -0.0262773 | 0.00176926 | 6.98715E-50 | 0.0021 | 0.0095 | 0.8242 | 0.000655152 | 220.5847982 |
| rs9824877 | 3 | 98896242 | A | G | 0.224647 | -0.0119441 | 0.00216851 | 3.63295E-08 | -0.0095 | 0.0116 | 0.4157 | 9.01557E-05 | 30.33761403 |
| rs9826470 | 3 | 38019428 | T | C | 0.249213 | 0.0183658 | 0.00200524 | 5.27108E-20 | -0.0147 | 0.0108 | 0.1727 | 0.000249245 | 83.88501786 |
| rs985344 | 3 | 67347767 | A | G | 0.101278 | -0.0201363 | 0.00287472 | 2.48142E-12 | 0.015 | 0.0154 | 0.3302 | 0.000145799 | 49.06430636 |
| rs9880232 | 3 | 185360578 | A | C | 0.701749 | 0.0120957 | 0.00189554 | 1.75991E-10 | -0.0053 | 0.0102 | 0.6055 | 0.000121002 | 40.71866449 |
| rs989393 | 9 | 101743336 | C | T | 0.28903 | -0.0156193 | 0.00190673 | 2.58523E-16 | -0.0319 | 0.0102 | 0.00184599 | 0.000199391 | 67.10303327 |
| rs9910161 | 17 | 59606244 | G | A | 0.284896 | 0.0160282 | 0.00191937 | 6.80613E-17 | -0.0162 | 0.0103 | 0.1164 | 0.00020721 | 69.73479355 |
| rs9974406 | 21 | 47435594 | A | T | 0.226587 | -0.0145649 | 0.00206503 | 1.75267E-12 | -0.0197 | 0.0111 | 0.0753998 | 0.000147825 | 49.74617603 |
| rs9988728 | 10 | 53289428 | A | G | 0.269544 | 0.0143639 | 0.00195366 | 1.95209E-13 | 0.003 | 0.0105 | 0.776201 | 0.00016063 | 54.05603599 |
| Genome-wide significant SNPs for Standing height（ukb-b-10787） | | | | | | | | | | | | | |
| SNP | Chr | Position | EA | OA | EAF | Standing height | | | Knee Osteoarthritis | | | R2 | F |
|  |  |  |  |  |  | beta | SE | pval | beta | SE | pval |  |  |
| rs10010325 | 4 | 106106353 | A | C | 0.484567 | 0.0219746 | 0.00130578 | 1.50003E-63 | -0.0078 | 0.0093 | 0.4017 | 0.00061269 | 283.204436 |
| rs10011137 | 4 | 56498199 | G | A | 0.559232 | 0.00889408 | 0.00131632 | 1.39991E-11 | -0.0065 | 0.0094 | 0.4875 | 9.88192E-05 | 45.65383058 |
| rs10031777 | 4 | 48498290 | C | T | 0.503003 | 0.0127793 | 0.00130223 | 9.8992E-23 | -0.0065 | 0.0097 | 0.5047 | 0.000208427 | 96.30234411 |
| rs10046853 | 9 | 14456931 | G | C | 0.45383 | 0.0099425 | 0.00131417 | 3.90032E-14 | -0.0175 | 0.0093 | 0.0608303 | 0.000123891 | 57.23823341 |
| rs1004982 | 15 | 51613811 | C | T | 0.383694 | -0.0125604 | 0.00134797 | 1.20005E-20 | 0.0142 | 0.0096 | 0.1392 | 0.000187919 | 86.82496294 |
| rs10059884 | 5 | 32832474 | A | C | 0.596197 | -0.0272971 | 0.00132891 | 9.30037E-94 | -0.0126 | 0.0095 | 0.1838 | 0.000912537 | 421.9296081 |
| rs10061757 | 5 | 56086805 | C | G | 0.652792 | 0.0133108 | 0.00137177 | 2.90001E-22 | 0.0001 | 0.0098 | 0.9921 | 0.00020378 | 94.1550968 |
| rs10082476 | 10 | 124164654 | G | A | 0.246683 | -0.0185987 | 0.00152018 | 1.99986E-34 | -0.0357 | 0.0108 | 0.000910102 | 0.000323921 | 149.6832724 |
| rs10098103 | 8 | 23397081 | C | T | 0.243018 | -0.010138 | 0.00152761 | 3.19963E-11 | 0.0033 | 0.0108 | 0.7642 | 9.5333E-05 | 44.04308263 |
| rs10105330 | 8 | 58136501 | T | C | 0.096945 | 0.0147157 | 0.00221217 | 2.90001E-11 | 0.0094 | 0.0157 | 0.5485 | 9.5783E-05 | 44.25098823 |
| rs10114341 | 9 | 96919182 | C | T | 0.440289 | 0.00855366 | 0.00131835 | 8.69961E-11 | -0.0074 | 0.0094 | 0.4262 | 9.11188E-05 | 42.09598147 |
| rs10114881 | 9 | 76676071 | C | T | 0.316802 | 0.00811157 | 0.00140559 | 7.90005E-09 | -0.0053 | 0.01 | 0.5928 | 7.20885E-05 | 33.30355869 |
| rs10123659 | 9 | 83235735 | G | T | 0.138786 | 0.0126909 | 0.00188947 | 1.9002E-11 | -0.0221 | 0.0134 | 0.1 | 9.76489E-05 | 45.11313023 |
| rs10129429 | 14 | 92430831 | A | G | 0.423775 | -0.0217387 | 0.00132933 | 4.10015E-60 | -0.0099 | 0.0094 | 0.2932 | 0.000578569 | 267.4235479 |
| rs10131337 | 14 | 37144516 | T | C | 0.244243 | 0.0144346 | 0.00153474 | 5.19996E-21 | -0.0256 | 0.0108 | 0.0180701 | 0.000191453 | 88.45818053 |
| rs1013495 | 10 | 81140253 | T | C | 0.457301 | -0.0214739 | 0.00131482 | 5.79963E-60 | -0.0106 | 0.0093 | 0.2567 | 0.00057709 | 266.7395224 |
| rs10139746 | 14 | 75040338 | G | A | 0.54866 | -0.0149366 | 0.00131883 | 9.79941E-30 | 0.0013 | 0.0093 | 0.8876 | 0.000277594 | 128.2696725 |
| rs10155941 | 7 | 134422732 | C | T | 0.460683 | -0.0116492 | 0.0013099 | 5.90065E-19 | -0.0101 | 0.0093 | 0.2794 | 0.000171177 | 79.08862744 |
| rs10165255 | 2 | 10199601 | G | A | 0.558397 | -0.0163525 | 0.00131152 | 1.10002E-35 | -0.0084 | 0.0094 | 0.3678 | 0.000336417 | 155.4592613 |
| rs10181220 | 2 | 20035567 | T | C | 0.408776 | -0.00841115 | 0.00131869 | 1.79999E-10 | 0.0085 | 0.0094 | 0.3696 | 8.80627E-05 | 40.68398168 |
| rs1019075 | 14 | 73884047 | T | C | 0.450002 | -0.00859973 | 0.00131969 | 7.19946E-11 | 0.0641 | 0.0772 | 0.4058 | 9.1916E-05 | 42.46429326 |
| rs1019075 | 14 | 73884047 | T | C | 0.450002 | -0.00859973 | 0.00131969 | 7.19946E-11 | 0.0035 | 0.0093 | 0.704301 | 9.1916E-05 | 42.46429326 |
| rs1020048 | 9 | 86666993 | C | A | 0.809093 | -0.0174125 | 0.00168128 | 3.90032E-25 | 0.0042 | 0.0119 | 0.7252 | 0.000232138 | 107.2606156 |
| rs10202701 | 2 | 232328681 | T | C | 0.541621 | 0.0186846 | 0.00130659 | 2.19989E-46 | 0.001 | 0.0093 | 0.9178 | 0.000442488 | 204.4970604 |
| rs10207736 | 2 | 217863554 | T | A | 0.31377 | 0.0126352 | 0.0014024 | 2.09991E-19 | 0.0054 | 0.01 | 0.5874 | 0.000175691 | 81.1743005 |
| rs10221274 | 17 | 68093498 | T | G | 0.403015 | -0.011654 | 0.00135112 | 6.4003E-18 | -0.0002 | 0.0096 | 0.9825 | 0.000161026 | 74.39783573 |
| rs10228350 | 7 | 114060663 | T | A | 0.424502 | -0.00995109 | 0.0013279 | 6.70039E-14 | 0.0167 | 0.0094 | 0.07703 | 0.000121552 | 56.15761901 |
| rs10233251 | 7 | 46211533 | G | A | 0.119342 | 0.0201774 | 0.00202269 | 1.99986E-23 | 0.0173 | 0.0144 | 0.2291 | 0.000215369 | 99.51072084 |
| rs10241336 | 7 | 19610215 | C | T | 0.631084 | -0.0147019 | 0.00135147 | 1.50003E-27 | 0.0068 | 0.0096 | 0.4798 | 0.000256111 | 118.340181 |
| rs10248846 | 7 | 101733647 | A | G | 0.060807 | -0.0162727 | 0.002733 | 2.59998E-09 | -0.0098 | 0.0194 | 0.6144 | 7.67382E-05 | 35.45178381 |
| rs10253161 | 7 | 46996392 | G | A | 0.29267 | -0.00819728 | 0.00143497 | 1.09999E-08 | -0.0083 | 0.0102 | 0.4132 | 7.06363E-05 | 32.63262544 |
| rs10283100 | 8 | 120596023 | G | A | 0.9445 | 0.0314181 | 0.00285863 | 4.19952E-28 | -0.013 | 0.0203 | 0.5221 | 0.000261418 | 120.7930816 |
| rs10401891 | 19 | 4962848 | T | C | 0.325542 | -0.0201602 | 0.0014045 | 1E-46 | -0.0126 | 0.0099 | 0.2041 | 0.000445818 | 206.036567 |
| rs1042838 | 11 | 100933412 | A | C | 0.165796 | -0.00999404 | 0.00175926 | 1.29999E-08 | -0.0379 | 0.0125 | 0.002452 | 6.98549E-05 | 32.27158007 |
| rs1043413 | 19 | 41939297 | G | C | 0.388615 | 0.0191614 | 0.00134541 | 5.00035E-46 | 0.0174 | 0.0095 | 0.0664492 | 0.000438894 | 202.8351843 |
| rs1043463 | 2 | 32843295 | T | A | 0.632507 | -0.00930946 | 0.00148502 | 3.59998E-10 | -0.0029 | 0.0105 | 0.7843 | 8.50653E-05 | 39.29909007 |
| rs1043547 | 14 | 93406789 | T | G | 0.25062 | -0.0119203 | 0.00153287 | 7.50067E-15 | -0.0078 | 0.0108 | 0.4715 | 0.000130892 | 60.47304558 |
| rs1044299 | 1 | 176811873 | T | C | 0.546034 | 0.0214915 | 0.00130623 | 8.00018E-61 | 0.011 | 0.0093 | 0.24 | 0.000585659 | 270.702501 |
| rs10498672 | 6 | 7797840 | G | C | 0.175595 | 0.0201025 | 0.00170145 | 3.29989E-32 | 0.0087 | 0.0122 | 0.4739 | 0.000302089 | 139.5917909 |
| rs1050969 | 8 | 103661730 | C | T | 0.017473 | 0.031159 | 0.00515171 | 1.5E-09 | -0.0234 | 0.0362 | 0.517399 | 7.91835E-05 | 36.58156884 |
| rs1051547 | 16 | 19279380 | C | T | 0.561563 | -0.00962221 | 0.00133294 | 5.19996E-13 | 0.0075 | 0.0094 | 0.4237 | 0.000112794 | 52.11066075 |
| rs1052256 | 1 | 172363488 | G | A | 0.187861 | 0.0193679 | 0.00165913 | 1.69981E-31 | 0.004 | 0.0118 | 0.734301 | 0.000294904 | 136.2706588 |
| rs1056173 | 7 | 5364041 | A | G | 0.113107 | -0.0176427 | 0.00207092 | 1.59993E-17 | 0.0003 | 0.0147 | 0.9858 | 0.000157087 | 72.57742802 |
| rs1065368 | 16 | 1036860 | C | T | 0.471824 | 0.0127506 | 0.00134749 | 2.99985E-21 | 0.0304 | 0.0095 | 0.001373 | 0.00019379 | 89.53817977 |
| rs1074683 | 20 | 32304653 | G | C | 0.26262 | -0.0300431 | 0.00149114 | 2.80027E-90 | 0.0068 | 0.0106 | 0.52 | 0.000877963 | 405.929642 |
| rs10748128 | 12 | 69827658 | T | G | 0.344563 | 0.0257921 | 0.0013728 | 9.49948E-79 | 0.0298 | 0.0098 | 0.00223501 | 0.000763541 | 352.9855983 |
| rs10756792 | 9 | 16726119 | T | C | 0.742902 | 0.0134419 | 0.00150166 | 3.50026E-19 | -0.0332 | 0.0107 | 0.00187301 | 0.000173423 | 80.12650779 |
| rs10770705 | 12 | 20857467 | C | A | 0.66273 | -0.0152113 | 0.00137879 | 2.70023E-28 | -0.0226 | 0.0098 | 0.02123 | 0.000263407 | 121.7123179 |
| rs10773083 | 12 | 124791650 | T | G | 0.645243 | -0.0167543 | 0.00139192 | 2.29985E-33 | -0.0202 | 0.0099 | 0.0412098 | 0.00031354 | 144.8845728 |
| rs10775348 | 16 | 88806348 | G | A | 0.703719 | 0.0189637 | 0.00144064 | 1.39991E-39 | 0.0368 | 0.0102 | 0.000299199 | 0.000374953 | 173.2739734 |
| rs10789401 | 1 | 42081111 | G | A | 0.676322 | -0.00996766 | 0.00138799 | 6.89922E-13 | 0.0027 | 0.0099 | 0.7879 | 0.000111627 | 51.57175094 |
| rs10811092 | 9 | 19002386 | A | T | 0.561838 | -0.00791964 | 0.0013272 | 2.39999E-09 | 0.0238 | 0.0094 | 0.0114501 | 7.70743E-05 | 35.60707134 |
| rs10826579 | 10 | 29491536 | A | G | 0.152964 | -0.0137741 | 0.00185607 | 1.20005E-13 | -0.006 | 0.0131 | 0.6446 | 0.000119204 | 55.07263809 |
| rs10831284 | 11 | 94667964 | A | G | 0.870718 | -0.0121675 | 0.00194698 | 4.09996E-10 | -0.0001 | 0.0138 | 0.9922 | 8.45372E-05 | 39.05510852 |
| rs10832961 | 11 | 18653957 | G | C | 0.758154 | -0.015509 | 0.00152973 | 3.69999E-24 | 0.0059 | 0.0108 | 0.5836 | 0.000222457 | 102.7865156 |
| rs10840242 | 11 | 9474382 | C | T | 0.503893 | 0.00954039 | 0.00131179 | 3.50026E-13 | 0.0043 | 0.0093 | 0.6417 | 0.000114488 | 52.89343173 |
| rs10861678 | 12 | 107319661 | A | G | 0.265789 | -0.015574 | 0.00147677 | 5.30029E-26 | 0.0059 | 0.0105 | 0.5739 | 0.000240699 | 111.2174 |
| rs10874746 | 1 | 93323971 | C | T | 0.656573 | 0.0157216 | 0.00136413 | 9.8992E-31 | 0.0117 | 0.0098 | 0.2305 | 0.00028745 | 132.8250795 |
| rs10901216 | 9 | 133471891 | A | G | 0.347973 | -0.0218278 | 0.00137391 | 7.70016E-57 | 0.0099 | 0.0098 | 0.3114 | 0.000546099 | 252.4070507 |
| rs10916606 | 1 | 224570425 | T | C | 0.787861 | 0.0165895 | 0.00158945 | 1.69981E-25 | 0.0044 | 0.0114 | 0.696101 | 0.000235763 | 108.9358865 |
| rs10922478 | 1 | 89144053 | G | A | 0.565479 | 0.0189841 | 0.00130635 | 7.59976E-48 | 0.012 | 0.0093 | 0.1999 | 0.000456949 | 211.1830444 |
| rs10923724 | 1 | 119546842 | T | C | 0.568101 | 0.00948975 | 0.00131261 | 4.79954E-13 | -0.0224 | 0.0094 | 0.0169301 | 0.000113134 | 52.26804369 |
| rs10943915 | 6 | 83617970 | T | A | 0.297278 | 0.0106931 | 0.00142534 | 6.29941E-14 | -0.0012 | 0.0102 | 0.908 | 0.000121821 | 56.28185851 |
| rs10945540 | 6 | 158738975 | A | G | 0.664795 | -0.0148538 | 0.0013739 | 2.99985E-27 | -0.0055 | 0.0098 | 0.572499 | 0.000252965 | 116.88614 |
| rs10948 | 19 | 10754905 | T | G | 0.663596 | -0.0174758 | 0.00139001 | 2.99985E-36 | -0.0415 | 0.0098 | 2.34698E-05 | 0.000342054 | 158.0652498 |
| rs10953083 | 7 | 92657034 | A | C | 0.445753 | 0.0102163 | 0.00131805 | 9.09913E-15 | 0.0012 | 0.0094 | 0.8947 | 0.000130039 | 60.07885175 |
| rs10980706 | 9 | 113803627 | T | G | 0.243752 | -0.0160075 | 0.00152561 | 9.30037E-26 | -0.0167 | 0.0108 | 0.1225 | 0.000238266 | 110.0925896 |
| rs10989088 | 9 | 98823407 | A | G | 0.281323 | 0.00984569 | 0.00145673 | 1.39991E-11 | 0.0049 | 0.0103 | 0.6357 | 9.88772E-05 | 45.6806533 |
| rs10995319 | 10 | 52762887 | C | T | 0.234453 | -0.0140418 | 0.00154754 | 1.20005E-19 | 0.027 | 0.011 | 0.0136399 | 0.000178193 | 82.33034195 |
| rs11001271 | 10 | 76822260 | A | G | 0.25369 | 0.00832546 | 0.00150599 | 0.000000032 | 0.0203 | 0.0107 | 0.05719 | 6.61529E-05 | 30.56120124 |
| rs11049708 | 12 | 28697814 | C | T | 0.292525 | -0.02959 | 0.0014357 | 2.19989E-94 | -0.0333 | 0.0102 | 0.00109999 | 0.000918689 | 424.7766635 |
| rs11051456 | 12 | 31700912 | T | C | 0.729874 | -0.0100846 | 0.00146932 | 6.70039E-12 | -0.0031 | 0.0702 | 0.9651 | 0.000101964 | 47.10670128 |
| rs11051456 | 12 | 31700912 | T | C | 0.729874 | -0.0100846 | 0.00146932 | 6.70039E-12 | -0.009 | 0.0105 | 0.3912 | 0.000101964 | 47.10670128 |
| rs11058226 | 12 | 122938958 | C | T | 0.742115 | 0.0160921 | 0.00149085 | 3.69999E-27 | 0.0278 | 0.0106 | 0.00872891 | 0.000252147 | 116.5079782 |
| rs11088114 | 21 | 30612396 | A | C | 0.10653 | 0.0133054 | 0.00215385 | 6.49995E-10 | 0.0132 | 0.0152 | 0.3824 | 8.26027E-05 | 38.16129299 |
| rs11122824 | 2 | 121591475 | T | A | 0.816544 | 0.012421 | 0.00168823 | 1.9002E-13 | -0.0103 | 0.0121 | 0.3975 | 0.000117167 | 54.13124507 |
| rs11129108 | 3 | 23179483 | C | T | 0.610254 | 0.00750992 | 0.00134127 | 2.19999E-08 | 0.0008 | 0.0096 | 0.9313 | 6.786E-05 | 31.34991151 |
| rs11141738 | 9 | 89910210 | T | C | 0.232993 | -0.0124312 | 0.00154544 | 8.69961E-16 | 0.0242 | 0.0109 | 0.0272201 | 0.000140044 | 64.70233138 |
| rs11144782 | 9 | 78795213 | G | C | 0.174583 | -0.0118348 | 0.00172767 | 7.39946E-12 | -0.0311 | 0.0122 | 0.01096 | 0.000101569 | 46.92436257 |
| rs11152363 | 18 | 53057188 | A | G | 0.18638 | 0.011541 | 0.00170482 | 1.29987E-11 | 0.0068 | 0.012 | 0.5706 | 9.91955E-05 | 45.82768703 |
| rs11167487 | 5 | 149301335 | C | T | 0.156187 | 0.0123317 | 0.00179552 | 6.4998E-12 | 0.0038 | 0.0128 | 0.7681 | 0.0001021 | 47.16974489 |
| rs111738917 | 4 | 106240476 | A | G | 0.026181 | -0.0288115 | 0.00408121 | 1.69981E-12 | -0.0282 | 0.0289 | 0.3294 | 0.000107873 | 49.83700948 |
| rs11196169 | 10 | 114721237 | G | A | 0.527333 | -0.00785155 | 0.00133834 | 4.39997E-09 | 0.0088 | 0.0095 | 0.3526 | 7.4499E-05 | 34.41723717 |
| rs11198898 | 10 | 121118492 | C | A | 0.763223 | -0.0202846 | 0.00154441 | 2.09991E-39 | -0.022 | 0.0109 | 0.0436496 | 0.000373294 | 172.5066359 |
| rs11205303 | 1 | 149906413 | C | T | 0.406554 | 0.0343588 | 0.00132152 | 5.0004E-149 | -0.0095 | 0.0099 | 0.34 | 0.001461162 | 675.9687427 |
| rs11209173 | 1 | 68369950 | A | G | 0.275705 | -0.00829042 | 0.00145688 | 1.29999E-08 | 0.0004 | 0.0104 | 0.9728 | 7.00939E-05 | 32.38199659 |
| rs112153300 | 21 | 47547474 | A | G | 0.08903 | 0.0241257 | 0.00231852 | 2.29985E-25 | -0.0322 | 0.0163 | 0.0481604 | 0.000234337 | 108.2770146 |
| rs11217863 | 11 | 120293138 | A | G | 0.116091 | -0.0222611 | 0.00204458 | 1.29987E-27 | 0.0195 | 0.0145 | 0.1793 | 0.000256554 | 118.5449736 |
| rs11221158 | 11 | 127980209 | T | C | 0.225284 | -0.0120672 | 0.00156702 | 1.39991E-14 | 0.0136 | 0.0111 | 0.221 | 0.000128355 | 59.30100619 |
| rs11245333 | 10 | 126383088 | A | C | 0.404943 | 0.0154443 | 0.00133681 | 7.10068E-31 | 0.0018 | 0.0095 | 0.851 | 0.000288853 | 133.4735485 |
| rs112471207 | 2 | 172415930 | T | C | 0.246621 | -0.0158455 | 0.00150518 | 6.4998E-26 | -0.0081 | 0.0107 | 0.4486 | 0.000239848 | 110.8238255 |
| rs11252860 | 10 | 5024356 | C | A | 0.74338 | 0.0139321 | 0.00154514 | 1.9002E-19 | 0.0218 | 0.1475 | 0.8824 | 0.000175965 | 81.30094895 |
| rs11252860 | 10 | 5024356 | C | A | 0.74338 | 0.0139321 | 0.00154514 | 1.9002E-19 | -0.0088 | 0.0109 | 0.4206 | 0.000175965 | 81.30094895 |
| rs11259936 | 15 | 84580582 | C | A | 0.522781 | 0.0357708 | 0.00130775 | 1E-164 | -0.0191 | 0.0093 | 0.0393604 | 0.001617 | 748.1797827 |
| rs112635811 | 11 | 1930713 | T | C | 0.084468 | -0.0142212 | 0.00244067 | 5.69994E-09 | 0.007 | 0.0172 | 0.6829 | 7.34899E-05 | 33.95099308 |
| rs113595815 | 15 | 86071450 | G | T | 0.052292 | -0.0200277 | 0.00293164 | 8.4004E-12 | -0.0213 | 0.0208 | 0.3057 | 0.000101019 | 46.67012855 |
| rs113851505 | 16 | 341988 | T | C | 0.183974 | -0.00927822 | 0.00169888 | 4.70002E-08 | 0.0025 | 0.012 | 0.8332 | 6.45626E-05 | 29.82648287 |
| rs11466399 | 1 | 218591623 | A | G | 0.286927 | 0.0275801 | 0.00143604 | 3.29989E-82 | 0.001 | 0.0103 | 0.9206 | 0.000797842 | 368.8558864 |
| rs114784809 | 5 | 127674221 | A | G | 0.025391 | 0.0313514 | 0.00418669 | 7.00003E-14 | 0.0119 | 0.0295 | 0.6875 | 0.000121374 | 56.07514273 |
| rs11553764 | 12 | 104415244 | T | C | 0.169703 | 0.0124731 | 0.00174189 | 8.00018E-13 | -0.0008 | 0.0124 | 0.9477 | 0.000110985 | 51.27497724 |
| rs116211567 | 2 | 68425404 | C | T | 0.080909 | -0.0239078 | 0.00238485 | 1.20005E-23 | 0.0111 | 0.017 | 0.514 | 0.000217504 | 100.4974891 |
| rs11664336 | 18 | 46604851 | T | A | 0.434569 | 0.0253684 | 0.00132728 | 1.99986E-81 | -0.04 | 0.0094 | 0.00001986 | 0.000790174 | 365.3079914 |
| rs11671106 | 19 | 46367025 | T | C | 0.354434 | -0.00829841 | 0.00137367 | 1.5E-09 | -0.0019 | 0.0097 | 0.8443 | 7.89943E-05 | 36.49413455 |
| rs11678288 | 2 | 65774687 | A | T | 0.588393 | -0.0101942 | 0.00132554 | 1.50003E-14 | -0.0077 | 0.0095 | 0.4154 | 0.000128018 | 59.14509638 |
| rs11681299 | 2 | 88901732 | T | C | 0.286715 | 0.0224663 | 0.00143297 | 2.09991E-55 | 0.0033 | 0.0102 | 0.7493 | 0.000531818 | 245.8029161 |
| rs116893322 | 15 | 85524628 | A | G | 0.147805 | 0.016379 | 0.0018393 | 5.30029E-19 | -0.0142 | 0.0131 | 0.279 | 0.000171633 | 79.29900606 |
| rs11690176 | 2 | 242356887 | A | G | 0.317904 | -0.0111347 | 0.00139315 | 1.29987E-15 | -0.0054 | 0.01 | 0.5909 | 0.000138263 | 63.87919021 |
| rs1169738 | 12 | 121584410 | A | G | 0.031918 | 0.0216198 | 0.00371777 | 6.1E-09 | 0.0485 | 0.0263 | 0.0658597 | 7.32001E-05 | 33.81710505 |
| rs11708067 | 3 | 123065778 | G | A | 0.242372 | -0.013468 | 0.00151846 | 7.29962E-19 | 0.0134 | 0.0108 | 0.2159 | 0.000170267 | 78.66791197 |
| rs11708810 | 3 | 55500045 | A | T | 0.25078 | 0.0143591 | 0.00151392 | 2.39994E-21 | 0.0082 | 0.0108 | 0.4454 | 0.000194701 | 89.9594325 |
| rs11710894 | 3 | 112991959 | T | C | 0.168578 | -0.0121378 | 0.00174268 | 3.29989E-12 | -0.0061 | 0.0124 | 0.6259 | 0.000105004 | 48.51128515 |
| rs11711770 | 3 | 43066768 | G | A | 0.1709 | -0.0142608 | 0.00173242 | 1.80011E-16 | -0.009 | 0.0123 | 0.4673 | 0.000146664 | 67.76095536 |
| rs11713042 | 3 | 41288004 | C | T | 0.473783 | 0.0123825 | 0.00130505 | 2.39994E-21 | 0.021 | 0.0093 | 0.02362 | 0.000194842 | 90.02445297 |
| rs1171614 | 10 | 61469538 | C | T | 0.769106 | -0.0186864 | 0.00155615 | 3.19963E-33 | 0.0028 | 0.011 | 0.7961 | 0.000312045 | 144.193767 |
| rs11720869 | 3 | 185619716 | A | G | 0.668249 | 0.00947484 | 0.00139009 | 9.3994E-12 | -0.0172 | 0.0099 | 0.0828705 | 0.000100559 | 46.45752306 |
| rs11722554 | 4 | 5016883 | A | G | 0.037916 | -0.0319218 | 0.00341099 | 8.10028E-21 | -0.0368 | 0.0244 | 0.1317 | 0.000189556 | 87.5814179 |
| rs11741562 | 5 | 79893662 | G | T | 0.278405 | 0.00822685 | 0.00147084 | 2.19999E-08 | -0.0029 | 0.0105 | 0.780899 | 6.77192E-05 | 31.28487422 |
| rs11743919 | 5 | 156754251 | T | C | 0.213761 | -0.011314 | 0.00158816 | 1E-12 | -0.0123 | 0.0113 | 0.2765 | 0.00010985 | 50.7506912 |
| rs11746047 | 5 | 123997308 | T | C | 0.379838 | 0.0114831 | 0.00134815 | 1.59993E-17 | 0.0182 | 0.0096 | 0.0579202 | 0.000157029 | 72.55042843 |
| rs11747997 | 5 | 142790752 | C | A | 0.328764 | 0.0115007 | 0.00139452 | 1.59993E-16 | -0.0026 | 0.0099 | 0.7926 | 0.000147211 | 68.01382114 |
| rs11754773 | 6 | 34577257 | G | A | 0.093353 | 0.0426973 | 0.00224063 | 5.90065E-81 | 0.0466 | 0.016 | 0.00350703 | 0.00078546 | 363.1270927 |
| rs11774206 | 8 | 144375815 | C | T | 0.764036 | -0.015328 | 0.00153946 | 2.39994E-23 | -0.0026 | 0.0109 | 0.8098 | 0.000214559 | 99.13620392 |
| rs11775903 | 8 | 25321076 | G | A | 0.399545 | 0.00918727 | 0.00133411 | 5.70033E-12 | 0.0047 | 0.0095 | 0.6204 | 0.000102648 | 47.42286637 |
| rs117793215 | 15 | 100535681 | T | C | 0.035384 | 0.0277968 | 0.00370544 | 6.29941E-14 | -0.0102 | 0.0262 | 0.6983 | 0.000121804 | 56.2740476 |
| rs1178172 | 7 | 18794541 | C | T | 0.212634 | 0.0152921 | 0.00159485 | 8.9002E-22 | 0.009 | 0.0113 | 0.4274 | 0.000198982 | 91.93750061 |
| rs117829095 | 7 | 1932374 | A | C | 0.077904 | 0.015465 | 0.00248074 | 4.49997E-10 | -0.0371 | 0.0177 | 0.0357199 | 8.41213E-05 | 38.86292368 |
| rs11783598 | 8 | 4798388 | C | T | 0.53242 | 0.008088 | 0.00131526 | 7.79992E-10 | -0.0222 | 0.0093 | 0.01739 | 8.18519E-05 | 37.81439705 |
| rs1179947 | 12 | 122442444 | A | G | 0.782563 | -0.00973182 | 0.00158282 | 7.79992E-10 | 0.0057 | 0.0113 | 0.612099 | 8.18266E-05 | 37.80273279 |
| rs118173451 | 22 | 28356600 | C | T | 0.016152 | -0.0545347 | 0.00530716 | 9.09913E-25 | -0.0285 | 0.0374 | 0.4457 | 0.000228522 | 105.589228 |
| rs11857726 | 15 | 41530359 | A | G | 0.379428 | -0.0138048 | 0.00134912 | 1.39991E-24 | 0.0022 | 0.0096 | 0.8168 | 0.000226603 | 104.702535 |
| rs11877685 | 18 | 41880210 | A | G | 0.155786 | -0.0115027 | 0.00183117 | 3.40001E-10 | -0.0048 | 0.0129 | 0.7089 | 8.54103E-05 | 39.45848228 |
| rs11898716 | 2 | 241828311 | G | A | 0.708074 | 0.0108291 | 0.00143669 | 4.79954E-14 | 0.0212 | 0.0103 | 0.0398098 | 0.000122973 | 56.81417574 |
| rs11947952 | 4 | 144252964 | G | C | 0.63615 | 0.00852434 | 0.00135555 | 3.2E-10 | 0.0136 | 0.0097 | 0.1592 | 8.5597E-05 | 39.5447254 |
| rs11955153 | 5 | 170864548 | C | T | 0.235789 | -0.0232208 | 0.00153826 | 1.69981E-51 | -0.0218 | 0.011 | 0.0469797 | 0.000493044 | 227.8730585 |
| rs11957435 | 5 | 122657858 | C | T | 0.209912 | -0.0121185 | 0.001616 | 6.4003E-14 | 0.0165 | 0.0115 | 0.1507 | 0.000121721 | 56.23583412 |
| rs11998884 | 9 | 33684436 | T | C | 0.06181 | 0.0188111 | 0.00274739 | 7.50067E-12 | -0.025 | 0.0194 | 0.198 | 0.000101473 | 46.87981376 |
| rs12051048 | 16 | 783864 | A | C | 0.231912 | 0.026557 | 0.00155556 | 2.39994E-65 | -0.0088 | 0.011 | 0.4226 | 0.000630544 | 291.462451 |
| rs12055151 | 5 | 50418925 | A | T | 0.656867 | -0.00910456 | 0.00137766 | 3.90032E-11 | 0.0136 | 0.0098 | 0.1665 | 9.45361E-05 | 43.67490273 |
| rs12103006 | 16 | 24726237 | G | A | 0.569082 | 0.0120475 | 0.00132582 | 1E-19 | 0.0066 | 0.0094 | 0.4809 | 0.000178711 | 82.57010893 |
| rs12126112 | 1 | 85730595 | T | C | 0.207655 | -0.00910344 | 0.00159907 | 0.000000012 | -0.0187 | 0.0114 | 0.1021 | 7.01537E-05 | 32.40964223 |
| rs12130046 | 1 | 67444043 | A | G | 0.148513 | -0.0168305 | 0.00183173 | 4.00037E-20 | -0.0098 | 0.013 | 0.454 | 0.000182725 | 84.42464428 |
| rs12130750 | 1 | 200208417 | C | T | 0.409005 | 0.0088774 | 0.00132347 | 1.99986E-11 | 0.0002 | 0.0095 | 0.9858 | 9.73881E-05 | 44.99264188 |
| rs12132534 | 1 | 147098986 | G | C | 0.142706 | 0.0107798 | 0.00185464 | 6.19998E-09 | -0.0135 | 0.0133 | 0.3099 | 7.31266E-05 | 33.78316093 |
| rs12148447 | 15 | 38366667 | T | C | 0.032582 | -0.0245162 | 0.00378919 | 9.79941E-11 | -0.0319 | 0.0266 | 0.2311 | 9.06106E-05 | 41.86119925 |
| rs12185993 | 3 | 188565410 | A | T | 0.22679 | 0.0102095 | 0.00156191 | 6.29941E-11 | -0.0062 | 0.0111 | 0.578801 | 9.2483E-05 | 42.7262774 |
| rs12209223 | 6 | 76164589 | A | C | 0.10134 | 0.0313272 | 0.00215756 | 9.09913E-48 | -0.0044 | 0.0154 | 0.774599 | 0.000456167 | 210.8217992 |
| rs12342779 | 9 | 119122437 | G | T | 0.201908 | -0.0308688 | 0.00162959 | 5.10035E-80 | -0.005 | 0.0115 | 0.6635 | 0.000776159 | 358.8235279 |
| rs12348423 | 9 | 4740251 | C | A | 0.250398 | -0.0131373 | 0.00150753 | 2.90001E-18 | 0.0069 | 0.0107 | 0.5217 | 0.000164367 | 75.94137004 |
| rs12362444 | 11 | 45278317 | A | G | 0.55602 | -0.00855126 | 0.00131951 | 9.09913E-11 | 0.0236 | 0.0094 | 0.0116399 | 9.09076E-05 | 41.99842176 |
| rs12406530 | 1 | 113117213 | G | A | 0.206441 | -0.016474 | 0.00160661 | 1.10002E-24 | -0.0065 | 0.0115 | 0.5699 | 0.000227553 | 105.1417767 |
| rs12452505 | 17 | 63556402 | G | C | 0.142356 | -0.0298046 | 0.00187781 | 9.8992E-57 | -0.0029 | 0.0133 | 0.8271 | 0.000545044 | 251.9192879 |
| rs12452590 | 17 | 60720058 | G | T | 0.361744 | 0.0129203 | 0.00137636 | 6.20012E-21 | 0.0132 | 0.0097 | 0.1755 | 0.000190723 | 88.1209621 |
| rs12459155 | 19 | 4774475 | G | C | 0.130694 | -0.0135334 | 0.00195153 | 4.10015E-12 | 0.0142 | 0.0138 | 0.3048 | 0.000104093 | 48.09074361 |
| rs12466227 | 2 | 60148940 | G | A | 0.548468 | -0.0102371 | 0.00130612 | 4.60045E-15 | 0.0119 | 0.0093 | 0.2033 | 0.000132964 | 61.43075483 |
| rs12478285 | 2 | 109051753 | A | G | 0.426287 | 0.00801252 | 0.00131266 | 0.000000001 | 0.0008 | 0.0094 | 0.9329 | 8.06499E-05 | 37.25905794 |
| rs12483653 | 21 | 17355049 | T | C | 0.78055 | -0.0129415 | 0.00160957 | 8.99912E-16 | -0.0146 | 0.0113 | 0.1954 | 0.000139924 | 64.6468868 |
| rs12497944 | 3 | 24044111 | A | G | 0.250397 | -0.0110169 | 0.00150698 | 2.70023E-13 | 0.0122 | 0.0107 | 0.2568 | 0.00011568 | 53.44436942 |
| rs12517974 | 5 | 137744369 | C | A | 0.173538 | 0.010044 | 0.00171827 | 5.1E-09 | -0.0068 | 0.0123 | 0.5798 | 7.39611E-05 | 34.16872021 |
| rs12552167 | 9 | 139322927 | T | C | 0.284014 | 0.0175781 | 0.00144744 | 6.20012E-34 | 0.0023 | 0.0103 | 0.8247 | 0.00031916 | 147.4826189 |
| rs12572775 | 10 | 104285594 | T | A | 0.558583 | 0.0207436 | 0.00131718 | 7.00003E-56 | 0.0048 | 0.0093 | 0.6051 | 0.000536599 | 248.0139578 |
| rs12584892 | 13 | 73624534 | T | C | 0.178344 | -0.0130725 | 0.00172305 | 3.29989E-14 | 0.0018 | 0.0122 | 0.8832 | 0.000124587 | 57.55985093 |
| rs12586289 | 14 | 55262780 | T | G | 0.56986 | 0.0126157 | 0.00132618 | 1.9002E-21 | -0.0028 | 0.0094 | 0.7692 | 0.000195856 | 90.49318575 |
| rs12592845 | 15 | 48684958 | T | C | 0.089884 | -0.0251655 | 0.00228515 | 3.29989E-28 | 0.0368 | 0.0162 | 0.0229298 | 0.000262466 | 121.2773841 |
| rs12622270 | 2 | 164449314 | C | T | 0.167122 | -0.0104133 | 0.00173992 | 2.19999E-09 | 0.0165 | 0.0125 | 0.1863 | 7.75335E-05 | 35.81923612 |
| rs12645070 | 4 | 57770106 | A | G | 0.183238 | 0.0242564 | 0.00168398 | 4.90004E-47 | 0.02 | 0.012 | 0.0952994 | 0.000448941 | 207.4803408 |
| rs12648093 | 4 | 123838758 | G | A | 0.745477 | -0.0136056 | 0.0014965 | 9.79941E-20 | -0.0087 | 0.0107 | 0.4176 | 0.0001789 | 82.657083 |
| rs12668269 | 7 | 65779510 | T | A | 0.278129 | 0.00998126 | 0.00145718 | 7.39946E-12 | 0.0237 | 0.0103 | 0.0222198 | 0.000101556 | 46.91832002 |
| rs12669267 | 7 | 73304636 | T | C | 0.133088 | -0.0176304 | 0.00194562 | 1.29987E-19 | -0.0194 | 0.0138 | 0.1601 | 0.00017772 | 82.11195782 |
| rs12813930 | 12 | 120950713 | C | T | 0.203731 | -0.0126495 | 0.00161763 | 5.30029E-15 | -0.0021 | 0.0115 | 0.8541 | 0.000132354 | 61.14859085 |
| rs12868316 | 13 | 113878523 | T | C | 0.225036 | 0.00931154 | 0.00157269 | 3.2E-09 | -0.0171 | 0.0111 | 0.1238 | 7.58803E-05 | 35.05539746 |
| rs12903939 | 15 | 101641034 | A | G | 0.296162 | -0.0114684 | 0.00162458 | 1.69981E-12 | 0.0107 | 0.0114 | 0.3478 | 0.000107865 | 49.83352165 |
| rs1291066 | 20 | 35790918 | C | G | 0.808481 | 0.0157438 | 0.00166635 | 3.50026E-21 | 0.0215 | 0.0118 | 0.0688098 | 0.0001932 | 89.26573727 |
| rs12924101 | 16 | 89862906 | C | A | 0.186072 | -0.0133876 | 0.00168415 | 1.9002E-15 | 0.0023 | 0.0119 | 0.8498 | 0.00013677 | 63.18908056 |
| rs12952981 | 17 | 79409189 | A | G | 0.355344 | 0.0122798 | 0.00136916 | 2.99985E-19 | 0.0177 | 0.0097 | 0.0676597 | 0.000174102 | 80.44004435 |
| rs12966785 | 18 | 22858941 | A | G | 0.381975 | -0.0116103 | 0.00135353 | 9.70063E-18 | 0.0029 | 0.0095 | 0.765301 | 0.000159253 | 73.57821218 |
| rs12968652 | 18 | 46501070 | A | G | 0.398207 | -0.014194 | 0.00135828 | 1.50003E-25 | -0.0184 | 0.0096 | 0.05424 | 0.000236338 | 109.2016271 |
| rs12982509 | 19 | 3412057 | C | T | 0.220759 | 0.0181866 | 0.00158573 | 1.9002E-30 | -0.0061 | 0.0112 | 0.5822 | 0.00028466 | 131.5354044 |
| rs12985850 | 19 | 2155042 | A | G | 0.401088 | 0.0250589 | 0.00134598 | 2.29985E-77 | 0.0205 | 0.0095 | 0.0310099 | 0.000749767 | 346.613052 |
| rs13014796 | 2 | 200321050 | A | G | 0.169176 | -0.0170775 | 0.00173772 | 8.60003E-23 | 0.0061 | 0.0124 | 0.6259 | 0.000209027 | 96.57997943 |
| rs13041213 | 20 | 47800829 | A | T | 0.235434 | 0.0247857 | 0.00155506 | 3.40017E-57 | 0.0007 | 0.011 | 0.9508 | 0.000549635 | 254.0423939 |
| rs13102005 | 4 | 152212704 | A | T | 0.438157 | 0.0076448 | 0.00131392 | 5.89997E-09 | -0.0182 | 0.0094 | 0.0511599 | 7.3277E-05 | 33.85263894 |
| rs13168903 | 5 | 158517233 | G | T | 0.414828 | -0.00800838 | 0.00132421 | 1.5E-09 | 0.0091 | 0.0094 | 0.3331 | 7.91674E-05 | 36.57410758 |
| rs1317867 | 17 | 21276114 | G | A | 0.511926 | 0.0185562 | 0.00131971 | 6.59933E-45 | 0.0036 | 0.0094 | 0.6967 | 0.000427799 | 197.7057016 |
| rs13178887 | 5 | 88355993 | C | T | 0.386875 | -0.0200087 | 0.00133714 | 1.29987E-50 | -0.0336 | 0.0095 | 0.000414801 | 0.000484483 | 223.914441 |
| rs13179048 | 5 | 95542726 | A | C | 0.306872 | 0.0122048 | 0.00141224 | 5.50047E-18 | 0.0238 | 0.01 | 0.0177399 | 0.000161651 | 74.68655669 |
| rs13226864 | 7 | 100499315 | A | G | 0.183354 | 0.0116434 | 0.00168352 | 4.60045E-12 | -0.0141 | 0.012 | 0.2386 | 0.000103534 | 47.83229233 |
| rs13251736 | 8 | 32291790 | C | T | 0.0507 | -0.0183806 | 0.00300147 | 9.09997E-10 | -0.0164 | 0.0213 | 0.4421 | 8.11748E-05 | 37.50157208 |
| rs13334364 | 16 | 67332365 | C | T | 0.075089 | -0.0254216 | 0.00249115 | 1.9002E-24 | 0.0364 | 0.0177 | 0.0400904 | 0.000225379 | 104.1367751 |
| rs13340461 | 6 | 41924278 | T | C | 0.274747 | 0.0179591 | 0.00145485 | 5.19996E-35 | -0.0092 | 0.0104 | 0.3793 | 0.000329757 | 152.3810004 |
| rs13406427 | 2 | 46878351 | G | C | 0.267148 | 0.0115864 | 0.00146473 | 2.60016E-15 | 0.0047 | 0.0105 | 0.6556 | 0.000135434 | 62.57199497 |
| rs13412980 | 2 | 1545231 | A | C | 0.176471 | -0.00985618 | 0.00170219 | 7.00003E-09 | 0.0023 | 0.0121 | 0.852 | 7.25729E-05 | 33.52735575 |
| rs1352982 | 10 | 25332461 | G | A | 0.36734 | 0.0100862 | 0.00135821 | 1.10002E-13 | -0.0027 | 0.0096 | 0.7792 | 0.000119364 | 55.14668657 |
| rs1353778 | 3 | 134088443 | T | A | 0.614449 | -0.00852307 | 0.00133957 | 2E-10 | -0.0007 | 0.0095 | 0.9395 | 8.7625E-05 | 40.48176176 |
| rs136029 | 22 | 46236425 | A | G | 0.413605 | 0.0119752 | 0.00133995 | 4.00037E-19 | -0.0089 | 0.0094 | 0.3467 | 0.000172869 | 79.87051494 |
| rs138198562 | 12 | 46429309 | C | T | 0.048489 | 0.0217132 | 0.00311052 | 2.90001E-12 | 0.0118 | 0.0219 | 0.5907 | 0.000105473 | 48.7281349 |
| rs138548025 | 4 | 135114664 | C | T | 0.044957 | 0.0194099 | 0.00331261 | 4.60002E-09 | 0.0196 | 0.0233 | 0.4 | 7.43154E-05 | 34.33239483 |
| rs138937927 | 16 | 51095561 | T | C | 0.025628 | -0.0449331 | 0.00430641 | 1.69981E-25 | -0.1031 | 0.0303 | 0.000677205 | 0.000235616 | 108.8679722 |
| rs1393421 | 15 | 38369210 | A | G | 0.936952 | 0.0159563 | 0.00269382 | 3.2E-09 | 0.0425 | 0.019 | 0.0257502 | 7.5945E-05 | 35.08531337 |
| rs1395602 | 16 | 4911239 | C | A | 0.48406 | 0.00983951 | 0.00131694 | 7.89951E-14 | 0.0094 | 0.0093 | 0.3105 | 0.000120828 | 55.82298875 |
| rs140182288 | 1 | 2226159 | T | C | 0.026695 | 0.0234688 | 0.00420287 | 2.39999E-08 | 0.0292 | 0.0298 | 0.3262 | 6.74941E-05 | 31.1808517 |
| rs140753094 | 1 | 40641118 | A | C | 0.124412 | 0.0149373 | 0.00196963 | 3.40017E-14 | 0.0165 | 0.014 | 0.2393 | 0.000124488 | 57.51392749 |
| rs1415701 | 6 | 130345835 | A | G | 0.267544 | -0.0314558 | 0.00148201 | 5.60015E-100 | 0.0084 | 0.0106 | 0.4305 | 0.000974274 | 450.5026202 |
| rs1420150 | 7 | 33321872 | G | C | 0.217195 | -0.00956276 | 0.0015813 | 1.5E-09 | 0.0124 | 0.0112 | 0.2711 | 7.91605E-05 | 36.57093722 |
| rs142190120 | 2 | 219953832 | T | C | 0.012999 | -0.0460584 | 0.00582691 | 2.70023E-15 | 0.0463 | 0.0413 | 0.2626 | 0.000135234 | 62.47973712 |
| rs143384 | 20 | 34025756 | G | A | 0.404382 | 0.0593269 | 0.00133434 | 1E-200 | -0.0935 | 0.0095 | 4.77309E-23 | 0.004261091 | 1976.825893 |
| rs143840904 | 11 | 2813322 | T | C | 0.019757 | -0.103446 | 0.00485719 | 1.20005E-100 | 0.0586 | 0.0343 | 0.0875105 | 0.000980926 | 453.5815072 |
| rs144351518 | 4 | 1893651 | G | A | 0.032685 | -0.0364062 | 0.00380837 | 1.20005E-21 | -0.0121 | 0.027 | 0.6548 | 0.000197784 | 91.38408129 |
| rs1443536 | 4 | 82174165 | G | A | 0.30494 | 0.031917 | 0.00141688 | 2.3014E-112 | 0.0195 | 0.0101 | 0.0536896 | 0.001097252 | 507.4299615 |
| rs1443749 | 7 | 121960438 | T | C | 0.366012 | 0.01181 | 0.00135269 | 2.49977E-18 | 0.0208 | 0.0096 | 0.0305197 | 0.000164982 | 76.22568855 |
| rs1443930 | 8 | 109012769 | G | T | 0.663783 | 0.00803607 | 0.0014314 | 0.00000002 | -0.0164 | 0.0101 | 0.1055 | 6.82246E-05 | 31.51835307 |
| rs145873050 | 1 | 150766213 | T | G | 0.027005 | -0.0255731 | 0.00404411 | 2.59998E-10 | -0.017 | 0.0286 | 0.551899 | 8.65542E-05 | 39.98701206 |
| rs146381464 | 3 | 51463177 | A | C | 0.02298 | 0.0798442 | 0.00438388 | 4.10015E-74 | 0.0236 | 0.0309 | 0.4459 | 0.000717567 | 331.7168648 |
| rs1467847 | 21 | 35714544 | C | G | 0.565774 | 0.0156957 | 0.00133329 | 5.40008E-32 | -0.0043 | 0.0094 | 0.6448 | 0.000299907 | 138.5830945 |
| rs1468177 | 22 | 30544559 | C | T | 0.361212 | 0.010722 | 0.00137294 | 5.70033E-15 | 0.0204 | 0.0097 | 0.0346003 | 0.000132007 | 60.98831141 |
| rs146851424 | 13 | 50377910 | C | A | 0.021614 | 0.0943173 | 0.00455703 | 3.69999E-95 | 0.0058 | 0.032 | 0.8558 | 0.000926449 | 428.3680428 |
| rs147110934 | 19 | 55993436 | T | G | 0.024305 | -0.0662951 | 0.00425986 | 1.29987E-54 | -0.0427 | 0.0304 | 0.1602 | 0.000524022 | 242.1978744 |
| rs147176253 | 16 | 3946485 | A | G | 0.063619 | -0.0183888 | 0.00274233 | 1.99986E-11 | 0.041 | 0.0194 | 0.0346801 | 9.73263E-05 | 44.96405486 |
| rs1472565 | 1 | 19755030 | C | T | 0.46706 | -0.0150252 | 0.00130066 | 7.19946E-31 | 0.0043 | 0.0093 | 0.6468 | 0.000288797 | 133.4476942 |
| rs1474647 | 1 | 22441865 | T | C | 0.551059 | -0.0133275 | 0.0013037 | 1.59993E-24 | 0.0133 | 0.0093 | 0.1529 | 0.000226177 | 104.5057463 |
| rs147472851 | 14 | 36245495 | A | G | 0.257217 | 0.0128882 | 0.00151727 | 1.99986E-17 | 0.0027 | 0.0107 | 0.7993 | 0.000156169 | 72.15341845 |
| rs1490384 | 6 | 126851160 | T | C | 0.50007 | 0.032697 | 0.00129566 | 1.5996E-140 | -0.0303 | 0.0093 | 0.001073 | 0.001376704 | 636.8421967 |
| rs1490819 | 5 | 67099798 | T | C | 0.162067 | 0.0162271 | 0.00177869 | 7.29962E-20 | 0.0004 | 0.0127 | 0.9732 | 0.000180139 | 83.22990864 |
| rs149290349 | 2 | 43451957 | A | G | 0.074709 | 0.0141698 | 0.00249271 | 1.29999E-08 | -0.0283 | 0.0179 | 0.1134 | 6.99453E-05 | 32.31335476 |
| rs149802978 | 5 | 102346866 | G | C | 0.052507 | -0.0218764 | 0.00292927 | 8.10028E-14 | 0.0625 | 0.0206 | 0.00243602 | 0.000120722 | 55.77390125 |
| rs149840699 | 20 | 49210579 | A | G | 0.079246 | 0.0189953 | 0.00246483 | 1.29987E-14 | -0.0391 | 0.0174 | 0.0242499 | 0.000128549 | 59.39043293 |
| rs150095746 | 8 | 76486799 | G | A | 0.029312 | 0.0225464 | 0.00396379 | 1.29999E-08 | 0.0168 | 0.0278 | 0.546 | 7.00338E-05 | 32.35424424 |
| rs151123887 | 20 | 34593288 | A | G | 0.022536 | -0.0406349 | 0.00461924 | 1.39991E-18 | 0.0623 | 0.0346 | 0.0717596 | 0.00016749 | 77.38476281 |
| rs1512104 | 4 | 26021310 | A | G | 0.210996 | 0.00890924 | 0.00159757 | 0.000000025 | -0.0115 | 0.0114 | 0.3148 | 6.73189E-05 | 31.09994659 |
| rs151305167 | 2 | 5832142 | T | C | 0.098453 | -0.0142065 | 0.00217822 | 6.89922E-11 | -0.0036 | 0.0156 | 0.8174 | 9.20737E-05 | 42.53719307 |
| rs1513572 | 4 | 21850305 | C | T | 0.378205 | 0.00745457 | 0.00134372 | 2.90001E-08 | 0.015 | 0.0096 | 0.118 | 6.66199E-05 | 30.77696223 |
| rs1514134 | 1 | 56116513 | C | T | 0.385063 | -0.00915765 | 0.00133489 | 6.89922E-12 | -0.0096 | 0.0095 | 0.3125 | 0.000101868 | 47.06252697 |
| rs1524065 | 7 | 38126589 | A | C | 0.653528 | 0.0161542 | 0.00137038 | 4.49987E-32 | 0.0081 | 0.0097 | 0.4055 | 0.000300721 | 138.9591047 |
| rs1531851 | 2 | 20694122 | C | T | 0.261307 | -0.00829137 | 0.00147785 | 0.00000002 | -0.0161 | 0.0106 | 0.127 | 6.81345E-05 | 31.47675832 |
| rs153661 | 5 | 141809136 | C | G | 0.551008 | 0.00734321 | 0.00131281 | 2.19999E-08 | -0.0263 | 0.0093 | 0.00489801 | 6.77242E-05 | 31.2871687 |
| rs1545552 | 2 | 33360338 | G | A | 0.708678 | 0.0237003 | 0.00143413 | 2.39994E-61 | -0.0246 | 0.0103 | 0.0166498 | 0.000590852 | 273.1044427 |
| rs1563387 | 8 | 121179924 | T | G | 0.379581 | -0.00831581 | 0.00136476 | 1.09999E-09 | -0.0007 | 0.0097 | 0.9437 | 8.03649E-05 | 37.12741097 |
| rs1569364 | 8 | 116629732 | T | C | 0.559979 | -0.0113582 | 0.00132553 | 1E-17 | -0.0295 | 0.0094 | 0.00166901 | 0.000158919 | 73.42399662 |
| rs1573891 | 15 | 99186488 | C | G | 0.158554 | -0.0339015 | 0.00179921 | 3.40017E-79 | 0.0341 | 0.0128 | 0.00792994 | 0.000767972 | 355.0358725 |
| rs1574220 | 5 | 314518 | C | T | 0.080908 | 0.0220335 | 0.00241534 | 7.39946E-20 | 0.0274 | 0.0171 | 0.1095 | 0.00018011 | 83.21632875 |
| rs1593071 | 5 | 42786309 | C | A | 0.452827 | -0.0181821 | 0.0013104 | 8.9002E-44 | 0.0046 | 0.0093 | 0.619699 | 0.000416586 | 192.5211446 |
| rs1599473 | 8 | 120475358 | T | G | 0.243616 | -0.0199866 | 0.00152862 | 4.60045E-39 | 0.0002 | 0.0108 | 0.9826 | 0.000369933 | 170.953063 |
| rs1624841 | 18 | 7556208 | T | C | 0.189407 | 0.0103182 | 0.00168092 | 8.30004E-10 | 0.0008 | 0.0119 | 0.9479 | 8.15612E-05 | 37.68008848 |
| rs1679910 | 1 | 53582321 | A | G | 0.43707 | 0.00900739 | 0.0013179 | 8.19974E-12 | 0.0097 | 0.0094 | 0.3015 | 0.00010111 | 46.7122878 |
| rs16824590 | 2 | 203137347 | A | G | 0.114179 | -0.0157893 | 0.00203714 | 9.09913E-15 | 0.0016 | 0.0146 | 0.9131 | 0.000130027 | 60.07338718 |
| rs16905189 | 8 | 135598132 | G | C | 0.366803 | -0.0274204 | 0.0013622 | 4.10015E-90 | -0.0085 | 0.0096 | 0.3796 | 0.000876375 | 405.1948711 |
| rs16928433 | 12 | 1546814 | A | G | 0.113665 | -0.0135168 | 0.00205824 | 5.10035E-11 | -0.0033 | 0.0147 | 0.8245 | 9.33513E-05 | 43.12745864 |
| rs16942323 | 15 | 89383764 | C | T | 0.026897 | -0.0926082 | 0.00404404 | 4.6989E-116 | 0.0193 | 0.0288 | 0.5024 | 0.001133914 | 524.4041508 |
| rs16951879 | 17 | 1969912 | T | C | 0.019924 | 0.0332192 | 0.0046744 | 1.20005E-12 | -0.028 | 0.0334 | 0.4014 | 0.000109316 | 50.50387621 |
| rs17034560 | 1 | 10229158 | A | G | 0.037123 | 0.0199313 | 0.00343081 | 6.29999E-09 | 0.0253 | 0.0246 | 0.3041 | 7.30552E-05 | 33.75017564 |
| rs17038164 | 1 | 118862669 | C | T | 0.256793 | -0.0306447 | 0.00148262 | 6.4998E-95 | 0.0181 | 0.0106 | 0.0885401 | 0.000923963 | 427.2176231 |
| rs17038954 | 2 | 1645673 | T | C | 0.062856 | 0.0199328 | 0.00268698 | 1.20005E-13 | 0.0193 | 0.0191 | 0.314 | 0.000119114 | 55.03080558 |
| rs17148514 | 11 | 85526399 | T | C | 0.241691 | -0.0135592 | 0.00153714 | 1.10002E-18 | 0.0102 | 0.0109 | 0.347 | 0.000168412 | 77.8107148 |
| rs17157112 | 7 | 28779946 | G | T | 0.470644 | -0.0162004 | 0.00131283 | 5.50047E-35 | -0.0025 | 0.0093 | 0.7902 | 0.000329531 | 152.2764033 |
| rs17197114 | 14 | 21894526 | C | T | 0.177044 | 0.0158329 | 0.00173112 | 5.90065E-20 | 0.0114 | 0.0122 | 0.3502 | 0.000181048 | 83.64976197 |
| rs17199964 | 4 | 102707791 | A | G | 0.078617 | -0.0155788 | 0.002421 | 1.2E-10 | 0.0483 | 0.0173 | 0.00517905 | 8.96282E-05 | 41.40726489 |
| rs172179 | 13 | 30177337 | C | G | 0.59189 | -0.00987559 | 0.00133695 | 1.50003E-13 | -0.0221 | 0.0094 | 0.0190498 | 0.0001181 | 54.56245352 |
| rs17236066 | 2 | 174863486 | A | G | 0.079443 | 0.017539 | 0.00240321 | 2.90001E-13 | -0.0143 | 0.0171 | 0.4028 | 0.000115287 | 53.26284141 |
| rs1729090 | 8 | 13107258 | T | C | 0.385022 | -0.00980812 | 0.00135255 | 4.10015E-13 | 0.0206 | 0.0096 | 0.0318897 | 0.00011382 | 52.58513627 |
| rs1730040 | 3 | 158030962 | G | A | 0.415777 | 0.0155556 | 0.00132077 | 5.10035E-32 | -0.0086 | 0.0094 | 0.3623 | 0.000300188 | 138.7130312 |
| rs17357954 | 1 | 41487337 | T | G | 0.219511 | 0.0304968 | 0.00156668 | 2.09991E-84 | 0.0053 | 0.0112 | 0.6329 | 0.00081959 | 378.9187224 |
| rs17400325 | 2 | 178565913 | C | T | 0.041564 | 0.0402301 | 0.00324912 | 3.29989E-35 | -0.0246 | 0.0234 | 0.2931 | 0.000331766 | 153.3094201 |
| rs17409588 | 5 | 31528627 | C | T | 0.317755 | 0.010157 | 0.00139921 | 3.90032E-13 | 0.0065 | 0.0099 | 0.5111 | 0.000114057 | 52.69424956 |
| rs1741344 | 20 | 4101800 | T | C | 0.634536 | -0.0200927 | 0.0013607 | 2.39994E-49 | -0.0152 | 0.0096 | 0.1132 | 0.000471793 | 218.0469168 |
| rs174529 | 11 | 61543961 | C | T | 0.373656 | -0.00931697 | 0.00135464 | 6.09958E-12 | -0.0095 | 0.0096 | 0.3236 | 0.000102391 | 47.30420229 |
| rs17454369 | 4 | 87666334 | C | G | 0.055533 | 0.0194408 | 0.00284344 | 8.10028E-12 | 0.0009 | 0.0203 | 0.964 | 0.000101182 | 46.74533188 |
| rs17472816 | 10 | 12020794 | G | A | 0.055958 | -0.0238025 | 0.00289137 | 1.80011E-16 | 0.009 | 0.0203 | 0.6574 | 0.000146683 | 67.76975665 |
| rs17511102 | 2 | 37960613 | T | A | 0.08908 | 0.0321776 | 0.00228094 | 3.40017E-45 | 0.0167 | 0.0163 | 0.306 | 0.000430623 | 199.0112806 |
| rs17592880 | 11 | 59286521 | G | A | 0.044358 | -0.0191343 | 0.00318943 | 0.000000002 | 0.0036 | 0.0226 | 0.8727 | 7.79059E-05 | 35.99126635 |
| rs17599450 | 19 | 30328753 | T | C | 0.321679 | 0.00909659 | 0.0014144 | 1.29999E-10 | 0.0103 | 0.01 | 0.3022 | 8.95321E-05 | 41.36288912 |
| rs17741562 | 2 | 148563066 | C | T | 0.200712 | 0.00956635 | 0.00162211 | 3.69999E-09 | -0.0091 | 0.0116 | 0.4318 | 7.52843E-05 | 34.78003773 |
| rs17800727 | 16 | 53481010 | G | A | 0.300422 | 0.020833 | 0.0014309 | 5.10035E-48 | -0.0066 | 0.0101 | 0.5128 | 0.00045866 | 211.9742855 |
| rs17855988 | 7 | 73474825 | C | G | 0.098535 | 0.0190333 | 0.00222538 | 1.20005E-17 | -0.0201 | 0.0159 | 0.2052 | 0.000158327 | 73.15060871 |
| rs184090834 | 2 | 219873482 | A | T | 0.014744 | -0.086633 | 0.0057025 | 4.00037E-52 | 0.068 | 0.0405 | 0.0930101 | 0.000499372 | 230.7991682 |
| rs1863758 | 15 | 56151030 | G | A | 0.702892 | 0.0081748 | 0.00143038 | 1.09999E-08 | -0.0114 | 0.0101 | 0.2634 | 7.0701E-05 | 32.66250838 |
| rs1867780 | 15 | 77407114 | G | C | 0.278162 | -0.014751 | 0.00145822 | 4.70002E-24 | -0.0245 | 0.0103 | 0.0177399 | 0.000221465 | 102.3281081 |
| rs1875969 | 5 | 168269313 | C | G | 0.417207 | -0.0130602 | 0.0013254 | 6.59933E-23 | -0.0044 | 0.0094 | 0.642401 | 0.000210145 | 97.09655973 |
| rs1884897 | 20 | 6612832 | G | A | 0.627397 | -0.0345351 | 0.00136093 | 4.6026E-142 | -0.0195 | 0.0096 | 0.0432803 | 0.001392034 | 643.9438209 |
| rs188571030 | 15 | 75786845 | T | C | 0.009833 | 0.0474622 | 0.00709877 | 2.29985E-11 | -0.0178 | 0.0503 | 0.723801 | 9.67593E-05 | 44.70207052 |
| rs1889643 | 6 | 141721197 | A | C | 0.744142 | -0.00967997 | 0.00148826 | 7.8001E-11 | -0.0132 | 0.0107 | 0.215 | 9.15705E-05 | 42.30469065 |
| rs1910252 | 8 | 49407362 | T | C | 0.167525 | 0.016643 | 0.00175447 | 2.39994E-21 | 0.0018 | 0.0125 | 0.8831 | 0.000194756 | 89.9848616 |
| rs1948047 | 6 | 47585912 | C | T | 0.275951 | 0.0114274 | 0.00144909 | 3.10027E-15 | 0.0245 | 0.0104 | 0.0178801 | 0.000134602 | 62.18737997 |
| rs1952256 | 1 | 184035116 | G | A | 0.344989 | 0.0327572 | 0.00136523 | 3.1989E-127 | -0.0167 | 0.0098 | 0.0859805 | 0.001244704 | 575.7050225 |
| rs1961247 | 11 | 32949014 | T | C | 0.333522 | 0.00841727 | 0.00138744 | 1.29999E-09 | -0.0207 | 0.0098 | 0.0351901 | 7.96681E-05 | 36.80545695 |
| rs1962482 | 1 | 86313536 | T | G | 0.16762 | -0.0122811 | 0.00174044 | 1.69981E-12 | -0.0335 | 0.0125 | 0.00708093 | 0.000107774 | 49.79142355 |
| rs1984119 | 9 | 98368761 | C | T | 0.255627 | -0.0295024 | 0.00151581 | 2.29985E-84 | 0.0082 | 0.0107 | 0.4457 | 0.000819359 | 378.8116074 |
| rs1985278 | 1 | 21091861 | T | G | 0.39085 | -0.0152699 | 0.001342 | 5.40008E-30 | -0.0072 | 0.0096 | 0.452101 | 0.000280189 | 129.4689047 |
| rs1985278 | 1 | 21091861 | T | G | 0.39085 | -0.0152699 | 0.001342 | 5.40008E-30 | -0.2213 | 0.1711 | 0.196 | 0.000280189 | 129.4689047 |
| rs1985299 | 3 | 183538416 | G | A | 0.501858 | 0.00882874 | 0.00131554 | 1.9002E-11 | -0.0205 | 0.0094 | 0.0283498 | 9.74882E-05 | 45.03886723 |
| rs1990657 | 5 | 171220503 | C | T | 0.428274 | -0.0178406 | 0.00131983 | 1.20005E-41 | -0.0069 | 0.0094 | 0.4596 | 0.000395381 | 182.717877 |
| rs1996711 | 3 | 146980646 | C | T | 0.493543 | -0.0120917 | 0.00130245 | 1.59993E-20 | 0.0006 | 0.0093 | 0.9474 | 0.000186542 | 86.18877871 |
| rs2006122 | 17 | 61987405 | T | A | 0.270182 | 0.0374011 | 0.00147924 | 4.7973E-141 | 0.0195 | 0.0105 | 0.0628102 | 0.001381961 | 639.277746 |
| rs2016437 | 1 | 235539355 | G | T | 0.6442 | 0.00948708 | 0.00135491 | 2.49977E-12 | 0.0013 | 0.0097 | 0.8947 | 0.000106121 | 49.0277936 |
| rs2034395 | 2 | 121610186 | T | G | 0.167946 | -0.0234328 | 0.00174005 | 2.49977E-41 | -0.0141 | 0.0124 | 0.2568 | 0.000392428 | 181.3522866 |
| rs2035901 | 4 | 145521867 | G | A | 0.468308 | 0.0239698 | 0.00130919 | 7.00003E-75 | -0.0015 | 0.0093 | 0.8711 | 0.000725126 | 335.2135626 |
| rs2061708 | 1 | 103417203 | C | G | 0.588873 | -0.0176111 | 0.00132202 | 1.69981E-40 | -0.0122 | 0.0094 | 0.196 | 0.000384003 | 177.4577763 |
| rs2065036 | 22 | 28099361 | T | C | 0.651044 | -0.0094055 | 0.00138815 | 1.20005E-11 | 0.014 | 0.0098 | 0.1516 | 9.93694E-05 | 45.9080769 |
| rs2072346 | 16 | 4027423 | T | C | 0.809557 | 0.0157038 | 0.00166713 | 4.49987E-21 | 0.0119 | 0.0118 | 0.3121 | 0.00019204 | 88.7296355 |
| rs2093210 | 14 | 60957279 | T | C | 0.607414 | -0.0289701 | 0.00134427 | 5.2E-103 | -0.0069 | 0.0095 | 0.4703 | 0.001004374 | 464.4351649 |
| rs2099333 | 19 | 19605963 | T | C | 0.178765 | -0.0197806 | 0.0017156 | 9.30037E-31 | -0.0029 | 0.0121 | 0.8123 | 0.000287691 | 132.9367222 |
| rs2110748 | 2 | 202345476 | A | C | 0.977882 | -0.0312838 | 0.00441624 | 1.39991E-12 | 0.0769 | 0.0315 | 0.0145001 | 0.000108615 | 50.18013058 |
| rs2131371 | 12 | 46796522 | C | A | 0.701066 | 0.014953 | 0.00142437 | 8.80035E-26 | -0.0002 | 0.0101 | 0.9852 | 0.000238513 | 110.2069924 |
| rs2138628 | 9 | 108931732 | A | T | 0.313646 | -0.0160451 | 0.00141015 | 5.40008E-30 | -0.0038 | 0.01 | 0.704001 | 0.00028018 | 129.4649931 |
| rs2138628 | 9 | 108931732 | A | T | 0.313646 | -0.0160451 | 0.00141015 | 5.40008E-30 | -0.0779 | 0.2309 | 0.7358 | 0.00028018 | 129.4649931 |
| rs215226 | 12 | 591300 | G | A | 0.403856 | 0.0188864 | 0.0013343 | 1.80011E-45 | -0.0048 | 0.0095 | 0.614899 | 0.000433519 | 200.3500774 |
| rs2157323 | 6 | 116729779 | C | T | 0.732553 | 0.0146702 | 0.00146427 | 1.29987E-23 | 0.0054 | 0.0105 | 0.609 | 0.00021724 | 100.3755319 |
| rs2181834 | 10 | 102661251 | T | G | 0.550246 | 0.0158175 | 0.00131684 | 3.10027E-33 | 0.002 | 0.0093 | 0.8301 | 0.000312233 | 144.2805489 |
| rs219162 | 2 | 33219099 | G | C | 0.672358 | 0.00962182 | 0.00138927 | 4.30031E-12 | 0.0132 | 0.0099 | 0.1826 | 0.000103825 | 47.9666359 |
| rs2194411 | 3 | 185548663 | A | G | 0.128404 | 0.0433997 | 0.00196985 | 1.3996E-107 | 0.0169 | 0.014 | 0.2281 | 0.001049678 | 485.406135 |
| rs2219320 | 1 | 26803430 | C | T | 0.25388 | -0.0181363 | 0.00148837 | 3.69999E-34 | 0.0182 | 0.0107 | 0.08684 | 0.000321322 | 148.4819582 |
| rs2229094 | 6 | 31540556 | C | T | 0.254512 | -0.0241271 | 0.00148792 | 3.90032E-59 | -0.0118 | 0.0107 | 0.2691 | 0.000568865 | 262.9354876 |
| rs2239748 | 13 | 33101817 | T | C | 0.361062 | 0.0141323 | 0.00136989 | 5.90065E-25 | 0.0019 | 0.0097 | 0.8437 | 0.000230335 | 106.4271482 |
| rs2240169 | 19 | 1018830 | T | C | 0.337209 | 0.00865227 | 0.00138757 | 4.49997E-10 | -0.0103 | 0.0098 | 0.294 | 8.41625E-05 | 38.88198635 |
| rs2270894 | 3 | 9975386 | G | C | 0.203212 | -0.0273135 | 0.0016803 | 2.09991E-59 | 0.0008 | 0.0119 | 0.9451 | 0.00057166 | 264.2282053 |
| rs227745 | 17 | 54762165 | A | T | 0.27042 | 0.0193513 | 0.00147101 | 1.59993E-39 | 0.0362 | 0.0104 | 0.000517798 | 0.000374482 | 173.0561992 |
| rs228279 | 17 | 36918350 | C | T | 0.226818 | -0.0160347 | 0.00156549 | 1.29987E-24 | 0.0074 | 0.0111 | 0.5059 | 0.000227053 | 104.9105592 |
| rs2290154 | 18 | 77211175 | C | T | 0.35832 | -0.0152067 | 0.00137417 | 1.80011E-28 | 0.0016 | 0.0097 | 0.8655 | 0.00026502 | 122.4579966 |
| rs2302580 | 4 | 8608634 | T | C | 0.420754 | -0.017225 | 0.00132649 | 1.50003E-38 | 0.0068 | 0.0095 | 0.4714 | 0.000364886 | 168.6198196 |
| rs2303597 | 2 | 71780821 | C | T | 0.705845 | -0.013328 | 0.00143228 | 1.29987E-20 | 0.0244 | 0.0102 | 0.01714 | 0.000187412 | 86.59089583 |
| rs2305141 | 2 | 233684402 | G | A | 0.596002 | 0.0136081 | 0.00132401 | 8.9002E-25 | 0.0178 | 0.0095 | 0.0605899 | 0.000228622 | 105.6356657 |
| rs2326181 | 20 | 3312596 | T | C | 0.498794 | -0.00858957 | 0.00130993 | 5.50047E-11 | -0.006 | 0.0093 | 0.516 | 9.30703E-05 | 42.99765685 |
| rs235763 | 20 | 6705246 | C | T | 0.402424 | -0.0159447 | 0.00134226 | 1.50003E-32 | -0.0026 | 0.0095 | 0.783 | 0.000305374 | 141.1099143 |
| rs2368274 | 10 | 28193276 | T | G | 0.09342 | -0.0129144 | 0.00227128 | 1.29999E-08 | -0.0078 | 0.0161 | 0.6278 | 6.99813E-05 | 32.32996496 |
| rs2378788 | 1 | 183918161 | G | A | 0.733254 | -0.00863486 | 0.00147526 | 4.79999E-09 | 0.0269 | 0.0105 | 0.0106001 | 7.4156E-05 | 34.25875582 |
| rs2380893 | 2 | 141585138 | G | A | 0.573642 | 0.00747402 | 0.00131187 | 0.000000012 | 0.0081 | 0.0094 | 0.3856 | 7.02589E-05 | 32.45824467 |
| rs2384145 | 2 | 25472066 | C | G | 0.413739 | 0.0275068 | 0.00131834 | 1.10002E-96 | 0.0304 | 0.0094 | 0.00123401 | 0.000941502 | 435.3348153 |
| rs2410728 | 5 | 86340997 | G | C | 0.758965 | 0.0110118 | 0.00152575 | 5.30029E-13 | -0.0012 | 0.0109 | 0.9095 | 0.000112747 | 52.08923658 |
| rs2427312 | 20 | 60970591 | T | C | 0.200183 | -0.0112594 | 0.00164021 | 6.70039E-12 | -0.0068 | 0.0116 | 0.5586 | 0.000101998 | 47.12264996 |
| rs244711 | 5 | 176509193 | T | C | 0.68588 | 0.0390758 | 0.00150735 | 3.5975E-148 | 0.0174 | 0.0106 | 0.102 | 0.001452651 | 672.0253649 |
| rs246185 | 16 | 14395432 | C | T | 0.322531 | 0.0180786 | 0.00141669 | 2.70023E-37 | 0.0079 | 0.01 | 0.4279 | 0.000352397 | 162.8463597 |
| rs2475314 | 10 | 121644781 | A | G | 0.600682 | -0.0108492 | 0.0013355 | 4.49987E-16 | 0.0084 | 0.0095 | 0.3757 | 0.00014284 | 65.99419954 |
| rs2503716 | 1 | 2149292 | T | C | 0.381908 | 0.0118798 | 0.00134316 | 9.20026E-19 | -0.0095 | 0.0096 | 0.3199 | 0.000169315 | 78.2277571 |
| rs2513299 | 11 | 68410157 | T | C | 0.846384 | 0.0221588 | 0.00183094 | 1E-33 | 0.001 | 0.0129 | 0.9367 | 0.000316965 | 146.4678713 |
| rs2527166 | 8 | 5532881 | T | C | 0.797445 | -0.00935315 | 0.00164269 | 0.000000012 | -0.0206 | 0.0116 | 0.07689 | 7.01745E-05 | 32.41922971 |
| rs2545101 | 5 | 180646014 | T | C | 0.209338 | -0.0110285 | 0.00160487 | 6.29941E-12 | -0.015 | 0.0114 | 0.1901 | 0.000102215 | 47.22275247 |
| rs2570515 | 2 | 47297082 | T | C | 0.741638 | -0.00929647 | 0.00149238 | 4.70002E-10 | 0.0151 | 0.0107 | 0.158 | 8.39935E-05 | 38.80390418 |
| rs2592831 | 4 | 1711404 | C | T | 0.335219 | 0.0175838 | 0.00137847 | 2.90001E-37 | 0.05 | 0.0098 | 3.56197E-07 | 0.000352114 | 162.7155104 |
| rs2595508 | 2 | 232253390 | G | A | 0.233248 | -0.0128559 | 0.00154298 | 8.00018E-17 | 0.012 | 0.011 | 0.2767 | 0.000150253 | 69.4196624 |
| rs2610986 | 4 | 18037231 | T | C | 0.655465 | -0.0208969 | 0.00140873 | 8.9002E-50 | 0.0203 | 0.01 | 0.0419295 | 0.000476109 | 220.0423741 |
| rs2613946 | 3 | 112825573 | G | A | 0.368215 | 0.0111566 | 0.00135865 | 2.19989E-16 | 0.0048 | 0.0097 | 0.619901 | 0.000145945 | 67.42899061 |
| rs261532 | 5 | 138949362 | T | G | 0.29319 | 0.0149261 | 0.00143194 | 1.9002E-25 | -0.0109 | 0.0102 | 0.288 | 0.000235151 | 108.6528623 |
| rs264766 | 5 | 64388979 | C | T | 0.671454 | 0.013044 | 0.00138713 | 5.30029E-21 | 0.0119 | 0.0099 | 0.2262 | 0.000191386 | 88.42709768 |
| rs2648725 | 10 | 93015079 | A | T | 0.213198 | 0.0212425 | 0.00159932 | 2.90001E-40 | -0.0096 | 0.0113 | 0.3959 | 0.000381751 | 176.4162706 |
| rs2654969 | 15 | 99532777 | G | A | 0.770165 | -0.0139065 | 0.0015544 | 3.69999E-19 | 0.0038 | 0.0111 | 0.7341 | 0.000173237 | 80.04021099 |
| rs2663335 | 17 | 929741 | T | A | 0.193058 | -0.00968285 | 0.0016694 | 6.59994E-09 | 0.0019 | 0.0118 | 0.8734 | 7.28214E-05 | 33.64214743 |
| rs2675228 | 3 | 13553380 | T | C | 0.903865 | -0.0273216 | 0.00221678 | 6.70039E-35 | -0.0293 | 0.0159 | 0.0645996 | 0.000328722 | 151.9025933 |
| rs2697546 | 5 | 90321288 | G | A | 0.67778 | -0.00906238 | 0.00139434 | 8.10028E-11 | -0.0034 | 0.0099 | 0.729201 | 9.1435E-05 | 42.24207904 |
| rs2715094 | 7 | 50730452 | A | G | 0.746788 | -0.0117822 | 0.00150163 | 4.30031E-15 | -0.0014 | 0.0107 | 0.8928 | 0.000133252 | 61.56374469 |
| rs2715553 | 17 | 38496320 | A | G | 0.542335 | 0.0145756 | 0.00131506 | 1.50003E-28 | 0.0092 | 0.0093 | 0.325 | 0.000265859 | 122.8456737 |
| rs2724616 | 12 | 11870768 | G | A | 0.645176 | -0.0207918 | 0.00136508 | 2.19989E-52 | -0.0219 | 0.0097 | 0.0239999 | 0.000501944 | 231.9883041 |
| rs2732744 | 7 | 84842884 | C | T | 0.234184 | -0.0096302 | 0.00154436 | 4.49997E-10 | -0.011 | 0.0109 | 0.3155 | 8.4167E-05 | 38.88406352 |
| rs273974 | 7 | 137614713 | T | C | 0.482511 | 0.0126058 | 0.00130606 | 4.79954E-22 | 0.0099 | 0.0093 | 0.2859 | 0.000201619 | 93.15639788 |
| rs2741311 | 2 | 233239743 | C | T | 0.921462 | -0.0287664 | 0.00242145 | 1.50003E-32 | -0.0171 | 0.0173 | 0.3216 | 0.000305416 | 141.129609 |
| rs2746026 | 17 | 18134354 | T | C | 0.40248 | 0.0118755 | 0.00133491 | 5.79963E-19 | -0.0155 | 0.0095 | 0.102 | 0.000171289 | 79.14034786 |
| rs274668 | 5 | 6711630 | T | C | 0.675726 | -0.0110548 | 0.00139559 | 2.39994E-15 | -0.0238 | 0.0099 | 0.0165101 | 0.00013581 | 62.74573366 |
| rs2755237 | 13 | 41109429 | C | A | 0.149374 | -0.0141735 | 0.00184178 | 1.39991E-14 | -0.0065 | 0.013 | 0.619801 | 0.000128182 | 59.22114918 |
| rs2763263 | 6 | 168814392 | A | T | 0.244617 | -0.0213886 | 0.00151867 | 4.79954E-45 | -0.0106 | 0.0109 | 0.3303 | 0.000429197 | 198.3517375 |
| rs2780226 | 6 | 34199092 | T | C | 0.910397 | -0.0626187 | 0.00226657 | 5.2966E-168 | -0.0326 | 0.0163 | 0.0449004 | 0.001649521 | 763.2519836 |
| rs2789514 | 9 | 129833029 | A | G | 0.867954 | 0.0172252 | 0.00194541 | 8.4004E-19 | -0.0062 | 0.0138 | 0.6556 | 0.000169683 | 78.3978907 |
| rs2793007 | 9 | 125664882 | C | G | 0.854338 | -0.0140682 | 0.00185575 | 3.40017E-14 | 0.0184 | 0.0132 | 0.1617 | 0.000124391 | 57.46934521 |
| rs2808290 | 10 | 27900882 | T | C | 0.502539 | 0.0142976 | 0.00131369 | 1.39991E-27 | 0.006 | 0.0093 | 0.516699 | 0.000256351 | 118.4509688 |
| rs2812237 | 13 | 51299120 | A | G | 0.161212 | -0.0180196 | 0.0017916 | 8.49963E-24 | -0.0208 | 0.0126 | 0.0996506 | 0.000218936 | 101.159414 |
| rs2830581 | 21 | 28293800 | A | G | 0.159809 | -0.0142002 | 0.00179788 | 2.80027E-15 | 0.0179 | 0.0126 | 0.1575 | 0.000135025 | 62.38291143 |
| rs2835676 | 21 | 38591311 | T | C | 0.339832 | 0.00934873 | 0.00139839 | 2.29985E-11 | 0.0064 | 0.0098 | 0.5138 | 9.67412E-05 | 44.69374376 |
| rs28481863 | 12 | 123902361 | A | G | 0.806258 | -0.0268967 | 0.00165343 | 1.69981E-59 | 0.0606 | 0.0118 | 2.67399E-07 | 0.000572509 | 264.6211127 |
| rs28503877 | 4 | 166319470 | G | C | 0.272566 | 0.00954249 | 0.00146801 | 8.00018E-11 | 0.0224 | 0.0104 | 0.03155 | 9.14599E-05 | 42.25358075 |
| rs285187 | 20 | 42316582 | A | G | 0.873172 | -0.0140466 | 0.00197257 | 1.10002E-12 | 0.0095 | 0.014 | 0.4967 | 0.000109758 | 50.70790906 |
| rs28519617 | 3 | 135874930 | G | T | 0.269831 | -0.0233554 | 0.00147512 | 1.80011E-56 | 0.0253 | 0.0105 | 0.0161801 | 0.000542362 | 250.6790479 |
| rs28576486 | 19 | 7213287 | A | G | 0.484186 | -0.0118971 | 0.00132391 | 2.60016E-19 | -0.011 | 0.0093 | 0.2411 | 0.000174781 | 80.75388381 |
| rs28625289 | 4 | 120523005 | T | A | 0.820096 | -0.00958788 | 0.00169722 | 0.000000016 | -0.0111 | 0.0121 | 0.3573 | 6.90787E-05 | 31.91295485 |
| rs28682325 | 2 | 54700447 | C | T | 0.041922 | 0.0251796 | 0.00325262 | 9.79941E-15 | 0.0392 | 0.0231 | 0.0901405 | 0.000129712 | 59.92790794 |
| rs28701981 | 9 | 98217581 | C | T | 0.349794 | 0.0302075 | 0.00137566 | 7.1945E-107 | -0.009 | 0.0098 | 0.3544 | 0.0010427 | 482.1758659 |
| rs2871960 | 3 | 141121814 | C | A | 0.444629 | 0.0567077 | 0.00130947 | 1E-200 | -0.0134 | 0.0093 | 0.151 | 0.004043323 | 1875.387771 |
| rs28757157 | 15 | 51545401 | T | C | 0.043329 | -0.0396557 | 0.00322524 | 9.60064E-35 | -0.0072 | 0.023 | 0.7561 | 0.000327152 | 151.1768479 |
| rs28929474 | 14 | 94844947 | T | C | 0.019759 | 0.101475 | 0.00471943 | 1.4997E-102 | -0.1249 | 0.0331 | 0.000163399 | 0.000999792 | 462.314103 |
| rs2925155 | 8 | 75886297 | T | C | 0.261235 | -0.0185374 | 0.00150258 | 5.70033E-35 | -0.0173 | 0.0106 | 0.1041 | 0.00032937 | 152.2020684 |
| rs2993548 | 1 | 212217131 | G | C | 0.01381 | 0.0423292 | 0.00559051 | 3.69999E-14 | 0.0262 | 0.0396 | 0.5082 | 0.000124088 | 57.32913429 |
| rs3020407 | 6 | 152307261 | A | G | 0.680885 | -0.0117944 | 0.00138977 | 2.09991E-17 | 0.0013 | 0.0099 | 0.8984 | 0.000155885 | 72.02179806 |
| rs3020623 | 7 | 100772845 | A | G | 0.369288 | 0.00953355 | 0.00136521 | 2.90001E-12 | 0.0049 | 0.0097 | 0.61 | 0.000105553 | 48.76502997 |
| rs3020644 | 6 | 31894626 | G | A | 0.363517 | -0.0236408 | 0.0013434 | 2.60016E-69 | -0.0178 | 0.0097 | 0.0661607 | 0.000669927 | 309.6790098 |
| rs310585 | 7 | 150895255 | C | T | 0.528082 | 0.00833599 | 0.00132376 | 2.99999E-10 | -0.0059 | 0.0094 | 0.5279 | 8.58348E-05 | 39.65463729 |
| rs310796 | 12 | 77453226 | T | G | 0.68097 | 0.0150456 | 0.00140286 | 7.8001E-27 | -0.0042 | 0.01 | 0.6721 | 0.000248936 | 115.0240032 |
| rs3116168 | 2 | 232989831 | C | T | 0.71883 | 0.0278148 | 0.00144247 | 7.50067E-83 | 0.0207 | 0.0103 | 0.0448797 | 0.000804255 | 371.8231481 |
| rs3118906 | 13 | 51106788 | A | G | 0.278307 | -0.0384798 | 0.00146566 | 6.3974E-152 | -0.0106 | 0.0103 | 0.3051 | 0.001489898 | 689.2825135 |
| rs332194 | 10 | 28922228 | C | T | 0.79989 | -0.0167507 | 0.00163959 | 1.69981E-24 | -0.0007 | 0.0116 | 0.9501 | 0.000225893 | 104.3742864 |
| rs34074573 | 14 | 23789955 | G | A | 0.162423 | 0.0223614 | 0.00177698 | 2.60016E-36 | -0.0173 | 0.0125 | 0.1685 | 0.00034268 | 158.3547229 |
| rs34118426 | 12 | 94201279 | G | A | 0.304409 | -0.0210752 | 0.00141818 | 5.90065E-50 | 0.0377 | 0.0101 | 0.000188101 | 0.000477835 | 220.8405481 |
| rs34454426 | 3 | 87346735 | A | G | 0.049438 | -0.0200949 | 0.00299813 | 1.99986E-11 | 0.0362 | 0.0214 | 0.0905191 | 9.72375E-05 | 44.92301521 |
| rs34517439 | 1 | 78450517 | A | C | 0.121783 | 0.0313075 | 0.0020067 | 7.10068E-55 | 0.0459 | 0.0143 | 0.00128201 | 0.000526633 | 243.4052812 |
| rs34544557 | 11 | 69911825 | T | C | 0.454439 | -0.0118505 | 0.00131973 | 2.70023E-19 | -0.0239 | 0.0094 | 0.01054 | 0.000174515 | 80.63085976 |
| rs34587452 | 4 | 1009900 | C | G | 0.215176 | -0.0192303 | 0.00158611 | 7.89951E-34 | -0.0221 | 0.0113 | 0.0499103 | 0.000318106 | 146.995362 |
| rs34751492 | 2 | 227268646 | C | T | 0.041991 | -0.0180555 | 0.00325241 | 2.80001E-08 | 0.0577 | 0.0231 | 0.0126401 | 6.6709E-05 | 30.81815134 |
| rs34773647 | 1 | 150130983 | T | G | 0.026536 | 0.0462097 | 0.00460311 | 1E-23 | 0.0175 | 0.0131 | 0.1823 | 0.000218109 | 100.7770662 |
| rs34773647 | 1 | 150130983 | T | G | 0.026536 | 0.0462097 | 0.00460311 | 1E-23 | 0.2526 | 0.1981 | 0.2022 | 0.000218109 | 100.7770662 |
| rs34776209 | 7 | 23513093 | T | C | 0.247527 | -0.0270927 | 0.00151477 | 1.50003E-71 | 0.0265 | 0.0108 | 0.0136499 | 0.000692015 | 319.896365 |
| rs34831515 | 19 | 17275777 | T | C | 0.231825 | -0.0184272 | 0.00155819 | 2.90001E-32 | -0.0082 | 0.011 | 0.4575 | 0.000302657 | 139.8543545 |
| rs34849253 | 18 | 33024107 | A | G | 0.32154 | 0.0125179 | 0.00142594 | 1.69981E-18 | -0.0003 | 0.01 | 0.9734 | 0.000166799 | 77.06524646 |
| rs34958982 | 11 | 47547046 | C | T | 0.350288 | -0.0209286 | 0.00136858 | 8.60003E-53 | -0.0183 | 0.0097 | 0.0591494 | 0.000505971 | 233.850389 |
| rs34970912 | 16 | 73068163 | G | C | 0.033234 | 0.0271975 | 0.00366 | 1.10002E-13 | 0.0044 | 0.0257 | 0.8636 | 0.000119522 | 55.21968435 |
| rs35130225 | 5 | 112116069 | A | G | 0.540623 | -0.0114584 | 0.00130715 | 1.9002E-18 | -0.0318 | 0.0093 | 0.000629695 | 0.000166314 | 76.84139191 |
| rs351373 | 1 | 212426631 | T | G | 0.377146 | -0.00935616 | 0.00133943 | 2.80027E-12 | 0.0089 | 0.0096 | 0.3548 | 0.000105612 | 48.79253206 |
| rs35307904 | 9 | 78511889 | A | G | 0.122458 | -0.0400395 | 0.00200502 | 1E-88 | 0.0083 | 0.0142 | 0.5612 | 0.000862522 | 398.7842456 |
| rs35348430 | 10 | 96100627 | G | A | 0.143071 | -0.011362 | 0.00187634 | 1.40001E-09 | -0.0227 | 0.0134 | 0.0896499 | 7.93701E-05 | 36.66777988 |
| rs35628589 | 20 | 61440005 | T | C | 0.070281 | 0.033654 | 0.00268123 | 3.90032E-36 | 0.0337 | 0.0188 | 0.0731897 | 0.000340928 | 157.5445414 |
| rs35665085 | 22 | 17625915 | A | G | 0.05597 | -0.0178688 | 0.00286536 | 4.49997E-10 | -0.0242 | 0.0201 | 0.2296 | 8.41784E-05 | 38.88933419 |
| rs35756741 | 12 | 12868701 | T | C | 0.09235 | -0.0257975 | 0.00226445 | 4.60045E-30 | -0.0004 | 0.0161 | 0.9802 | 0.000280875 | 129.7860128 |
| rs35874463 | 15 | 67457698 | G | A | 0.057656 | 0.0557117 | 0.00279897 | 3.69999E-88 | 0.0295 | 0.02 | 0.141 | 0.000856898 | 396.1817392 |
| rs35917062 | 2 | 191660828 | A | G | 0.107076 | -0.0173058 | 0.00209621 | 1.50003E-16 | -0.0229 | 0.015 | 0.1273 | 0.000147521 | 68.15722556 |
| rs35954730 | 10 | 12943111 | A | G | 0.289539 | -0.0217078 | 0.00144826 | 8.69961E-51 | 0.003 | 0.0103 | 0.7722 | 0.000486108 | 224.6656472 |
| rs35990522 | 9 | 119274481 | A | T | 0.074826 | 0.0259624 | 0.00248646 | 1.59993E-25 | 0.0065 | 0.0176 | 0.709601 | 0.000235955 | 109.0246846 |
| rs36226649 | 14 | 24835500 | C | T | 0.066539 | 0.0372293 | 0.00263466 | 2.49977E-45 | 0.0066 | 0.0185 | 0.721601 | 0.000432054 | 199.6727066 |
| rs3751877 | 16 | 15154898 | C | T | 0.10413 | -0.0138927 | 0.00214781 | 9.8992E-11 | -0.0178 | 0.0152 | 0.2413 | 9.05623E-05 | 41.83884769 |
| rs3751921 | 17 | 25642315 | G | A | 0.415964 | 0.00848555 | 0.0013272 | 1.6E-10 | 0.0087 | 0.0094 | 0.354 | 8.84818E-05 | 40.8775979 |
| rs3756668 | 5 | 67596088 | A | G | 0.45957 | -0.0128523 | 0.00130743 | 8.30042E-23 | -0.0104 | 0.0093 | 0.2624 | 0.000209141 | 96.63243794 |
| rs3771382 | 2 | 71559445 | G | C | 0.571147 | -0.0225667 | 0.00131291 | 3.29989E-66 | -0.0052 | 0.0094 | 0.5764 | 0.000639136 | 295.4366147 |
| rs3790086 | 16 | 69887707 | G | C | 0.454622 | -0.0176461 | 0.00131905 | 8.10028E-41 | 0.0537 | 0.0094 | 1.006E-08 | 0.000387268 | 178.9670483 |
| rs3795949 | 2 | 23926932 | A | C | 0.483136 | 0.0194778 | 0.00129877 | 7.70016E-51 | 0.0016 | 0.0093 | 0.8629 | 0.000486641 | 224.9124142 |
| rs37978 | 7 | 8017851 | G | A | 0.488796 | 0.0152603 | 0.00130978 | 2.29985E-31 | -0.0194 | 0.0093 | 0.0375301 | 0.00029377 | 135.7461601 |
| rs3807945 | 7 | 20415826 | A | G | 0.474282 | 0.0198638 | 0.00130768 | 4.10015E-52 | 0.0163 | 0.0093 | 0.0791498 | 0.000499241 | 230.7383721 |
| rs3812163 | 6 | 7725760 | T | A | 0.468535 | 0.0315446 | 0.00130033 | 5.2966E-130 | -0.0375 | 0.0093 | 5.64495E-05 | 0.001272316 | 588.4925978 |
| rs3821156 | 2 | 36741530 | G | A | 0.402278 | -0.0133128 | 0.00133246 | 1.69981E-23 | -0.0133 | 0.0095 | 0.1633 | 0.000216044 | 99.82253023 |
| rs3866205 | 4 | 124089399 | T | A | 0.862612 | 0.0119719 | 0.00189933 | 2.90001E-10 | 0.0016 | 0.0136 | 0.9063 | 8.59989E-05 | 39.73044459 |
| rs3925 | 8 | 38281658 | A | G | 0.244828 | -0.0096461 | 0.00152323 | 2.39999E-10 | 0.0011 | 0.0108 | 0.9187 | 8.6804E-05 | 40.10242824 |
| rs41271299 | 6 | 19839415 | T | C | 0.051243 | 0.088775 | 0.00293732 | 1.1995E-200 | 0.0001 | 0.021 | 0.9966 | 0.001973449 | 913.4335456 |
| rs418280 | 9 | 89082325 | C | G | 0.537266 | 0.0156747 | 0.00131515 | 9.49948E-33 | -0.0151 | 0.0093 | 0.1052 | 0.000307411 | 142.0515629 |
| rs42039 | 7 | 92244422 | T | C | 0.244327 | 0.0452162 | 0.00152023 | 2.0989E-194 | 0.0092 | 0.0108 | 0.3935 | 0.001911365 | 884.6421841 |
| rs4239436 | 18 | 20731930 | G | A | 0.789071 | 0.0497094 | 0.00161076 | 1E-200 | 0.0014 | 0.0113 | 0.9003 | 0.002057434 | 952.3870678 |
| rs4252548 | 19 | 55879672 | T | C | 0.021912 | -0.0671304 | 0.00447618 | 7.59976E-51 | 0.0512 | 0.0319 | 0.1088 | 0.00048665 | 224.9165974 |
| rs4287972 | 4 | 88645798 | T | C | 0.498265 | -0.0125379 | 0.00130013 | 5.19996E-22 | -0.0171 | 0.0093 | 0.0659903 | 0.000201277 | 92.99811887 |
| rs4291276 | 8 | 23173591 | G | A | 0.76558 | -0.0193876 | 0.00154799 | 5.50047E-36 | -0.0277 | 0.011 | 0.01149 | 0.000339445 | 156.8591726 |
| rs4293126 | 11 | 122804133 | A | G | 0.484213 | 0.0126315 | 0.00131815 | 9.3994E-22 | -0.0078 | 0.0093 | 0.4051 | 0.000198746 | 91.82867218 |
| rs4302014 | 15 | 100778771 | C | G | 0.508004 | -0.0201231 | 0.00131167 | 4.00037E-53 | -0.011 | 0.0093 | 0.2384 | 0.000509242 | 235.3632862 |
| rs4339935 | 10 | 4986044 | T | G | 0.484404 | -0.00819393 | 0.00131074 | 4.09996E-10 | -0.0046 | 0.0093 | 0.623199 | 8.45901E-05 | 39.07954626 |
| rs4361209 | 22 | 39281636 | G | A | 0.222777 | 0.0139216 | 0.00158507 | 1.59993E-18 | 0.0018 | 0.0112 | 0.8691 | 0.000166961 | 77.13998138 |
| rs437853 | 2 | 196973 | C | T | 0.869143 | 0.0151544 | 0.00192581 | 3.59998E-15 | -0.0132 | 0.0138 | 0.3363 | 0.000134029 | 61.92253642 |
| rs4401680 | 6 | 140164286 | G | T | 0.782231 | 0.0103098 | 0.00156749 | 4.79954E-11 | -0.0051 | 0.0112 | 0.6496 | 9.36387E-05 | 43.2602518 |
| rs4415953 | 14 | 80943372 | A | G | 0.338573 | 0.00871477 | 0.00139026 | 3.59998E-10 | -0.0063 | 0.0098 | 0.5201 | 8.50527E-05 | 39.29324752 |
| rs4421120 | 5 | 32763118 | A | G | 0.160512 | 0.0254146 | 0.00177766 | 2.29985E-46 | -0.0085 | 0.0126 | 0.4999 | 0.000442264 | 204.3935923 |
| rs4510865 | 8 | 12685204 | T | A | 0.203058 | -0.0111613 | 0.00163696 | 9.20026E-12 | 0.0153 | 0.0116 | 0.1867 | 0.000100627 | 46.48914384 |
| rs4541010 | 15 | 62207619 | C | T | 0.450245 | 0.0106854 | 0.00131094 | 3.59998E-16 | 0.0007 | 0.0093 | 0.9402 | 0.0001438 | 66.43761876 |
| rs45446698 | 7 | 99332948 | G | T | 0.041869 | 0.0332674 | 0.0032584 | 1.80011E-24 | -0.0235 | 0.0233 | 0.3138 | 0.000225598 | 104.2382342 |
| rs4545829 | 16 | 80005642 | A | G | 0.564367 | 0.00790042 | 0.00133003 | 2.90001E-09 | 0.0021 | 0.0094 | 0.8245 | 7.63747E-05 | 35.2838206 |
| rs45474992 | 19 | 47724564 | T | C | 0.036171 | -0.0254169 | 0.0035529 | 8.4004E-13 | -0.0291 | 0.0249 | 0.2417 | 0.000110774 | 51.17729322 |
| rs45478299 | 7 | 87111321 | A | G | 0.180978 | 0.0107146 | 0.00169696 | 2.69998E-10 | -0.0126 | 0.0121 | 0.2976 | 8.62931E-05 | 39.86638292 |
| rs45601237 | 1 | 31196255 | A | G | 0.327647 | 0.00767436 | 0.00138764 | 0.000000032 | -0.0092 | 0.0099 | 0.3525 | 6.62075E-05 | 30.58643318 |
| rs4630854 | 21 | 47437220 | G | A | 0.646258 | 0.00797178 | 0.00137982 | 7.59994E-09 | 0.0122 | 0.0097 | 0.2069 | 7.22503E-05 | 33.37827267 |
| rs467022 | 5 | 55805639 | T | C | 0.744666 | -0.0089874 | 0.00149242 | 1.7E-09 | 0.0178 | 0.0106 | 0.0941499 | 7.84977E-05 | 36.26470435 |
| rs4720831 | 7 | 1566207 | A | G | 0.574623 | -0.00933862 | 0.00132154 | 1.59993E-12 | -0.0103 | 0.0094 | 0.2747 | 0.000108084 | 49.93475251 |
| rs4723399 | 7 | 35273116 | C | G | 0.387636 | -0.00950191 | 0.00133957 | 1.29987E-12 | -0.0038 | 0.0095 | 0.686501 | 0.000108905 | 50.31403171 |
| rs4735769 | 8 | 78104676 | C | A | 0.285563 | 0.0282712 | 0.00144919 | 9.30037E-85 | 0.0281 | 0.0103 | 0.00624597 | 0.000823162 | 380.5712253 |
| rs4756786 | 11 | 14285900 | A | C | 0.311714 | -0.0105649 | 0.00141259 | 7.50067E-14 | 0.0243 | 0.01 | 0.0153699 | 0.000121074 | 55.93667484 |
| rs4788218 | 16 | 30055750 | C | T | 0.400729 | 0.0157475 | 0.00133877 | 6.09958E-32 | 0.0234 | 0.0095 | 0.01366 | 0.000299424 | 138.3596358 |
| rs4788891 | 17 | 73391145 | G | A | 0.834692 | 0.0174413 | 0.00175852 | 3.50026E-23 | 0.0143 | 0.0125 | 0.2556 | 0.0002129 | 98.36966678 |
| rs4814656 | 20 | 17769540 | A | G | 0.436631 | -0.0113647 | 0.00131368 | 5.10035E-18 | 0.0033 | 0.0093 | 0.7227 | 0.000161984 | 74.84019241 |
| rs4865956 | 5 | 54882505 | A | T | 0.696527 | -0.0249252 | 0.00142258 | 9.8992E-69 | 0.0028 | 0.0101 | 0.7804 | 0.000664111 | 306.9884428 |
| rs4870056 | 6 | 152162227 | A | G | 0.456635 | 0.0226879 | 0.00129985 | 3.19963E-68 | -0.0165 | 0.0093 | 0.0767096 | 0.000659054 | 304.6493398 |
| rs4870941 | 8 | 126498828 | C | G | 0.23786 | -0.017544 | 0.00155796 | 1.99986E-29 | 0.0257 | 0.011 | 0.0197301 | 0.000274429 | 126.8068606 |
| rs4886782 | 15 | 74228810 | A | G | 0.355017 | -0.0234146 | 0.00137081 | 2.09991E-65 | 0.0083 | 0.0097 | 0.3931 | 0.000631175 | 291.7541739 |
| rs4887952 | 16 | 78347216 | A | G | 0.203701 | -0.0129638 | 0.0016307 | 1.9002E-15 | 0.0164 | 0.0115 | 0.154 | 0.000136792 | 63.19964254 |
| rs4899401 | 14 | 37004549 | G | A | 0.553185 | -0.0115377 | 0.00132402 | 2.90001E-18 | 0.003 | 0.0094 | 0.7466 | 0.000164355 | 75.93596453 |
| rs4906203 | 14 | 102928991 | T | C | 0.233784 | -0.0162773 | 0.00155042 | 8.80035E-26 | -0.0292 | 0.011 | 0.00772805 | 0.000238543 | 110.2209453 |
| rs4908774 | 1 | 8770696 | A | G | 0.20617 | 0.00927777 | 0.00160304 | 7.10003E-09 | 0.0153 | 0.0115 | 0.1833 | 7.25057E-05 | 33.49628208 |
| rs4926633 | 1 | 54887567 | C | T | 0.089009 | -0.0215072 | 0.00228009 | 4.00037E-21 | -0.0128 | 0.0163 | 0.4311 | 0.000192568 | 88.97374839 |
| rs4938330 | 11 | 116928514 | G | C | 0.206447 | -0.0091676 | 0.00161505 | 1.40001E-08 | 0.0296 | 0.0115 | 0.00987393 | 6.97452E-05 | 32.22088667 |
| rs4944961 | 11 | 74559481 | T | C | 0.316746 | 0.00772868 | 0.00140517 | 3.79997E-08 | 0.0085 | 0.01 | 0.395 | 6.54831E-05 | 30.25178697 |
| rs4969471 | 17 | 79977813 | G | C | 0.702685 | 0.0158481 | 0.00143486 | 2.29985E-28 | 0.0154 | 0.0102 | 0.131 | 0.000264013 | 121.9925945 |
| rs4974930 | 4 | 39238456 | C | A | 0.239398 | 0.0103433 | 0.00156905 | 4.30031E-11 | 0.0042 | 0.0112 | 0.7061 | 9.40608E-05 | 43.45530434 |
| rs4974968 | 4 | 40172933 | T | C | 0.759877 | 0.0136176 | 0.00153668 | 7.89951E-19 | 0.0058 | 0.0109 | 0.598 | 0.000169967 | 78.5294197 |
| rs4982404 | 14 | 21539684 | T | C | 0.541608 | 0.0158641 | 0.00132206 | 3.59998E-33 | -0.027 | 0.0093 | 0.00385203 | 0.000311601 | 143.9881217 |
| rs4986172 | 17 | 43216281 | T | C | 0.342279 | -0.0214313 | 0.00137892 | 1.80011E-54 | 0.0327 | 0.0098 | 0.000839692 | 0.000522633 | 241.5555441 |
| rs498685 | 18 | 13088673 | C | T | 0.594217 | -0.0124053 | 0.00133729 | 1.80011E-20 | 0.025 | 0.0094 | 0.008179 | 0.000186246 | 86.05209904 |
| rs505110 | 18 | 331506 | A | G | 0.375047 | 0.010915 | 0.00139506 | 5.10035E-15 | 0.0106 | 0.0098 | 0.2785 | 0.000132498 | 61.21527809 |
| rs508347 | 7 | 28212824 | C | T | 0.703136 | -0.0350415 | 0.00142837 | 6.6989E-133 | -0.0232 | 0.0101 | 0.0220201 | 0.001301138 | 601.8413963 |
| rs511987 | 11 | 125981569 | C | T | 0.368301 | -0.0117531 | 0.00138112 | 1.69981E-17 | -0.0094 | 0.0098 | 0.337 | 0.00015674 | 72.41693735 |
| rs519384 | 3 | 172168507 | A | T | 0.285298 | 0.0240742 | 0.00144628 | 3.29989E-62 | 0.0226 | 0.0103 | 0.0282202 | 0.000599437 | 277.0748373 |
| rs527393 | 2 | 169709757 | A | G | 0.468129 | 0.0177166 | 0.00129885 | 2.29985E-42 | -0.0216 | 0.0093 | 0.0201799 | 0.000402599 | 186.0548014 |
| rs52826764 | 2 | 20205541 | T | C | 0.029585 | -0.0574798 | 0.0038301 | 6.59933E-51 | -0.0213 | 0.0274 | 0.4377 | 0.000487308 | 225.2207447 |
| rs55726184 | 14 | 24834971 | A | G | 0.113022 | -0.0183882 | 0.0021617 | 1.80011E-17 | 0.0175 | 0.0152 | 0.2512 | 0.000156612 | 72.35785967 |
| rs55749333 | 17 | 7371932 | T | C | 0.638543 | -0.0247922 | 0.00136224 | 5.19996E-74 | -0.0025 | 0.0097 | 0.7947 | 0.000716501 | 331.2233888 |
| rs55778236 | 5 | 115022380 | C | T | 0.278947 | 0.0175666 | 0.00145449 | 1.39991E-33 | 0.0003 | 0.0104 | 0.974 | 0.000315662 | 145.8653253 |
| rs55837722 | 10 | 94850473 | C | G | 0.087226 | 0.0159298 | 0.00232007 | 6.59933E-12 | -0.0196 | 0.0165 | 0.2346 | 0.000102042 | 47.14293199 |
| rs55914260 | 18 | 29745501 | T | C | 0.183915 | 0.0109028 | 0.00170058 | 1.40001E-10 | -0.0216 | 0.012 | 0.0724403 | 8.8971E-05 | 41.10362042 |
| rs55944294 | 6 | 18993264 | A | C | 0.108855 | 0.0134572 | 0.00207971 | 9.79941E-11 | -0.0177 | 0.0149 | 0.2345 | 9.06295E-05 | 41.8699048 |
| rs55998444 | 2 | 128119846 | C | T | 0.337796 | 0.00795873 | 0.0013724 | 6.69993E-09 | 0.0096 | 0.0098 | 0.327 | 7.27947E-05 | 33.62979686 |
| rs56011514 | 4 | 109456614 | G | A | 0.370548 | 0.0129732 | 0.00135036 | 7.50067E-22 | -0.0183 | 0.0096 | 0.0562302 | 0.000199762 | 92.29819685 |
| rs56077950 | 4 | 122721412 | T | C | 0.319509 | 0.0173134 | 0.00139806 | 3.19963E-35 | 0.0047 | 0.0099 | 0.6339 | 0.000331875 | 153.359691 |
| rs56128104 | 7 | 56071984 | C | G | 0.236083 | 0.0112112 | 0.00154036 | 3.40017E-13 | -0.0103 | 0.011 | 0.3485 | 0.000114661 | 52.97340087 |
| rs56400819 | 8 | 143925374 | G | A | 0.555519 | 0.0100086 | 0.00131696 | 2.99985E-14 | -0.0198 | 0.0093 | 0.0335398 | 0.000125012 | 57.75633364 |
| rs56694848 | 9 | 691909 | A | G | 0.235992 | -0.00969126 | 0.00154558 | 3.59998E-10 | -0.0168 | 0.011 | 0.1243 | 8.51031E-05 | 39.31657078 |
| rs57240476 | 17 | 44195432 | T | C | 0.056623 | 0.0172839 | 0.00283084 | 0.000000001 | 0.0053 | 0.02 | 0.7898 | 8.06906E-05 | 37.27785909 |
| rs57519358 | 2 | 218595240 | G | A | 0.025459 | -0.0375832 | 0.0041318 | 9.3994E-20 | -0.0132 | 0.0299 | 0.659601 | 0.000179076 | 82.73838649 |
| rs5759006 | 22 | 43273740 | G | C | 0.490917 | -0.0100196 | 0.00131845 | 2.99985E-14 | 0.0054 | 0.0093 | 0.5587 | 0.000125004 | 57.75260239 |
| rs578475 | 5 | 134354896 | G | C | 0.305216 | -0.0220614 | 0.0014196 | 1.80011E-54 | 0.0122 | 0.0101 | 0.2269 | 0.000522531 | 241.5084198 |
| rs58087925 | 14 | 105983096 | T | C | 0.237847 | 0.0125891 | 0.00155325 | 5.30029E-16 | -0.0126 | 0.0115 | 0.2735 | 0.000142184 | 65.69083191 |
| rs591269 | 6 | 140227929 | T | C | 0.589417 | -0.00787997 | 0.00131621 | 2.1E-09 | -0.0091 | 0.0094 | 0.3325 | 7.75836E-05 | 35.84238296 |
| rs59407416 | 10 | 23779283 | T | G | 0.327235 | 0.0108766 | 0.00139721 | 7.00003E-15 | 0.0176 | 0.0099 | 0.0760992 | 0.000131163 | 60.59838688 |
| rs595767 | 17 | 46957987 | G | A | 0.518981 | -0.0210904 | 0.00131118 | 3.19963E-58 | -0.0015 | 0.0093 | 0.8702 | 0.000559766 | 258.7278149 |
| rs59735916 | 12 | 94385877 | T | C | 0.164934 | 0.0123654 | 0.00176647 | 2.60016E-12 | -0.0325 | 0.0125 | 0.00940503 | 0.000106063 | 49.00065965 |
| rs59951000 | 18 | 74975828 | T | C | 0.041874 | -0.0214303 | 0.00328485 | 6.79986E-11 | -0.0109 | 0.023 | 0.634 | 9.21277E-05 | 42.56214784 |
| rs59985551 | 2 | 56106928 | T | C | 0.226217 | -0.0500625 | 0.0015497 | 1E-200 | -0.0305 | 0.0111 | 0.00587598 | 0.002254005 | 1043.585222 |
| rs6010778 | 20 | 61522019 | C | T | 0.344158 | 0.00952757 | 0.00138161 | 5.30029E-12 | 0.0033 | 0.0098 | 0.7366 | 0.000102933 | 47.55448473 |
| rs60131767 | 4 | 186703959 | A | G | 0.474893 | 0.0087525 | 0.00130467 | 1.99986E-11 | 0.0158 | 0.0093 | 0.0889795 | 9.74149E-05 | 45.00502489 |
| rs6020201 | 20 | 48634762 | A | C | 0.223856 | -0.0131454 | 0.00157609 | 7.39946E-17 | 0.0134 | 0.0112 | 0.2285 | 0.000150565 | 69.56386509 |
| rs6024734 | 20 | 54830299 | T | C | 0.260226 | 0.0114482 | 0.00150445 | 2.80027E-14 | 0.0068 | 0.0106 | 0.5253 | 0.000125334 | 57.90512698 |
| rs60655524 | 8 | 67503514 | A | G | 0.01804 | -0.0324986 | 0.00492088 | 4.00037E-11 | 0.0648 | 0.035 | 0.0643206 | 9.44078E-05 | 43.61560246 |
| rs6071822 | 20 | 38472158 | A | G | 0.238349 | 0.0107817 | 0.00153667 | 2.29985E-12 | 0.0025 | 0.0109 | 0.8145 | 0.000106555 | 49.2279081 |
| rs6082358 | 20 | 21230455 | T | C | 0.669912 | -0.0125094 | 0.00140881 | 6.70039E-19 | -0.0039 | 0.0099 | 0.6955 | 0.000170647 | 78.8435604 |
| rs610798 | 1 | 32347703 | C | G | 0.105938 | 0.0230391 | 0.00210923 | 8.99912E-28 | 0.0248 | 0.0151 | 0.101 | 0.000258212 | 119.3112127 |
| rs6119945 | 20 | 31329256 | A | G | 0.177382 | -0.01335 | 0.00173956 | 1.69981E-14 | 0.0105 | 0.0123 | 0.3936 | 0.000127477 | 58.895461 |
| rs61635866 | 9 | 78555995 | C | T | 0.25271 | 0.0096686 | 0.00150792 | 1.40001E-10 | -0.0012 | 0.0107 | 0.9077 | 8.89891E-05 | 41.11200937 |
| rs61729527 | 8 | 77761919 | T | C | 0.051901 | -0.0234478 | 0.00295108 | 1.9002E-15 | 0.0322 | 0.021 | 0.1248 | 0.000136643 | 63.13066386 |
| rs61732778 | 3 | 187443314 | A | G | 0.071042 | 0.0332657 | 0.00253121 | 1.9002E-39 | -0.0265 | 0.018 | 0.1404 | 0.000373749 | 172.7170047 |
| rs61776719 | 1 | 38461319 | A | C | 0.553802 | -0.0163478 | 0.00130358 | 4.49987E-36 | -0.0187 | 0.0093 | 0.0451502 | 0.00034033 | 157.2683658 |
| rs61849823 | 10 | 53290991 | C | T | 0.164084 | 0.0173239 | 0.00178179 | 2.39994E-22 | 0.0083 | 0.0126 | 0.5083 | 0.000204595 | 94.53147223 |
| rs61919240 | 12 | 8831954 | A | T | 0.324041 | 0.0104752 | 0.00139343 | 5.60015E-14 | 0.0072 | 0.0099 | 0.4679 | 0.000122322 | 56.5135324 |
| rs61950149 | 13 | 21543502 | C | T | 0.116182 | -0.0217942 | 0.00209401 | 2.29985E-25 | 0.0133 | 0.0147 | 0.3681 | 0.000234438 | 108.3234494 |
| rs61987429 | 14 | 65606248 | T | C | 0.349796 | -0.0170242 | 0.00138508 | 1E-34 | 0.0397 | 0.0098 | 4.81205E-05 | 0.000326924 | 151.0712521 |
| rs62010237 | 15 | 66760891 | A | T | 0.074682 | -0.0160563 | 0.00249107 | 1.2E-10 | -0.011 | 0.0178 | 0.534399 | 8.9926E-05 | 41.5448509 |
| rs62090043 | 17 | 1627704 | C | T | 0.258346 | -0.0135924 | 0.00150117 | 1.39991E-19 | -0.0016 | 0.0106 | 0.8814 | 0.000177444 | 81.98429368 |
| rs62274159 | 3 | 156885773 | C | T | 0.250194 | -0.01453 | 0.00150568 | 4.90004E-22 | -0.0048 | 0.0107 | 0.657201 | 0.00020155 | 93.12450664 |
| rs62277396 | 3 | 156432088 | G | C | 0.041094 | -0.0297442 | 0.00331846 | 3.19963E-19 | 0.0063 | 0.0235 | 0.7894 | 0.000173885 | 80.33957515 |
| rs62289317 | 4 | 7858189 | T | G | 0.372197 | 0.0091854 | 0.00136355 | 1.59993E-11 | -0.0258 | 0.0097 | 0.00797095 | 9.82237E-05 | 45.37870847 |
| rs62294340 | 3 | 169155476 | A | G | 0.3811 | 0.00919195 | 0.00134039 | 7.00003E-12 | 0.0136 | 0.0095 | 0.1533 | 0.000101792 | 47.02741078 |
| rs62337444 | 4 | 144524940 | T | C | 0.153191 | 0.0133312 | 0.00182118 | 2.49977E-13 | 0.0124 | 0.0129 | 0.339 | 0.000115981 | 53.58347351 |
| rs62338304 | 4 | 149719861 | A | G | 0.315641 | -0.00782167 | 0.00140937 | 2.90001E-08 | 0.0039 | 0.01 | 0.6937 | 6.66692E-05 | 30.79974011 |
| rs62358929 | 5 | 37785917 | C | G | 0.073209 | 0.0193207 | 0.00251547 | 1.59993E-14 | -0.0047 | 0.0179 | 0.792801 | 0.00012769 | 58.99368834 |
| rs62370473 | 5 | 52767121 | A | G | 0.20845 | -0.0115168 | 0.00160859 | 8.10028E-13 | 0.0195 | 0.0114 | 0.0880805 | 0.000110951 | 51.25910755 |
| rs62390617 | 6 | 1623627 | C | G | 0.136156 | 0.0155324 | 0.00192026 | 6.00067E-16 | -0.0107 | 0.0138 | 0.4358 | 0.000141612 | 65.42672493 |
| rs62396185 | 6 | 26180634 | C | G | 0.259982 | -0.040883 | 0.00147838 | 2.5004E-168 | -0.0519 | 0.0106 | 0.000001012 | 0.001652723 | 764.7358963 |
| rs62423399 | 6 | 169030032 | A | C | 0.221985 | -0.00939641 | 0.00156511 | 1.89998E-09 | 0.0127 | 0.0112 | 0.2565 | 7.80199E-05 | 36.0439438 |
| rs62473545 | 7 | 130636493 | A | G | 0.162687 | -0.0146949 | 0.00177688 | 1.29987E-16 | -0.0338 | 0.0127 | 0.00759207 | 0.000148033 | 68.39355708 |
| rs62501195 | 8 | 24041988 | C | A | 0.170876 | -0.0287444 | 0.00176644 | 1.50003E-59 | -0.0063 | 0.0125 | 0.6136 | 0.000572882 | 264.7932038 |
| rs62515437 | 8 | 57160328 | T | G | 0.22515 | 0.0311254 | 0.00156698 | 8.4004E-88 | 0.0071 | 0.0111 | 0.5239 | 0.00085337 | 394.549135 |
| rs62621197 | 19 | 8670147 | T | C | 0.03717 | -0.102433 | 0.00360316 | 8.995E-178 | 0.0026 | 0.0253 | 0.9194 | 0.00174646 | 808.1850436 |
| rs6445804 | 3 | 56664852 | G | A | 0.471687 | -0.0168729 | 0.00130582 | 3.40017E-38 | -0.0105 | 0.0093 | 0.2583 | 0.000361294 | 166.9594298 |
| rs6464921 | 7 | 148386114 | T | A | 0.427934 | -0.00985017 | 0.00133827 | 1.80011E-13 | -0.0048 | 0.0095 | 0.611699 | 0.000117261 | 54.17489603 |
| rs6480350 | 10 | 70396822 | C | T | 0.575091 | 0.00879218 | 0.00132808 | 3.59998E-11 | 0.029 | 0.0094 | 0.00206001 | 9.48655E-05 | 43.82710538 |
| rs6508350 | 18 | 22707449 | T | C | 0.439531 | 0.0100026 | 0.00132562 | 4.49987E-14 | 0.0066 | 0.0094 | 0.4831 | 0.000123236 | 56.93585253 |
| rs6567160 | 18 | 57829135 | C | T | 0.232717 | 0.0213133 | 0.00155566 | 1E-42 | 0.0313 | 0.011 | 0.00437804 | 0.000406163 | 187.7025383 |
| rs6570503 | 6 | 142611580 | T | C | 0.45675 | -0.0140372 | 0.00130471 | 5.40008E-27 | 0.002 | 0.0093 | 0.8294 | 0.000250512 | 115.7526955 |
| rs6581626 | 12 | 65711378 | G | A | 0.534806 | -0.0161888 | 0.00130947 | 4.10015E-35 | 0.0083 | 0.0093 | 0.3715 | 0.00033075 | 152.8397536 |
| rs659418 | 11 | 75284334 | G | T | 0.14022 | 0.0361118 | 0.00188716 | 1.29987E-81 | 0.0012 | 0.0134 | 0.9312 | 0.00079203 | 366.1667853 |
| rs6605522 | 6 | 169656531 | G | A | 0.842945 | 0.0131363 | 0.00177952 | 1.59993E-13 | 0.0158 | 0.0127 | 0.214 | 0.000117949 | 54.49272036 |
| rs664317 | 10 | 89812230 | C | A | 0.837819 | -0.0137206 | 0.00177662 | 1.10002E-14 | -0.0099 | 0.0126 | 0.4307 | 0.000129094 | 59.64241674 |
| rs664317 | 10 | 89812230 | C | A | 0.837819 | -0.0137206 | 0.00177662 | 1.10002E-14 | 0.0719 | 0.0352 | 0.0410403 | 0.000129094 | 59.64241674 |
| rs66461782 | 1 | 71533287 | T | A | 0.270344 | 0.0113561 | 0.00146373 | 8.60003E-15 | -0.0106 | 0.0105 | 0.3093 | 0.000130282 | 60.19141986 |
| rs6658835 | 1 | 218520995 | G | A | 0.269563 | 0.0151755 | 0.0014666 | 4.30031E-25 | 0.0144 | 0.0105 | 0.1697 | 0.000231722 | 107.0682678 |
| rs6689375 | 1 | 227721627 | T | A | 0.185444 | -0.0266256 | 0.00167189 | 4.19952E-57 | 0.0097 | 0.0119 | 0.417 | 0.000548719 | 253.6188482 |
| rs6691831 | 1 | 11220180 | G | A | 0.254107 | 0.0146009 | 0.0014968 | 1.80011E-22 | 0.0224 | 0.0107 | 0.0371903 | 0.000205943 | 95.15460786 |
| rs66989638 | 2 | 106689736 | A | G | 0.120174 | 0.0184674 | 0.00201951 | 6.00067E-20 | -0.0077 | 0.0144 | 0.5945 | 0.000180986 | 83.62143494 |
| rs67012296 | 1 | 93900325 | G | C | 0.083732 | 0.0157353 | 0.00234508 | 1.9002E-11 | 0.0045 | 0.0168 | 0.787 | 9.74535E-05 | 45.0228361 |
| rs670318 | 1 | 63727542 | C | T | 0.951601 | 0.0201031 | 0.00301842 | 2.70023E-11 | 0.0226 | 0.0214 | 0.2917 | 9.6013E-05 | 44.35727375 |
| rs6739701 | 2 | 105132332 | G | A | 0.433039 | 0.0122695 | 0.00131646 | 1.20005E-20 | 0.0044 | 0.0094 | 0.641501 | 0.000188002 | 86.8633379 |
| rs6744603 | 2 | 44476117 | T | C | 0.758752 | -0.0169682 | 0.0015237 | 8.4004E-29 | 0.0131 | 0.0109 | 0.2299 | 0.000268387 | 124.0140051 |
| rs67551338 | 12 | 3393100 | T | C | 0.061471 | 0.0209748 | 0.00274535 | 2.19989E-14 | 0.0147 | 0.0195 | 0.4501 | 0.000126343 | 58.37116174 |
| rs6756738 | 2 | 47008964 | T | C | 0.339157 | -0.0167469 | 0.00138348 | 1E-33 | 0.0017 | 0.0099 | 0.8624 | 0.000317096 | 146.5281962 |
| rs6761931 | 2 | 136150512 | G | A | 0.075088 | -0.0174316 | 0.00245903 | 1.39991E-12 | 0.0202 | 0.018 | 0.2604 | 0.000108769 | 50.2510301 |
| rs6762578 | 3 | 128992047 | A | G | 0.778395 | 0.0284391 | 0.00157434 | 6.09958E-73 | -0.0044 | 0.0112 | 0.693999 | 0.000705883 | 326.3117832 |
| rs6780813 | 3 | 33196428 | C | T | 0.126886 | -0.0128969 | 0.00196277 | 5.00035E-11 | 0.0046 | 0.014 | 0.742899 | 9.34537E-05 | 43.17476295 |
| rs6814272 | 4 | 83263637 | A | G | 0.232344 | -0.00883618 | 0.0015399 | 9.59997E-09 | -0.0185 | 0.011 | 0.0929694 | 7.12719E-05 | 32.92624385 |
| rs6822154 | 4 | 120761791 | A | C | 0.340893 | -0.0100071 | 0.0013786 | 3.90032E-13 | -0.0038 | 0.0098 | 0.696101 | 0.00011405 | 52.69119531 |
| rs6824592 | 4 | 140059852 | C | G | 0.29109 | 0.00791503 | 0.00143528 | 3.50002E-08 | -0.0057 | 0.0102 | 0.574 | 6.58276E-05 | 30.41094977 |
| rs6828122 | 4 | 18006052 | G | A | 0.159339 | -0.055648 | 0.00177543 | 1E-200 | 0.024 | 0.0127 | 0.0592693 | 0.002122142 | 982.4040232 |
| rs683633 | 5 | 72400190 | C | T | 0.142337 | 0.0134783 | 0.00187392 | 6.4003E-13 | 0.0192 | 0.0134 | 0.1519 | 0.000111976 | 51.73283328 |
| rs686112 | 17 | 47325717 | C | T | 0.53573 | 0.0116947 | 0.00131371 | 5.50047E-19 | -0.0173 | 0.0093 | 0.0635697 | 0.000171518 | 79.24598705 |
| rs6863213 | 5 | 131575932 | T | C | 0.458246 | 0.0257648 | 0.0013102 | 4.30031E-86 | -0.0149 | 0.0093 | 0.1091 | 0.000836411 | 386.7020556 |
| rs686489 | 11 | 57659832 | G | A | 0.438132 | 0.00900068 | 0.00132256 | 1E-11 | 0.024 | 0.0094 | 0.0104299 | 0.000100249 | 46.31460833 |
| rs6872083 | 5 | 39418421 | G | A | 0.292156 | -0.0153536 | 0.00144046 | 1.59993E-26 | -0.0053 | 0.0102 | 0.6017 | 0.000245876 | 113.6098948 |
| rs6880702 | 5 | 158921895 | A | G | 0.65939 | -0.00882512 | 0.00138251 | 1.7E-10 | 0.0084 | 0.0098 | 0.3925 | 8.82006E-05 | 40.74767147 |
| rs6892868 | 5 | 41974551 | A | G | 0.257433 | -0.00853485 | 0.00149325 | 1.09999E-08 | 0.0073 | 0.0107 | 0.4959 | 7.07133E-05 | 32.66817388 |
| rs6900530 | 6 | 35280971 | T | C | 0.027103 | -0.0676267 | 0.0039844 | 1.29987E-64 | 0.0775 | 0.0287 | 0.00687306 | 0.000623225 | 288.0770413 |
| rs6902285 | 6 | 52384286 | C | A | 0.72424 | 0.00982159 | 0.0014515 | 1.29987E-11 | -0.0029 | 0.0104 | 0.7784 | 9.91041E-05 | 45.78546621 |
| rs6908258 | 6 | 155551005 | T | C | 0.380777 | -0.0118598 | 0.0013387 | 8.10028E-19 | 0.0012 | 0.0096 | 0.8963 | 0.000169871 | 78.48493872 |
| rs6923431 | 6 | 129824754 | C | A | 0.734283 | 0.0148565 | 0.0014538 | 1.59993E-24 | 0.0004 | 0.0104 | 0.9706 | 0.000226012 | 104.429165 |
| rs6929227 | 6 | 47646468 | C | T | 0.634884 | 0.00800658 | 0.00134716 | 2.80001E-09 | -0.0228 | 0.0096 | 0.0179399 | 7.64588E-05 | 35.32269701 |
| rs6938592 | 6 | 45097503 | T | A | 0.742179 | 0.0194436 | 0.00148293 | 2.80027E-39 | 0.0246 | 0.0106 | 0.02069 | 0.000372011 | 171.9135757 |
| rs6951990 | 7 | 120777717 | A | C | 0.38732 | -0.0120024 | 0.00133989 | 3.29989E-19 | 0.0003 | 0.0095 | 0.9768 | 0.000173671 | 80.24094237 |
| rs6962887 | 7 | 135045786 | G | T | 0.307259 | -0.01535 | 0.00142181 | 3.59998E-27 | 0.0113 | 0.0101 | 0.2643 | 0.000252249 | 116.5552259 |
| rs6964319 | 7 | 121633671 | G | A | 0.45293 | 0.00830409 | 0.00131699 | 2.90001E-10 | 0.0108 | 0.0094 | 0.2463 | 8.60571E-05 | 39.75733451 |
| rs6981529 | 8 | 145040567 | G | C | 0.399595 | -0.0190513 | 0.00133965 | 6.79986E-46 | 0.0013 | 0.0095 | 0.8893 | 0.000437604 | 202.2388815 |
| rs6985031 | 8 | 130550637 | C | T | 0.382571 | -0.0121374 | 0.00135029 | 2.49977E-19 | -0.0034 | 0.0096 | 0.7263 | 0.000174874 | 80.79702063 |
| rs6987106 | 8 | 117552823 | G | A | 0.800062 | -0.0133184 | 0.0016349 | 3.80014E-16 | -0.0156 | 0.0116 | 0.1785 | 0.000143636 | 66.36205719 |
| rs699371 | 14 | 74989433 | C | T | 0.657213 | 0.021208 | 0.00138583 | 7.29962E-53 | 0.0058 | 0.0098 | 0.5535 | 0.000506716 | 234.1950072 |
| rs7010108 | 8 | 9130674 | A | G | 0.337898 | 0.0109201 | 0.00138717 | 3.50026E-15 | -0.0232 | 0.0098 | 0.0181501 | 0.000134135 | 61.97149524 |
| rs7019613 | 9 | 101814852 | C | T | 0.136974 | -0.0151704 | 0.00190196 | 1.50003E-15 | -0.0232 | 0.0135 | 0.0857294 | 0.000137701 | 63.61937901 |
| rs702101 | 5 | 171276393 | A | G | 0.597838 | 0.0260441 | 0.00132842 | 1.39991E-85 | 0.0091 | 0.0094 | 0.3334 | 0.000831365 | 384.366932 |
| rs7038554 | 9 | 139134981 | G | A | 0.333218 | 0.0208044 | 0.00139765 | 4.10015E-50 | 0.0204 | 0.0099 | 0.0394503 | 0.000479414 | 221.5703545 |
| rs703998 | 10 | 80912499 | T | A | 0.404042 | -0.0184205 | 0.00134335 | 8.60003E-43 | 0.0094 | 0.0095 | 0.3233 | 0.000406867 | 188.0280271 |
| rs704660 | 11 | 30447998 | T | C | 0.409453 | 0.0141432 | 0.00133131 | 2.29985E-26 | 0.0095 | 0.0094 | 0.3122 | 0.000244251 | 112.8587139 |
| rs7071655 | 10 | 81234506 | T | A | 0.159122 | 0.011699 | 0.00179328 | 6.89922E-11 | -0.0048 | 0.0128 | 0.7082 | 9.21226E-05 | 42.55978171 |
| rs7078243 | 10 | 94414263 | C | A | 0.518025 | -0.00815492 | 0.0013101 | 4.79999E-10 | -0.0141 | 0.0093 | 0.1299 | 8.38685E-05 | 38.74615722 |
| rs7087507 | 10 | 63745689 | G | A | 0.347131 | 0.010655 | 0.00137673 | 1E-14 | 0.0131 | 0.0098 | 0.1792 | 0.000129646 | 59.89733165 |
| rs709631 | 17 | 40555939 | T | A | 0.691785 | -0.00783199 | 0.00141949 | 3.40001E-08 | -0.0263 | 0.0101 | 0.00893799 | 6.58955E-05 | 30.44231627 |
| rs7127911 | 11 | 128492549 | T | G | 0.266185 | 0.00939684 | 0.00148455 | 2.5E-10 | 0.0099 | 0.0105 | 0.3453 | 8.67244E-05 | 40.06564378 |
| rs7129975 | 11 | 35483521 | C | A | 0.697678 | 0.0089041 | 0.00142839 | 4.60002E-10 | 0.0136 | 0.0101 | 0.1782 | 8.41114E-05 | 38.85836964 |
| rs7134283 | 12 | 24071748 | A | G | 0.28337 | -0.0139339 | 0.00145123 | 7.89951E-22 | 0.0041 | 0.0103 | 0.691301 | 0.000199522 | 92.18728044 |
| rs71385734 | 16 | 2160503 | G | T | 0.169864 | -0.0277297 | 0.00175786 | 4.60045E-56 | 0.008 | 0.0125 | 0.5185 | 0.000538385 | 248.8398289 |
| rs71393462 | 15 | 70022499 | C | T | 0.102645 | -0.0328769 | 0.00215478 | 1.50003E-52 | -0.0159 | 0.0153 | 0.3002 | 0.000503689 | 232.7951721 |
| rs71395043 | 15 | 72110592 | A | C | 0.023915 | 0.0410177 | 0.00432793 | 2.60016E-21 | 0.0373 | 0.0305 | 0.221 | 0.000194403 | 89.82149365 |
| rs71427097 | 2 | 97526963 | T | C | 0.029401 | -0.0445082 | 0.00391406 | 5.79963E-30 | -0.0209 | 0.0277 | 0.4506 | 0.000279839 | 129.3073537 |
| rs71471298 | 10 | 105507145 | T | C | 0.111165 | 0.0205028 | 0.00208085 | 6.59933E-23 | 0.0473 | 0.0147 | 0.00130299 | 0.000210116 | 97.0829402 |
| rs7148360 | 14 | 76383138 | A | G | 0.766938 | -0.0131893 | 0.00155202 | 1.9002E-17 | -0.0153 | 0.011 | 0.1624 | 0.00015631 | 72.2182543 |
| rs71522578 | 8 | 131487853 | T | C | 0.146846 | 0.0105337 | 0.00185695 | 1.40001E-08 | 0.0102 | 0.0132 | 0.4376 | 6.96524E-05 | 32.17804024 |
| rs716203 | 4 | 88836036 | C | T | 0.525316 | -0.00726389 | 0.00130456 | 2.59998E-08 | 0.0051 | 0.0093 | 0.5848 | 6.71099E-05 | 31.00334272 |
| rs7194734 | 16 | 82199980 | T | C | 0.768655 | -0.0161231 | 0.00155432 | 3.29989E-25 | -0.0117 | 0.011 | 0.2856 | 0.000232874 | 107.6005136 |
| rs7208285 | 17 | 76746325 | A | G | 0.526335 | -0.0105311 | 0.00131427 | 1.10002E-15 | -0.006 | 0.0093 | 0.5193 | 0.000138971 | 64.20611671 |
| rs7223535 | 17 | 29211667 | A | G | 0.270324 | -0.0410865 | 0.00147254 | 2.5004E-171 | -0.0343 | 0.0104 | 0.00100399 | 0.001682433 | 778.5064447 |
| rs723149 | 7 | 46577056 | G | A | 0.562893 | -0.0169091 | 0.00131631 | 9.09913E-38 | 0.0013 | 0.0094 | 0.8878 | 0.000357087 | 165.0147416 |
| rs7239515 | 18 | 41462359 | C | G | 0.365428 | 0.00909532 | 0.00136556 | 2.70023E-11 | -0.0137 | 0.0096 | 0.1555 | 9.60235E-05 | 44.36214252 |
| rs7246328 | 19 | 18619057 | C | T | 0.138576 | -0.0132982 | 0.00189779 | 2.39994E-12 | 0.0238 | 0.0133 | 0.0740304 | 0.000106279 | 49.10068292 |
| rs7248294 | 19 | 46898234 | C | T | 0.949288 | 0.0169192 | 0.00303015 | 2.39999E-08 | 0.0315 | 0.0214 | 0.1412 | 6.7485E-05 | 31.17665469 |
| rs7255241 | 19 | 37517289 | A | C | 0.368445 | -0.0117378 | 0.00136391 | 7.59976E-18 | -0.0014 | 0.0096 | 0.8863 | 0.000160301 | 74.06279596 |
| rs72649040 | 7 | 73028240 | A | G | 0.019664 | 0.0327274 | 0.00476537 | 6.4998E-12 | -0.0361 | 0.0335 | 0.2816 | 0.000102092 | 47.16587286 |
| rs72656010 | 8 | 57122215 | C | T | 0.132179 | -0.0478676 | 0.00193835 | 1.1995E-134 | -0.0307 | 0.0138 | 0.0259102 | 0.001318411 | 609.8415759 |
| rs72703414 | 4 | 184227430 | G | A | 0.065363 | -0.0286949 | 0.002639 | 1.50003E-27 | -0.027 | 0.0188 | 0.1515 | 0.000255873 | 118.2302943 |
| rs72720395 | 14 | 64709091 | T | C | 0.259237 | -0.0102132 | 0.00150118 | 1E-11 | 0.0017 | 0.0106 | 0.8756 | 0.000100189 | 46.28670367 |
| rs72721182 | 1 | 172258325 | C | T | 0.212967 | 0.0345057 | 0.00158398 | 3.3037E-105 | 0.0331 | 0.0113 | 0.00348698 | 0.001026222 | 474.5482942 |
| rs72755233 | 15 | 100692953 | A | G | 0.111483 | -0.0618771 | 0.00207374 | 1.1995E-195 | -0.0065 | 0.0147 | 0.6567 | 0.001923624 | 890.3267314 |
| rs72759895 | 1 | 243664098 | C | G | 0.056014 | 0.0243449 | 0.00284109 | 1E-17 | -0.0203 | 0.0202 | 0.3161 | 0.000158921 | 73.42502898 |
| rs72776486 | 5 | 77515056 | A | C | 0.233035 | -0.0174637 | 0.00154154 | 9.49948E-30 | -0.013 | 0.0109 | 0.233 | 0.000277746 | 128.3397082 |
| rs72843872 | 2 | 85820027 | G | A | 0.305025 | 0.0129039 | 0.00141051 | 5.79963E-20 | -0.0105 | 0.0101 | 0.2974 | 0.000181141 | 83.69273459 |
| rs72887152 | 2 | 156604025 | A | T | 0.166018 | 0.0146187 | 0.00174535 | 5.50047E-17 | 0.0025 | 0.0125 | 0.8383 | 0.000151842 | 70.15369763 |
| rs72904749 | 1 | 51367420 | T | C | 0.089538 | 0.0327857 | 0.00226977 | 2.70023E-47 | -0.0022 | 0.0163 | 0.8936 | 0.000451455 | 208.642769 |
| rs72976986 | 19 | 4050424 | A | G | 0.190117 | 0.0158615 | 0.00168816 | 5.70033E-21 | -0.0091 | 0.0119 | 0.4454 | 0.000191066 | 88.27940949 |
| rs7299310 | 12 | 66121262 | C | T | 0.138301 | 0.0155412 | 0.00188919 | 1.9002E-16 | 0.0304 | 0.0135 | 0.0241002 | 0.000146474 | 67.67307992 |
| rs73013411 | 6 | 164126233 | A | C | 0.131224 | 0.0198838 | 0.00192424 | 5.00035E-25 | 0.0165 | 0.0138 | 0.2296 | 0.000231092 | 106.7771723 |
| rs7305516 | 12 | 102382052 | G | A | 0.493703 | -0.0188061 | 0.00131283 | 1.50003E-46 | -0.0036 | 0.0093 | 0.699199 | 0.00044401 | 205.2005957 |
| rs7306710 | 12 | 66376091 | C | T | 0.520352 | -0.0417707 | 0.00131322 | 1E-200 | -0.0179 | 0.0093 | 0.0555098 | 0.002185362 | 1011.73471 |
| rs73074166 | 20 | 5112714 | A | T | 0.102115 | -0.0185841 | 0.00217511 | 1.29987E-17 | -0.0072 | 0.0154 | 0.6381 | 0.000158 | 72.99930696 |
| rs7311238 | 12 | 54020492 | A | G | 0.182308 | -0.0135053 | 0.00168804 | 1.20005E-15 | -0.0059 | 0.012 | 0.621001 | 0.000138544 | 64.00901209 |
| rs73125628 | 20 | 20066701 | T | C | 0.278622 | -0.0117306 | 0.00146508 | 1.20005E-15 | -0.009 | 0.0104 | 0.3853 | 0.000138759 | 64.10854088 |
| rs73155600 | 4 | 56915061 | A | C | 0.270475 | -0.00938513 | 0.00146607 | 1.5E-10 | -0.0052 | 0.0104 | 0.621199 | 8.87029E-05 | 40.97974874 |
| rs731646 | 3 | 12857823 | T | C | 0.540261 | -0.00762512 | 0.00130648 | 5.30005E-09 | -0.0069 | 0.0093 | 0.4556 | 7.37328E-05 | 34.06323994 |
| rs7318247 | 13 | 96814456 | A | G | 0.857815 | -0.0117863 | 0.00188863 | 4.39997E-10 | -0.0241 | 0.0134 | 0.0716407 | 8.43004E-05 | 38.94568385 |
| rs7319045 | 13 | 92024574 | G | A | 0.63062 | -0.0201419 | 0.00136073 | 1.39991E-49 | 0.0084 | 0.0096 | 0.3837 | 0.000474085 | 219.1064039 |
| rs73234767 | 3 | 142634502 | T | C | 0.170878 | -0.00991395 | 0.00174277 | 1.29999E-08 | 0.0127 | 0.0124 | 0.3064 | 7.00467E-05 | 32.36021642 |
| rs73453871 | 15 | 89261671 | A | G | 0.150849 | 0.013147 | 0.00183507 | 7.8001E-13 | 0.0353 | 0.013 | 0.00657204 | 0.000111098 | 51.32703473 |
| rs73471549 | 7 | 139910746 | A | G | 0.279317 | -0.00973791 | 0.00145875 | 2.49977E-11 | -0.0099 | 0.0104 | 0.3397 | 9.64568E-05 | 44.56233041 |
| rs734764 | 9 | 90852624 | C | G | 0.789266 | -0.019271 | 0.00160672 | 3.80014E-33 | 0.0143 | 0.0114 | 0.2103 | 0.000311313 | 143.855418 |
| rs737380 | 21 | 38077468 | G | A | 0.734692 | 0.00949177 | 0.00150143 | 2.59998E-10 | -0.0359 | 0.0105 | 0.000653898 | 8.65071E-05 | 39.96523338 |
| rs7430034 | 3 | 134342127 | T | C | 0.627668 | 0.0131989 | 0.00135124 | 1.50003E-22 | -0.0009 | 0.0096 | 0.9278 | 0.000206503 | 95.41323279 |
| rs743282 | 8 | 37336539 | T | C | 0.805607 | 0.00953801 | 0.00165392 | 8.10009E-09 | 0.0028 | 0.0117 | 0.8094 | 7.1988E-05 | 33.25712239 |
| rs74461473 | 3 | 50250747 | T | C | 0.112763 | -0.0199824 | 0.00208302 | 8.60003E-22 | -0.006 | 0.0148 | 0.6871 | 0.000199171 | 92.02515055 |
| rs74564500 | 2 | 183786420 | A | C | 0.078936 | 0.016023 | 0.00240421 | 2.70023E-11 | 0.0179 | 0.0172 | 0.2966 | 9.61404E-05 | 44.41615773 |
| rs745749 | 5 | 179715803 | G | A | 0.336509 | -0.0123212 | 0.00139026 | 7.8001E-19 | -0.0139 | 0.0099 | 0.1599 | 0.000169999 | 78.54383075 |
| rs7469817 | 9 | 118296829 | C | G | 0.742898 | 0.0152418 | 0.00149813 | 2.60016E-24 | -0.0243 | 0.0106 | 0.0224802 | 0.000224017 | 103.5074561 |
| rs75076724 | 14 | 104014690 | C | T | 0.144591 | 0.0209625 | 0.00187952 | 6.90081E-29 | -0.0095 | 0.0133 | 0.4779 | 0.000269203 | 124.3914036 |
| rs7518201 | 1 | 203773096 | C | A | 0.147313 | 0.0136844 | 0.0018278 | 7.10068E-14 | -0.0195 | 0.0131 | 0.1363 | 0.000121324 | 56.05215242 |
| rs7539511 | 1 | 2862320 | T | G | 0.323974 | 0.00762629 | 0.00138553 | 3.69999E-08 | -0.0231 | 0.0099 | 0.0197102 | 6.558E-05 | 30.29652961 |
| rs75396144 | 6 | 56890083 | C | T | 0.154223 | -0.0109228 | 0.00180926 | 1.6E-09 | -0.0046 | 0.0129 | 0.719399 | 7.88927E-05 | 36.44719578 |
| rs754532 | 11 | 65747057 | A | G | 0.300697 | 0.0107081 | 0.00142517 | 5.79963E-14 | 0.0059 | 0.0101 | 0.5615 | 0.000122192 | 56.45333624 |
| rs7574116 | 2 | 134466301 | C | T | 0.147038 | 0.0140782 | 0.00183908 | 1.9002E-14 | 0.006 | 0.0131 | 0.646399 | 0.000126836 | 58.59912593 |
| rs7575825 | 2 | 199176823 | T | C | 0.200858 | -0.00969077 | 0.00167501 | 7.19996E-09 | 0.0031 | 0.012 | 0.7959 | 7.24528E-05 | 33.47186288 |
| rs757834 | 7 | 139717200 | C | T | 0.185712 | 0.0146988 | 0.00168871 | 3.19963E-18 | 0.0163 | 0.012 | 0.1744 | 0.000163979 | 75.76205233 |
| rs7585767 | 2 | 70338344 | G | A | 0.489682 | -0.0104754 | 0.00131296 | 1.50003E-15 | -0.0097 | 0.0094 | 0.2986 | 0.000137779 | 63.65556245 |
| rs7593100 | 2 | 183694499 | T | C | 0.080656 | 0.0142588 | 0.00238391 | 2.19999E-09 | -0.0294 | 0.017 | 0.0844501 | 7.74386E-05 | 35.77538893 |
| rs7594625 | 2 | 218293564 | G | T | 0.342396 | -0.0192116 | 0.00137205 | 1.50003E-44 | -0.0085 | 0.0098 | 0.3865 | 0.000424236 | 196.0583372 |
| rs7595271 | 2 | 105873193 | A | G | 0.143369 | -0.0126289 | 0.00192804 | 5.70033E-11 | 0.0165 | 0.0138 | 0.2299 | 9.28675E-05 | 42.90393135 |
| rs76147573 | 2 | 12174147 | G | A | 0.120241 | 0.0115846 | 0.00199481 | 6.29999E-09 | -0.014 | 0.0143 | 0.327 | 7.30016E-05 | 33.72540176 |
| rs76152268 | 2 | 216303813 | C | T | 0.015293 | -0.0474133 | 0.00550889 | 7.50067E-18 | -0.003 | 0.0388 | 0.9387 | 0.000160327 | 74.07476557 |
| rs7621604 | 3 | 61557718 | G | A | 0.444895 | -0.0186408 | 0.00131178 | 7.89951E-46 | -0.0102 | 0.0093 | 0.2753 | 0.000436941 | 201.9320262 |
| rs7625645 | 3 | 99299413 | A | G | 0.634163 | -0.014713 | 0.00135601 | 1.99986E-27 | 0.0169 | 0.0097 | 0.08041 | 0.000254783 | 117.7266552 |
| rs7627625 | 3 | 168899725 | C | G | 0.046448 | 0.0200832 | 0.00311015 | 1.09999E-10 | -0.0118 | 0.0223 | 0.5971 | 9.02545E-05 | 41.69666071 |
| rs76292187 | 19 | 42754403 | A | G | 0.065409 | -0.0252743 | 0.00267302 | 3.19963E-21 | -0.0261 | 0.0189 | 0.1681 | 0.000193497 | 89.40297599 |
| rs76325149 | 7 | 100389590 | T | C | 0.062127 | -0.0147584 | 0.00270152 | 4.70002E-08 | -0.0028 | 0.0193 | 0.8851 | 6.4601E-05 | 29.84422458 |
| rs7632937 | 3 | 14169079 | C | T | 0.037952 | -0.0239391 | 0.00340264 | 1.99986E-12 | -0.01 | 0.0243 | 0.6798 | 0.000107138 | 49.49732757 |
| rs76364830 | 8 | 13372120 | A | G | 0.063402 | -0.0310013 | 0.00270888 | 2.49977E-30 | 0.0063 | 0.0193 | 0.744399 | 0.000283441 | 130.9719814 |
| rs7636734 | 3 | 37746851 | C | T | 0.543339 | 0.00839161 | 0.00131042 | 1.5E-10 | -0.0007 | 0.0093 | 0.9438 | 8.87638E-05 | 41.00791397 |
| rs7641322 | 3 | 145868489 | T | G | 0.545647 | -0.00837368 | 0.00130751 | 1.5E-10 | 0.0119 | 0.0093 | 0.2011 | 8.87788E-05 | 41.01481927 |
| rs7643462 | 3 | 11645992 | C | T | 0.950715 | 0.0286771 | 0.00302548 | 2.60016E-21 | 0.0214 | 0.0217 | 0.3223 | 0.000194448 | 89.84212354 |
| rs7651940 | 3 | 169256240 | T | C | 0.510609 | -0.0111481 | 0.00130751 | 1.50003E-17 | -0.0048 | 0.0093 | 0.603 | 0.000157343 | 72.69587914 |
| rs7652117 | 3 | 58008093 | C | T | 0.641789 | -0.0140967 | 0.00136017 | 3.59998E-25 | -0.0333 | 0.0097 | 0.000589006 | 0.000232462 | 107.4104784 |
| rs7652177 | 3 | 171969077 | G | C | 0.504695 | 0.025765 | 0.00130271 | 4.60045E-87 | -0.0037 | 0.0093 | 0.690601 | 0.000846062 | 391.1676396 |
| rs76602912 | 20 | 57459868 | C | T | 0.023799 | -0.0410432 | 0.00429997 | 1.39991E-21 | 0.034 | 0.0305 | 0.2652 | 0.000197184 | 91.10656995 |
| rs768023 | 6 | 108876002 | A | G | 0.626535 | 0.0159756 | 0.00133919 | 8.30042E-33 | 0.0244 | 0.0096 | 0.0110999 | 0.000307965 | 142.3075954 |
| rs7681267 | 4 | 87073599 | C | T | 0.983245 | 0.0415054 | 0.00512247 | 5.40008E-16 | 0.007 | 0.0362 | 0.8462 | 0.0001421 | 65.65207845 |
| rs76887969 | 9 | 99224962 | G | C | 0.079384 | 0.0304853 | 0.00245995 | 2.90001E-35 | -0.003 | 0.0175 | 0.8626 | 0.000332345 | 153.5771155 |
| rs7689420 | 4 | 145568352 | C | T | 0.831059 | 0.0595938 | 0.00173885 | 1E-200 | 0.0131 | 0.0124 | 0.2887 | 0.00253618 | 1174.56225 |
| rs76895963 | 12 | 4384844 | G | T | 0.020685 | 0.12465 | 0.00505065 | 1.7989E-134 | 0.0205 | 0.0353 | 0.562101 | 0.001316809 | 609.0993524 |
| rs7697556 | 4 | 73515313 | C | T | 0.526012 | -0.0233709 | 0.00130373 | 7.39946E-72 | -0.0024 | 0.0093 | 0.792301 | 0.00069515 | 321.3465752 |
| rs7712162 | 5 | 78945171 | T | C | 0.174492 | 0.0142116 | 0.00171927 | 1.39991E-16 | -0.0308 | 0.0122 | 0.0118201 | 0.00014789 | 68.3275581 |
| rs77124518 | 15 | 38362264 | C | T | 0.109488 | 0.0159106 | 0.00209542 | 3.10027E-14 | 0.0191 | 0.0149 | 0.2006 | 0.000124791 | 57.65394974 |
| rs77303550 | 16 | 72079657 | T | C | 0.191991 | 0.0113673 | 0.00166836 | 9.49948E-12 | -0.0002 | 0.0118 | 0.9833 | 0.000100484 | 46.42300249 |
| rs77330849 | 12 | 133374905 | T | C | 0.13875 | 0.0130477 | 0.00189255 | 5.40008E-12 | -0.0092 | 0.0134 | 0.4957 | 0.000102881 | 47.53038375 |
| rs7753558 | 6 | 117523471 | A | C | 0.633931 | 0.0188536 | 0.0013532 | 4.00037E-44 | 0.0016 | 0.0097 | 0.8723 | 0.000420037 | 194.1166193 |
| rs77672559 | 17 | 28135103 | T | A | 0.110107 | -0.0210762 | 0.00209714 | 9.20026E-24 | -0.0376 | 0.0148 | 0.01104 | 0.000218595 | 101.0015164 |
| rs778407 | 1 | 56704424 | T | G | 0.228021 | 0.013751 | 0.00154409 | 5.30029E-19 | -0.0055 | 0.0111 | 0.6179 | 0.000171654 | 79.30881542 |
| rs7794796 | 7 | 150540196 | T | C | 0.332215 | 0.0198123 | 0.00139517 | 9.09913E-46 | -0.0367 | 0.0099 | 0.000219801 | 0.000436346 | 201.6571627 |
| rs78051210 | 6 | 131379491 | C | T | 0.077083 | 0.030651 | 0.00244955 | 6.29941E-36 | 0.0144 | 0.0175 | 0.4124 | 0.000338824 | 156.5722522 |
| rs7814941 | 8 | 130718859 | G | A | 0.203034 | -0.0355956 | 0.00162687 | 3.9995E-106 | 0.0093 | 0.0115 | 0.4216 | 0.001035243 | 478.7238383 |
| rs7818782 | 8 | 96420039 | T | C | 0.165363 | -0.0116084 | 0.00176613 | 4.90004E-11 | 0.0398 | 0.0125 | 0.001461 | 9.35112E-05 | 43.20136941 |
| rs78249916 | 4 | 12740962 | T | C | 0.054344 | 0.0263778 | 0.00287776 | 4.90004E-20 | 0.0308 | 0.0205 | 0.133 | 0.000181842 | 84.01679654 |
| rs78264848 | 5 | 121191166 | A | G | 0.042578 | 0.0188139 | 0.00323524 | 6.1E-09 | 0.0297 | 0.023 | 0.1977 | 7.32011E-05 | 33.81759941 |
| rs78306761 | 20 | 7385054 | A | G | 0.17084 | -0.00995912 | 0.00174086 | 1.09999E-08 | -0.0083 | 0.0123 | 0.499501 | 7.08416E-05 | 32.72746426 |
| rs78428113 | 17 | 79115880 | T | C | 0.142728 | 0.0122228 | 0.00189438 | 1.09999E-10 | 0.0038 | 0.0134 | 0.7755 | 9.011E-05 | 41.62989647 |
| rs7847059 | 9 | 35901731 | A | T | 0.455897 | -0.0118416 | 0.0013184 | 2.70023E-19 | -0.0278 | 0.0094 | 0.00292799 | 0.000174605 | 80.67231221 |
| rs7847753 | 9 | 4163904 | C | T | 0.309435 | 0.00812682 | 0.0014222 | 1.09999E-08 | -0.0225 | 0.0101 | 0.0254501 | 7.06796E-05 | 32.65262138 |
| rs7861226 | 9 | 123351121 | A | C | 0.287606 | 0.0103954 | 0.00145559 | 9.20026E-13 | -0.0024 | 0.0103 | 0.8188 | 0.000110398 | 51.00378463 |
| rs78805413 | 11 | 2131018 | A | G | 0.059254 | -0.0319534 | 0.00284095 | 2.39994E-29 | -0.0108 | 0.0203 | 0.5935 | 0.000273775 | 126.5042427 |
| rs78817479 | 9 | 17040371 | A | C | 0.084061 | -0.0202205 | 0.00236668 | 1.29987E-17 | -0.0166 | 0.0167 | 0.3194 | 0.000157994 | 72.99663139 |
| rs7896518 | 10 | 65104500 | G | A | 0.427889 | 0.011941 | 0.0013367 | 4.10015E-19 | -0.0023 | 0.0095 | 0.8085 | 0.00017272 | 79.80160393 |
| rs7911018 | 10 | 131348554 | T | C | 0.609537 | 0.0104926 | 0.00134291 | 5.60015E-15 | -0.0046 | 0.0095 | 0.6275 | 0.000132136 | 61.04787313 |
| rs7916821 | 10 | 69933969 | A | G | 0.494018 | 0.0172636 | 0.00130828 | 9.30037E-40 | -0.0166 | 0.0093 | 0.0726708 | 0.000376793 | 174.1243246 |
| rs7928703 | 11 | 29080664 | G | T | 0.492074 | -0.00854464 | 0.00131418 | 7.89951E-11 | 0.0272 | 0.0093 | 0.00353199 | 9.15046E-05 | 42.27425399 |
| rs7943920 | 11 | 12695614 | G | A | 0.4658 | 0.0157165 | 0.00131352 | 5.40008E-33 | -0.025 | 0.0093 | 0.00715501 | 0.00030982 | 143.1648537 |
| rs79449299 | 4 | 38615469 | T | C | 0.11834 | -0.0168227 | 0.00201895 | 7.89951E-17 | 0.0291 | 0.0144 | 0.0436797 | 0.000150273 | 69.42859759 |
| rs7946010 | 11 | 17176057 | A | G | 0.489136 | -0.0115729 | 0.00130728 | 8.49963E-19 | 0.0086 | 0.0093 | 0.3529 | 0.000169621 | 78.36917712 |
| rs7952436 | 11 | 67024534 | T | C | 0.081991 | -0.0736727 | 0.0023849 | 1E-200 | -0.0466 | 0.0168 | 0.00554498 | 0.002061492 | 954.2692849 |
| rs7957659 | 12 | 50638810 | C | T | 0.578277 | -0.0113315 | 0.00131922 | 8.69961E-18 | 0.0075 | 0.0094 | 0.4211 | 0.000159689 | 73.77996967 |
| rs79584445 | 14 | 88893418 | C | T | 0.01371 | -0.0341759 | 0.00573526 | 2.5E-09 | -0.0322 | 0.0404 | 0.4257 | 7.68609E-05 | 35.50845846 |
| rs79621178 | 5 | 108091191 | C | G | 0.083798 | 0.0306612 | 0.00235022 | 6.70039E-39 | -0.0136 | 0.0168 | 0.416 | 0.000368304 | 170.1999312 |
| rs7964039 | 12 | 39736740 | T | C | 0.068704 | 0.0144131 | 0.00258546 | 0.000000025 | -0.0055 | 0.0184 | 0.7635 | 6.7269E-05 | 31.07687015 |
| rs7971877 | 12 | 58287630 | G | T | 0.434851 | -0.0125179 | 0.00132449 | 3.40017E-21 | 0.0126 | 0.0094 | 0.1808 | 0.000193324 | 89.32308375 |
| rs79728014 | 11 | 46062245 | G | A | 0.149156 | 0.0207825 | 0.00184179 | 1.59993E-29 | 0.0163 | 0.013 | 0.2103 | 0.00027555 | 127.324939 |
| rs79747671 | 12 | 103140344 | T | C | 0.107873 | -0.0253417 | 0.00209995 | 1.59993E-33 | -0.0148 | 0.0149 | 0.3215 | 0.000315153 | 145.6302858 |
| rs79749090 | 17 | 27417209 | G | A | 0.07255 | 0.0161666 | 0.0025581 | 2.59998E-10 | 0.0186 | 0.0181 | 0.3032 | 8.6451E-05 | 39.93930197 |
| rs7976738 | 12 | 91495838 | C | T | 0.84966 | -0.0104259 | 0.00182351 | 1.09999E-08 | 0.0061 | 0.013 | 0.6383 | 7.07596E-05 | 32.68954918 |
| rs7978217 | 12 | 20630869 | G | T | 0.614917 | 0.00816856 | 0.00134396 | 1.2E-09 | 0.0373 | 0.0652 | 0.5671 | 7.99629E-05 | 36.94166284 |
| rs7978217 | 12 | 20630869 | G | T | 0.614917 | 0.00816856 | 0.00134396 | 1.2E-09 | 0.0005 | 0.0096 | 0.9607 | 7.99629E-05 | 36.94166284 |
| rs798490 | 7 | 2801542 | A | G | 0.293148 | -0.0415024 | 0.00143369 | 2.9992E-184 | -0.0055 | 0.0102 | 0.5926 | 0.001810731 | 837.9807289 |
| rs7987131 | 13 | 99593241 | A | G | 0.712426 | -0.00833188 | 0.00146657 | 1.29999E-08 | 0.0134 | 0.0103 | 0.1954 | 6.98643E-05 | 32.27591319 |
| rs8016947 | 14 | 35832666 | G | T | 0.561817 | 0.00785152 | 0.00132247 | 2.90001E-09 | -0.0159 | 0.0093 | 0.0899001 | 7.6297E-05 | 35.24795655 |
| rs8029053 | 15 | 89450087 | T | C | 0.2843 | -0.0142942 | 0.00145498 | 8.80035E-23 | 0.0015 | 0.0103 | 0.8833 | 0.000208891 | 96.51699261 |
| rs8033211 | 15 | 63061887 | G | C | 0.677733 | 0.00824942 | 0.00140269 | 4.09996E-09 | -0.0049 | 0.01 | 0.625801 | 7.48679E-05 | 34.58768926 |
| rs803804 | 13 | 71599302 | T | C | 0.637542 | 0.00807584 | 0.00138785 | 5.89997E-09 | -0.0023 | 0.0098 | 0.8137 | 7.32932E-05 | 33.86011839 |
| rs8094658 | 18 | 2761338 | G | A | 0.351911 | 0.0078789 | 0.00137869 | 1.09999E-08 | 0.0129 | 0.0097 | 0.1855 | 7.06923E-05 | 32.65848584 |
| rs811133 | 14 | 54647436 | C | T | 0.811166 | 0.00941763 | 0.0016797 | 0.000000021 | -0.0225 | 0.0119 | 0.0580203 | 6.80449E-05 | 31.43532271 |
| rs822530 | 7 | 148631555 | T | A | 0.795351 | 0.0341081 | 0.00162826 | 1.99986E-97 | -0.0121 | 0.0116 | 0.2957 | 0.000948987 | 438.799038 |
| rs823118 | 1 | 205723572 | T | C | 0.547645 | -0.0151059 | 0.00130196 | 4.00037E-31 | 0.0034 | 0.0093 | 0.712901 | 0.000291324 | 134.6158033 |
| rs832806 | 2 | 219473914 | T | A | 0.565841 | 0.0212611 | 0.00131021 | 3.19963E-59 | 0.0067 | 0.0094 | 0.4716 | 0.000569701 | 263.3223731 |
| rs833149 | 2 | 183223013 | C | T | 0.576576 | -0.00763082 | 0.00131427 | 6.4E-09 | -0.0151 | 0.0094 | 0.1084 | 7.29704E-05 | 33.71097776 |
| rs835118 | 5 | 14956699 | A | G | 0.702214 | 0.00888568 | 0.00143178 | 5.39995E-10 | 0.013 | 0.0102 | 0.2042 | 8.33677E-05 | 38.51473148 |
| rs852946 | 6 | 72165420 | A | G | 0.421049 | 0.00915594 | 0.00131419 | 3.19963E-12 | 0.0087 | 0.0094 | 0.3558 | 0.000105063 | 48.53864846 |
| rs855286 | 12 | 102946454 | C | T | 0.907384 | -0.0218329 | 0.00225724 | 4.00037E-22 | -0.0088 | 0.0161 | 0.584901 | 0.000202481 | 93.55467524 |
| rs876122 | 6 | 6886297 | G | A | 0.87882 | 0.0251734 | 0.00198561 | 7.8001E-37 | 0.0655 | 0.0142 | 4.21299E-06 | 0.000347816 | 160.7288992 |
| rs883668 | 3 | 114554021 | G | A | 0.939946 | -0.0249074 | 0.00274041 | 1E-19 | 0.0203 | 0.0198 | 0.3056 | 0.000178794 | 82.60832631 |
| rs893900 | 15 | 67014528 | T | C | 0.547507 | -0.0160018 | 0.00131622 | 5.19996E-34 | 0.0145 | 0.0093 | 0.1202 | 0.00031985 | 147.8014942 |
| rs927578 | 13 | 28075022 | T | G | 0.142236 | 0.0108078 | 0.0018846 | 9.80009E-09 | 0.005 | 0.0133 | 0.7067 | 7.11886E-05 | 32.88776382 |
| rs9300607 | 13 | 101223459 | C | A | 0.480638 | -0.00852662 | 0.00131829 | 9.8992E-11 | 0.0125 | 0.0093 | 0.1773 | 9.05519E-05 | 41.83406061 |
| rs9314323 | 8 | 26243136 | G | A | 0.698375 | 0.0105859 | 0.00143187 | 1.39991E-13 | -0.008 | 0.0102 | 0.4296 | 0.000118305 | 54.65709104 |
| rs933561 | 16 | 49874676 | G | A | 0.346754 | -0.0119191 | 0.00138043 | 5.90065E-18 | -0.0072 | 0.0098 | 0.4593 | 0.000161359 | 74.55147987 |
| rs9345893 | 6 | 67512311 | T | G | 0.291623 | -0.00819179 | 0.00142981 | 0.00000001 | 0.0064 | 0.0102 | 0.53 | 7.10518E-05 | 32.82457262 |
| rs9350850 | 6 | 81050236 | C | T | 0.079527 | 0.0401944 | 0.00239466 | 3.10027E-63 | -0.0399 | 0.0171 | 0.0194702 | 0.000609514 | 281.7354524 |
| rs9352895 | 6 | 81590856 | A | T | 0.420123 | -0.0188992 | 0.00131174 | 4.60045E-47 | 0.0025 | 0.0094 | 0.787501 | 0.00044916 | 207.5818784 |
| rs9365939 | 6 | 166336825 | G | A | 0.547807 | -0.0117754 | 0.00129998 | 1.29987E-19 | 0.0013 | 0.0093 | 0.8857 | 0.000177585 | 82.04953336 |
| rs9379084 | 6 | 7231843 | A | G | 0.115607 | -0.031594 | 0.00208829 | 1E-51 | -0.0308 | 0.0149 | 0.0391796 | 0.000495242 | 228.8894258 |
| rs9391254 | 6 | 105377347 | T | C | 0.320391 | 0.035681 | 0.00138798 | 9.7051E-146 | 0.0007 | 0.0099 | 0.9407 | 0.001428538 | 660.8543274 |
| rs9405064 | 6 | 31292582 | C | A | 0.357581 | -0.0123203 | 0.0016344 | 4.79954E-14 | 0.0024 | 0.0117 | 0.8394 | 0.000122992 | 56.82297642 |
| rs941346 | 7 | 70254674 | T | G | 0.623244 | -0.00792309 | 0.00135098 | 4.49997E-09 | 0.0073 | 0.0096 | 0.4476 | 7.44499E-05 | 34.39453629 |
| rs9435731 | 1 | 17306029 | A | C | 0.519102 | 0.028591 | 0.00129754 | 1.3002E-107 | -0.0028 | 0.0093 | 0.759899 | 0.001049944 | 485.5291409 |
| rs9442571 | 1 | 9349611 | A | T | 0.129559 | 0.0254415 | 0.0019433 | 3.69999E-39 | 0.0122 | 0.0139 | 0.3798 | 0.000370894 | 171.3972481 |
| rs9443189 | 6 | 76495882 | G | A | 0.144701 | -0.013989 | 0.00184267 | 3.19963E-14 | 0.0053 | 0.0132 | 0.6883 | 0.000124747 | 57.63367717 |
| rs9487084 | 6 | 109717088 | G | T | 0.410796 | -0.0174405 | 0.00131831 | 5.90065E-40 | -0.001 | 0.0094 | 0.9183 | 0.000378724 | 175.0172638 |
| rs9493589 | 6 | 133632465 | A | G | 0.526621 | -0.0113287 | 0.00130049 | 2.99985E-18 | 0.004 | 0.0093 | 0.6711 | 0.00016424 | 75.88295559 |
| rs9496369 | 6 | 142724918 | T | C | 0.286031 | -0.0395896 | 0.00143389 | 8.4918E-168 | 0.0034 | 0.0103 | 0.738901 | 0.001647478 | 762.3048369 |
| rs9535015 | 13 | 48904509 | A | C | 0.721237 | -0.0115236 | 0.00146705 | 4.00037E-15 | 0.005 | 0.0104 | 0.6265 | 0.000133547 | 61.69992528 |
| rs9543310 | 13 | 73859958 | A | G | 0.305172 | 0.00852784 | 0.00142675 | 2.30001E-09 | -0.0014 | 0.0101 | 0.8856 | 7.7331E-05 | 35.7256751 |
| rs9545588 | 13 | 81615536 | A | G | 0.61999 | -0.00850337 | 0.00136365 | 4.49997E-10 | 0.0112 | 0.0096 | 0.2435 | 8.41676E-05 | 38.8843138 |
| rs9574556 | 13 | 80646366 | C | G | 0.206141 | 0.0115622 | 0.00162978 | 1.29987E-12 | 0.0105 | 0.0115 | 0.3616 | 0.000108938 | 50.32932508 |
| rs9575875 | 13 | 85931285 | G | A | 0.376906 | 0.0101811 | 0.00135753 | 6.4003E-14 | 0.0078 | 0.0096 | 0.4158 | 0.000121743 | 56.24561301 |
| rs9576006 | 13 | 36944732 | A | G | 0.294658 | -0.00819473 | 0.00144735 | 0.000000015 | 0.0062 | 0.0102 | 0.5446 | 6.939E-05 | 32.05680879 |
| rs9576134 | 13 | 37481694 | G | C | 0.218449 | 0.00949455 | 0.00159466 | 2.59998E-09 | -0.0014 | 0.0113 | 0.9029 | 7.67334E-05 | 35.44954704 |
| rs9590405 | 13 | 114999838 | G | A | 0.235299 | -0.0182649 | 0.00154886 | 4.30031E-32 | 0.0097 | 0.0109 | 0.3774 | 0.000300943 | 139.0619738 |
| rs960225 | 11 | 1952089 | A | C | 0.104807 | 0.0163195 | 0.00214406 | 2.70023E-14 | -0.0265 | 0.0153 | 0.0831496 | 0.000125398 | 57.93459132 |
| rs9614670 | 22 | 45838817 | T | C | 0.200304 | 0.0169639 | 0.00164911 | 8.10028E-25 | -0.0183 | 0.0116 | 0.1156 | 0.000229012 | 105.8157408 |
| rs9634212 | 12 | 93993266 | A | C | 0.220674 | 0.0392212 | 0.00157834 | 2.6002E-136 | 0.0411 | 0.0112 | 0.000238199 | 0.001334952 | 617.5025393 |
| rs966541 | 12 | 29491528 | G | A | 0.276542 | 0.0148463 | 0.00145936 | 2.60016E-24 | -0.0005 | 0.0104 | 0.9622 | 0.000223985 | 103.4926976 |
| rs9696477 | 9 | 136399315 | T | C | 0.23296 | -0.0105483 | 0.00162374 | 8.19974E-11 | 0.0067 | 0.0114 | 0.5577 | 9.13476E-05 | 42.20171368 |
| rs9747062 | 17 | 8023057 | C | A | 0.600697 | 0.0137871 | 0.00133756 | 6.4998E-25 | 0.0015 | 0.0095 | 0.8713 | 0.000229945 | 106.2471834 |
| rs9809116 | 3 | 72397279 | G | A | 0.408494 | -0.0244981 | 0.00133454 | 2.90001E-75 | 0.0021 | 0.0095 | 0.8242 | 0.000728937 | 336.9765931 |
| rs9824877 | 3 | 98896242 | A | G | 0.222424 | -0.0104824 | 0.00163753 | 1.5E-10 | -0.0095 | 0.0116 | 0.4157 | 8.86971E-05 | 40.97708357 |
| rs9826470 | 3 | 38019428 | T | C | 0.248942 | 0.0167257 | 0.00151482 | 2.39994E-28 | -0.0147 | 0.0108 | 0.1727 | 0.000263838 | 121.9114971 |
| rs9830029 | 3 | 33262129 | A | G | 0.385234 | 0.0100338 | 0.00134179 | 7.50067E-14 | 0.0258 | 0.0096 | 0.00699407 | 0.000121036 | 55.91906463 |
| rs985344 | 3 | 67347767 | A | G | 0.104292 | -0.0188136 | 0.00214659 | 1.9002E-18 | 0.015 | 0.0154 | 0.3302 | 0.000166256 | 76.81459204 |
| rs9875575 | 3 | 88299112 | G | A | 0.757154 | 0.0086437 | 0.00152797 | 0.000000015 | 0.0202 | 0.0109 | 0.0631597 | 6.92699E-05 | 32.00131578 |
| rs9880232 | 3 | 185360578 | A | C | 0.701452 | 0.011601 | 0.00142982 | 4.90004E-16 | -0.0053 | 0.0102 | 0.6055 | 0.000142486 | 65.83036122 |
| rs9894127 | 17 | 59625479 | A | G | 0.271576 | 0.0134548 | 0.00147042 | 5.70033E-20 | -0.0161 | 0.0104 | 0.1222 | 0.000181217 | 83.72782516 |
| rs9894946 | 17 | 7571080 | G | A | 0.841046 | 0.0120306 | 0.00181899 | 3.69999E-11 | -0.0066 | 0.0129 | 0.6111 | 9.46843E-05 | 43.74335321 |
| rs9941239 | 16 | 84987947 | G | A | 0.755802 | -0.0147342 | 0.00153182 | 6.70039E-22 | -0.0252 | 0.0108 | 0.0201201 | 0.000200242 | 92.52003106 |
| rs9976812 | 21 | 39690245 | G | C | 0.56377 | -0.0184561 | 0.00133896 | 3.19963E-43 | 0.0089 | 0.0094 | 0.3466 | 0.000411122 | 189.9952672 |
| Genome-wide significant SNPs for Essential (primary) hypertension | | | | | | | | | | | | | |
| SNP | Chr | Position | EA | OA | EAF | Essential (primary) hypertension | | | Knee Osteoarthritis | | | R2 | F |
|  |  |  |  |  |  | beta | SE | pval | beta | SE | pval |  |  |
| rs10245376 | 7 | 7272368 | T | G | 0.155336 | 0.00580593 | 0.000919677 | 2.69998E-10 | 0.007 | 0.0128 | 0.5844 | 8.60687E-05 | 39.85392177 |
| rs10749409 | 10 | 122976566 | G | C | 0.684325 | -0.00473099 | 0.000717266 | 4.19952E-11 | -0.013 | 0.01 | 0.1943 | 9.39534E-05 | 43.5052526 |
| rs1077394 | 6 | 31610384 | T | C | 0.672304 | 0.0041629 | 0.000709324 | 4.39997E-09 | 0.0256 | 0.0099 | 0.00973711 | 7.43841E-05 | 34.44298418 |
| rs10804330 | 2 | 227185749 | C | T | 0.431675 | -0.00405057 | 0.00067765 | 2.30001E-09 | -0.0062 | 0.0094 | 0.510199 | 7.71609E-05 | 35.72888986 |
| rs11191559 | 10 | 104867686 | T | C | 0.07762 | -0.0077192 | 0.00124218 | 5.19996E-10 | 0.0162 | 0.0173 | 0.351 | 8.33967E-05 | 38.61656551 |
| rs11604462 | 11 | 65551648 | A | G | 0.343347 | 0.00441834 | 0.000700185 | 2.80001E-10 | 0.0202 | 0.0098 | 0.0383902 | 8.59934E-05 | 39.81903987 |
| rs11801879 | 1 | 11928819 | C | T | 0.088418 | -0.00760518 | 0.00117836 | 1.09999E-10 | -0.0141 | 0.0165 | 0.391 | 8.99567E-05 | 41.65442419 |
| rs12258967 | 10 | 18727959 | G | C | 0.299316 | -0.00503873 | 0.000727902 | 4.40048E-12 | 0.0155 | 0.0101 | 0.1266 | 0.000103481 | 47.91753551 |
| rs12263737 | 10 | 96044913 | A | G | 0.271095 | -0.00440961 | 0.000748548 | 3.79997E-09 | -0.0178 | 0.0105 | 0.0897305 | 7.49442E-05 | 34.70237327 |
| rs12360772 | 11 | 1899962 | A | G | 0.186914 | 0.00580045 | 0.000859047 | 1.50003E-11 | -0.0103 | 0.012 | 0.391 | 9.84592E-05 | 45.59189545 |
| rs12656497 | 5 | 32831939 | C | T | 0.596328 | 0.0053786 | 0.000678572 | 2.29985E-15 | -0.0126 | 0.0095 | 0.183 | 0.000135674 | 62.82677228 |
| rs1275985 | 2 | 26911745 | T | C | 0.617253 | -0.00621895 | 0.000685911 | 1.20005E-19 | -0.0127 | 0.0096 | 0.1827 | 0.000177513 | 82.20471327 |
| rs12762222 | 10 | 107289232 | C | T | 0.01942 | 0.0134877 | 0.00244222 | 3.29997E-08 | 0.0531 | 0.0336 | 0.1146 | 6.58699E-05 | 30.50031694 |
| rs12932686 | 16 | 51759252 | C | T | 0.413548 | 0.00389659 | 0.000677037 | 8.60003E-09 | -0.0053 | 0.0094 | 0.5718 | 7.15358E-05 | 33.12399728 |
| rs13125101 | 4 | 81174592 | A | G | 0.29184 | 0.00955196 | 0.000733784 | 9.70063E-39 | -0.0148 | 0.0102 | 0.1478 | 0.000365847 | 169.4519764 |
| rs1327235 | 20 | 10969030 | G | A | 0.476253 | 0.00429115 | 0.000667351 | 1.29999E-10 | 0.0006 | 0.0093 | 0.9495 | 8.92914E-05 | 41.34632219 |
| rs162395 | 21 | 44945759 | C | T | 0.571316 | 0.00387748 | 0.000672543 | 8.10009E-09 | 0.0091 | 0.0094 | 0.3304 | 7.17856E-05 | 33.23970366 |
| rs167479 | 19 | 11526765 | T | G | 0.472463 | -0.0058173 | 0.000666621 | 2.60016E-18 | 0.0011 | 0.0093 | 0.902 | 0.000164446 | 76.15230701 |
| rs16948048 | 17 | 47440466 | G | A | 0.366921 | 0.00429544 | 0.000691113 | 5.1E-10 | 0.0101 | 0.0096 | 0.2952 | 8.34239E-05 | 38.62916438 |
| rs17035646 | 1 | 10796547 | A | G | 0.337161 | 0.00607011 | 0.000708376 | 1E-17 | 0.0076 | 0.0099 | 0.4436 | 0.000158565 | 73.42831614 |
| rs17558745 | 1 | 218548521 | T | C | 0.311877 | 0.00399348 | 0.000721776 | 0.000000032 | 0.0146 | 0.0101 | 0.1453 | 6.61118E-05 | 30.61232232 |
| rs1870735 | 7 | 155744303 | G | C | 0.548152 | -0.00368047 | 0.000674799 | 4.90004E-08 | -0.0133 | 0.0094 | 0.1559 | 6.4245E-05 | 29.74786676 |
| rs1918898 | 2 | 188100639 | T | C | 0.356125 | -0.00396663 | 0.000697055 | 1.29999E-08 | 0.0007 | 0.0097 | 0.9407 | 6.9934E-05 | 32.38227934 |
| rs2003476 | 19 | 18806668 | C | T | 0.405754 | -0.00399459 | 0.000682021 | 4.70002E-09 | -0.0127 | 0.0095 | 0.1819 | 7.40843E-05 | 34.30418064 |
| rs2643826 | 3 | 27562988 | T | C | 0.45231 | 0.00493899 | 0.000670806 | 1.80011E-13 | 0.0063 | 0.0093 | 0.4997 | 0.000117069 | 54.21014148 |
| rs2728624 | 12 | 20155052 | A | G | 0.226934 | -0.00458701 | 0.00079658 | 8.50002E-09 | -0.0011 | 0.0111 | 0.9225 | 7.16109E-05 | 33.15879207 |
| rs2759315 | 15 | 81009646 | A | C | 0.44312 | 0.00471393 | 0.000670907 | 2.09991E-12 | 0.01 | 0.0093 | 0.284 | 0.000106612 | 49.36734099 |
| rs3184504 | 12 | 111884608 | C | T | 0.517267 | -0.0060921 | 0.000665312 | 5.30029E-20 | 0.0057 | 0.0093 | 0.5387 | 0.000181057 | 83.84582796 |
| rs346078 | 3 | 11327840 | C | G | 0.378129 | 0.00387229 | 0.000687829 | 1.79999E-08 | -0.0003 | 0.0096 | 0.9759 | 6.8447E-05 | 31.6936975 |
| rs35184780 | 17 | 27928622 | G | C | 0.438777 | 0.00389689 | 0.000680343 | 0.00000001 | -0.0068 | 0.0095 | 0.473 | 7.08532E-05 | 32.80791086 |
| rs35443 | 12 | 115552878 | C | G | 0.381805 | -0.00495279 | 0.000684503 | 4.60045E-13 | -0.008 | 0.0095 | 0.3985 | 0.00011306 | 52.35368318 |
| rs3735533 | 7 | 27245893 | C | T | 0.926702 | 0.00877999 | 0.0012761 | 6.00067E-12 | -0.0004 | 0.0177 | 0.9823 | 0.000102231 | 47.33874599 |
| rs3790604 | 1 | 113046879 | A | C | 0.073159 | 0.00908223 | 0.00128186 | 1.39991E-12 | 0.0116 | 0.0178 | 0.5158 | 0.000108409 | 50.19979276 |
| rs3796581 | 4 | 156642884 | G | A | 0.183958 | -0.00574699 | 0.000859728 | 2.29985E-11 | -0.0037 | 0.012 | 0.758901 | 9.64998E-05 | 44.68449606 |
| rs3821843 | 3 | 53558012 | A | G | 0.678977 | 0.0046448 | 0.000724649 | 1.5E-10 | 0.0127 | 0.0101 | 0.207 | 8.87258E-05 | 41.08438275 |
| rs3918226 | 7 | 150690176 | T | C | 0.081012 | 0.0101555 | 0.00123821 | 2.39994E-16 | -0.0455 | 0.0173 | 0.00837799 | 0.000145265 | 67.26855997 |
| rs4291 | 17 | 61554194 | A | T | 0.623299 | -0.0039207 | 0.000690852 | 1.40001E-08 | -0.0091 | 0.0096 | 0.3454 | 6.95563E-05 | 32.2073736 |
| rs4412193 | 6 | 26338056 | G | A | 0.366882 | -0.00472 | 0.000692322 | 9.30037E-12 | -0.0376 | 0.0097 | 9.63208E-05 | 0.000100377 | 46.47997185 |
| rs55670730 | 11 | 43620008 | T | A | 0.110744 | 0.0060027 | 0.00107194 | 0.000000021 | 0.0313 | 0.0149 | 0.0361701 | 6.77224E-05 | 31.35815104 |
| rs55730499 | 6 | 161005610 | T | C | 0.079714 | 0.0077937 | 0.00123046 | 2.39999E-10 | -0.0156 | 0.0171 | 0.3598 | 8.66412E-05 | 40.11903637 |
| rs56094641 | 16 | 53806453 | G | A | 0.404625 | 0.0040277 | 0.00067826 | 2.90001E-09 | 0.0429 | 0.0095 | 5.58496E-06 | 7.6155E-05 | 35.26305569 |
| rs56273825 | 5 | 110840889 | C | T | 0.021882 | -0.0134791 | 0.00240449 | 0.000000021 | -0.013 | 0.0337 | 0.7008 | 6.78666E-05 | 31.42490433 |
| rs568546 | 11 | 107321156 | T | C | 0.521082 | -0.00522564 | 0.000668889 | 5.60015E-15 | -0.0105 | 0.0093 | 0.2588 | 0.000131802 | 61.03360054 |
| rs57139556 | 6 | 150998511 | G | A | 0.071572 | -0.00772819 | 0.00129089 | 2.1E-09 | 0.0115 | 0.018 | 0.5234 | 7.74022E-05 | 35.84059679 |
| rs6026744 | 20 | 57742388 | T | A | 0.119013 | 0.00835569 | 0.00103214 | 5.70033E-16 | -0.0055 | 0.0143 | 0.6987 | 0.000141526 | 65.53684704 |
| rs6031435 | 20 | 42797358 | G | A | 0.459301 | 0.00418812 | 0.000671774 | 4.49997E-10 | -0.0103 | 0.0094 | 0.2719 | 8.39392E-05 | 38.86780144 |
| rs6108171 | 20 | 8635551 | T | A | 0.247296 | -0.00749288 | 0.000775264 | 4.19952E-22 | -0.0103 | 0.0108 | 0.3416 | 0.000201707 | 93.41066061 |
| rs62089932 | 18 | 72724362 | T | C | 0.859954 | -0.00579903 | 0.00102685 | 0.000000016 | 0.0053 | 0.0143 | 0.711101 | 6.88773E-05 | 31.89295941 |
| rs633185 | 11 | 100593538 | C | G | 0.715077 | 0.00652925 | 0.00074143 | 1.29987E-18 | 0.0056 | 0.0103 | 0.5888 | 0.000167465 | 77.55046572 |
| rs6766859 | 3 | 138055136 | T | C | 0.626849 | -0.00404117 | 0.000691984 | 5.19996E-09 | -0.0153 | 0.0096 | 0.1129 | 7.36546E-05 | 34.10517316 |
| rs68096471 | 19 | 5175709 | A | G | 0.269191 | -0.00444907 | 0.000751777 | 3.29997E-09 | 0.0089 | 0.0105 | 0.3921 | 7.56375E-05 | 35.02341839 |
| rs6822044 | 4 | 26787745 | G | C | 0.347199 | -0.00421033 | 0.000700993 | 1.89998E-09 | -0.0067 | 0.0098 | 0.4948 | 7.79078E-05 | 36.07472547 |
| rs6866614 | 5 | 131787137 | G | A | 0.576646 | 0.00392113 | 0.000679224 | 7.79992E-09 | 0.0075 | 0.0095 | 0.4262 | 7.19739E-05 | 33.3268725 |
| rs6918911 | 6 | 133999752 | T | A | 0.079876 | -0.00824449 | 0.00127656 | 1.09999E-10 | -0.0283 | 0.0177 | 0.1106 | 9.00774E-05 | 41.71029401 |
| rs6961048 | 7 | 27328187 | G | C | 0.101304 | 0.00642496 | 0.00110368 | 5.80003E-09 | 0.0179 | 0.0154 | 0.2453 | 7.31867E-05 | 33.88852254 |
| rs6991641 | 8 | 10586860 | C | G | 0.598329 | -0.00468068 | 0.0006864 | 9.20026E-12 | -0.0323 | 0.0096 | 0.000715501 | 0.000100422 | 46.50091264 |
| rs72831345 | 10 | 63518748 | A | G | 0.145089 | -0.00958081 | 0.000944429 | 3.50026E-24 | -0.027 | 0.0132 | 0.03983 | 0.000222218 | 102.9115101 |
| rs7297416 | 12 | 54443090 | C | A | 0.297749 | -0.00401239 | 0.000728434 | 3.59998E-08 | 0.0012 | 0.0101 | 0.907 | 6.5525E-05 | 30.34058748 |
| rs740746 | 10 | 115792787 | A | G | 0.73319 | 0.00558046 | 0.000754909 | 1.39991E-13 | 0.0037 | 0.0105 | 0.7224 | 0.000118007 | 54.64480959 |
| rs7497304 | 15 | 91429176 | T | G | 0.325747 | 0.00586179 | 0.000709748 | 1.50003E-16 | -0.0188 | 0.0099 | 0.0575003 | 0.000147298 | 68.21035041 |
| rs7528118 | 1 | 56968755 | A | G | 0.240027 | 0.00437858 | 0.000783161 | 2.30001E-08 | 0.0083 | 0.0109 | 0.447 | 6.75064E-05 | 31.25810153 |
| rs76452347 | 9 | 35906471 | T | C | 0.204442 | -0.0051363 | 0.000857013 | 2.1E-09 | -0.0012 | 0.0119 | 0.9221 | 7.75714E-05 | 35.91898604 |
| rs7685862 | 4 | 111389101 | A | C | 0.795291 | -0.0047658 | 0.000825582 | 7.79992E-09 | 0.0008 | 0.0115 | 0.9466 | 7.19664E-05 | 33.32340096 |
| rs7700842 | 5 | 157824183 | C | T | 0.371105 | -0.00692396 | 0.000688685 | 8.80035E-24 | 0.0035 | 0.0096 | 0.7125 | 0.000218264 | 101.0801731 |
| rs7763350 | 6 | 43349308 | C | A | 0.322191 | 0.00448797 | 0.000711841 | 2.90001E-10 | 0.0108 | 0.0099 | 0.2752 | 8.58433E-05 | 39.74953179 |
| rs77924615 | 16 | 20392332 | A | G | 0.196675 | -0.00516325 | 0.000845642 | 0.000000001 | 0.0073 | 0.0117 | 0.5359 | 8.05096E-05 | 37.27960843 |
| rs8042127 | 15 | 41251512 | T | C | 0.479558 | 0.00391874 | 0.000669546 | 4.79999E-09 | 0.0107 | 0.0093 | 0.2496 | 7.39792E-05 | 34.25549001 |
| rs8118848 | 20 | 62461572 | A | G | 0.237633 | -0.0048929 | 0.000782966 | 4.09996E-10 | 0.0134 | 0.0109 | 0.2182 | 8.43373E-05 | 39.05215433 |
| rs9330353 | 4 | 138439630 | A | T | 0.417876 | 0.00460601 | 0.000676769 | 1E-11 | 0.0005 | 0.0094 | 0.9583 | 0.000100031 | 46.31983062 |
| rs9375459 | 6 | 127147704 | T | C | 0.437127 | 0.00620813 | 0.000670375 | 1.99986E-20 | -0.0245 | 0.0093 | 0.00866802 | 0.000185189 | 85.75986906 |
| Genome-wide significant SNPs for Age completed full time education | | | | | | | | | | | | | |
| SNP | Chr | Position | EA | OA | EAF | Age completed full time education | | | Knee Osteoarthritis | | | R2 | F |
|  |  |  |  |  |  | beta | SE | pval | beta | SE | pval |  |  |
| rs10189857 | 2 | 60713235 | G | A | 0.435409 | -0.0124054 | 0.00208474 | 2.69998E-09 | 0.0077 | 0.0094 | 0.4133 | 0.000114991 | 35.40910297 |
| rs10200379 | 2 | 144183008 | A | C | 0.519841 | 0.0139597 | 0.00207137 | 1.59993E-11 | -0.0137 | 0.0093 | 0.1414 | 0.000147492 | 45.41862532 |
| rs10760199 | 9 | 124614772 | T | G | 0.551851 | 0.0115963 | 0.00208033 | 0.000000025 | -0.0187 | 0.0093 | 0.0450101 | 0.000100908 | 31.07217107 |
| rs10786662 | 10 | 103989812 | C | G | 0.574335 | -0.0116835 | 0.00209243 | 2.39999E-08 | 0.0119 | 0.0094 | 0.2038 | 0.00010125 | 31.17749575 |
| rs10953765 | 7 | 114291435 | A | G | 0.550745 | -0.0130933 | 0.002104 | 4.90004E-10 | 0.0333 | 0.0094 | 0.000423799 | 0.000125761 | 38.72611631 |
| rs114408770 | 3 | 43873454 | A | G | 0.024888 | -0.0436574 | 0.00682815 | 1.6E-10 | 0.0492 | 0.0308 | 0.1095 | 0.000132754 | 40.87961609 |
| rs11665242 | 18 | 50907127 | G | A | 0.425576 | -0.0125523 | 0.00209622 | 2.1E-09 | 0.0186 | 0.0094 | 0.0480397 | 0.000116444 | 35.85668031 |
| rs13064576 | 3 | 49642430 | T | C | 0.294739 | 0.0172696 | 0.00226489 | 2.39994E-14 | -0.0209 | 0.0101 | 0.0388204 | 0.000188792 | 58.13899329 |
| rs13220159 | 6 | 30419343 | T | C | 0.04562 | 0.0297715 | 0.00498639 | 2.39999E-09 | 0.009 | 0.0223 | 0.6862 | 0.000115764 | 35.64725774 |
| rs13238996 | 7 | 74069645 | G | A | 0.242877 | -0.0133142 | 0.00240789 | 0.000000032 | 0.008 | 0.0108 | 0.4616 | 9.92906E-05 | 30.57412562 |
| rs13274119 | 8 | 143342967 | A | C | 0.13803 | 0.0198214 | 0.00301673 | 5.00035E-11 | -0.0083 | 0.0135 | 0.54 | 0.000140194 | 43.17108325 |
| rs13394374 | 2 | 62263120 | T | C | 0.030466 | 0.0335998 | 0.00611413 | 3.89996E-08 | -0.0111 | 0.0272 | 0.6821 | 9.80745E-05 | 30.19960249 |
| rs1462163 | 2 | 164333146 | T | C | 0.40086 | -0.0127222 | 0.00211969 | 1.89998E-09 | 0.0244 | 0.0095 | 0.0105 | 0.000116983 | 36.02275417 |
| rs1557341 | 18 | 35127427 | C | A | 0.663393 | 0.0129858 | 0.00220266 | 3.69999E-09 | -0.0121 | 0.0099 | 0.2237 | 0.000112872 | 34.75679067 |
| rs17563464 | 5 | 26913774 | A | C | 0.225571 | -0.0148391 | 0.00251248 | 3.50002E-09 | 0.0026 | 0.0113 | 0.8174 | 0.000113281 | 34.88245762 |
| rs178217 | 14 | 26939332 | C | T | 0.750378 | -0.0143669 | 0.00239746 | 2.1E-09 | 0.0002 | 0.0107 | 0.9826 | 0.000116618 | 35.91042762 |
| rs2588962 | 10 | 63606492 | A | G | 0.458738 | 0.0150825 | 0.00207972 | 4.10015E-13 | -0.0128 | 0.0093 | 0.1703 | 0.000170788 | 52.59374669 |
| rs2709814 | 4 | 152360870 | C | T | 0.568651 | -0.0114761 | 0.0020926 | 4.20001E-08 | 0.0071 | 0.0094 | 0.4467 | 9.76716E-05 | 30.07553661 |
| rs2857693 | 6 | 31588384 | T | G | 0.365317 | -0.0121507 | 0.00214204 | 1.40001E-08 | -0.0004 | 0.0097 | 0.9629 | 0.000104495 | 32.17693125 |
| rs34945223 | 18 | 77575871 | G | A | 0.278543 | -0.0136288 | 0.00230526 | 3.40001E-09 | 0.0055 | 0.0104 | 0.5991 | 0.000113506 | 34.95204465 |
| rs4557720 | 8 | 87932823 | G | A | 0.378226 | 0.0119687 | 0.00213393 | 0.00000002 | -0.0043 | 0.0096 | 0.6522 | 0.000102161 | 31.45797795 |
| rs4674403 | 2 | 220345117 | G | T | 0.718268 | -0.0127249 | 0.0023324 | 4.90004E-08 | 0.0132 | 0.0105 | 0.2054 | 9.66619E-05 | 29.7645876 |
| rs4731951 | 7 | 133102282 | T | G | 0.80058 | 0.0153215 | 0.00259236 | 3.40001E-09 | -0.0229 | 0.0117 | 0.0500599 | 0.000113438 | 34.93084781 |
| rs55771711 | 10 | 133802737 | C | G | 0.25549 | 0.0132681 | 0.00237707 | 2.39999E-08 | -0.0085 | 0.0106 | 0.4242 | 0.000101178 | 31.15521187 |
| rs57513571 | 17 | 2309130 | T | C | 0.200955 | -0.0153935 | 0.00258666 | 2.69998E-09 | -0.0219 | 0.0116 | 0.0587395 | 0.000115011 | 35.4154885 |
| rs62039529 | 16 | 15205984 | A | C | 0.837847 | 0.0165739 | 0.00284063 | 5.39995E-09 | -0.0077 | 0.0128 | 0.546799 | 0.000110552 | 34.04217254 |
| rs6449503 | 5 | 60095272 | A | G | 0.491513 | 0.0123947 | 0.00208193 | 2.59998E-09 | 0.0118 | 0.0093 | 0.2053 | 0.000115102 | 35.44353022 |
| rs6508344 | 18 | 22646735 | A | G | 0.571312 | -0.0115957 | 0.00208724 | 2.80001E-08 | -0.0143 | 0.0094 | 0.1253 | 0.000100231 | 30.86358301 |
| rs6679399 | 1 | 211093346 | C | A | 0.025961 | 0.0426608 | 0.00677016 | 2.99999E-10 | -0.0107 | 0.0301 | 0.722301 | 0.000128943 | 39.70610663 |
| rs6729586 | 2 | 44808475 | A | G | 0.599259 | -0.0117953 | 0.00211341 | 2.39999E-08 | 0.0011 | 0.0095 | 0.9091 | 0.000101158 | 31.14925365 |
| rs6754311 | 2 | 136707982 | C | T | 0.257831 | 0.0150378 | 0.002367 | 2.1E-10 | 0.0083 | 0.0109 | 0.4485 | 0.000131072 | 40.36168346 |
| rs68191270 | 1 | 44026180 | C | A | 0.778588 | 0.0166103 | 0.00248918 | 2.49977E-11 | -0.0073 | 0.0112 | 0.514301 | 0.000144602 | 44.52864973 |
| rs6931604 | 6 | 98578215 | T | C | 0.597011 | 0.0136265 | 0.0021131 | 1.09999E-10 | 0.0055 | 0.0095 | 0.559099 | 0.000135041 | 41.58394809 |
| rs7110786 | 11 | 95656648 | T | C | 0.418918 | 0.0127141 | 0.00209121 | 1.2E-09 | -0.0206 | 0.0094 | 0.0283602 | 0.000120038 | 36.96350374 |
| rs7335432 | 13 | 30908299 | G | C | 0.436974 | 0.0121373 | 0.00208869 | 6.19998E-09 | -0.0257 | 0.0094 | 0.00606904 | 0.000109659 | 33.76706993 |
| rs7768758 | 6 | 88251077 | C | T | 0.080585 | -0.0227349 | 0.00379371 | 2.1E-09 | 0.0266 | 0.0172 | 0.1203 | 0.000116628 | 35.91327548 |
| rs7896518 | 10 | 65104500 | G | A | 0.425249 | 0.0145245 | 0.00211298 | 6.20012E-12 | -0.0023 | 0.0095 | 0.8085 | 0.00015344 | 47.25075889 |
| rs7975763 | 12 | 123604053 | T | C | 0.203454 | 0.0164068 | 0.00257604 | 1.89998E-10 | -0.0594 | 0.0115 | 2.64198E-07 | 0.000131729 | 40.56390364 |
| rs9536961 | 13 | 55678332 | G | A | 0.346028 | 0.0139369 | 0.0022011 | 2.39999E-10 | 0.0003 | 0.0099 | 0.9794 | 0.000130194 | 40.0912871 |
| rs9655780 | 7 | 104667334 | A | G | 0.832553 | -0.0174579 | 0.00279646 | 4.30002E-10 | 0.0116 | 0.0125 | 0.3547 | 0.000126563 | 38.97301087 |
| rs9866630 | 3 | 78505146 | A | G | 0.504663 | -0.0126597 | 0.0020719 | 0.000000001 | 0.0183 | 0.0093 | 0.0487697 | 0.000121241 | 37.33416363 |
| Genome-wide significant SNPs for Years of schooling | | | | | | | | | | | | | |
| SNP | Chr | Position | EA | OA | EAF | Years of schooling | | | Knee Osteoarthritis | | | R2 | F |
|  |  |  |  |  |  | beta | SE | pval | beta | SE | pval |  |  |
| rs10073890 | 5 | 124288028 | G | A | 0.7364 | -0.01262 | 0.00196 | 1.10999E-10 | 0.0318 | 0.0106 | 0.00264801 | 5.40952E-05 | 41.45772187 |
| rs1008078 | 1 | 91189731 | T | C | 0.4099 | -0.01738 | 0.00173 | 1.20005E-23 | 0.007 | 0.0095 | 0.4586 | 0.000131682 | 100.9267305 |
| rs10189857 | 2 | 60713235 | G | A | 0.4184 | -0.01725 | 0.00171 | 6.70039E-24 | 0.0077 | 0.0094 | 0.4133 | 0.000132771 | 101.7618151 |
| rs10191758 | 2 | 144263280 | G | A | 0.381 | 0.01631 | 0.00175 | 9.60064E-21 | -0.0142 | 0.0095 | 0.1372 | 0.000113333 | 86.86217331 |
| rs10205801 | 2 | 233597196 | A | G | 0.5068 | -0.01053 | 0.00171 | 7.17001E-10 | -0.0186 | 0.0093 | 0.0458395 | 4.94787E-05 | 37.91956863 |
| rs10215082 | 7 | 92657985 | G | A | 0.5612 | 0.01303 | 0.00172 | 3.32966E-14 | -0.0063 | 0.0093 | 0.499401 | 7.48816E-05 | 57.3892837 |
| rs10240905 | 7 | 32263069 | C | T | 0.6684 | 0.01167 | 0.00177 | 3.78966E-11 | -0.0205 | 0.0097 | 0.0340298 | 5.67213E-05 | 43.47044099 |
| rs10456918 | 6 | 119233480 | C | A | 0.182 | 0.01485 | 0.00224 | 3.67029E-11 | -0.0135 | 0.012 | 0.2626 | 5.73466E-05 | 43.94968202 |
| rs10460095 | 18 | 22624708 | A | G | 0.5867 | -0.01066 | 0.00171 | 4.87001E-10 | -0.0132 | 0.0094 | 0.1589 | 5.07079E-05 | 38.86163381 |
| rs1051474 | 9 | 111881856 | C | T | 0.2738 | 0.01301 | 0.00188 | 4.85959E-12 | 0.0007 | 0.0103 | 0.9438 | 6.24867E-05 | 47.88921974 |
| rs10760023 | 9 | 121958917 | G | C | 0.3316 | 0.01095 | 0.00183 | 2.12002E-09 | -0.0094 | 0.01 | 0.3507 | 4.67177E-05 | 35.80345399 |
| rs10765775 | 11 | 95656362 | A | G | 0.3963 | 0.01488 | 0.00176 | 2.61999E-17 | -0.0225 | 0.0095 | 0.01869 | 9.32644E-05 | 71.4791523 |
| rs10772644 | 12 | 13417617 | C | G | 0.8929 | 0.01614 | 0.00267 | 1.5E-09 | -0.0259 | 0.015 | 0.0841996 | 4.76804E-05 | 36.54125042 |
| rs10773002 | 12 | 123746961 | T | A | 0.7211 | -0.02191 | 0.00197 | 8.67961E-29 | 0.0503 | 0.0108 | 3.01301E-06 | 0.000161383 | 123.6947221 |
| rs10797055 | 1 | 159906849 | G | A | 0.5102 | 0.00986 | 0.00172 | 1.03999E-08 | -0.0002 | 0.0093 | 0.9823 | 4.28799E-05 | 32.86213706 |
| rs10798418 | 1 | 175839834 | T | C | 0.466 | -0.00957 | 0.00173 | 3.29003E-08 | -0.0025 | 0.0095 | 0.791099 | 3.99291E-05 | 30.60064185 |
| rs10856785 | 2 | 16650175 | T | C | 0.7296 | -0.01132 | 0.00192 | 3.83001E-09 | 0.0271 | 0.0104 | 0.00918692 | 4.53572E-05 | 34.76075998 |
| rs10862376 | 12 | 82257633 | A | T | 0.1361 | 0.01616 | 0.00239 | 1.39991E-11 | -0.0259 | 0.0132 | 0.0501199 | 5.96536E-05 | 45.71784781 |
| rs10875121 | 1 | 98417446 | C | G | 0.8571 | 0.01834 | 0.00226 | 5.52968E-16 | -0.0404 | 0.0125 | 0.00126 | 8.59251E-05 | 65.85377128 |
| rs10887801 | 10 | 90079714 | T | G | 0.4371 | 0.01087 | 0.00171 | 2.27002E-10 | 0.0009 | 0.0094 | 0.9272 | 5.27254E-05 | 40.40784913 |
| rs10940921 | 5 | 30808645 | G | T | 0.5697 | -0.01089 | 0.00177 | 7.00003E-10 | 0.0174 | 0.0094 | 0.0655194 | 4.93928E-05 | 37.85367886 |
| rs10963297 | 9 | 1791832 | G | C | 0.2517 | 0.01904 | 0.00198 | 7.36038E-22 | -0.0035 | 0.0109 | 0.744 | 0.00012065 | 92.4703229 |
| rs10994777 | 10 | 63233988 | A | G | 0.1395 | 0.0146 | 0.00232 | 3.36001E-10 | 0.0069 | 0.0126 | 0.5869 | 5.16753E-05 | 39.60304765 |
| rs11023749 | 11 | 15889272 | A | G | 0.6701 | 0.01132 | 0.0018 | 2.95999E-10 | -0.0211 | 0.0098 | 0.0319801 | 5.16061E-05 | 39.55002024 |
| rs1105307 | 9 | 72046040 | A | G | 0.2449 | -0.01173 | 0.00195 | 1.67001E-09 | 0.015 | 0.0106 | 0.1567 | 4.72152E-05 | 36.18475764 |
| rs1106090 | 2 | 58068741 | A | G | 0.6259 | 0.01173 | 0.00175 | 2.08978E-11 | 0.0007 | 0.0097 | 0.9427 | 5.86233E-05 | 44.92817662 |
| rs11081529 | 18 | 75902735 | C | T | 0.2568 | -0.01311 | 0.00186 | 1.82012E-12 | 0.0088 | 0.0102 | 0.3869 | 6.48227E-05 | 49.67963101 |
| rs11123818 | 2 | 100867308 | A | G | 0.3946 | 0.02081 | 0.00175 | 1.71989E-32 | -0.0187 | 0.0096 | 0.0505603 | 0.000184486 | 141.4057044 |
| rs111821073 | 9 | 99084793 | T | C | 0.1633 | 0.01385 | 0.00237 | 4.84998E-09 | -0.0156 | 0.0129 | 0.2242 | 4.45614E-05 | 34.15086603 |
| rs112687095 | 4 | 1777045 | A | G | 0.165 | 0.01325 | 0.00238 | 2.42003E-08 | -0.003 | 0.0125 | 0.811 | 4.04423E-05 | 30.99393437 |
| rs112806496 | 2 | 80421805 | G | C | 0.08503 | 0.0187 | 0.00305 | 8.27999E-10 | -0.0188 | 0.0162 | 0.2464 | 4.90499E-05 | 37.59087206 |
| rs113182709 | 8 | 143297485 | A | G | 0.02381 | 0.03225 | 0.00567 | 1.29E-08 | -0.0209 | 0.0295 | 0.4775 | 4.22134E-05 | 32.3513335 |
| rs113520408 | 7 | 128402782 | A | G | 0.2857 | 0.01304 | 0.00192 | 1.02E-11 | 0.0103 | 0.0103 | 0.3211 | 6.01869E-05 | 46.12661573 |
| rs113615161 | 7 | 114519339 | T | C | 0.1395 | -0.01472 | 0.0025 | 3.97E-09 | 0.024 | 0.0136 | 0.0783105 | 4.52368E-05 | 34.66845352 |
| rs1143770 | 11 | 122017598 | T | C | 0.5918 | 0.01136 | 0.00172 | 4.31023E-11 | 0.0045 | 0.0094 | 0.634699 | 5.69181E-05 | 43.62130314 |
| rs115000530 | 3 | 69930625 | T | A | 0.06122 | 0.02892 | 0.00381 | 3.29989E-14 | 0.0267 | 0.0204 | 0.1914 | 7.51778E-05 | 57.61631687 |
| rs115454970 | 4 | 67091953 | T | G | 0.3044 | -0.01185 | 0.00199 | 2.76001E-09 | 0.013 | 0.0108 | 0.2288 | 4.62686E-05 | 35.45923929 |
| rs11601122 | 11 | 72362718 | G | A | 0.1497 | -0.01947 | 0.0023 | 2.23975E-17 | 0.0069 | 0.0127 | 0.5862 | 9.34999E-05 | 71.65971846 |
| rs11620355 | 13 | 92044587 | A | G | 0.1156 | 0.01756 | 0.003 | 4.77002E-09 | 0.0199 | 0.0166 | 0.2329 | 4.47057E-05 | 34.2614217 |
| rs11627087 | 14 | 57300708 | G | A | 0.08503 | -0.01788 | 0.00325 | 3.70997E-08 | -0.0011 | 0.0181 | 0.9533 | 3.94936E-05 | 30.26684645 |
| rs11635092 | 15 | 27234553 | A | G | 0.3639 | -0.01231 | 0.00177 | 3.88956E-12 | 0.0147 | 0.0097 | 0.1303 | 6.31129E-05 | 48.36914824 |
| rs11657342 | 17 | 79355294 | A | G | 0.3554 | 0.01404 | 0.00191 | 1.93999E-13 | 0.0036 | 0.0096 | 0.7069 | 7.05038E-05 | 54.0339041 |
| rs11663602 | 18 | 77578191 | A | C | 0.2568 | -0.01213 | 0.0019 | 1.63999E-10 | 0.0066 | 0.0104 | 0.5232 | 5.31823E-05 | 40.75803767 |
| rs11678980 | 2 | 162101261 | A | G | 0.4456 | -0.01744 | 0.00172 | 4.29042E-24 | 0.0185 | 0.0096 | 0.0539896 | 0.000134139 | 102.8098993 |
| rs11681861 | 2 | 29633087 | G | T | 0.1565 | -0.01435 | 0.00259 | 2.87998E-08 | -0.0031 | 0.0138 | 0.8249 | 4.00555E-05 | 30.69750937 |
| rs11694904 | 2 | 180925445 | T | C | 0.3384 | 0.01215 | 0.00185 | 4.77969E-11 | -0.0085 | 0.0101 | 0.3961 | 5.62808E-05 | 43.13283119 |
| rs11732657 | 4 | 172427073 | A | G | 0.7007 | -0.01274 | 0.00197 | 9.53894E-11 | 0.0061 | 0.0107 | 0.5692 | 5.45706E-05 | 41.82204551 |
| rs117468730 | 16 | 10205467 | A | G | 0.0119 | -0.03521 | 0.00597 | 3.79001E-09 | 0.0103 | 0.033 | 0.754401 | 4.53878E-05 | 34.78421882 |
| rs11752914 | 6 | 155910988 | C | T | 0.1973 | -0.01208 | 0.00216 | 2.13E-08 | 0.0123 | 0.0117 | 0.2912 | 4.08117E-05 | 31.27701028 |
| rs11772580 | 7 | 100101648 | T | G | 0.2534 | -0.01199 | 0.00201 | 2.56998E-09 | 0.0114 | 0.011 | 0.3007 | 4.64303E-05 | 35.58320953 |
| rs117799466 | 15 | 34659517 | C | G | 0.3776 | 0.01173 | 0.00198 | 2.90998E-09 | -0.0259 | 0.0101 | 0.0104501 | 4.57954E-05 | 35.09655671 |
| rs11871429 | 17 | 42920929 | G | A | 0.2041 | -0.01425 | 0.00202 | 1.91999E-12 | 0.0011 | 0.0111 | 0.9181 | 6.49343E-05 | 49.76521176 |
| rs12028010 | 1 | 41764471 | C | T | 0.2228 | -0.01696 | 0.00202 | 4.51024E-17 | 0.0272 | 0.011 | 0.0136499 | 9.19782E-05 | 70.49329706 |
| rs12134151 | 1 | 96202443 | C | G | 0.5221 | -0.01245 | 0.0017 | 2.41991E-13 | -0.0095 | 0.0093 | 0.308 | 6.9982E-05 | 53.63394307 |
| rs12332731 | 5 | 93033082 | A | T | 0.2024 | 0.01374 | 0.00218 | 3.11997E-10 | -0.0027 | 0.0117 | 0.8143 | 5.18339E-05 | 39.7245828 |
| rs12375949 | 9 | 124617900 | C | T | 0.5697 | 0.01447 | 0.00172 | 3.30979E-17 | -0.0156 | 0.0093 | 0.0936807 | 9.23455E-05 | 70.77486261 |
| rs12468040 | 2 | 44854981 | G | T | 0.6037 | -0.01432 | 0.00175 | 2.4598E-16 | 0.0038 | 0.0096 | 0.692599 | 8.7367E-05 | 66.95897627 |
| rs12503522 | 4 | 94543233 | T | C | 0.2483 | -0.01125 | 0.00188 | 2.24001E-09 | -0.0125 | 0.0103 | 0.2247 | 4.67245E-05 | 35.80867183 |
| rs12519073 | 5 | 136776762 | T | C | 0.2381 | -0.01221 | 0.00202 | 1.6E-09 | 0.0056 | 0.0111 | 0.617101 | 4.76742E-05 | 36.53654321 |
| rs12574281 | 11 | 131205421 | C | A | 0.3997 | 0.01077 | 0.00176 | 8.84993E-10 | -0.0171 | 0.0096 | 0.0737208 | 4.88608E-05 | 37.44595728 |
| rs12602286 | 17 | 19236954 | T | G | 0.8861 | 0.01701 | 0.00255 | 2.36974E-11 | -0.0136 | 0.0139 | 0.3285 | 5.80602E-05 | 44.49663128 |
| rs12643771 | 4 | 140753103 | T | C | 0.3112 | 0.01518 | 0.00184 | 1.6099E-16 | -0.0176 | 0.01 | 0.0804007 | 8.88066E-05 | 68.06232237 |
| rs12682775 | 9 | 135490491 | C | T | 0.2143 | 0.01187 | 0.00204 | 5.99004E-09 | -0.0048 | 0.0112 | 0.670199 | 4.41771E-05 | 33.85633706 |
| rs12804787 | 11 | 133811072 | G | A | 0.06803 | -0.01814 | 0.00327 | 2.95999E-08 | -0.0004 | 0.0171 | 0.9819 | 4.01548E-05 | 30.77357323 |
| rs1291818 | 10 | 11132190 | C | T | 0.5153 | -0.01085 | 0.0017 | 1.78E-10 | -0.0006 | 0.0094 | 0.9518 | 5.31513E-05 | 40.73432276 |
| rs12940014 | 17 | 17603317 | C | T | 0.5204 | 0.00936 | 0.0017 | 3.73001E-08 | 0.0042 | 0.0093 | 0.654 | 3.9556E-05 | 30.31466137 |
| rs12955211 | 18 | 25572105 | A | T | 0.3554 | 0.01097 | 0.00182 | 1.59001E-09 | 0.0068 | 0.0099 | 0.4911 | 4.74052E-05 | 36.33033025 |
| rs13010566 | 2 | 125873208 | C | A | 0.5612 | 0.0106 | 0.0017 | 4.58997E-10 | 0.002 | 0.0093 | 0.8265 | 5.07303E-05 | 38.87879127 |
| rs13029509 | 2 | 215374209 | A | G | 0.4677 | -0.01049 | 0.0017 | 7.17001E-10 | 0.0139 | 0.0093 | 0.1344 | 4.96829E-05 | 38.0760598 |
| rs13090388 | 3 | 49391082 | T | C | 0.3095 | 0.02852 | 0.00184 | 4.29042E-54 | -0.0221 | 0.0101 | 0.0284001 | 0.000313403 | 240.249373 |
| rs13130765 | 4 | 45163333 | C | G | 0.4558 | -0.01014 | 0.00173 | 4.68997E-09 | 0.0133 | 0.0093 | 0.1513 | 4.4827E-05 | 34.35441601 |
| rs13141210 | 4 | 67891641 | T | C | 0.5085 | 0.01361 | 0.00172 | 2.25996E-15 | -0.0102 | 0.0093 | 0.272 | 8.16958E-05 | 62.61209322 |
| rs13145650 | 4 | 122963344 | T | C | 0.90816 | -0.01918 | 0.00306 | 3.79997E-10 | 0.0232 | 0.0165 | 0.1609 | 5.12634E-05 | 39.28739373 |
| rs1334297 | 13 | 58335375 | A | G | 0.784 | 0.02449 | 0.00192 | 3.05985E-37 | -0.0193 | 0.0105 | 0.06601 | 0.000212255 | 162.694915 |
| rs13422673 | 2 | 155474355 | T | C | 0.4847 | -0.01201 | 0.0017 | 1.7398E-12 | -0.0019 | 0.0093 | 0.8404 | 6.51232E-05 | 49.90993895 |
| rs1363862 | 5 | 59670035 | A | G | 0.2602 | -0.01171 | 0.00192 | 1.02E-09 | 0.0013 | 0.0104 | 0.8989 | 4.85362E-05 | 37.19719567 |
| rs1381247 | 17 | 2302387 | C | T | 0.2908 | -0.01013 | 0.00182 | 2.45997E-08 | -0.0182 | 0.0098 | 0.0639602 | 4.04235E-05 | 30.97954118 |
| rs1391438 | 4 | 106151843 | C | T | 0.6854 | -0.0167 | 0.00183 | 5.79029E-20 | 0.0023 | 0.01 | 0.8165 | 0.000108657 | 83.27787398 |
| rs1427298 | 2 | 145214421 | T | C | 0.4116 | 0.0102 | 0.00172 | 3.27997E-09 | -0.0117 | 0.0095 | 0.2171 | 4.5888E-05 | 35.16756641 |
| rs1450782 | 4 | 112514407 | G | T | 0.602 | -0.00945 | 0.00173 | 4.93003E-08 | 0.0036 | 0.0094 | 0.699 | 3.89341E-05 | 29.83803901 |
| rs1455350 | 2 | 199497115 | A | T | 0.4711 | -0.01614 | 0.0017 | 2.60976E-21 | -0.0064 | 0.0093 | 0.4936 | 0.000117607 | 90.13803465 |
| rs152603 | 5 | 106774922 | G | A | 0.3861 | 0.01019 | 0.00177 | 9.47E-09 | -0.003 | 0.0096 | 0.756901 | 4.32473E-05 | 33.14367806 |
| rs1558727 | 12 | 97680631 | T | C | 0.4847 | -0.01069 | 0.0017 | 3.09001E-10 | 0.0192 | 0.0093 | 0.0389 | 5.15954E-05 | 39.54179992 |
| rs1566085 | 8 | 142624527 | T | G | 0.5697 | 0.01645 | 0.00171 | 6.90081E-22 | -0.0263 | 0.0094 | 0.00508698 | 0.000120743 | 92.54190821 |
| rs1569092 | 1 | 74858724 | A | G | 0.182 | 0.01807 | 0.00234 | 1.16011E-14 | -0.0137 | 0.0128 | 0.2818 | 7.78084E-05 | 59.63256042 |
| rs1584469 | 5 | 120101694 | T | C | 0.3112 | -0.01303 | 0.00185 | 2.09991E-12 | 0.0169 | 0.0102 | 0.0974294 | 6.47281E-05 | 49.60714592 |
| rs1592757 | 5 | 103889998 | C | G | 0.3759 | -0.01045 | 0.0018 | 5.88993E-09 | 0.008 | 0.0097 | 0.4117 | 4.39789E-05 | 33.70438735 |
| rs1595973 | 4 | 65798787 | T | C | 0.5595 | -0.01002 | 0.00173 | 6.55994E-09 | 0.0104 | 0.0094 | 0.269 | 4.37724E-05 | 33.54610511 |
| rs1618725 | 18 | 21126952 | T | C | 0.5204 | 0.01477 | 0.00174 | 2.22024E-17 | -0.0214 | 0.0093 | 0.02103 | 9.4015E-05 | 72.05454177 |
| rs1620977 | 1 | 72729142 | G | A | 0.6905 | -0.02046 | 0.00195 | 1.13999E-25 | -0.0253 | 0.0105 | 0.0156899 | 0.000143633 | 110.0882334 |
| rs1671770 | 12 | 120946568 | C | A | 0.8061 | -0.01342 | 0.00223 | 1.90999E-09 | 0.0061 | 0.0124 | 0.6242 | 4.72553E-05 | 36.21547387 |
| rs16846463 | 2 | 142316255 | G | A | 0.1173 | -0.02256 | 0.00283 | 1.38007E-15 | 0.026 | 0.0154 | 0.092221 | 8.29173E-05 | 63.54833644 |
| rs16854920 | 1 | 204966170 | C | T | 0.3554 | 0.01007 | 0.00181 | 2.50998E-08 | -0.0137 | 0.0098 | 0.1587 | 4.03887E-05 | 30.95285106 |
| rs1689510 | 12 | 56396768 | C | G | 0.3435 | 0.01761 | 0.0018 | 1.39991E-22 | -0.0199 | 0.0098 | 0.0420901 | 0.000124881 | 95.71336132 |
| rs16995054 | 20 | 14811733 | T | C | 0.2007 | -0.0139 | 0.00208 | 2.52E-11 | 0.004 | 0.0115 | 0.727699 | 5.82711E-05 | 44.65825993 |
| rs17048855 | 3 | 8258174 | A | G | 0.3248 | 0.01184 | 0.00179 | 3.26964E-11 | 0.003 | 0.0097 | 0.7614 | 5.70884E-05 | 43.75182864 |
| rs17110109 | 12 | 54668908 | C | T | 0.3776 | 0.01023 | 0.00175 | 4.70999E-09 | 0.008 | 0.0095 | 0.4029 | 4.45894E-05 | 34.17228633 |
| rs17126938 | 10 | 111761351 | C | T | 0.1207 | 0.01536 | 0.0025 | 8.13992E-10 | -0.0069 | 0.0136 | 0.6123 | 4.92557E-05 | 37.74863748 |
| rs17425572 | 9 | 88006338 | G | A | 0.5425 | -0.01224 | 0.0017 | 6.8897E-13 | 0.0124 | 0.0093 | 0.1825 | 6.76412E-05 | 51.83986471 |
| rs17489649 | 5 | 109156184 | G | A | 0.3265 | -0.0139 | 0.00181 | 1.57E-14 | -0.0023 | 0.0098 | 0.8161 | 7.69511E-05 | 58.97545733 |
| rs175325 | 20 | 22317310 | A | T | 0.5816 | -0.01179 | 0.00174 | 1.10994E-11 | 0.0051 | 0.0095 | 0.5918 | 5.99072E-05 | 45.91218696 |
| rs17551064 | 7 | 49869771 | G | A | 0.1599 | -0.01493 | 0.0023 | 8.61986E-11 | -0.01 | 0.0126 | 0.4265 | 5.49814E-05 | 42.13692217 |
| rs17563464 | 5 | 26913774 | A | C | 0.2041 | -0.01477 | 0.00212 | 2.89001E-12 | 0.0026 | 0.0113 | 0.8174 | 6.33341E-05 | 48.53869942 |
| rs17565975 | 11 | 111586950 | A | G | 0.5306 | -0.01142 | 0.00171 | 2.55976E-11 | 0.0052 | 0.0094 | 0.5823 | 5.81956E-05 | 44.60041026 |
| rs17598675 | 4 | 176647637 | C | T | 0.5187 | 0.01199 | 0.0017 | 1.74985E-12 | -0.014 | 0.0093 | 0.1342 | 6.49065E-05 | 49.74384942 |
| rs176218 | 14 | 29600506 | T | G | 0.2007 | 0.01883 | 0.00215 | 1.85012E-18 | 0.0076 | 0.0118 | 0.5196 | 0.000100082 | 76.70480793 |
| rs1827540 | 5 | 63001049 | G | A | 0.466 | -0.0106 | 0.0017 | 4.49997E-10 | 0.0211 | 0.0093 | 0.0233502 | 5.07303E-05 | 38.87879127 |
| rs1866823 | 8 | 57436577 | A | G | 0.551 | 0.01009 | 0.00171 | 3.80996E-09 | -0.0035 | 0.0094 | 0.708101 | 4.54303E-05 | 34.81681006 |
| rs1882273 | 2 | 194028079 | C | G | 0.3469 | -0.01231 | 0.00181 | 1.08993E-11 | -0.004 | 0.0098 | 0.6791 | 6.03543E-05 | 46.25490813 |
| rs192436652 | 19 | 54960747 | T | C | 0.02211 | -0.03497 | 0.00545 | 1.34999E-10 | 0.074 | 0.0286 | 0.00968501 | 5.37218E-05 | 41.1715414 |
| rs1925576 | 10 | 68689083 | G | A | 0.4422 | 0.00997 | 0.00171 | 4.94003E-09 | -0.0032 | 0.0094 | 0.732 | 4.43562E-05 | 33.99358455 |
| rs1947114 | 2 | 166180772 | G | A | 0.2551 | 0.01071 | 0.00192 | 2.63998E-08 | -0.0161 | 0.0106 | 0.1283 | 4.06008E-05 | 31.11539731 |
| rs1949226 | 4 | 21919895 | A | T | 0.5901 | -0.00965 | 0.00174 | 2.84001E-08 | -0.0032 | 0.0095 | 0.7329 | 4.01342E-05 | 30.75778074 |
| rs1964927 | 21 | 42653237 | G | A | 0.6378 | -0.01423 | 0.00177 | 9.8992E-16 | 0.0057 | 0.0097 | 0.554201 | 8.43339E-05 | 64.63416373 |
| rs2052285 | 16 | 51188432 | A | G | 0.5765 | 0.01123 | 0.00175 | 1.33999E-10 | -0.0055 | 0.0095 | 0.5604 | 5.37323E-05 | 41.17961498 |
| rs2067854 | 14 | 72425819 | A | G | 0.182 | 0.01477 | 0.00209 | 1.38007E-12 | -0.0201 | 0.0114 | 0.0776694 | 6.51652E-05 | 49.94215578 |
| rs2179152 | 6 | 26325888 | C | T | 0.6429 | 0.01455 | 0.00176 | 1.21004E-16 | -0.0236 | 0.0096 | 0.0137499 | 8.91739E-05 | 68.34386218 |
| rs2182505 | 6 | 51653864 | C | T | 0.7364 | -0.01086 | 0.00192 | 1.63999E-08 | 0.0045 | 0.0105 | 0.668599 | 4.1746E-05 | 31.99308057 |
| rs225291 | 17 | 33927126 | G | A | 0.8027 | -0.01205 | 0.00214 | 1.84001E-08 | -0.0171 | 0.0117 | 0.1453 | 4.13718E-05 | 31.70628899 |
| rs2256965 | 6 | 31555130 | G | A | 0.5425 | -0.01128 | 0.00176 | 1.59001E-10 | 0.0014 | 0.0094 | 0.8806 | 5.35976E-05 | 41.07633908 |
| rs2283076 | 7 | 126478190 | G | A | 0.2143 | -0.01143 | 0.00204 | 2.07E-08 | -0.0004 | 0.0111 | 0.9724 | 4.09628E-05 | 31.3928679 |
| rs2287838 | 19 | 9959014 | A | G | 0.534 | -0.01152 | 0.00171 | 1.53003E-11 | -0.0099 | 0.0093 | 0.2866 | 5.92192E-05 | 45.38492311 |
| rs2302761 | 17 | 7358520 | T | C | 0.1905 | 0.01354 | 0.00209 | 1E-10 | 0.0031 | 0.0113 | 0.786401 | 5.47642E-05 | 41.97044975 |
| rs2347526 | 4 | 159853577 | C | T | 0.6378 | 0.01395 | 0.00179 | 6.84069E-15 | 0.0014 | 0.0098 | 0.8869 | 7.92471E-05 | 60.73530543 |
| rs2414072 | 15 | 50850562 | A | T | 0.4864 | -0.01005 | 0.00171 | 4.35001E-09 | 0.0132 | 0.0093 | 0.1571 | 4.50709E-05 | 34.54130721 |
| rs242093 | 14 | 69481343 | A | G | 0.5476 | -0.01031 | 0.00172 | 2.07E-09 | 0.0053 | 0.0094 | 0.577301 | 4.6883E-05 | 35.93017259 |
| rs2441111 | 5 | 57601225 | A | G | 0.5323 | 0.01087 | 0.0017 | 1.78E-10 | -0.011 | 0.0093 | 0.2398 | 5.33475E-05 | 40.88463378 |
| rs2447535 | 8 | 118946183 | G | A | 0.7245 | 0.01181 | 0.00185 | 1.69001E-10 | -0.0042 | 0.0102 | 0.6827 | 5.31752E-05 | 40.75258904 |
| rs2478208 | 13 | 81471113 | C | G | 0.5 | -0.0106 | 0.0017 | 4.82003E-10 | -0.0074 | 0.0093 | 0.4242 | 5.07303E-05 | 38.87879127 |
| rs2545798 | 5 | 176855627 | A | T | 0.4949 | -0.01346 | 0.00171 | 3.11028E-15 | -0.0125 | 0.0093 | 0.1791 | 8.08423E-05 | 61.95791087 |
| rs2554835 | 18 | 74141190 | A | G | 0.398 | 0.00974 | 0.00175 | 2.68999E-08 | -0.0098 | 0.0095 | 0.3037 | 4.04203E-05 | 30.97709467 |
| rs2570497 | 2 | 104441546 | T | C | 0.6735 | -0.01233 | 0.00177 | 3.0297E-12 | 0.0215 | 0.0097 | 0.02666 | 6.33181E-05 | 48.52644618 |
| rs2725370 | 8 | 30852826 | C | T | 0.7109 | 0.01536 | 0.00187 | 1.97015E-16 | -0.0251 | 0.0101 | 0.01293 | 8.80312E-05 | 67.46803863 |
| rs277828 | 13 | 109693885 | A | C | 0.2568 | -0.01091 | 0.00196 | 2.71E-08 | 0.0371 | 0.0106 | 0.000474002 | 4.04292E-05 | 30.98391018 |
| rs2787101 | 14 | 30105121 | T | C | 0.6173 | 0.00968 | 0.00174 | 0.000000025 | -0.0155 | 0.0095 | 0.1046 | 4.03841E-05 | 30.94931809 |
| rs2819336 | 1 | 44015809 | C | T | 0.6616 | -0.01828 | 0.00177 | 5.46009E-25 | 0.0011 | 0.0097 | 0.9118 | 0.000139162 | 106.6607705 |
| rs2820314 | 1 | 201872209 | C | A | 0.3163 | -0.011 | 0.0018 | 9.34007E-10 | 0.0206 | 0.0098 | 0.03555 | 4.87298E-05 | 37.34558155 |
| rs28373063 | 4 | 183724843 | C | G | 0.2075 | 0.01389 | 0.00229 | 1.22999E-09 | 0.0198 | 0.0121 | 0.1023 | 4.80052E-05 | 36.79022072 |
| rs28513670 | 15 | 65982677 | G | A | 0.1531 | 0.01477 | 0.00225 | 5.06058E-11 | -0.0253 | 0.0123 | 0.0401402 | 5.62273E-05 | 43.0918184 |
| rs2885198 | 3 | 143636421 | G | A | 0.5017 | -0.01025 | 0.0017 | 1.81001E-09 | 0.0031 | 0.0094 | 0.7424 | 4.74357E-05 | 36.35371135 |
| rs2901616 | 1 | 72122959 | A | G | 0.517 | 0.00941 | 0.00171 | 3.76999E-08 | -0.0015 | 0.0093 | 0.8718 | 3.95135E-05 | 30.28209326 |
| rs2905426 | 19 | 19478022 | T | G | 0.6446 | 0.01037 | 0.00181 | 9.25998E-09 | 0.0059 | 0.0097 | 0.542199 | 4.28309E-05 | 32.82458391 |
| rs2923431 | 8 | 42394425 | C | G | 0.6446 | 0.0114 | 0.00176 | 9.84011E-11 | 0.0231 | 0.0096 | 0.01637 | 5.4744E-05 | 41.95495249 |
| rs2971970 | 7 | 133643778 | G | T | 0.7721 | 0.01654 | 0.00207 | 1.24997E-15 | -0.0277 | 0.0112 | 0.01358 | 8.33048E-05 | 63.84533736 |
| rs2998315 | 14 | 85032707 | G | A | 0.5782 | 0.01269 | 0.00171 | 1.11995E-13 | -0.0027 | 0.0093 | 0.768999 | 7.18581E-05 | 55.07187843 |
| rs3013014 | 10 | 12396699 | A | G | 0.6105 | -0.01024 | 0.00172 | 2.91998E-09 | 0.0169 | 0.0094 | 0.0713099 | 4.62486E-05 | 35.4439313 |
| rs301800 | 1 | 8490603 | C | T | 0.8197 | -0.01516 | 0.00224 | 1.33015E-11 | 0.0151 | 0.0122 | 0.2166 | 5.97657E-05 | 45.80377077 |
| rs3026996 | 1 | 159167290 | C | A | 0.284 | -0.01537 | 0.00199 | 1.05003E-14 | 0.0037 | 0.0108 | 0.728799 | 7.78365E-05 | 59.65412072 |
| rs31940 | 5 | 11476812 | A | G | 0.1344 | 0.01548 | 0.00246 | 3.23996E-10 | -0.0108 | 0.0134 | 0.4209 | 5.16684E-05 | 39.59775508 |
| rs320693 | 7 | 137043868 | C | G | 0.4728 | 0.01204 | 0.0017 | 1.58016E-12 | -0.0031 | 0.0093 | 0.742099 | 6.54489E-05 | 50.15959228 |
| rs337637 | 4 | 38604470 | A | G | 0.3367 | 0.01123 | 0.00177 | 2.10999E-10 | -0.0219 | 0.0096 | 0.0222802 | 5.2525E-05 | 40.25425991 |
| rs34316 | 5 | 88015545 | C | A | 0.5799 | -0.02016 | 0.00177 | 3.34965E-30 | -0.0012 | 0.0094 | 0.9026 | 0.000169253 | 129.7279004 |
| rs34394051 | 1 | 6853091 | G | A | 0.1599 | 0.01392 | 0.0024 | 6.19998E-09 | -0.0084 | 0.0127 | 0.5123 | 4.38948E-05 | 33.63991221 |
| rs34485537 | 16 | 83608924 | T | C | 0.3895 | 0.01075 | 0.00173 | 5.66996E-10 | -0.0188 | 0.0095 | 0.0477804 | 5.03824E-05 | 38.61211481 |
| rs34853711 | 7 | 2214187 | C | G | 0.2466 | -0.01596 | 0.00206 | 9.88098E-15 | 0.0381 | 0.0111 | 0.000614695 | 7.83201E-05 | 60.02472788 |
| rs35039375 | 1 | 171516863 | G | A | 0.09354 | -0.01983 | 0.00293 | 1.22011E-11 | -0.0011 | 0.0161 | 0.9469 | 5.97668E-05 | 45.80459572 |
| rs35309068 | 12 | 14535908 | G | T | 0.466 | 0.01321 | 0.00171 | 1.15001E-14 | -0.0213 | 0.0094 | 0.0228302 | 7.78673E-05 | 59.67772805 |
| rs35316276 | 16 | 67850700 | T | C | 0.2942 | 0.01173 | 0.00194 | 1.51999E-09 | -0.002 | 0.0112 | 0.8584 | 4.77032E-05 | 36.55875781 |
| rs35417702 | 7 | 71739916 | T | C | 0.5765 | -0.01445 | 0.0017 | 1.93019E-17 | 0.0248 | 0.0093 | 0.00763396 | 9.42698E-05 | 72.24981144 |
| rs35475880 | 3 | 168853348 | T | G | 0.1837 | -0.01511 | 0.00208 | 3.80014E-13 | 0.0145 | 0.0115 | 0.2072 | 6.8857E-05 | 52.77170492 |
| rs35532491 | 22 | 34329603 | T | A | 0.1071 | 0.02007 | 0.00286 | 2.41991E-12 | -0.0009 | 0.0153 | 0.9512 | 6.42555E-05 | 49.24493236 |
| rs36083520 | 4 | 23715876 | C | T | 0.1667 | 0.01629 | 0.00223 | 2.60016E-13 | -0.0115 | 0.0122 | 0.3457 | 6.9627E-05 | 53.36190301 |
| rs36119825 | 7 | 48820536 | A | G | 0.4694 | 0.01063 | 0.00171 | 4.82003E-10 | 0.0045 | 0.0093 | 0.631801 | 5.04229E-05 | 38.6432082 |
| rs363096 | 4 | 3180021 | C | T | 0.5748 | 0.01363 | 0.00172 | 2.03986E-15 | -0.0006 | 0.0094 | 0.9518 | 8.1936E-05 | 62.79624634 |
| rs3747631 | 1 | 204587569 | C | G | 0.2279 | 0.02207 | 0.00208 | 2.9703E-26 | -0.0029 | 0.0114 | 0.7975 | 0.000146889 | 112.5840488 |
| rs3788556 | 22 | 39972162 | C | T | 0.5408 | -0.01138 | 0.00171 | 2.77971E-11 | 0.0037 | 0.0094 | 0.6953 | 5.77887E-05 | 44.28852024 |
| rs3800546 | 6 | 170067022 | G | C | 0.2704 | -0.01183 | 0.00194 | 9.72994E-10 | 0.0022 | 0.0107 | 0.8359 | 4.852E-05 | 37.18475257 |
| rs3809634 | 16 | 53538157 | G | A | 0.335 | 0.01058 | 0.00185 | 1.09001E-08 | -0.0117 | 0.0101 | 0.2439 | 4.26761E-05 | 32.70594825 |
| rs3890802 | 5 | 152081927 | A | G | 0.2687 | -0.01133 | 0.00191 | 2.74E-09 | -0.0032 | 0.0104 | 0.7565 | 4.59144E-05 | 35.18778679 |
| rs3897821 | 1 | 243420388 | G | A | 0.3503 | -0.01502 | 0.0018 | 8.25087E-17 | 0.0075 | 0.0098 | 0.4449 | 9.08513E-05 | 69.62957137 |
| rs401687 | 12 | 83924986 | C | G | 0.4558 | 0.01144 | 0.0017 | 1.85994E-11 | -0.0184 | 0.0093 | 0.0481005 | 5.90887E-05 | 45.28486451 |
| rs406413 | 5 | 113898581 | T | A | 0.2109 | -0.01695 | 0.00209 | 4.83949E-16 | 0.0133 | 0.0114 | 0.2426 | 8.58194E-05 | 65.77270443 |
| rs4073894 | 7 | 104466964 | A | G | 0.1769 | 0.01524 | 0.00211 | 5.40008E-13 | -0.0084 | 0.0116 | 0.4679 | 6.80693E-05 | 52.1679643 |
| rs4328757 | 3 | 36938180 | T | C | 0.6514 | 0.01067 | 0.00174 | 9.3901E-10 | -0.0126 | 0.0095 | 0.1857 | 4.90665E-05 | 37.60358134 |
| rs4352658 | 6 | 88279872 | T | C | 0.09014 | -0.0212 | 0.00308 | 5.55009E-12 | 0.0263 | 0.017 | 0.1215 | 6.18186E-05 | 47.37717438 |
| rs4369924 | 20 | 41954383 | A | G | 0.1684 | 0.01362 | 0.00234 | 5.81996E-09 | 0.0105 | 0.0127 | 0.409 | 4.42058E-05 | 33.87828108 |
| rs4382592 | 9 | 134870755 | G | T | 0.699 | 0.01636 | 0.00185 | 1.00995E-18 | 0.0071 | 0.0101 | 0.483 | 0.000102036 | 78.20274697 |
| rs4384309 | 10 | 133110596 | A | G | 0.4796 | 0.0109 | 0.00172 | 2.52E-10 | -0.0064 | 0.0094 | 0.4967 | 5.24021E-05 | 40.16011693 |
| rs4392737 | 6 | 145110726 | G | A | 0.3827 | -0.0097 | 0.00173 | 2.24001E-08 | 0.0078 | 0.0095 | 0.4134 | 4.10213E-05 | 31.43765393 |
| rs4442732 | 14 | 61025617 | G | A | 0.5969 | -0.01063 | 0.00176 | 1.49001E-09 | -0.0046 | 0.0096 | 0.6293 | 4.75988E-05 | 36.47875939 |
| rs4497562 | 13 | 62612604 | G | A | 0.2823 | -0.01204 | 0.00192 | 3.73001E-10 | 0.0192 | 0.0106 | 0.0710101 | 5.13102E-05 | 39.32324807 |
| rs4667025 | 2 | 186150287 | A | G | 0.3878 | 0.00957 | 0.00174 | 3.84999E-08 | 0.0073 | 0.0095 | 0.4416 | 3.94715E-05 | 30.24992105 |
| rs4700393 | 5 | 60098267 | G | A | 0.5289 | 0.02086 | 0.0017 | 1.51008E-34 | 0.0091 | 0.0093 | 0.3249 | 0.000196436 | 150.5669427 |
| rs4726070 | 7 | 151328218 | A | G | 0.6207 | 0.01251 | 0.00174 | 5.94977E-13 | 0.0076 | 0.0095 | 0.4247 | 6.7447E-05 | 51.6910066 |
| rs4733264 | 8 | 31490621 | C | G | 0.631 | -0.00954 | 0.00174 | 4.53002E-08 | 0.0163 | 0.0096 | 0.0893799 | 3.92245E-05 | 30.06056364 |
| rs4743923 | 9 | 96251061 | C | T | 0.3673 | 0.00985 | 0.00176 | 2.42003E-08 | -0.0092 | 0.0097 | 0.3427 | 4.087E-05 | 31.32174806 |
| rs4757957 | 11 | 12881398 | C | G | 0.6514 | 0.0141 | 0.00184 | 1.81009E-14 | 0.0215 | 0.01 | 0.0308297 | 7.66205E-05 | 58.7220821 |
| rs4766424 | 12 | 1954096 | G | C | 0.91156 | -0.0141 | 0.00258 | 4.42996E-08 | 0.0279 | 0.0139 | 0.0449004 | 3.89724E-05 | 29.867418 |
| rs4778058 | 15 | 93456069 | C | T | 0.5221 | 0.01017 | 0.0017 | 2.39999E-09 | -0.0013 | 0.0094 | 0.8935 | 4.66981E-05 | 35.78845331 |
| rs4787457 | 16 | 28555400 | G | A | 0.3146 | -0.01741 | 0.00176 | 3.72992E-23 | 0.0124 | 0.0096 | 0.1962 | 0.000127671 | 97.8523079 |
| rs4810227 | 20 | 59841800 | A | G | 0.6344 | 0.01272 | 0.00175 | 3.57026E-13 | 0.0051 | 0.0096 | 0.5946 | 6.89356E-05 | 52.83199273 |
| rs4839155 | 1 | 112161489 | G | T | 0.25 | -0.01251 | 0.002 | 3.94003E-10 | 0.0148 | 0.0109 | 0.1745 | 5.10515E-05 | 39.12492289 |
| rs4846724 | 1 | 221967817 | A | G | 0.4915 | 0.01018 | 0.0017 | 2.26001E-09 | -0.0258 | 0.0093 | 0.005724 | 4.679E-05 | 35.85886835 |
| rs4870482 | 6 | 157079675 | G | C | 0.2585 | -0.01083 | 0.0019 | 1.26999E-08 | -0.0169 | 0.0104 | 0.1041 | 4.23943E-05 | 32.48991521 |
| rs4888746 | 16 | 78172252 | G | A | 0.3776 | -0.00952 | 0.00174 | 4.15002E-08 | 0.0064 | 0.0096 | 0.504001 | 3.90602E-05 | 29.93465566 |
| rs4904523 | 14 | 89723630 | A | G | 0.4592 | -0.00936 | 0.0017 | 3.70997E-08 | -0.0023 | 0.0093 | 0.8075 | 3.9556E-05 | 30.31466137 |
| rs4945424 | 11 | 80305937 | A | C | 0.415 | -0.00992 | 0.00171 | 6.94001E-09 | 0.0091 | 0.0094 | 0.3361 | 4.39125E-05 | 33.65348079 |
| rs4964046 | 12 | 27305310 | G | A | 0.335 | 0.01053 | 0.00178 | 3.36001E-09 | -0.0054 | 0.0097 | 0.5758 | 4.56639E-05 | 34.99577409 |
| rs4972400 | 2 | 174171884 | A | G | 0.352 | 0.01156 | 0.00181 | 1.7E-10 | -0.0027 | 0.0097 | 0.7796 | 5.32244E-05 | 40.79034561 |
| rs4984541 | 15 | 96911139 | G | A | 0.2415 | 0.01233 | 0.00207 | 2.77E-09 | -0.0081 | 0.0111 | 0.4626 | 4.62957E-05 | 35.48005863 |
| rs510706 | 11 | 90191286 | C | G | 0.6156 | 0.01071 | 0.0018 | 2.72998E-09 | 0.0167 | 0.0375 | 0.656 | 4.61944E-05 | 35.40240761 |
| rs510706 | 11 | 90191286 | C | G | 0.6156 | 0.01071 | 0.0018 | 2.72998E-09 | -0.0079 | 0.0096 | 0.4077 | 4.61944E-05 | 35.40240761 |
| rs535307 | 7 | 105345398 | G | A | 0.6769 | -0.01004 | 0.00184 | 4.72999E-08 | 0.0209 | 0.0101 | 0.0384804 | 3.885E-05 | 29.77355179 |
| rs55736314 | 3 | 71586293 | G | C | 0.4167 | 0.01431 | 0.00174 | 1.63005E-16 | -0.0071 | 0.0095 | 0.4523 | 8.82507E-05 | 67.63626819 |
| rs55771711 | 10 | 133802737 | C | G | 0.2279 | 0.01555 | 0.00199 | 5.41003E-15 | -0.0085 | 0.0106 | 0.4242 | 7.96702E-05 | 61.05953611 |
| rs56391344 | 15 | 78006899 | A | G | 0.2381 | 0.01571 | 0.00197 | 1.33999E-15 | -0.0096 | 0.0107 | 0.3692 | 8.29773E-05 | 63.59438684 |
| rs575113 | 1 | 110046373 | A | G | 0.2772 | 0.01285 | 0.00186 | 5.30029E-12 | -0.0036 | 0.0103 | 0.723401 | 6.22772E-05 | 47.72865911 |
| rs59123361 | 1 | 110766454 | A | G | 0.1054 | -0.02094 | 0.00291 | 5.86949E-13 | 0.0118 | 0.0152 | 0.4363 | 6.75637E-05 | 51.78050043 |
| rs59480703 | 8 | 9653130 | C | G | 0.1633 | -0.01237 | 0.00215 | 8.31994E-09 | 0.0133 | 0.0117 | 0.2562 | 4.31936E-05 | 33.10254206 |
| rs60483752 | 16 | 87444253 | C | G | 0.5816 | 0.01078 | 0.00172 | 3.89E-10 | -0.0295 | 0.0095 | 0.00177799 | 5.12547E-05 | 39.28072496 |
| rs6122735 | 20 | 47523732 | T | C | 0.4133 | 0.0105 | 0.00174 | 1.49001E-09 | -0.0063 | 0.0095 | 0.5039 | 4.75155E-05 | 36.41488713 |
| rs6123924 | 20 | 58219764 | G | A | 0.1599 | -0.01528 | 0.00235 | 7.54918E-11 | 0.0249 | 0.0129 | 0.0530994 | 5.51649E-05 | 42.27755377 |
| rs613872 | 18 | 53210302 | T | G | 0.8282 | -0.0175 | 0.00227 | 1.20005E-14 | 0.0216 | 0.0123 | 0.0775693 | 7.75472E-05 | 59.43239744 |
| rs61747885 | 4 | 140640703 | T | G | 0.1412 | 0.01383 | 0.00235 | 4.13E-09 | 0.008 | 0.0128 | 0.5343 | 4.51923E-05 | 34.63438675 |
| rs62097985 | 18 | 50810675 | T | C | 0.4116 | -0.01288 | 0.00172 | 6.06038E-14 | 0.017 | 0.0094 | 0.0717596 | 7.31676E-05 | 56.07557026 |
| rs62157915 | 2 | 98897183 | C | T | 0.05952 | 0.02091 | 0.00348 | 1.96002E-09 | -0.0322 | 0.0182 | 0.0773197 | 4.71091E-05 | 36.10342837 |
| rs62183776 | 2 | 172858117 | T | C | 0.1905 | -0.01308 | 0.00217 | 1.64999E-09 | 0.0112 | 0.0117 | 0.338 | 4.74079E-05 | 36.33246692 |
| rs62184480 | 2 | 212654200 | T | C | 0.2449 | -0.01528 | 0.00191 | 1.27997E-15 | 0.0203 | 0.0103 | 0.0489598 | 8.35063E-05 | 63.99983297 |
| rs622169 | 1 | 244437407 | T | C | 0.4677 | 0.00999 | 0.00178 | 1.88999E-08 | 0.0074 | 0.0097 | 0.4416 | 4.11007E-05 | 31.49849752 |
| rs62439690 | 7 | 21417556 | A | G | 0.267 | -0.01087 | 0.00194 | 2.18002E-08 | 0.0085 | 0.0106 | 0.4236 | 4.09651E-05 | 31.39456681 |
| rs62444881 | 7 | 2052318 | T | C | 0.1905 | 0.01815 | 0.00217 | 5.79029E-17 | -0.0293 | 0.0121 | 0.0154501 | 9.12788E-05 | 69.95723848 |
| rs6493265 | 15 | 47513253 | T | C | 0.3895 | -0.01385 | 0.00174 | 1.69981E-15 | -0.0042 | 0.0095 | 0.655901 | 8.26686E-05 | 63.35777493 |
| rs6513959 | 20 | 43620856 | G | A | 0.2789 | -0.01177 | 0.00185 | 1.88001E-10 | 0.0109 | 0.0102 | 0.2868 | 5.28156E-05 | 40.47700174 |
| rs6557171 | 6 | 152234593 | C | T | 0.7245 | 0.01567 | 0.00181 | 4.14954E-18 | -0.01 | 0.0098 | 0.3092 | 9.77944E-05 | 74.95139317 |
| rs663234 | 1 | 57735595 | G | C | 0.6122 | -0.01005 | 0.00174 | 7.38992E-09 | 0.0042 | 0.0095 | 0.658901 | 4.35302E-05 | 33.36049558 |
| rs66568921 | 3 | 85672018 | G | T | 0.3639 | 0.01565 | 0.00182 | 7.49031E-18 | -0.0114 | 0.0098 | 0.2411 | 9.64761E-05 | 73.94090714 |
| rs6731373 | 2 | 68503044 | A | G | 0.3367 | -0.01256 | 0.00181 | 3.46976E-12 | 0.0132 | 0.0097 | 0.172 | 6.28305E-05 | 48.15273902 |
| rs6731967 | 2 | 237145606 | C | G | 0.2092 | -0.01186 | 0.00199 | 2.35999E-09 | 0.0015 | 0.011 | 0.8911 | 4.63467E-05 | 35.51911136 |
| rs67885444 | 8 | 28678973 | T | C | 0.1718 | 0.01406 | 0.00232 | 1.47999E-09 | -0.0229 | 0.0126 | 0.0684305 | 4.79236E-05 | 36.72768358 |
| rs67890737 | 2 | 228984644 | A | C | 0.3265 | -0.01141 | 0.00179 | 2.01002E-10 | 0.0293 | 0.0098 | 0.00276401 | 5.30173E-05 | 40.63161582 |
| rs6803651 | 3 | 64431730 | T | G | 0.415 | 0.01131 | 0.00172 | 4.36014E-11 | -0.0044 | 0.0094 | 0.637699 | 5.64182E-05 | 43.23815784 |
| rs6805241 | 3 | 116532683 | C | T | 0.1973 | -0.01413 | 0.00203 | 3.0903E-12 | 0.0116 | 0.011 | 0.2919 | 6.3218E-05 | 48.44970248 |
| rs6867851 | 5 | 92182752 | C | G | 0.4235 | -0.012 | 0.00173 | 3.97009E-12 | 0.0057 | 0.0095 | 0.5487 | 6.27796E-05 | 48.11374392 |
| rs6938002 | 6 | 37526024 | A | G | 0.3963 | -0.01008 | 0.00173 | 5.41003E-09 | 0.0001 | 0.0094 | 0.9951 | 4.42981E-05 | 33.94905771 |
| rs6959891 | 7 | 54714000 | G | A | 0.2959 | -0.01136 | 0.00189 | 1.74E-09 | 0.0089 | 0.0103 | 0.3874 | 4.71399E-05 | 36.12700182 |
| rs7012546 | 8 | 105067737 | T | C | 0.4201 | 0.01009 | 0.00172 | 4.93003E-09 | -0.0071 | 0.0094 | 0.4529 | 4.49036E-05 | 34.41314031 |
| rs7016302 | 8 | 4833041 | G | C | 0.1769 | 0.01243 | 0.00228 | 4.98001E-08 | -0.0149 | 0.0124 | 0.2303 | 3.87821E-05 | 29.72154832 |
| rs702606 | 5 | 53167117 | C | T | 0.1701 | -0.01427 | 0.0025 | 1.12001E-08 | 0.0324 | 0.0136 | 0.0169699 | 4.25133E-05 | 32.58117897 |
| rs7029718 | 9 | 23358495 | A | G | 0.4354 | 0.02439 | 0.00174 | 1.85012E-44 | -0.0052 | 0.0094 | 0.5777 | 0.000256324 | 196.4825431 |
| rs7031698 | 9 | 82441486 | C | T | 0.7755 | 0.01248 | 0.00206 | 1.26E-09 | 0.0152 | 0.0111 | 0.1708 | 4.78905E-05 | 36.70232669 |
| rs710629 | 12 | 67697856 | A | G | 0.6565 | 0.01053 | 0.00177 | 2.95999E-09 | 0.0024 | 0.0098 | 0.8038 | 4.61813E-05 | 35.39232361 |
| rs71646142 | 1 | 212369228 | T | C | 0.1735 | 0.01286 | 0.00217 | 3.11E-09 | -0.0201 | 0.0119 | 0.0896293 | 4.58267E-05 | 35.12055223 |
| rs7233920 | 18 | 37416318 | A | G | 0.216 | -0.01315 | 0.00202 | 7.13017E-11 | 0.0208 | 0.0111 | 0.0619598 | 5.52969E-05 | 42.3787003 |
| rs7257460 | 19 | 30746753 | C | T | 0.2704 | -0.01145 | 0.00189 | 1.25E-09 | 0.0036 | 0.0103 | 0.723801 | 4.78897E-05 | 36.70170428 |
| rs7278859 | 21 | 20026532 | T | A | 0.3078 | 0.01013 | 0.00185 | 4.15002E-08 | -0.0107 | 0.0101 | 0.2904 | 3.91232E-05 | 29.98294586 |
| rs72807818 | 2 | 51819019 | A | G | 0.1241 | 0.01915 | 0.00252 | 2.78997E-14 | -0.0454 | 0.0136 | 0.000824499 | 7.53493E-05 | 57.74778643 |
| rs72828517 | 6 | 19036035 | C | T | 0.1412 | 0.01836 | 0.00224 | 2.83009E-16 | -0.0098 | 0.0121 | 0.4195 | 8.76571E-05 | 67.181266 |
| rs72840994 | 10 | 87124801 | G | T | 0.182 | 0.01247 | 0.00216 | 7.76998E-09 | 0.0108 | 0.0117 | 0.3579 | 4.34893E-05 | 33.32915256 |
| rs730384 | 14 | 74889870 | A | G | 0.4558 | 0.01016 | 0.00171 | 3.01002E-09 | 0.0039 | 0.0094 | 0.6765 | 4.60629E-05 | 35.30157334 |
| rs7315713 | 12 | 75383884 | T | A | 0.6633 | -0.01022 | 0.00187 | 4.34E-08 | 0.026 | 0.0102 | 0.01086 | 3.89742E-05 | 29.86877732 |
| rs7321274 | 13 | 69146186 | G | A | 0.1956 | -0.01275 | 0.00211 | 1.59001E-09 | -0.0053 | 0.0115 | 0.6451 | 4.76442E-05 | 36.51357241 |
| rs73301698 | 12 | 15539771 | A | G | 0.2262 | -0.01291 | 0.00208 | 5.81005E-10 | -0.0005 | 0.0115 | 0.9681 | 5.02666E-05 | 38.5234063 |
| rs7332724 | 13 | 91629272 | T | C | 0.2619 | -0.01149 | 0.00189 | 1.26E-09 | -0.0009 | 0.0104 | 0.9311 | 4.82249E-05 | 36.95858331 |
| rs73344830 | 10 | 103816828 | G | A | 0.602 | -0.0172 | 0.00172 | 1.94984E-23 | -0.0017 | 0.0094 | 0.86 | 0.000130473 | 99.99973902 |
| rs736282 | 14 | 94287860 | C | T | 0.5153 | -0.01082 | 0.0017 | 2.07E-10 | 0.0101 | 0.0093 | 0.2757 | 5.28578E-05 | 40.50937525 |
| rs73874335 | 3 | 157933483 | T | C | 0.05952 | -0.0199 | 0.00361 | 3.40001E-08 | 0.0086 | 0.0203 | 0.6725 | 3.96506E-05 | 30.38719519 |
| rs743316 | 21 | 35252696 | C | T | 0.182 | -0.01185 | 0.00208 | 0.000000012 | 0.0334 | 0.0114 | 0.003425 | 4.23514E-05 | 32.457039 |
| rs74643044 | 5 | 102535528 | C | T | 0.02721 | 0.02323 | 0.00386 | 1.79999E-09 | 0.066 | 0.0206 | 0.00137199 | 4.72584E-05 | 36.21785093 |
| rs74701752 | 1 | 20875676 | T | G | 0.09524 | 0.01591 | 0.00285 | 2.38002E-08 | 0.0112 | 0.0156 | 0.4713 | 4.06639E-05 | 31.16373523 |
| rs7481514 | 11 | 131291963 | G | A | 0.665 | 0.01072 | 0.00178 | 1.55998E-09 | -0.0122 | 0.0097 | 0.2102 | 4.73265E-05 | 36.27007325 |
| rs74998289 | 17 | 43913558 | G | T | 0.2398 | -0.01821 | 0.00213 | 1.31009E-17 | -0.0066 | 0.0112 | 0.5574 | 9.53663E-05 | 73.09026749 |
| rs7594904 | 2 | 101184651 | C | T | 0.4184 | 0.00969 | 0.00173 | 2.04998E-08 | -0.0109 | 0.0094 | 0.2466 | 4.09367E-05 | 31.37286744 |
| rs7603132 | 2 | 4951548 | A | G | 0.1548 | 0.01317 | 0.00215 | 9.17001E-10 | -0.015 | 0.0117 | 0.199 | 4.89609E-05 | 37.5226495 |
| rs76076331 | 2 | 10977585 | T | C | 0.131 | 0.01873 | 0.00248 | 4.40048E-14 | -0.0199 | 0.0141 | 0.1583 | 7.44244E-05 | 57.03888925 |
| rs7650602 | 3 | 141147414 | C | T | 0.4286 | 0.00939 | 0.00171 | 4.10998E-08 | -0.0142 | 0.0094 | 0.13 | 3.93457E-05 | 30.15350702 |
| rs76608582 | 19 | 4474725 | A | C | 0.04082 | 0.02798 | 0.00445 | 3.11E-10 | -0.0121 | 0.0228 | 0.5962 | 5.15856E-05 | 39.53431924 |
| rs76878669 | 11 | 66092567 | G | C | 0.2534 | -0.01399 | 0.00205 | 8.66962E-12 | -0.0188 | 0.011 | 0.0875306 | 6.07683E-05 | 46.57218066 |
| rs77025239 | 3 | 180734185 | A | G | 0.1088 | -0.01422 | 0.00234 | 1.32999E-09 | 0.0104 | 0.0129 | 0.4216 | 4.81861E-05 | 36.92889771 |
| rs77128898 | 11 | 61313525 | T | C | 0.02211 | -0.02769 | 0.00482 | 9.47E-09 | -0.0092 | 0.0257 | 0.718899 | 4.30634E-05 | 33.00279347 |
| rs77702622 | 8 | 143367857 | A | G | 0.07653 | -0.02447 | 0.00351 | 2.9902E-12 | -0.0369 | 0.0187 | 0.0485199 | 6.34164E-05 | 48.60182444 |
| rs77719387 | 3 | 49917021 | A | T | 0.01701 | -0.04597 | 0.00726 | 2.45997E-10 | 0.0025 | 0.0373 | 0.9464 | 5.23153E-05 | 40.09356117 |
| rs77835879 | 12 | 26522623 | G | A | 0.09014 | -0.01601 | 0.00288 | 2.67997E-08 | 0.0153 | 0.0154 | 0.3221 | 4.03233E-05 | 30.90270918 |
| rs7796203 | 7 | 117505487 | A | G | 0.5255 | -0.01074 | 0.00171 | 3.59998E-10 | -0.0171 | 0.0093 | 0.0660602 | 5.14718E-05 | 39.44711158 |
| rs7803932 | 7 | 70203673 | A | G | 0.1565 | 0.0143 | 0.00226 | 2.44E-10 | 0.0063 | 0.0124 | 0.6105 | 5.22406E-05 | 40.03631183 |
| rs7808399 | 7 | 8075222 | G | A | 0.5476 | 0.0107 | 0.00171 | 3.77998E-10 | -0.0166 | 0.0094 | 0.0763308 | 5.10892E-05 | 39.15382552 |
| rs7833201 | 8 | 87561978 | C | G | 0.1344 | -0.01532 | 0.00262 | 5.04998E-09 | 0.0111 | 0.0141 | 0.4325 | 4.4614E-05 | 34.19115836 |
| rs7863447 | 9 | 14155418 | A | G | 0.8333 | 0.01678 | 0.00233 | 5.91017E-13 | -0.0018 | 0.0129 | 0.8874 | 6.76735E-05 | 51.86458862 |
| rs78721320 | 5 | 3304854 | A | G | 0.2041 | 0.01307 | 0.00219 | 2.27997E-09 | 0.0051 | 0.0118 | 0.667599 | 4.64749E-05 | 35.61736707 |
| rs790647 | 10 | 106776484 | A | C | 0.2347 | -0.01482 | 0.00202 | 2.1702E-13 | -0.0011 | 0.011 | 0.9224 | 7.02326E-05 | 53.82605304 |
| rs7920624 | 10 | 67963186 | T | A | 0.4932 | -0.01181 | 0.0017 | 3.97009E-12 | -0.0051 | 0.0093 | 0.584901 | 6.29724E-05 | 48.26150034 |
| rs7924036 | 10 | 65191645 | T | G | 0.5391 | 0.01501 | 0.0017 | 1.07004E-18 | 0.0076 | 0.0093 | 0.4114 | 0.000101717 | 77.95830866 |
| rs79265434 | 7 | 24621381 | G | A | 0.1173 | 0.02331 | 0.00262 | 6.07995E-19 | -0.0282 | 0.0144 | 0.0498506 | 0.000103279 | 79.1554516 |
| rs79269403 | 3 | 108036819 | A | G | 0.2228 | 0.01447 | 0.00204 | 1.17004E-12 | -0.0187 | 0.011 | 0.0904191 | 6.56484E-05 | 50.31246481 |
| rs7928622 | 11 | 11670871 | T | A | 0.3078 | 0.01011 | 0.00181 | 2.52E-08 | -0.0114 | 0.01 | 0.2539 | 4.07102E-05 | 31.19924094 |
| rs795230 | 11 | 30774525 | T | C | 0.4184 | 0.00952 | 0.00172 | 2.97002E-08 | 0.0219 | 0.0094 | 0.0193299 | 3.99738E-05 | 30.63485785 |
| rs79523955 | 1 | 28799065 | G | A | 0.09524 | -0.01802 | 0.00283 | 1.86999E-10 | -0.0125 | 0.0156 | 0.4237 | 5.29041E-05 | 40.54483794 |
| rs7977614 | 12 | 110115286 | G | A | 0.3078 | 0.01325 | 0.00198 | 2.08978E-11 | -0.0179 | 0.0102 | 0.0785507 | 5.84321E-05 | 44.7816656 |
| rs7993663 | 13 | 55761771 | C | T | 0.3571 | 0.0118 | 0.00178 | 3.25012E-11 | -0.0068 | 0.0097 | 0.4812 | 5.73423E-05 | 43.94635671 |
| rs8008382 | 14 | 104054425 | C | T | 0.6871 | 0.01208 | 0.00185 | 6.12068E-11 | -0.025 | 0.0102 | 0.0138 | 5.56342E-05 | 42.63725907 |
| rs80171383 | 11 | 46084677 | A | G | 0.1241 | 0.0145 | 0.00241 | 1.83E-09 | 0.0156 | 0.0132 | 0.2363 | 4.72343E-05 | 36.19935113 |
| rs8020034 | 14 | 26981100 | A | G | 0.2058 | 0.01782 | 0.00223 | 1.17004E-15 | 0.0054 | 0.0121 | 0.654699 | 8.33192E-05 | 63.85641603 |
| rs818415 | 16 | 65448079 | G | T | 0.182 | 0.01235 | 0.00219 | 1.72001E-08 | -0.0327 | 0.0119 | 0.00599294 | 4.14957E-05 | 31.80127644 |
| rs837080 | 8 | 130925116 | C | T | 0.4932 | 0.01092 | 0.0017 | 1.43001E-10 | 0.0071 | 0.0093 | 0.4438 | 5.38393E-05 | 41.26162242 |
| rs892612 | 5 | 136533365 | C | A | 0.8418 | 0.01464 | 0.00237 | 6.62995E-10 | -0.0066 | 0.0128 | 0.6071 | 4.97897E-05 | 38.15788792 |
| rs894067 | 11 | 76454408 | A | G | 0.3929 | 0.01041 | 0.00175 | 2.74E-09 | -0.0298 | 0.0096 | 0.00183 | 4.61722E-05 | 35.38540969 |
| rs9289300 | 3 | 127144988 | C | T | 0.1837 | 0.01512 | 0.00234 | 1.09999E-10 | -0.0094 | 0.0128 | 0.4594 | 5.44783E-05 | 41.75137033 |
| rs9320493 | 6 | 114742697 | G | A | 0.8639 | -0.01394 | 0.0024 | 6.12999E-09 | 0.0155 | 0.013 | 0.2325 | 4.4021E-05 | 33.73664807 |
| rs9342482 | 6 | 66223843 | T | G | 0.2908 | 0.01264 | 0.00197 | 1.36E-10 | -0.0174 | 0.0107 | 0.1043 | 5.37173E-05 | 41.1680752 |
| rs9349956 | 6 | 14718260 | C | A | 0.2398 | 0.01881 | 0.00225 | 6.28058E-17 | -0.0303 | 0.012 | 0.0116799 | 9.11903E-05 | 69.8894176 |
| rs9372625 | 6 | 98344031 | A | G | 0.4133 | 0.02383 | 0.00176 | 6.75927E-42 | -0.017 | 0.0096 | 0.0754606 | 0.000239163 | 183.3249671 |
| rs9384679 | 6 | 108864419 | T | C | 0.4082 | -0.00959 | 0.00176 | 4.88E-08 | -0.0215 | 0.0096 | 0.0250398 | 3.8741E-05 | 29.69003744 |
| rs9386319 | 6 | 96566419 | G | A | 0.4269 | 0.00991 | 0.00174 | 1.26999E-08 | 0.0306 | 0.0095 | 0.00130801 | 4.23259E-05 | 32.43752269 |
| rs9386787 | 6 | 109600293 | G | A | 0.5136 | 0.00958 | 0.0017 | 1.81999E-08 | -0.024 | 0.0093 | 0.0097609 | 4.14372E-05 | 31.75645691 |
| rs9436866 | 1 | 69427576 | C | A | 0.09524 | 0.01882 | 0.00289 | 7.45075E-11 | -0.0001 | 0.0159 | 0.9944 | 5.53344E-05 | 42.40747544 |
| rs9503598 | 6 | 3446263 | A | G | 0.4388 | 0.01079 | 0.00171 | 3.11997E-10 | -0.0065 | 0.0094 | 0.4895 | 5.19522E-05 | 39.81525808 |
| rs9529119 | 13 | 31783977 | G | C | 0.8027 | -0.01295 | 0.00204 | 2.13E-10 | 0.0197 | 0.0112 | 0.0775997 | 5.25814E-05 | 40.29749672 |
| rs9556958 | 13 | 99100046 | T | C | 0.5289 | -0.0108 | 0.0017 | 2.38002E-10 | 0.0121 | 0.0093 | 0.1947 | 5.26626E-05 | 40.35975626 |
| rs9616906 | 22 | 51104680 | A | G | 0.4235 | 0.01497 | 0.00172 | 2.92012E-18 | -0.0102 | 0.0093 | 0.2767 | 9.8837E-05 | 75.75051215 |
| rs9679654 | 2 | 61836773 | C | T | 0.4847 | 0.01042 | 0.00172 | 1.29E-09 | -0.0067 | 0.0094 | 0.4752 | 4.78887E-05 | 36.70095884 |
| rs969512 | 4 | 147872742 | T | A | 0.2959 | 0.01249 | 0.00179 | 3.22033E-12 | -0.0004 | 0.0098 | 0.9695 | 6.35283E-05 | 48.68752313 |
| rs9704097 | 11 | 24980643 | A | C | 0.4728 | -0.0103 | 0.00171 | 1.61002E-09 | -0.0096 | 0.0093 | 0.3011 | 4.7341E-05 | 36.28115424 |
| rs9882532 | 3 | 16865845 | C | T | 0.3639 | -0.01208 | 0.00177 | 8.16959E-12 | 0.0035 | 0.0097 | 0.7196 | 6.07766E-05 | 46.57857549 |
| rs9914918 | 17 | 46962021 | A | G | 0.2823 | 0.01155 | 0.00189 | 8.9E-10 | -0.0051 | 0.0102 | 0.6173 | 4.87298E-05 | 37.34558155 |
| rs9933256 | 16 | 1246748 | G | A | 0.4082 | -0.01134 | 0.00172 | 4.56983E-11 | 0.0056 | 0.0093 | 0.5454 | 5.67179E-05 | 43.46784221 |
| rs9936270 | 16 | 12215741 | T | C | 0.3078 | -0.0136 | 0.00198 | 6.42984E-12 | -0.0167 | 0.0108 | 0.122 | 6.15597E-05 | 47.17873617 |
| rs9938678 | 16 | 61503635 | T | A | 0.2432 | 0.01355 | 0.00205 | 4.12003E-11 | 0.0016 | 0.011 | 0.8826 | 5.70062E-05 | 43.68876165 |
| rs9964724 | 18 | 35159124 | T | C | 0.6599 | 0.01978 | 0.00183 | 2.66011E-27 | -0.0147 | 0.01 | 0.1416 | 0.000152426 | 116.828624 |
| rs9995567 | 4 | 163698499 | A | G | 0.3827 | 0.00998 | 0.00178 | 1.93001E-08 | -0.0106 | 0.0097 | 0.2716 | 4.10184E-05 | 31.43546903 |
| Genome-wide significant SNPs for Hot drink temperature | | | | | | | | | | | | | |
| SNP | Chr | Position | EA | OA | EAF | Hot drink temperature | | | Knee Osteoarthritis | | | R2 | F |
|  |  |  |  |  |  | beta | SE | pval | beta | SE | pval |  |  |
| rs1001880 | 1 | 37144802 | C | T | 0.313764 | 0.00738412 | 0.0012841 | 8.9E-09 | 0.0015 | 0.0101 | 0.8826 | 7.22143E-05 | 33.06723659 |
| rs1027583 | 9 | 8267866 | C | G | 0.272924 | -0.00766076 | 0.00134369 | 0.000000012 | -0.0073 | 0.0105 | 0.485801 | 7.09855E-05 | 32.50451171 |
| rs10744560 | 12 | 2387099 | T | C | 0.340057 | -0.00689025 | 0.00125393 | 3.89996E-08 | 0.0134 | 0.0098 | 0.1707 | 6.59401E-05 | 30.19405743 |
| rs10764990 | 10 | 129152608 | A | G | 0.607319 | 0.00948211 | 0.00121444 | 5.79963E-15 | 0.0102 | 0.0095 | 0.2811 | 0.000133124 | 60.96154358 |
| rs10822089 | 10 | 64695143 | G | A | 0.523143 | -0.00727765 | 0.0011875 | 8.9E-10 | 0.029 | 0.0093 | 0.00180302 | 8.20228E-05 | 37.55892879 |
| rs10829603 | 10 | 131327351 | G | T | 0.451828 | 0.00735716 | 0.00119538 | 7.49998E-10 | -0.0104 | 0.0093 | 0.2641 | 8.27232E-05 | 37.8797003 |
| rs10927006 | 1 | 243557659 | C | T | 0.143611 | -0.0137404 | 0.00169286 | 4.79954E-16 | -0.0198 | 0.0132 | 0.1339 | 0.000143863 | 65.8801786 |
| rs111251222 | 5 | 176735612 | G | A | 0.258804 | -0.00773272 | 0.00135975 | 1.29999E-08 | -0.003 | 0.0106 | 0.778501 | 7.0627E-05 | 32.34033673 |
| rs1132845 | 18 | 72912469 | T | C | 0.404377 | -0.00699287 | 0.00121299 | 8.19993E-09 | -0.0063 | 0.0095 | 0.5048 | 7.25805E-05 | 33.23491491 |
| rs1144428 | 2 | 201097903 | A | G | 0.790277 | 0.0122206 | 0.00146033 | 5.79963E-17 | 0.002 | 0.0114 | 0.8635 | 0.000152923 | 70.02951959 |
| rs11570094 | 11 | 47359706 | A | C | 0.295801 | 0.00754557 | 0.00130192 | 6.80002E-09 | -0.0276 | 0.0102 | 0.00686894 | 7.33565E-05 | 33.59027895 |
| rs11710570 | 3 | 158056654 | C | T | 0.448108 | -0.00767936 | 0.00119156 | 1.2E-10 | 0.0059 | 0.0093 | 0.5295 | 9.07055E-05 | 41.53520192 |
| rs12038134 | 1 | 1703865 | A | T | 0.508213 | 0.0065373 | 0.00118661 | 3.59998E-08 | 0.0242 | 0.0093 | 0.009166 | 6.62837E-05 | 30.35141381 |
| rs12132579 | 1 | 188321352 | T | C | 0.357498 | 0.00705328 | 0.00123845 | 0.000000012 | 0.0128 | 0.0097 | 0.1864 | 7.08353E-05 | 32.43570915 |
| rs1260326 | 2 | 27730940 | C | T | 0.604332 | -0.00918503 | 0.0012103 | 3.19963E-14 | 0.0245 | 0.0095 | 0.00981206 | 0.00012577 | 57.59346326 |
| rs12622811 | 2 | 52787949 | T | C | 0.298571 | -0.0102643 | 0.00130168 | 3.10027E-15 | 0.0087 | 0.0102 | 0.3952 | 0.000135783 | 62.17965566 |
| rs12664031 | 6 | 57623371 | A | C | 0.289569 | -0.0104514 | 0.00137573 | 2.99985E-14 | -0.002 | 0.0107 | 0.8513 | 0.000126033 | 57.71392707 |
| rs12695358 | 3 | 117773652 | T | G | 0.597342 | -0.00706156 | 0.00121026 | 5.39995E-09 | -0.0197 | 0.0095 | 0.0369598 | 7.43475E-05 | 34.04411513 |
| rs1326609 | 6 | 51610248 | A | G | 0.371579 | -0.00780654 | 0.00122834 | 2.1E-10 | 0.007 | 0.0096 | 0.4692 | 8.82057E-05 | 40.39039427 |
| rs1447182 | 11 | 57916512 | C | T | 0.782248 | 0.00939207 | 0.00145062 | 9.49948E-11 | -0.0107 | 0.0113 | 0.3417 | 9.15441E-05 | 41.91924217 |
| rs1513475 | 3 | 35724256 | C | T | 0.34937 | -0.00891422 | 0.00124407 | 7.8001E-13 | 0.0002 | 0.0097 | 0.9797 | 0.00011212 | 51.34228159 |
| rs1568452 | 2 | 58012833 | T | C | 0.382005 | -0.0120516 | 0.00122159 | 5.90065E-23 | -0.0004 | 0.0096 | 0.9628 | 0.000212521 | 97.32772738 |
| rs1680349 | 18 | 22307958 | A | G | 0.563781 | -0.00673031 | 0.00119781 | 1.89998E-08 | 0.0142 | 0.0094 | 0.129 | 6.89476E-05 | 31.57129315 |
| rs17023019 | 3 | 85614272 | G | A | 0.647408 | 0.0121208 | 0.00124196 | 1.69981E-22 | -0.0256 | 0.0097 | 0.00830195 | 0.000207975 | 95.24571712 |
| rs17024335 | 2 | 101066927 | C | T | 0.223585 | -0.00828937 | 0.00143076 | 6.90001E-09 | 0.0019 | 0.0112 | 0.8659 | 7.33048E-05 | 33.56661698 |
| rs17409597 | 2 | 146045060 | C | T | 0.491526 | -0.0069862 | 0.00119428 | 4.90004E-09 | 0.0022 | 0.0093 | 0.8128 | 7.47295E-05 | 34.21903933 |
| rs17461712 | 11 | 16301315 | C | T | 0.167426 | 0.0120892 | 0.00159479 | 3.40017E-14 | 0.0263 | 0.0125 | 0.0350098 | 0.000125484 | 57.46272541 |
| rs17513240 | 18 | 53208982 | T | A | 0.172466 | -0.0106455 | 0.00156881 | 1.20005E-11 | 0.0363 | 0.0123 | 0.00304299 | 0.000100555 | 46.04574813 |
| rs1997468 | 2 | 77171297 | T | C | 0.439876 | 0.0108619 | 0.0011942 | 9.3994E-20 | -0.0107 | 0.0094 | 0.2532 | 0.000180648 | 82.72857994 |
| rs2032512 | 22 | 41071097 | C | A | 0.607796 | -0.00891807 | 0.00121481 | 2.09991E-13 | 0.0221 | 0.0095 | 0.0198999 | 0.000117687 | 53.89185595 |
| rs210600 | 7 | 69238281 | A | G | 0.27676 | 0.00767525 | 0.00132603 | 7.10003E-09 | -0.0048 | 0.0104 | 0.643701 | 7.31647E-05 | 33.50244203 |
| rs2113336 | 7 | 133478107 | A | C | 0.470426 | -0.00721001 | 0.00119169 | 1.40001E-09 | 0.016 | 0.0093 | 0.0856505 | 7.99402E-05 | 36.60523903 |
| rs2255015 | 3 | 183534976 | A | G | 0.454555 | -0.00697811 | 0.00119165 | 4.70002E-09 | -0.015 | 0.0093 | 0.1088 | 7.4886E-05 | 34.29069526 |
| rs2360802 | 8 | 53132694 | T | A | 0.225125 | -0.00942447 | 0.00142345 | 3.59998E-11 | 0.0146 | 0.0111 | 0.1907 | 9.57288E-05 | 43.83565923 |
| rs2472297 | 15 | 75027880 | T | C | 0.261771 | -0.0153851 | 0.00134465 | 2.60016E-30 | 0.0557 | 0.0105 | 1.11099E-07 | 0.000285833 | 130.912235 |
| rs2702576 | 4 | 15058245 | G | A | 0.621311 | 0.00741552 | 0.0012235 | 1.40001E-09 | -0.0211 | 0.0096 | 0.0273899 | 8.02224E-05 | 36.73443603 |
| rs2712661 | 12 | 99600297 | A | G | 0.769898 | -0.00823825 | 0.00140949 | 5.1E-09 | -0.0098 | 0.011 | 0.3752 | 7.46051E-05 | 34.16205905 |
| rs2728784 | 5 | 87624837 | G | A | 0.208554 | -0.0110087 | 0.00146285 | 5.30029E-14 | -0.032 | 0.0115 | 0.00550503 | 0.000123673 | 56.63313257 |
| rs2786529 | 1 | 98017090 | G | C | 0.209494 | -0.0083068 | 0.00145596 | 0.000000012 | 0.0152 | 0.0114 | 0.182 | 7.10874E-05 | 32.55117907 |
| rs2825972 | 21 | 21424129 | G | A | 0.292758 | 0.00875118 | 0.00130395 | 1.9002E-11 | 0.0067 | 0.0102 | 0.5142 | 9.83612E-05 | 45.04114883 |
| rs2952894 | 4 | 130738623 | C | T | 0.664339 | 0.00719966 | 0.00125775 | 0.00000001 | -0.013 | 0.0099 | 0.1855 | 7.15582E-05 | 32.76675442 |
| rs2952894 | 4 | 130738623 | C | T | 0.664339 | 0.00719966 | 0.00125775 | 0.00000001 | -0.0324 | 0.0486 | 0.5051 | 7.15582E-05 | 32.76675442 |
| rs3117103 | 6 | 32349557 | T | A | 0.139554 | -0.0152595 | 0.00170721 | 4.00037E-19 | 0.0418 | 0.0133 | 0.001702 | 0.000174456 | 79.8922805 |
| rs3132487 | 6 | 31243008 | T | G | 0.345911 | -0.00964644 | 0.00128001 | 4.79954E-14 | -0.0001 | 0.012 | 0.9946 | 0.000124025 | 56.79439958 |
| rs3132487 | 6 | 31243008 | T | G | 0.345911 | -0.00964644 | 0.00128001 | 4.79954E-14 | -0.0025 | 0.01 | 0.8037 | 0.000124025 | 56.79439958 |
| rs34292254 | 2 | 157050759 | T | G | 0.552492 | 0.0074428 | 0.0011946 | 4.70002E-10 | 0.0161 | 0.0094 | 0.0851805 | 8.47707E-05 | 38.81734076 |
| rs34759521 | 14 | 68351707 | A | T | 0.227087 | -0.00825854 | 0.00141691 | 5.60003E-09 | -0.0057 | 0.0111 | 0.604301 | 7.41899E-05 | 33.97192256 |
| rs34935263 | 16 | 74217860 | G | A | 0.533192 | -0.00681729 | 0.00118993 | 0.00000001 | -0.0074 | 0.0093 | 0.4271 | 7.16811E-05 | 32.82304081 |
| rs35968894 | 7 | 91762145 | G | A | 0.377461 | -0.00721604 | 0.00122465 | 3.79997E-09 | -0.022 | 0.0096 | 0.02151 | 7.58221E-05 | 34.71938256 |
| rs4410790 | 7 | 17284577 | C | T | 0.630852 | -0.0109577 | 0.0012289 | 4.79954E-19 | 0.0226 | 0.0096 | 0.0191501 | 0.000173614 | 79.50671809 |
| rs4452038 | 18 | 44856529 | T | C | 0.389867 | -0.0111751 | 0.00122327 | 6.4998E-20 | -0.0086 | 0.0096 | 0.3674 | 0.000182236 | 83.45575474 |
| rs4477562 | 13 | 54104968 | T | C | 0.1287 | -0.0104703 | 0.00178552 | 4.49997E-09 | 0.0221 | 0.014 | 0.114 | 7.5095E-05 | 34.38641621 |
| rs4492837 | 11 | 127079218 | C | G | 0.586887 | -0.00679179 | 0.00120606 | 1.79999E-08 | 0.0161 | 0.0094 | 0.0875709 | 6.92557E-05 | 31.71237537 |
| rs55880962 | 18 | 40659360 | A | G | 0.192325 | 0.00848655 | 0.00150523 | 0.000000017 | -0.0013 | 0.0117 | 0.912 | 6.94195E-05 | 31.78737905 |
| rs58391518 | 7 | 107030787 | C | T | 0.291369 | -0.0073709 | 0.00130713 | 0.000000017 | -0.034 | 0.0102 | 0.000903608 | 6.9443E-05 | 31.79812973 |
| rs58726064 | 17 | 42847033 | G | C | 0.442086 | 0.00677132 | 0.00119583 | 0.000000015 | -0.0079 | 0.0094 | 0.4007 | 7.00217E-05 | 32.06312813 |
| rs6045331 | 20 | 1885099 | G | C | 0.37918 | -0.00759061 | 0.00122681 | 6.1E-10 | 0.0067 | 0.0096 | 0.4845 | 8.36021E-05 | 38.28219621 |
| rs61909866 | 11 | 122061369 | T | C | 0.316065 | -0.00734016 | 0.00127652 | 8.9E-09 | 0.0093 | 0.01 | 0.3493 | 7.2207E-05 | 33.0638871 |
| rs62134736 | 2 | 46766446 | A | G | 0.2591 | 0.00757696 | 0.00135104 | 0.00000002 | 0.0016 | 0.0106 | 0.8789 | 6.86877E-05 | 31.45224699 |
| rs62403110 | 6 | 18951131 | A | G | 0.302512 | -0.00743216 | 0.00129009 | 8.40001E-09 | 0.0002 | 0.0101 | 0.9869 | 7.24792E-05 | 33.18854296 |
| rs62572325 | 9 | 22535878 | A | C | 0.13467 | 0.0112111 | 0.00173948 | 1.2E-10 | -0.003 | 0.0136 | 0.8268 | 9.07138E-05 | 41.53896548 |
| rs62580693 | 9 | 83351536 | C | G | 0.252484 | 0.00804118 | 0.0013674 | 4.09996E-09 | 0.0118 | 0.0107 | 0.2686 | 7.55215E-05 | 34.58171438 |
| rs6469 | 6 | 32008451 | T | C | 0.127478 | 0.0109331 | 0.00193107 | 0.000000015 | -0.0227 | 0.0151 | 0.133 | 7.00028E-05 | 32.05447808 |
| rs6736362 | 2 | 219115108 | T | C | 0.560656 | -0.00664831 | 0.00119534 | 2.69998E-08 | -0.0016 | 0.0094 | 0.8647 | 6.75562E-05 | 30.9341168 |
| rs6876382 | 5 | 167637012 | G | C | 0.05895 | -0.0151419 | 0.00252443 | 0.000000002 | 0.0037 | 0.0198 | 0.8516 | 7.85697E-05 | 35.97759968 |
| rs6892119 | 5 | 107142920 | G | A | 0.574513 | -0.0080653 | 0.00122024 | 3.90032E-11 | 0.0037 | 0.0095 | 0.6996 | 9.54034E-05 | 43.68663971 |
| rs74384251 | 2 | 63526179 | T | C | 0.136886 | 0.00952526 | 0.00172669 | 3.50002E-08 | -0.032 | 0.0135 | 0.0172199 | 6.64586E-05 | 30.43147712 |
| rs79564740 | 1 | 210027359 | C | T | 0.030417 | -0.019251 | 0.00345293 | 0.000000025 | 0.0334 | 0.027 | 0.2172 | 6.78823E-05 | 31.08344477 |
| rs826848 | 12 | 39156767 | T | C | 0.933713 | -0.014781 | 0.00238831 | 6.1E-10 | -0.0191 | 0.0188 | 0.3097 | 8.36459E-05 | 38.30225496 |
| rs888405 | 3 | 64201702 | G | A | 0.794793 | 0.00919625 | 0.00146844 | 3.79997E-10 | 0.0055 | 0.0115 | 0.6295 | 8.56499E-05 | 39.21996745 |
| rs9372734 | 6 | 98577689 | T | C | 0.482495 | -0.00802444 | 0.00118828 | 1.39991E-11 | 0.002 | 0.0093 | 0.833 | 9.95872E-05 | 45.60264113 |
| rs9570736 | 13 | 63369166 | G | A | 0.518982 | -0.00655673 | 0.00118731 | 3.29997E-08 | 0.024 | 0.0093 | 0.00966407 | 6.65997E-05 | 30.49611047 |
| rs9835772 | 3 | 85766025 | T | A | 0.243662 | -0.00807999 | 0.00138074 | 4.90004E-09 | 0.0198 | 0.0108 | 0.0668005 | 7.4786E-05 | 34.24490269 |
| Genome-wide significant SNPs for Seen a psychiatrist for nerves, anxiety, tension or depression | | | | | | | | | | | | | |
| SNP | Chr | Position | EA | OA | EAF | Seen a psychiatrist for nerves, anxiety, tension or depression | | | Knee Osteoarthritis | | | R2 | F |
|  |  |  |  |  |  | beta | SE | pval | beta | SE | pval |  |  |
| rs10809465 | 9 | 11409171 | C | T | 0.637767 | -0.00387614 | 0.000694083 | 2.30001E-08 | -0.0041 | 0.0097 | 0.6699 | 6.76903E-05 | 31.18704372 |
| rs11210177 | 1 | 73655821 | G | A | 0.501825 | 0.0042095 | 0.000667464 | 2.90001E-10 | -0.0044 | 0.0093 | 0.6383 | 8.63272E-05 | 39.77438276 |
| rs1264690 | 6 | 30195038 | T | G | 0.163433 | -0.00694275 | 0.000899793 | 1.20005E-14 | 0.0411 | 0.0125 | 0.00099731 | 0.000129212 | 59.5354922 |
| rs143502921 | 20 | 59827231 | C | G | 0.179539 | 0.00616455 | 0.00097128 | 2.19999E-10 | -0.0022 | 0.0133 | 0.8701 | 8.74291E-05 | 40.28208869 |
| rs3129962 | 6 | 32379383 | A | G | 0.12934 | -0.00665779 | 0.000992351 | 1.99986E-11 | 0.0456 | 0.0138 | 0.000927107 | 9.76938E-05 | 45.0119343 |
| rs3129962 | 6 | 32379383 | A | G | 0.12934 | -0.00665779 | 0.000992351 | 1.99986E-11 | -0.0267 | 0.0117 | 0.02227 | 9.76938E-05 | 45.0119343 |
| rs942866 | 14 | 104014935 | T | G | 0.655767 | 0.0042643 | 0.000705959 | 1.5E-09 | -0.0175 | 0.0098 | 0.0740798 | 7.91921E-05 | 36.48670597 |
| Genome-wide significant SNPs for Metabolic disorders | | | | | | | | | | | | | |
| SNP | Chr | Position | EA | OA | EAF | Metabolic disorders | | | Knee Osteoarthritis | | | R2 | F |
|  |  |  |  |  |  | beta | SE | pval | beta | SE | pval |  |  |
| rs114483871 | 4 | 73990168 | C | T | 0.0359332 | 0.128095 | 0.0193875 | 3.92013E-11 | -0.0219 | 0.0311 | 0.4815 | 0.000108277 | 43.65345074 |
| rs115478735 | 9 | 136149711 | A | T | 0.198546 | 0.0682256 | 0.00911963 | 7.36546E-14 | 0.0336 | 0.0126 | 0.00769396 | 0.000138817 | 55.96778708 |
| rs11591147 | 1 | 55505647 | G | T | 0.0359342 | -0.280953 | 0.0209989 | 7.97995E-41 | -0.0884 | 0.0351 | 0.0116799 | 0.000443857 | 179.0078606 |
| rs11648003 | 16 | 72052348 | A | G | 0.22216 | 0.0488357 | 0.00876897 | 2.55988E-08 | 0.0045 | 0.0112 | 0.686 | 7.69317E-05 | 31.01526607 |
| rs1260326 | 2 | 27730940 | T | C | 0.650744 | -0.043663 | 0.00769498 | 1.39329E-08 | -0.0245 | 0.0095 | 0.00981206 | 7.98617E-05 | 32.19659899 |
| rs12740374 | 1 | 109817590 | G | T | 0.2144 | -0.0969052 | 0.00904843 | 9.17487E-27 | -0.0032 | 0.0112 | 0.7722 | 0.000284437 | 114.6952805 |
| rs12916 | 5 | 74656539 | T | C | 0.453515 | 0.0486016 | 0.00736348 | 4.10204E-11 | 0.0216 | 0.0095 | 0.0227499 | 0.000108056 | 43.5645022 |
| rs1367117 | 2 | 21263900 | G | A | 0.280596 | 0.0747818 | 0.00808848 | 2.34207E-20 | 0.0161 | 0.0098 | 0.1013 | 0.000211996 | 85.4782933 |
| rs1556516 | 9 | 22100176 | G | C | 0.421516 | 0.0551722 | 0.00741034 | 9.67386E-14 | -0.0165 | 0.0093 | 0.0754501 | 0.000137488 | 55.43220823 |
| rs17248720 | 19 | 11198187 | C | T | 0.101285 | -0.169454 | 0.0125453 | 1.41449E-41 | -0.01 | 0.0144 | 0.4869 | 0.000452383 | 182.4481201 |
| rs185567543 | 4 | 74990001 | A | T | 0.0218115 | 0.201662 | 0.0243476 | 1.20504E-16 | -0.043 | 0.0692 | 0.5345 | 0.000170147 | 68.60150902 |
| rs1883711 | 20 | 39179822 | G | C | 0.0623769 | 0.10864 | 0.0149761 | 4.04017E-13 | -0.0385 | 0.027 | 0.1532 | 0.000130523 | 52.62352022 |
| rs2954021 | 8 | 126482077 | A | G | 0.535277 | -0.0708875 | 0.00733599 | 4.33112E-22 | -0.0011 | 0.0093 | 0.9089 | 0.00023157 | 93.37265429 |
| rs3005923 | 1 | 56801542 | G | A | 0.0259031 | -0.167509 | 0.0241984 | 4.44324E-12 | -0.0442 | 0.0522 | 0.3969 | 0.000118854 | 47.91823738 |
| rs4299376 | 2 | 44072576 | G | T | 0.783983 | -0.0664367 | 0.00887089 | 6.92468E-14 | -0.0165 | 0.0099 | 0.09553 | 0.000139118 | 56.08924085 |
| rs499883 | 1 | 55519174 | G | A | 0.528693 | 0.0611241 | 0.0073917 | 1.34772E-16 | 0.0117 | 0.0097 | 0.2282 | 0.000169599 | 68.38079348 |
| rs7412 | 19 | 45412079 | C | T | 0.0534036 | -0.269683 | 0.0171177 | 6.38117E-56 | -0.0419 | 0.017 | 0.0138899 | 0.000615333 | 248.2070799 |
| rs77645768 | 4 | 73668828 | G | A | 0.0269055 | 0.171995 | 0.022149 | 8.13954E-15 | -0.0462 | 0.0312 | 0.1383 | 0.000149561 | 60.30054576 |
| rs8263 | 17 | 56423114 | C | T | 0.490534 | 0.0413894 | 0.00735283 | 1.81222E-08 | -0.0095 | 0.0093 | 0.3081 | 7.85953E-05 | 31.68597365 |
| rs9457906 | 6 | 160759553 | C | G | 0.0122608 | 0.216709 | 0.0320202 | 1.30677E-11 | 0.0137 | 0.0336 | 0.684301 | 0.00011361 | 45.80402691 |
| rs964184 | 11 | 116648917 | G | C | 0.855014 | -0.113521 | 0.0102369 | 1.41286E-28 | 0.0001 | 0.0137 | 0.9953 | 0.000304961 | 122.9740114 |
