# Supplementary material for: Causal relationship between modifiable risk factors and knee osteoarthritis: a Mendelian randomization study
Source: Front Med (Lausanne). 2024 Sep 2;11:1405188. doi: 10.3389/fmed.2024.1405188 (PMC11402680; doi:10.3389/fmed.2024.1405188)
Supplement: Supplementary file 5 [file Table_4.docx]

| **Supplementary Table 4.SNP data summaries with incompatible alleles and palindromic structures were removed** | | | |
| --- | --- | --- | --- |
| Trait | GWAS ID | SNPs for incompatible alleles | SNPs for being palindromic with intermediate allele frequencies |
| Hypothyroidism, unspecified | ukb-b-4226 | NA | NA |
| Hyperthyroidism/thyrotoxicosis | ukb-b-20289 | NA | NA |
| Average total household income before tax | ukb-b-7408 | rs1239705 | rs11165472, rs1455350, rs3130264 |
| Never eat eggs, dairy, wheat, sugar: Wheat products | ukb-b-3599 | NA | NA |
| Never eat eggs, dairy, wheat, sugar: Sugar or foods/drinks containing sugar | ukb-b-5495 | NA | rs3756362 |
| Standing height | ukb-a-389 | rs11252860 | rs10817161, rs11233117, rs11664336, rs12572775, rs134092, rs1467847, rs1990656, rs35943760, rs3812163, rs41369549, rs4901548, rs7090035, rs7102462, rs7652177, rs8181166 |
| Standing height | ukb-b-10787 | rs1019075, rs11051456, rs11252860, rs1985278, rs2138628, rs34773647, rs664317, rs7978217 | rs10046853, rs10228350, rs10811092, rs11664336, rs12572775, rs13102005, rs1467847, rs153661, rs3771382, rs3790086, rs3812163, rs418280, rs4302014, rs5759006, rs6464921, rs7652177, rs7847059, rs832806, rs9352895, rs9976812 |
| Essential (primary) hypertension | ukb-b-12493 | NA | rs1870735, rs35184780 |
| Age completed full time education | ukb-b-6134 | NA | rs10786662, rs7335432 |
| Years of schooling | ieu-a-1239 | rs510706 | rs12134151, rs13130765, rs1455350, rs2414072, rs2478208, rs2545798, rs320693, rs401687, rs6867851, rs7920624 |
| Hot drink temperature | ukb-b-14203 | rs2952894, rs3132487 | rs12038134, rs58726064 |
| Seen a psychiatrist for nerves, anxiety, tension or depression | ukb-b-18336 | rs3129962 | NA |
| Metabolic disorders | finngen_R8_E4_METABOLIA | NA | rs1556516 |
