# Supplementary material for: Causal relationship between modifiable risk factors and knee osteoarthritis: a Mendelian randomization study
Source: Front Med (Lausanne). 2024 Sep 2;11:1405188. doi: 10.3389/fmed.2024.1405188 (PMC11402680; doi:10.3389/fmed.2024.1405188)
Supplement: Supplementary file 6 [file Table_5.docx]

| **Supplementary Table 5.Risk factors for the results of MR Analysis of KOA** | | | | | | | | | |  |  |
| --- | --- | --- | --- | --- | --- | --- | --- | --- | --- | --- | --- |
| Trait | SNPs | IVW |  | MR-Egger |  | Weighted median |  | Simple mode |  | Weighted mode |  |
|  |  | OR(95%CI) | P | OR(95%CI) | P | OR(95%CI) | P | OR(95%CI) | P | OR(95%CI) | P |
| Hypothyroidism, unspecified | 36 | 5.56(1.22-25.32) | 0.026 | 26.39(0.53-1314.14) | 0.11 | 7.98(1.33-47.84) | 0.023 | 25.05(0.61-1033.77) | 0.099 | 12.11(0.95-154.94) | 0.063 |
| Hyperthyroidism/thyrotoxicosis | 13 | 711.17(49.38-10242.78) | 1.3978E-06 | 684.49(4.73-99031.32) | 0.026 | 538.04(17.49-16543.49） | 0.0003 | 544.21(2.26-131196.25) | 0.044 | 604.43(20.48-17838.44) | 0.003 |
| Average total household income before tax | 45 | 0.69（0.58-0.82） | 4.06265E-05 | 0.55（0.26-1.18） | 0.133 | 0.78（0.62-0.99） | 0.038 | 0.44(0.24-0.79） | 0.009 | 0.89（0.54-2.49） | 0.676 |
| Never eat eggs, dairy, wheat, sugar: Wheat products | 5 | 23.89（5.13-111.22） | 5.256E-05 | 174.14（2.23-13550.97） | 0.103 | 21.86（3.53-111.21) | 0.0009 | 5.37(0.3-96.05) | 0.317 | 20.99(2.84-155.04) | 0.041 |
| Never eat eggs, dairy, wheat, sugar: Sugar or foods/drinks containing sugar | 20 | 6.95(1.83-26.42) | 0.004 | 283.91(0.14-579945.88) | 0.164 | 5.97(1.69-21.02) | 0.005 | 3.99(0.45-35.36) | 0.229 | 3.69(0.45-39.29) | 0.239 |
| Standing height | 590 | 1.07(1.006-1.15) | 0.031 | 1.02(0.88-1.17) | 0.806 | 1.09(1.007-1.18) | 0.032 | 1.15(0.87-1.52) | 0.317 | 1.03(0.83-1.28) | 0.777 |
| Standing height | 751 | 1.09(1.03-1.17) | 0.006 | 1.08(0.95-1.23) | 0.251 | 1.13(1.04-1.22) | 0.004 | 1.28(0.97-1.68) | 0.082 | 1.15(0.94-1.41) | 0.184 |
| Essential (primary) hypertension | 68 | 2.11(1.11-4.02) | 0.023 | 0.09(0.01-0.99) | 0.053 | 2.08(0.96-4.49) | 0.064 | 5.53(0.93-33.02) | 0.065 | 4.53(0.66-31.07) | 0.129 |
| Age completed full time education | 39 | 0.52(0.38-0.71) | 2.82848E-05 | 0.54(0.15-1.95) | 0.356 | 0.58(0.41-0.83) | 0.002 | 0.63(0.31-1.31) | 0.225 | 0.63(0.32-1.23) | 0.204 |
| Years of schooling | 307 | 0.58(0.52-0.64) | 3.25477E-24 | 0.56(0.37-0.86) | 0.007 | 0.57(0.49-0.66) | 1.67181E-14 | 0.57(0.35-0.93) | 0.025 | 0.54(0.36-0.79) | 0.002 |
| Hot drink temperature | 69 | 0.55(0.36-0.85) | 0.007 | 0.17(0.03-1.09) | 0.066 | 0.76(0.47-1.23) | 0.268 | 1.02(0.27-3.95) | 0.972 | 1.05(0.28-3.99) | 0.943 |
| Seen a psychiatrist for nerves, anxiety, tension or depression | 6 | 0.04(0.002-0.47) | 0.011 | 1.38E-05(1.46E-09-0.13) | 0.075 | 0.05(0.003-0.55) | 0.015 | 0.55（0.003-99.45） | 0.829 | 0.49（0.003-89.39) | 0.798 |
| Metabolic disorders | 20 | 1.13(1.04-1.22) | 0.003 | 1.07(0.92-1.24) | 0.375 | 1.11(1.01-1.22) | 0.023 | 1.13(0.96-1.32) | 0.151 | 1.12(1.02-1.23) | 0.03 |
