# Supplementary material for: Causal relationship between modifiable risk factors and knee osteoarthritis: a Mendelian randomization study
Source: Front Med (Lausanne). 2024 Sep 2;11:1405188. doi: 10.3389/fmed.2024.1405188 (PMC11402680; doi:10.3389/fmed.2024.1405188)
Supplement: Supplementary file 7 [file Table_6.docx]

**Supplementary Table 6.Description of the results of heterogeneity and pleiotropy analysis.**

| Trait | Heterogeneity | | | IVW (multiplicative random effects) | | | Pleiotropy | | | MR-PRESSO | |
| --- | --- | --- | --- | --- | --- | --- | --- | --- | --- | --- | --- |
|  | Q | Q_df | Q_pval | Beta | Se | P | Egger_intercept | Se | P | P-globe test | P-globe test（Correcting outliers） |
| Hypothyroidism, unspecified | 57.94 | 35 | 0.008 | 1.716 | 0.773 | 0.026 | -0.004864289 | 0.00573831 | 0.402 | 0.008 | 0.1 |
| Hyperthyroidism/thyrotoxicosis | 13.58 | 12 | 0.328 |  |  |  | 0.00011147 | 0.006131134 | 0.985 | 0.417 |  |
| Average total household income before tax | 53.69 | 44 | 0.15 |  |  |  | 0.004378664 | 0.007479095 | 0.561 | 0.072 |  |
| Never eat eggs, dairy, wheat, sugar: Wheat products | 4.11 | 4 | 0.391 |  |  |  | -0.017786264 | 0.018581009 | 0.409 | 0.525 |  |
| Never eat eggs, dairy, wheat, sugar: Sugar or foods/drinks containing sugar | 50.26 | 19 | 0.0001 | 1.938 | 0.682 | 0.004 | -0.020064308 | 0.020703831 | 0.345 | 0.0002 | 0.055 |
| Standing height | 1488.71 | 589 | 2.53E-79 | 7.20E-02 | 3.30E-02 | 3.10E-02 | 0.001336727 | 0.001560115 | 0.392 | <2e-04 | 0.952 |
| Standing height | 1652.25 | 750 | 8.72E-70 | 8.90E-02 | 3.30E-02 | 6.00E-03 | 0.00029999 | 0.001222661 | 0.806 | <2e-04 | 0.383 |
| Essential (primary) hypertension | 123.79 | 67 | 3.01E-05 | 7.46E-01 | 3.29E-01 | 2.30E-02 | 0.01712063 | 0.006320568 | 0.008 | <2e-04 | 0.012 |
| Age completed full time education | 66.09 | 38 | 0.003 | -0.65 | 0.155 | 2.83E-05 | -0.000601127 | 0.009508197 | 0.949 | 0.003 | 0.104 |
| Years of schooling | 460.06 | 306 | 2.71E-08 | -5.53E-01 | 5.40E-02 | 3.25E-24 | 0.000292771 | 0.002920382 | 0.92 | <2e-04 | 0.886 |
| Hot drink temperature | 169.56 | 68 | 1.21E-10 | -5.95E-01 | 2.22E-01 | 7.00E-03 | 0.010945419 | 0.008529686 | 0.203 | <2e-04 | 0.031 |
| Seen a psychiatrist for nerves, anxiety, tension or depression | 11.11 | 5 | 0.049 | -3.297 | 1.302 | 0.011 | 0.04161417 | 0.023918426 | 0.156 | 0.0024 | 0.504 |
| Metabolic disorders | 31.26 | 19 | 0.037 | 0.119 | 0.039 | 0.002 | 0.005658087 | 0.007044383 | 0.432 | 0.016 | 0.057 |
